# Phosphorylation tunes p62 condensates to drive autophagic degradation of ubiquitinated proteins

**DOI:** 10.1038/s44318-026-00785-1

**Published:** 2026-05-05

**Authors:** Satoko Komatsu-Hirota, Keisuke Tabata, Yu-shin Sou, Soichiro Kakuta, Jun-ichi Sakamaki, Hikaru Tsuchiya, Jiachen Li, Hiroyuki Kumeta, Yuji Sakai, Yuko Fujioka, Daisuke Noshiro, Shunsuke F Shimobayashi, Tomo Kurimura, Takashi Taniguchi, Manabu Abe, Masato Koike, Hideaki Morishita, Nobuo N Noda, Masaaki Komatsu

**Affiliations:** 1 Department of Physiology, Juntendo University Graduate School of Medicine, Tokyo, Japan; 2 Department of Cell Biology and Neuroscience, Juntendo University Graduate School of Medicine, Tokyo, Japan; 3 Laboratory of Morphology and Image Analysis, Biomedical Research Core Facilities, Juntendo University Graduate School of Medicine, Tokyo, Japan; 4 Autophagy Research Center, Juntendo University Graduate School of Medicine, Tokyo, Japan; 5 Frontier Research Center for Advanced Material and Life Science, Graduate School of Life Science and Faculty of Advanced Life Science, Hokkaido University, Sapporo, Japan; 6 Graduate School of Nanobioscience, Yokohama City University, Yokohama, Japan; 7 Institute for Genetic Medicine, Hokkaido University, Sapporo, Japan; 8 Department of Life Science Frontiers Center for iPS Cell Research and Application (CiRA), Kyoto University, Kyoto, Japan; 9 Graduate School of Engineering, Department of Chemical Engineering, Kyoto University, Kyoto, Japan; 10 Department of Animal Model Development, Brain Research Institute, Niigata University, Niigata, Japan; 11 Department of Molecular Cell Biology, Graduate School of Medical Sciences, Kyushu University, Fukuoka, Japan

**Keywords:** Organelles, Post-translational Modifications & Proteolysis

## Abstract

p62/SQSTM1 self-assembles with polyubiquitin into liquid-like condensates (“p62 bodies”) that function as stress-signaling hubs and selective autophagy cargo. We show that TBK1-dependent phosphorylation at Ser403 acts as a threshold-dependent modulator of a condensate’s physical properties and promotes their rapid autophagic clearance. Phosphorylation within p62 bodies drives a transition from large, fluid droplets to compact, gel-like condensates that efficiently capture LC3-positive isolation membranes and accelerate the autophagic removal of ubiquitinated proteins. PP2A holoenzymes containing PPP2R5A/B/E, recruited via a KEAP1 bridge, counteract TBK1 by dephosphorylating Ser403. Homozygous p62S403E/S403E knock-in embryonic stem cells differentiate into post-mitotic neurons enriched in miniaturized, gel-like p62 bodies. Consistently, phosphorylation-mimetic knock-in mice show similar remodeling of p62 condensates in vivo, demonstrating that this phosphorylation-driven mechanism maintains proteostasis across scales. We propose that Ser403 phosphorylation functions as a molecular switch that couples the material state of p62 condensates to their stability and serves as a central control point for p62-mediated protein degradation.

## Synopsis

p62/SQSTM1 forms phase-separated condensates that function in both stress signaling and selective autophagy, yet whether material state affects degradability remains unclear. This article shows that phosphorylation of p62 at Ser403, switches condensates from fluid to compact, gel-like structures that are efficiently cleared by autophagy.

- TBK1 and PP2A regulate p62 Ser403 phosphorylation status within condensates.
- Ser403 phosphorylation induces a transition from fluid to compact, gel-like p62 condensates.
- Phosphorylated p62 condensates exhibit reduced mobility, increased surface tension, and enhanced autophagic degradation.
- This phosphorylation-dependent mechanism operates in cells and in vivo, producing smaller and more numerous p62 bodies that enhance proteostasis.

## Abstract (web summary)

Phosphorylation of p62 by TBK1 switches condensates to a more compact, degradable state.

## Graphical abstract

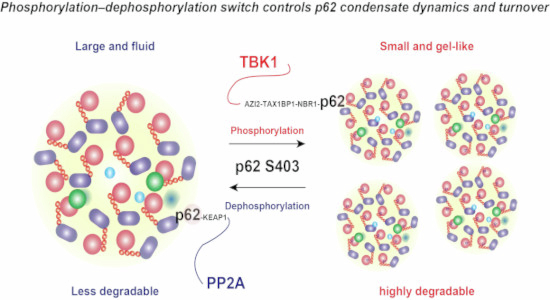

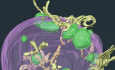

## Introduction

Cells utilize biomolecular condensates formed via liquid–liquid phase separation (LLPS) to spatially compartmentalize key biochemical reactions without membrane boundaries. Such condensates provide a dynamic framework for regulating signaling, metabolism, and protein quality control (Banani et al, 2017; Noda et al, 2020; Shin and Brangwynne, 2017). However, despite their functional versatility, the degradability of LLPS-derived structures remains a critical question, especially in the context of proteostasis and cellular homeostasis. Recent studies suggest that only a subset of condensates are efficiently cleared by autophagy (Kuno et al, 2022; Ohshima et al, 2022; Turakhiya et al, 2018; Wilfling et al, 2020; Yamasaki et al, 2020; Zhang et al, 2018), but the molecular basis underlying this selectivity is largely unknown.

Among known autophagy-related condensates, p62 bodies represent a paradigmatic example of phase-separated structures that are both stress-responsive and degradable (Kageyama et al, 2021; Sun et al, 2018; Zaffagnini et al, 2018). These cytoplasmic puncta are formed by multivalent interactions between the selective autophagy receptor p62/SQSTM1 and polyubiquitinated proteins, and are eventually engulfed by autophagosomes. Structurally, p62 contains multiple modular domains—a PB1 domain for self-oligomerization (Ciuffa et al, 2015; Jakobi et al, 2020), a UBA domain for ubiquitin binding (Matsumoto et al, 2011; Pilli et al, 2012), a FIR for FIP200 interaction and a LIR motif for LC3 interaction (Ichimura et al, 2008; Pankiv et al, 2007)—that facilitate its role as a scaffold for both condensate formation and autophagic targeting.

Intriguingly, p62 bodies are now recognized as multifunctional condensates rather than mere depots for selective autophagic degradation. The best-characterized activity of p62 bodies is their role as signaling hubs: under proteotoxic stress, p62 bodies sequester the E3 adapter KEAP1, thereby stabilizing the master antioxidant transcription factor NRF2 and inducing antioxidant genes (Ichimura et al, 2013; Ikeda et al, 2023). Recent work further shows that, when translation initiation is blocked by mTORC1 inhibition or amino-acid starvation, p62 bodies capture the initiation factors eIF2α and eIF4E (Danieli et al, 2023), leading to a transient repression of global protein synthesis. This dual function—as both a signaling hub and a degradable condensate—raises a fundamental question: how do cells switch the functional state of p62 bodies between signaling activation and degradative clearance? One plausible mechanism involves post-translational modifications that regulate p62’s molecular interactions and condensate properties. In particular, phosphorylation at Ser403 within the UBA domain—by TBK1, CK2, or ULK1—has been shown to increase p62’s affinity for ubiquitin chains (Lim et al, 2015; Matsumoto et al, 2011; Pilli et al, 2012). It is therefore conceivable that Ser403 phosphorylation also remodels condensate size and fluidity, thereby licensing their autophagic degradation, but this possibility remains to be tested.

Here, we demonstrate that Ser403 phosphorylation functions not merely to enhance ubiquitin binding, but rather as a biophysical and functional switch that governs the autophagic fate of p62 bodies. We identify a TBK1–PP2A axis, involving PPP2R5 regulatory subunits, that dynamically controls the phosphorylation status of p62. Phosphorylation drives a transition toward smaller, less fluid condensates that exhibit enhanced recruitment of autophagy machinery and are more readily degraded. Moreover, using a physiological model of neuronal differentiation and in phosphorylation-mimetic *p62*^*S403E/S403E*^ knock-in mice, we show that this mechanism operates even under basal, non-stressed conditions, thereby ensuring developmental and tissue proteostasis. Our findings establish Ser403 phosphorylation as a determinant of p62 body function and fate, revealing a tunable mechanism by which cells regulate the balance between signaling and degradation within phase-separated compartments.

## Results

### Localization of TBK1 within p62 bodies and phosphorylation of p62

**Figure 1:**
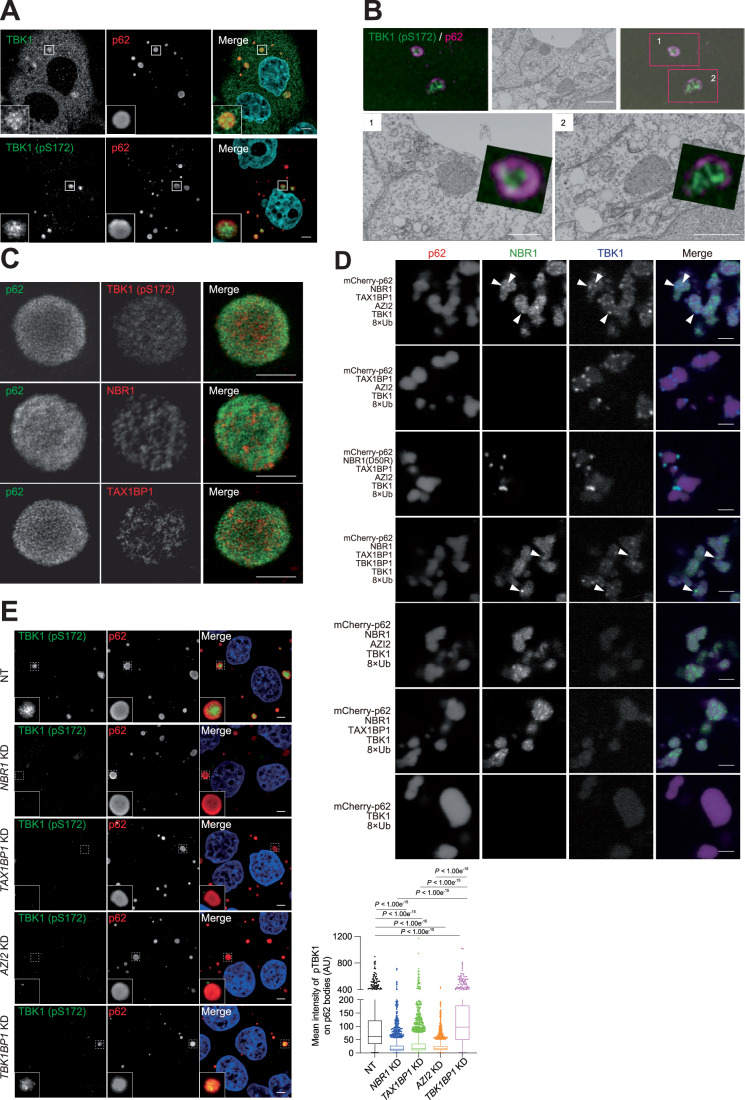
TBK1 is recruited to p62 bodies via the TBK1BP1–AZI2–TAX1BP1–NBR1 axis. (**A**) Immunofluorescence microscopy of Huh-1 cells stained with indicated antibodies. Scale bars, 5 µm. (**B**) Correlative light and electron microscopy (CLEM) showing colocalization of endogenous pS172-TBK1 (green) and p62 (magenta). Scale bars: overview, 3 µm; inset 1, 1 µm; inset 2, 2 µm. (**C**) STED imaging of endogenous pS172-TBK1, NBR1, and TAX1BP1 within p62 bodies. Scale bars, 1 µm. (**D**) In vitro reconstitution assay. mCherry-p62 (10 µM) and SNAP-8×Ub were mixed with SNAP-TBK1 (Alexa Fluor 649) in the presence or absence of NBR1, NBR1^D50R^, TAX1BP1, AZI2, and TBK1BP1. White arrowheads indicate TBK1 puncta within p62 bodies. Scale bars, 2 µm. (**E**) Immunofluorescence of siRNA-treated Huh-1 cells stained for pS172-TBK1 and p62. Fluorescence intensity of pS172-TBK1 on individual p62 bodies is plotted for non-targeting control (NT) (*n* = 2564), *NBR1*knockdown (*n* = 2553), *TAX1BP1*-knockdown (*n* = 3164), *AZI2*-knockdown (*n* = 2230) and *TBK1BP1*knockdown (*n* = 1702) cells. Statistics: one-way ANOVA followed by Šidák’s multiple-comparison test. Exact *P* values are indicated; values below the detection limit are reported as *P* < 1 × 10⁻¹⁵. Horizontal bars indicate medians; whiskers indicate the minimum and maximum values; boxes indicate the interquartile range. Scale bar, 5 µm. Source data are available online for this figure.

**Figure EV1:**
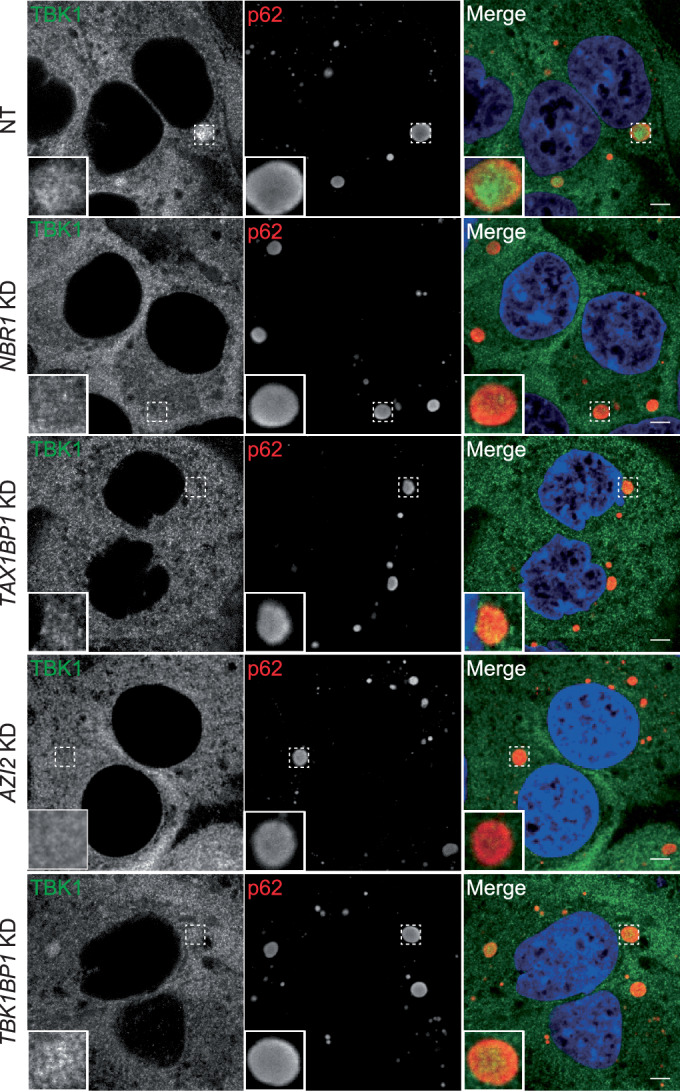
TBK1 localizes to p62 bodies. Immunofluorescence microscopy. Huh-1 cells transfected with siRNAs targeting *NBR1*, *TAX1BP1*, *AZI2*, or *TBK1BP1* were stained with antibodies against p62 and TBK1. Scale bars, 5 µm. Source data are available online for this figure.

To investigate the localization of serine/threonine-protein kinase TBK1 in p62 bodies, we performed double immunofluorescence analysis using antibodies against TBK1 or phosphorylated Ser172 of TBK1 (TBK1 pS172), the active form of TBK1 (Ma et al, 2012), and p62. As shown in Fig. 1A, both TBK1 and its active form were detected in the p62 bodies of Huh-1 cells, which exhibit high levels of endogenous p62 bodies. While total TBK1 appeared broadly distributed within condensates, pTBK1 signals were more spatially restricted, suggesting localized activation within defined subdomains. Correlative light and electron microscopy (CLEM) confirmed that TBK1 pS172 localized to filamentous round structures previously identified as p62 bodies (Berkamp et al, 2024; Jakobi et al, 2020) (Fig. 1B). To further resolve its spatial organization, Stimulated Emission Depletion (STED) nanoscopy demonstrated that pTBK1 signals appeared as discrete puncta rather than a uniform distribution within condensates (Fig. 1C). TBK1 is thought to translocate to p62 bodies via TBK1 adaptor proteins, 5-azacytidine-induced protein 2 (AZI2) and TANK-binding kinase 1-binding protein 1 (TBK1BP1), as these proteins can interact with selective autophagy receptor proteins, including Tax1-binding protein 1 (TAX1BP1) and Calcium-binding and coiled-coil domain-containing protein 2 (CALCOCO2) (Adriaenssens et al, 2024; Bauer et al, 2024; Fu et al, 2018), which localize to p62 bodies. TAX1BP1 also has the ability to bind to next to BRCA1 gene 1 protein (NBR1) (Bauer et al, 2024; North et al, 2025; Turco et al, 2021). Notably, the signal pattern of TBK1 closely resembled those of selective autophagy receptor proteins, including NBR1 and TAX1BP1 (Fig. 1C). An in vitro reconstitution assay using recombinant p62, NBR1, TAX1BP1, AZI2, TBK1, and octa-linear ubiquitin chains (8xUb) demonstrated the formation of p62 bodies and the recruitment of TBK1 into them (Fig. 1D). The TBK1 signal appeared as puncta within the p62 bodies, consistent with cellular observations (Fig. 1C). When either TAX1BP1 or AZI2 was depleted, TBK1-positive puncta were not observed, and instead, a markedly weak but diffuse TBK1 signal was detected within the p62 bodies (Fig. 1D). In contrast, upon depletion of NBR1, TBK1-positive puncta were still present but localized on the surface of the p62 bodies (Fig. 1D). Similarly, when the NBR1^D50R^ mutant, which is unable to bind p62, was used in place of wild-type NBR1, TBK1-positive puncta were also observed on the surface of the p62 bodies (Fig. 1D). These findings suggest that the interaction between p62 and NBR1 is required for TBK1-positive structures to penetrate into p62 bodies. Notably, TBK1-positive puncta in the p62 bodies were also observed when AZI2 was substituted with TBK1BP1 (Fig. 1D), although the frequency of TBK1 puncta formation in p62 bodies was lower compared to when AZI2 was present. To further examine the requirement for NBR1, TAX1BP1, AZI2, and TBK1BP1 in TBK1 translocation, we performed knockdown experiments in Huh-1 cells. Depletion of *NBR1*, *TAX1BP1*, or *AZI2* strongly suppressed the recruitment of TBK1 and TBK1 pS172 to p62 bodies compared with control siRNA, whereas *TBK1BP1* knockdown produced only a modest reduction (Figs. 1E and  EV1). These results indicate that the selective autophagy adaptors NBR1 and TAX1BP1 are essential for TBK1 localization, and that among the TBK1 kinase adaptors, AZI2—but not TBK1BP1—plays a dominant role in targeting TBK1 to p62 bodies.

**Figure 2:**
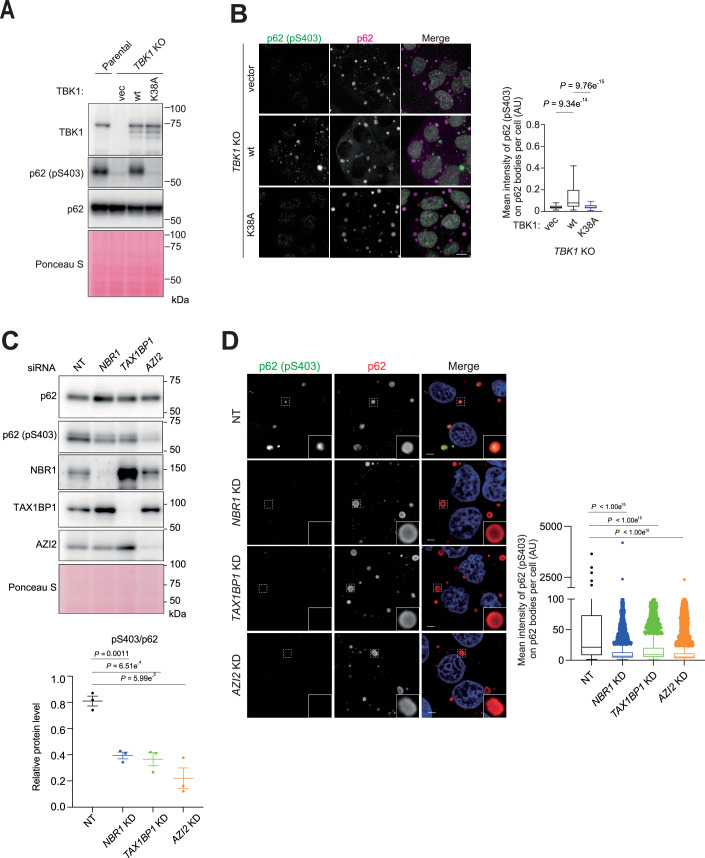
TBK1 phosphorylates p62 at Ser403 within p62 bodies. (**A**) Immunoblot of *TBK1*-null Huh-1 cells stably expressing empty vector, wild-type TBK1, or the kinase-dead mutant TBK1^K38A^. (**B**) Immunofluorescence of the cells in (A) stained for Ser403- phosphorylated p62 (pS403-p62) and total p62. Fluorescence intensity of pS403-p62 on individual p62 bodies was quantified for cells carrying the empty vector (*n* = 589), TBK1 (*n* = 857), or TBK1^K38A^ (*n* = 1183). Statistics: one-way ANOVA with Šidák’s multiple-comparison test. Horizontal bars indicate medians; boxes indicate the interquartile range (25th–75th percentiles); whiskers extend to 1.5× the interquartile range. Individual data points outside this range are plotted as outliers. Scale bar, 10 µm. (**C**) Immunoblot of Huh-1 cells treated with siRNAs targeting *NBR1*, *TAX1BP1*, or *AZI2*. Densitometric ratios of pS403-p62 to total p62 are shown (*n* = 3; mean ± s.e.m.). Statistics: one-way ANOVA followed by Tukey’s test. (**D**) Immunofluorescence of the cells in (**C**) stained for pS403-p62 and p62. Quantified pS403-p62 fluorescence on p62 bodies for non-targeting control (*n* = 1031), *NBR1* knockdown (*n* = 6712), *TAX1BP1* knockdown (*n* = 6122), and *AZI2* knockdown (*n* = 6349) cells. Statistics: one-way ANOVA with Šidák’s test. Exact *P* values are indicated; values below the detection limit are reported as *P* < 1 × 10⁻¹⁵. Horizontal bars indicate medians; boxes indicate the interquartile range (25th–75th percentiles); whiskers extend to 1.5× the interquartile range. Individual data points outside this range are plotted as outliers. Scale bar, 5 µm. Source data are available online for this figure.

To examine whether TBK1 phosphorylates p62 at Ser403 within p62 bodies, we generated *TBK1*-deficient Huh-1 cells (Appendix Fig. S1A). Consistent with previous reports (Matsumoto et al, 2015; Pilli et al, 2012), deletion of TBK1 completely abolished Ser403 phosphorylation of p62 (Fig. 2A). Re-expression of wild-type TBK1, but not the kinase-dead mutant TBK1^K38A^ (Pomerantz and Baltimore, 1999), restored Ser403 phosphorylation (Fig. 2A). Given that TBK1 and its active form localize to p62 bodies (Fig. 1A), these results suggest that TBK1 phosphorylates p62 at Ser403 within p62 bodies. In support of this, the signal intensity of Ser403-phosphorylated p62 in p62 bodies was restored by wild-type TBK1 expression, but not by TBK1^K38A^, in *TBK1*-deficient cells (Fig. 2B). Moreover, the level of p62 phosphorylation at Ser403, relative to total p62, was significantly reduced in *NBR1*- and *TAX1BP1*-knockdown cells (Fig. 2C). A similar decrease was also observed upon knockdown of *AZI2* (Fig. 2C). Double immunofluorescence staining for total p62 and Ser403-phosphorylated p62 further revealed that the phosphorylated signal within p62 bodies was diminished by knockdown of *NBR1*, *TAX1BP1*, and *AZI2* (Fig. 2D). Taken together, these findings indicate that TBK1 phosphorylates p62 at Ser403 within p62 bodies, primarily through the TBK1–AZI2–TAX1BP1–NBR1 axis.

### PP2A containing PPP2R5 as regulatory subunit is responsible phosphatase for Ser403 of p62

**Figure 3:**
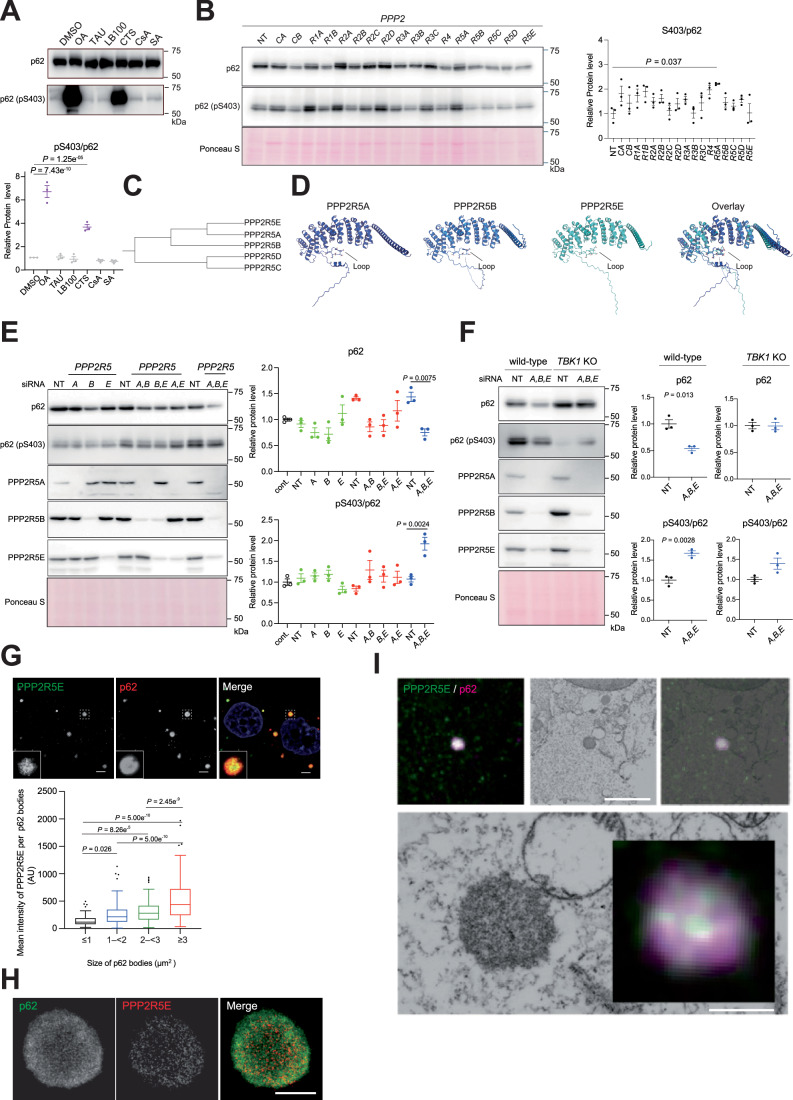
PPP2R5s suppress TBK1-driven p62 phosphorylation at Ser403. (**A**) Immunoblot of Huh-1 cells treated with the indicated phosphatase inhibitors. Okadaic acid (OA), tautomycin (TAU), LB-100, cytostatin (CTS), cyclosporin A (CsA), and sanguinarine (SA). Densitometric ratios of pS403-p62 to total p62 are plotted (*n* = 3). Data are mean ± s.e.m. Statistics: one-way ANOVA followed by Šidák’s multiple-comparison test. (**B**) siRNA screen of PP2A catalytic and regulatory subunits. pS403-p62 signals were normalised to total p62 (*n* = 3). Data are mean ± s.e.m. Statistics: one-way ANOVA followed by Tukey’s multiple-comparison test. (**C**) Phylogenetic tree of the PPP2R5 family. (**D**) AlphaFold-predicted 3D structures of PPP2R5A, PPP2R5B and PPP2R5E. (**E**, **F**) Immunoblot analysis of single, double and triple *PPP2R5* knockdowns in parental and *TBK1*-deficient Huh-1 cells. Quantified band intensities for total p62 and pS403-p62 are shown (*n* = 3). Data are mean ± s.e.m. Statistics: one-way ANOVA followed by Tukey’s multiple-comparison test. (**G**) Immunofluorescence of endogenous PPP2R5E on p62 bodies, stratified by body diameter: ≤ 1 µm (*n* = 73), 1 – <2 µm (*n* = 172), 2 – <3 µm (*n* = 119), and ≥ 3 µm (*n* = 227). Statistics: Welch’s *t* test. Horizontal bars indicate medians; boxes indicate the interquartile range (25th–75th percentiles); whiskers extend to 1.5× the interquartile range. Individual data points outside this range are plotted as outliers. Scale bar, 5 µm. (**H**) STED nanoscopy of PPP2R5E within p62 bodies. Scale bar, 1 µm. (**I**) Correlative light and electron microscopy (CLEM) showing colocalization of endogenous PPP2R5E (green) and p62 (magenta). Scale bars: overview, 5 µm; inset, 1 µm. Source data are available online for this figure.

**Figure EV2:**
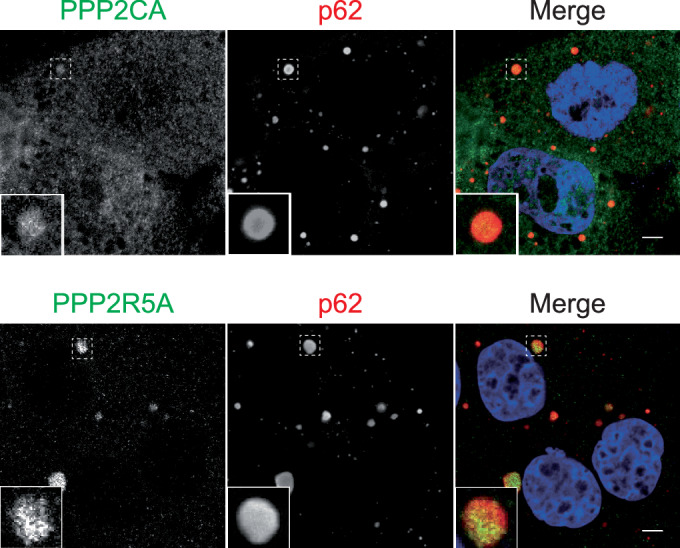
PPP2CA and PPP2R5A colocalize with p62 bodies. Immunofluorescence microscopy of Huh-1 cells stained for PPP2CA and p62 (upper) or PPP2R5A and p62 (bottom). Scale bars, 5 µm. Source data are available online for this figure.

We demonstrated that Ser403 of p62 in p62 bodies is phosphorylated by TBK1. Is this process subject to reversible regulation? To investigate those, we tested the effects of various pharmacological inhibitors of protein phosphatases on the phosphorylation of Ser403 in p62. Among the inhibitors tested—okadaic acid (OA), tautomycin, cytostatin, LB-100, cyclosporin A, and sanguinarine—treatment with OA and cytostatin dramatically increased the levels of Ser403-phosphorylated p62 (Fig. 3A). Since OA is an inhibitor of Protein Phosphatase 2A (PP2A) and PP1 (Bialojan and Takai, 1988), and cytostatin selectively inhibits PP2A (Kawada et al, 1999), we focused our subsequent analyses on PP2A. PP2A is a heterotrimeric enzyme composed of a catalytic subunit (Serine/threonine-protein phosphatase 2A catalytic subunit alpha or beta isoform), a scaffold subunit (Serine/threonine-protein phosphatase 2A 65 kDa regulatory subunit A alpha or beta isoform), and a regulatory subunit, with the 13 known regulatory subunits determining substrate specificity (Serine/threonine-protein phosphatase 2A 56 kDa regulatory subunits) (Fowle et al, 2019; Xu et al, 2006). To identify which regulatory subunits are involved in p62 phosphorylation, we performed a siRNA-based screen. Knockdown of *PPP2R5A*, a member of the *PPP2R5* family, slightly but significantly increased S403-phosphorylated p62 (Fig. 3B). As shown in the phylogenetic tree (Fig. 3C), PPP2R5A, PPP2R5B, and PPP2R5E are closely related, and their overall structural architecture is remarkably conserved (Fig. 3D). This suggests that they may functionally compensate for one another. Therefore, we performed multiple knockdowns targeting members of the *PPP2R5* family. As a result, we found that triple knockdown of *PPP2R5A*, *PPP2R5B*, and *PPP2R5E*—but not single or double knockdowns—drastically reduced total p62 levels, while levels of S403-phosphorylated p62 remained high despite the overall reduction in total p62 (Fig. 3E). This effect was markedly reduced by the loss of TBK1 (Fig. 3F). These results indicate that triple knockdown of *PPP2R5A*, *PPP2R5B*, and *PPP2R5E* increases the proportion of S403-phosphorylated p62 relative to total p62 in a predominantly TBK1-dependent manner, although additional kinase may contribute under these conditions. Next, we investigated the cellular localization of the PPP2R5E. Double immunofluorescence analysis using antibodies against the PPP2R5E and p62 revealed extensive co-localization of endogenous PPP2R5E with p62 bodies (Fig. 3G). We observed the localization of not only the endogenous PP2A catalytic subunit, PPP2CA, but also PPP2R5A within p62 bodies (Fig. EV2). The PPP2R5B antibody failed to detect endogenous PPP2R5B (unpublished observations, S Komatsu-Hirota). STED nanoscopy revealed that the signal pattern of PPP2R5E was detected within p62 bodies, appearing as punctate structures rather than being uniformly distributed throughout the entire p62 body (Fig. 3H). CLEM showed that endogenous PPP2R5E is present in filamentous assembly structures, which are characteristic of typical p62 bodies (Berkamp et al, 2024; Jakobi et al, 2020) (Fig. 3I).

**Figure 4:**
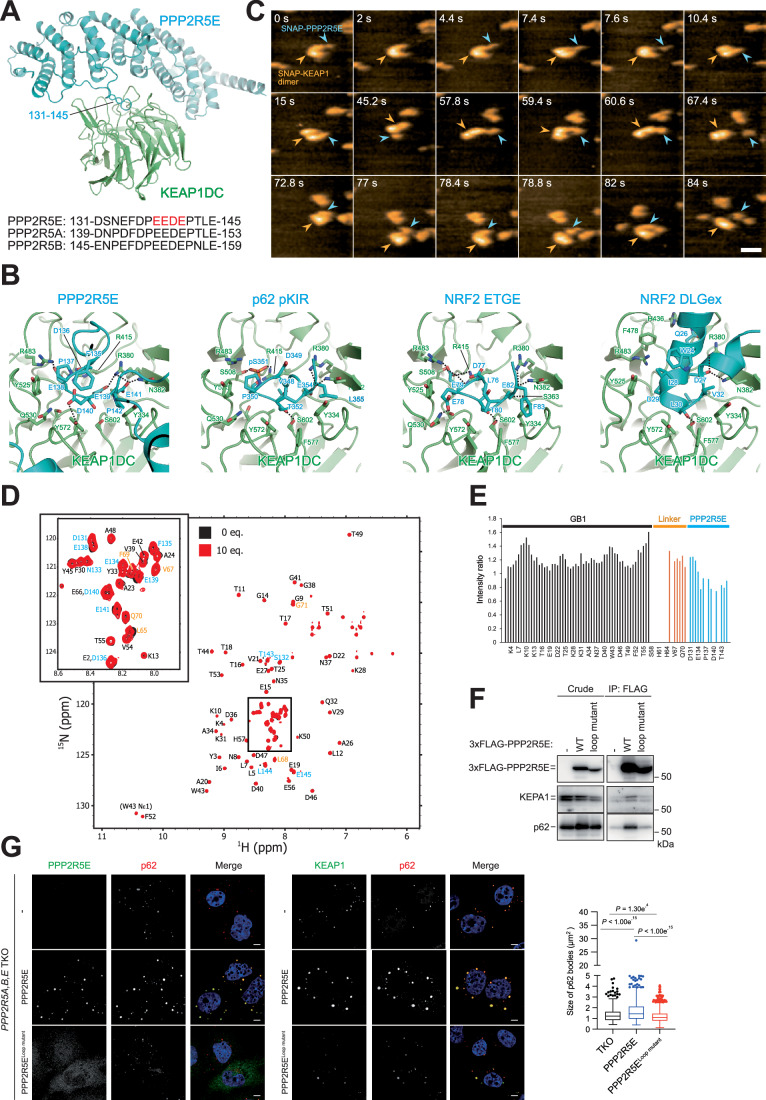
KEAP1 mediates PPP2R5E recruitment to p62 bodies. (**A**) Molecular model of the KEAP1 Kelch domain bound to the acidic loop of PPP2R5E; key interface residues are highlighted. The sequences of the acidic loop regions of PPP2R5A, PPP2R5B, and PPP2R5E are shown. (**B**) Comparative structural model showing the KEAP1-binding orientations of PPP2R5E, p62, and the NRF2 ETGE and DLGex motifs. (**C**) HS-AFM imaging of KEAP1–PPP2R5E interaction. SNAP-KEAP1 and SNAP-PPP2R5E are indicated by orange and cyan arrowheads, respectively. Height scale: 0–4 nm. Scale bar: 20 nm. (**D**, **E**) Two-dimensional ^1^H–^15^N HSQC spectra demonstrating chemical-shift perturbations in PPP2R5E upon titration with KEAP1, confirming direct binding. (**F**) Immunoprecipitation was performed using *PPP2R5A*, *B* and *E* triple-knockout cells in which FLAG-tagged wild-type PPP2R5E or the loop mutant (PPP2R5E^Loop mutant^) was knocked in. The cells were lysed and subjected to immunoprecipitation with anti–FLAG M2 affinity gel, and the resulting immunoprecipitates were analyzed by immunoblotting with the indicated antibodies. (**G**) Immunofluorescence analysis of the cells shown in (**F**). The cells were immunostained with anti-PPP2R5E and anti-p62 antibodies, or with anti-KEAP1 and anti-p62 antibodies. The diameters of p62 bodies were quantified in *PPP2R5A*, *B* and *E* triple-knockout cells (*n* = 475), triple-knockout cells expressing wild-type PPP2R5E (*n* = 621), and triple-knockout cells expressing the loop mutant (*n* = 1770). Statistics: Welch’s *t* test. Exact *P* values are indicated; values below the detection limit are reported as *P* < 1 × 10⁻¹⁵. Horizontal bars indicate medians; boxes indicate the interquartile range (25th–75th percentiles); whiskers extend to 1.5× the interquartile range. Individual data points outside this range are plotted as outliers. Scale bar, 5 µm. Source data are available online for this figure.

How does PP2A containing PPP2R5A, PPP2R5B or PPP2R5E translocate into p62 bodies? AlphaFold3 predicts that a loop containing the acidic cluster (residues 131-145) of the PPP2R5E binds to a basic pocket in the β-propeller domain of KEAP1 (Fig. 4A), which is known to interact with proteins harboring similar acidic clusters, such as NRF2 (Fukutomi et al, 2014; Padmanabhan et al, 2006) and p62 (Komatsu et al, 2010) (Fig. 4B). All PP2A B56 family isoforms—PPP2R5A, B, C, D, and E—contain an acidic cluster (Figs. 3D and  4A) capable of binding KEAP1, a major client protein of the p62 body (Jakobi et al, 2020; Kageyama et al, 2021). Notably, PPP2R5C and PPP2R5D possess a nuclear localization signal and are localized in the nucleus, whereas PPP2R5A, PPP2R5B, and PPP2R5E localize to the cytoplasm (McCright et al, 1996). Based on this, we hypothesized that the PPP2R5A, B, and E are recruited into p62 bodies through their interaction with KEAP1. To investigate this possibility, we first conducted high-speed atomic force microscopic (HS-AFM) analysis using recombinant PPP2R5E and KEAP1 proteins (Appendix Fig. S2). HS-AFM imaging of recombinant PPP2R5E revealed elongated, oval-shaped particles (Movie EV1), consistent with the curved α-helical solenoid architecture formed by eight tandem HEAT-like repeats (Cho and Xu, 2007). Meanwhile, HS-AFM imaging of recombinant KEAP1 visualized a homodimeric structure composed of two globular particles, corresponding to the double glycine repeat and C-terminal (DC) regions, connected by a short stalk (Movie EV2). This architecture was consistent with the dimeric structure previously reported by single-particle EM analysis (Ogura et al, 2010). When recombinant PPP2R5E and KEAP1 were co-imaged by HS-AFM, PPP2R5E molecules were observed to repeatedly associate and dissociate with one of the two globular domains of the KEAP1 homodimer (Fig. 4C; Movie EV3). This observation suggests that PPP2R5E binds asymmetrically to the dimeric KEAP1, most likely through an interaction with the DC domain. Next, we performed NMR titration experiments using the KEAP1 DC domain and PPP2R5E (residues 131–145) fused to GB1 via a flexible linker (Fig. 4D). The KEAP1 DC domain contains the β-propeller structure. In this experiment, chemical shift difference (dppm) were used to quantify chemical-shift changes reflecting alterations in the local environment upon binding, whereas data height ratio (DHR) was used to measure changes in signal intensity, which decreased upon interaction. Both dppm and DHR analyses indicated that PPP2R5E interacts with KEAP1-DC through the loop region (Fig. 4E; Appendix Fig. S3). To verify the interaction in cells, we generated *PPP2R5A*, *PPP2R5B*, and *PPP2R5E* triple-knockout Huh-1 cells (Appendix Fig. S1B) and knocked-in FLAG-tagged wild type PPP2R5E or PPP2R5E loop mutant in which acidic amino acids of the loop region were replaced with alanine (Fig. 4F). Immunoprecipitates from triple-knockout cells expressing wild-type PPP2R5E—but not the loop mutant—contained both KEAP1 and p62 (Fig. 4F). Immunofluorescent analysis indicated that the size of p62 bodies was markedly reduced in the triple-knockout cells (Fig. 4G). Re-expression of wild-type PPP2R5E restored PPP2R5E localization to KEAP1-positive p62 bodies and rescued their size (Fig. 4G), whereas expression of the loop mutant resulted in predominantly diffuse cytoplasmic localization and failed to restore p62 body size (Fig. 4G). Together, these results indicate that PP2A containing PPP2R5A, B, or E interacts with KEAP1 through the conserved acidic loop, thereby facilitating its localization to p62 bodies and regulating their size.

### Phosphorylation-driven alteration in the material properties of p62 bodies

**Figure EV3:**
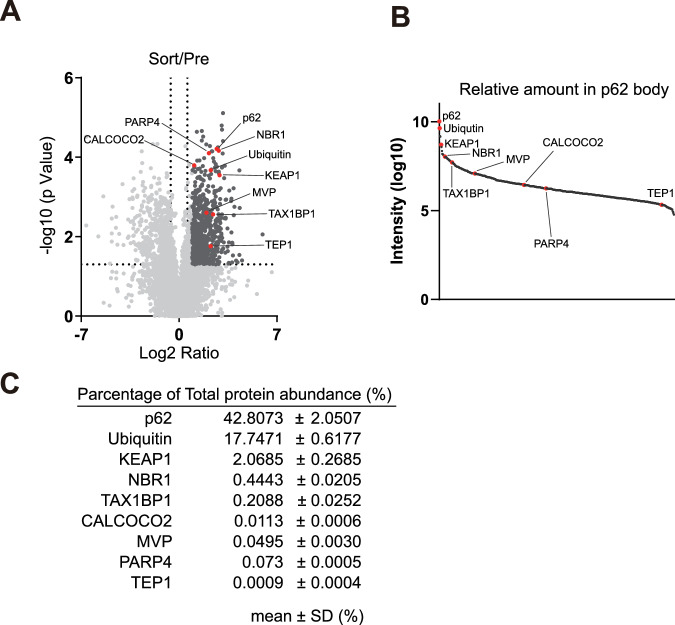
Proteomic analysis of p62 bodies. (**A**) Proteomic profiles of pre- and post-sorted p62 bodies isolated from mEGFP–p62–expressing Huh-1 cells (*n* = 3). Statistics: two-tailed unpaired *t* test. Selective autophagy receptors (NBR1, TAX1BP1, and CALCOCO2) and known client proteins (Ubiquitin, KEAP1, PARP4, and TEP1) are highlighted in red. (**B**) Relative abundance of each protein in p62 bodies was calculated based on label-free quantification (LFQ) intensity. Data are presented as the mean from three independent experiments. (**C**) The abundance ratio of each protein was calculated from the relative abundance data shown in (**B**). The total amount was defined as the sum of abundances of all p62 body–enriched proteins. Source data are available online for this figure.

**Figure 5:**
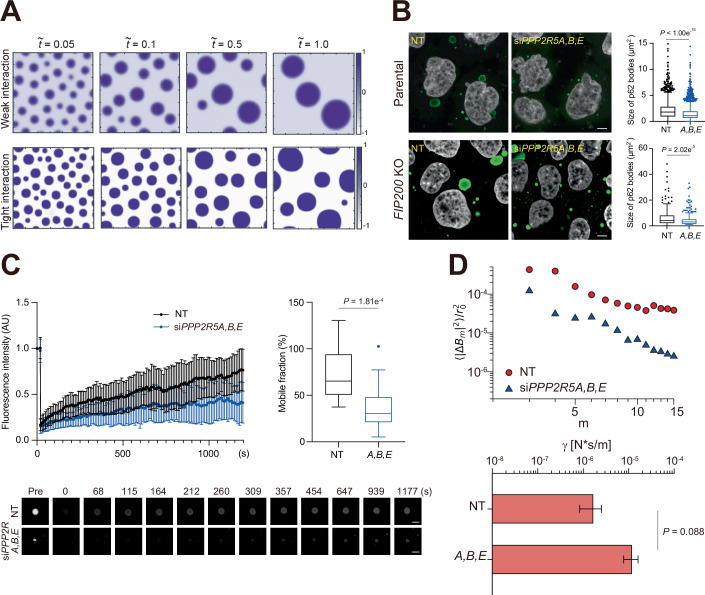
Depletion of PPP2R5s changes material properties of p62 bodies. (**A**) Mathematical model simulation of p62 condensate formation. Cahn–Hilliard–based simulations illustrating how variations in intermolecular interaction strength influence condensate organization in a simplified p62–ubiquitin system. A uniform initial concentration field (ϕ = −0.35) was allowed to evolve within a 5.1-μm square domain under periodic boundary conditions. Under weaker interactions (χ = 2.5), droplets exhibited pronounced dynamic rearrangement. Under stronger interactions (χ = 4.0), condensate reorganization was markedly suppressed, and numerous small droplets persisted. These simulations provide a conceptual illustration of how increased intermolecular interactions can stabilize condensates against dynamic remodeling. (**B**) Immunofluorescence of parental and *FIP200*-knock-out Huh-1 cells after *PPP2R5s* knockdown. p62-body diameters were quantified in wild-type cells treated with control siRNA (*n* = 2247) or si*PPP2R5s* (*n* = 2664) and in *FIP200*-knock-out cells treated with control siRNA (*n* = 515) or si*PPP2R5s* (*n* = 365). Statistics: Welch’s *t* test. Exact *P* values are indicated; values below the detection limit are reported as *P* < 1 × 10⁻¹⁵. Horizontal bars indicate medians; boxes indicate the interquartile range (25th–75th percentiles); whiskers extend to 1.5× the interquartile range. Individual data points outside this range are plotted as outliers. Scale bar, 5 µm. (**C**) FRAP analysis of GFP–p62 mobility in control (*n* = 19) and si*PPP2R5s*-treated cells (*n* = 24). FRAP curve is shown as mean ± s.d. Mean mobile fractions are shown; two-tailed unpaired *t* test. Horizontal bars indicate medians; boxes indicate the interquartile range (25th–75th percentiles); whiskers extend to 1.5× the interquartile range. Individual data points outside this range are plotted as outliers. Scale bar, 1 µm. (**D**) Surface tension analysis of p62 condensates. Fourier spectra of surface fluctuation in Huh-1 cells treated with control (NT) or *PPP2R5s*-knockdown siRNAs. The spectra were obtained by decomposing radial fluctuations into azimuthal modes (*m*) and fitted to a theoretical model of thermally driven surface fluctuations to estimate effective surface tension (*γ*). (bottom) Quantification of γ (N·s/m) obtained from the fitting analysis in control cells (*n* = 4) and si*PPP2R5s*-treated cells (*n* = 4). Statistics: Welch’s *t* test. Data are mean ± s.e.m. Source data are available online for this figure.

We hypothesized that phosphorylation of p62 within p62 bodies, which increases its affinity for ubiquitin chains, could alter the liquidity and size of p62 condensates. Mass spectrometry analysis of purified p62 bodies (Kurusu et al, 2023) revealed that p62 and ubiquitin are the predominant components, accounting for ~60% of total protein abundance, whereas other known client proteins such as KEAP1, NBR1, and TAX1BP1 were detected at much lower levels (Fig. EV3). These results justify our modeling approach in which the condensate behavior is primarily governed by p62 and its multivalent interactions with ubiquitin. Consistent with this idea, we used a mathematical model (see “Methods”) to explore how changes in intermolecular interaction strength influence condensate dynamics in a simplified p62–ubiquitin system. When the interaction parameter χ was small (χ = 2.5), droplets exhibited pronounced dynamic rearrangement over time. In contrast, when χ was large (χ = 4.0), condensate dynamics were markedly suppressed, and numerous small droplets persisted throughout the simulation (Fig. 5A).

**Figure EV4:**
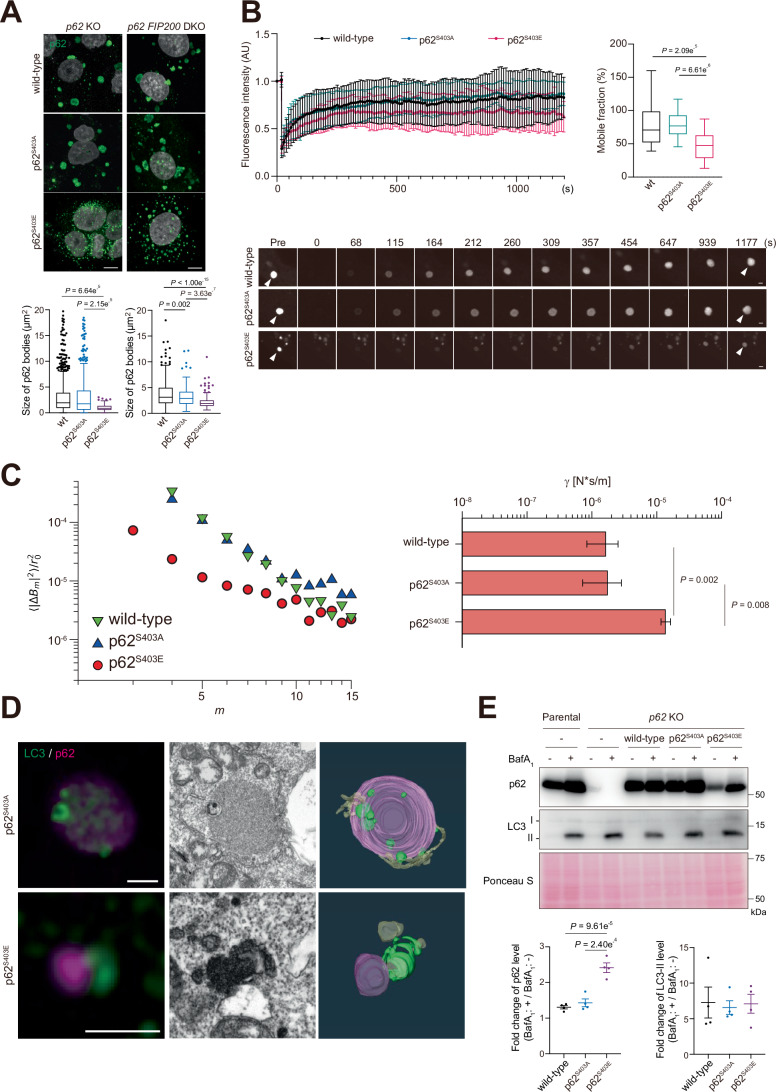
Phospho-mimetic p62 enhances p62 body function. (**A**) Immunofluorescence of *p62*-knock-out (KO) and *p62/FIP200* Huh-1 cells re-expressing wild-type (WT) p62, the phospho-defective mutant p62^S403A^, or the phospho-mimetic mutant p62^S403E^. Diameters of individual p62 bodies were quantified in p62-KO cells expressing WT p62 (*n* = 1154), p62^S403A^ (*n* = 529) or p62^S403E^ (*n* = 81), and in *p62/FIP200* double-KO cells expressing WT p62 (*n* = 349, p62^S403A^ (*n* = 187) or p62^S403E^ (*n* = 410). Statistics: one-way ANOVA with Tukey’s multiple-comparison test. Exact *P* values are indicated; values below the detection limit are reported as *P* < 1 × 10⁻¹⁵. Horizontal bars indicate medians; boxes indicate the interquartile range (25th–75th percentiles); whiskers extend to 1.5× the interquartile range. Individual data points outside this range are plotted as outliers. Scale bar, 10 µm. (**B**) FRAP analysis of GFP-tagged p62 variants. FRAP curve is shown as mean ± s.d. Mobile fractions were determined for cells expressing WT p62 (*n* = 26), p62^S403A^ (*n* = 23) or p62^S403E^ (*n* = 31). One-way ANOVA with Tukey’s test. Scale bar, 1 µm. (**C**) Surface tension analysis of p62 condensates. Fourier spectra of condensates in p62-KO Huh-1 cells expressing WT p62, p62^S403A^ or p62^S403E^. The spectra were obtained by decomposing radial fluctuations into azimuthal modes (*m*) and fitted to a theoretical model of thermally driven surface fluctuations to estimate effective surface tension (*γ*).(Bottom) Quantification of *γ* (N·s/m) obtained from the fitting analysis in p62-KO Huh-1 cells expressing p62 wild-type (*n* = 4), p62^S403A^ (*n* = 3) or p62^S403E^ (*n* = 3). Data are presented as mean ±  s.e.m. Statistics: one-way ANOVA followed by Tukey’s multiple-comparison test. (**D**) Three-dimensional CLEM showing LC3 colocalization with p62 bodies in cells expressing p62^S403A^ or p62^S403E^. LC3 (green), p62 (magenta), and the ER (yellow). Scale bar, 1 µm. (**E**) Immunoblot analysis of autophagic flux in parental and *p62*-null cells reconstituted with WT or mutant p62 in the absence or presence of bafilomycin A_1_ (BafA_1_). Band intensities for p62 and LC3-II were normalised to total protein (*n* = 3). Data are mean ± s.e.m. Statistics: one-way ANOVA with Tukey’s multiple-comparison test. Source data are available online for this figure.

To investigate how p62 phosphorylation affects condensate dynamics in cells, we examined p62 body behavior in *PPP2R5s*-knockdown Huh-1 cells. Immunofluorescence analysis with an antibody against p62 showed that in wild-type Huh-1 cells, p62 bodies ranged in size from 200 nm to 5 μm (average diameter: 2.12 ± 0.04 μm), whereas *PPP2R5s*-knockdown Huh-1 cells exhibited uniformly smaller p62 bodies, with an average size of 1.46 ± 0.03 μm (Fig. 5B). Notably, knockdown of *PPP2R5s* in *FIP200*-knockout Huh-1 cells also resulted in a reduction in p62 body size (Fig. 5B). The average diameter of p62 bodies in *FIP200*-knockout cells treated with control siRNA was 5.73 ± 0.24 μm, whereas in cells treated with *PPP2R5s* siRNA, it was 4.30 ± 0.22 μm. These results indicate that the *PPP2R5s* knockdown–induced reduction in p62 body size occurs independently of autophagy. The fluorescence recovery after photobleaching (FRAP) analysis of GFP-p62 in p62 bodies, which reflects the fluidity of GFP-p62 within these structures, showed that knockdown of *PPP2R5s* delayed recovery compared to control knockdown cells (Fig. 5C). This suggests that the fluidity of p62 bodies was reduced in *PPP2R5s*-knockdown cells relative to control siRNA-treated cells. To further evaluate how phosphorylation influences condensate mechanics, we quantified surface fluctuations of p62 bodies using a previously described analytical framework (Shimobayashi et al, 2025). By decomposing radial fluctuations into Fourier modes and fitting the fluctuation spectra to a theoretical model of thermally driven surface fluctuations (see “Methods”), we obtained the effective surface tension (*γ*) of individual condensates. This analysis revealed that the effective surface tension of p62 bodies in *PPP2R5s*-knockdown Huh-1 cells tended to be higher than that in control siRNA-treated cells (Fig. 5D). Consistently, in *p62*-knockout cells reconstituted with wild-type, S403A, or S403E p62, the effective surface tension of p62^S403E^ condensates was significantly higher than that of wild-type or S403A condensates (refer to Fig. EV4C), further supporting that Ser403 phosphorylation stiffens the condensate interface. Collectively, these results demonstrate that phosphorylation of p62, which enhances its affinity for ubiquitin chains, leads to reduced fluidity, increased surface tension, and smaller droplet size—hallmarks of a liquid-to-gel-like transition of p62 bodies.

### Phosphorylation-driven material transition of p62 bodies enhances autophagic clearance

**Figure 6:**
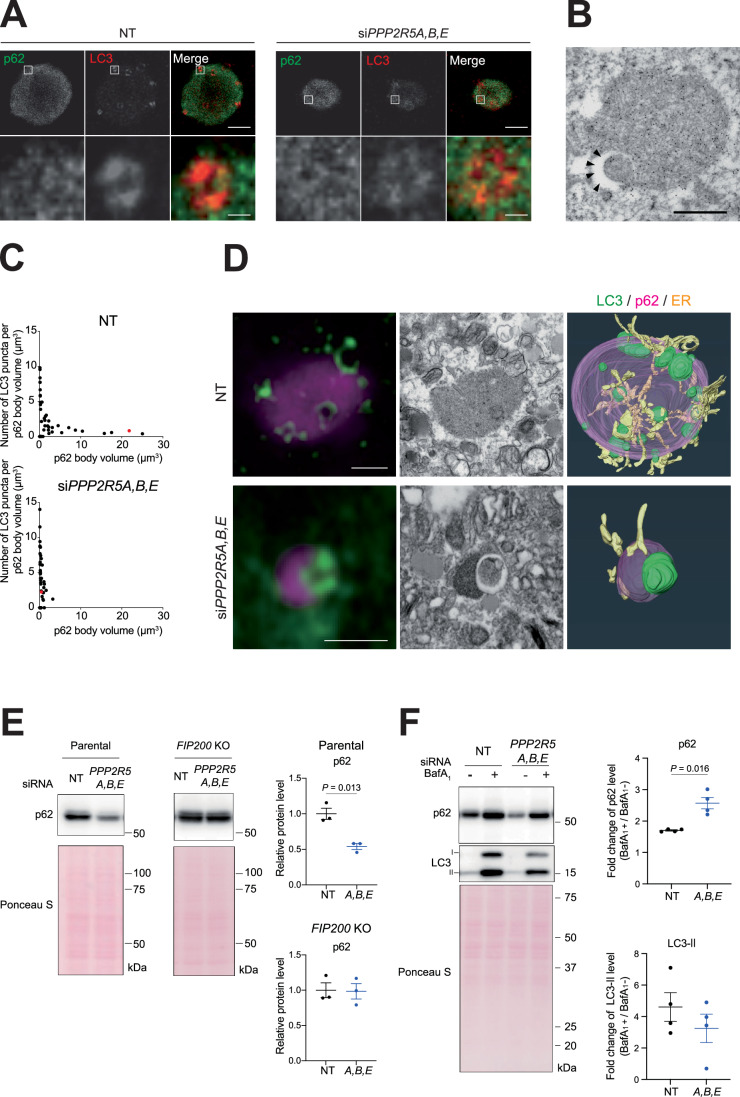
PPP2R5s depletion promotes autophagic degradation of p62 bodies. (**A**) STED imaging of endogenous LC3 within p62 bodies in control siRNA and si*PPP2R5s*-treated cells. Scale bars, 1 µm; enlarged views, 100 nm. (**B**) Immuno-electron microscopy of si*PPP2R5s*-treated cell. The ultrathin section of the cells was incubated with an anti-p62 antibody and the secondary antibody with 12 nm colloidal gold particles. Arrowheads: isolation membrane. Scale bar, 500 nm. (**C**) The number of LC3 puncta normalized to the volume of individual p62 bodies is shown for control siRNA-treated cells (NT) and si*PPP2R5s*-treated cells (*n* = 50 each). Red dots correspond to the p62 bodies analyzed by three-dimensional CLEM in (**D**). (**D**) Three-dimensional CLEM of LC3 (green), p62 (magenta), and the ER (yellow) in control and *PPP2R5s*-knockdown cells. Scale bar, 1 µm. (**E**, **F**) Immunoblot analysis of autophagic flux in parental and *FIP200*-knock-out cells after *PPP2R5s* knockdown ± Bafilomycin A_1_ (BafA_1_). Band intensities for p62 and LC3-II were normalized to total protein (**E**, *n* = 3, **F**, *n* = 4). Data are mean ± s.e.m. Statistics: one-way ANOVA followed by Tukey’s multiple-comparison test. Source data are available online for this figure.

To investigate how PPP2R5s depletion modulates autophagic clearance of p62 bodies, we performed STED nanoscopy with anti-LC3 and anti-p62 antibodies in both control siRNA–treated and *PPP2R5s*-knockdown cells. In both conditions, individual p62 bodies contained multiple LC3-positive substructures (Fig. 6A). Consistent with this, immunoelectron microscopy using an anti-p62 antibody showed that p62 bodies were segmented by isolation membranes (Fig. 6B). Although the spatial relationship between p62 and the isolation membranes remained essentially unchanged, p62 bodies were noticeably smaller in *PPP2R5s*-knockdown cells than in controls (Fig. 6A). These data suggest that *PPP2R5s* depletion enhances the autophagic processing of p62 bodies. Next, we quantified the number of LC3-positive autophagic structures per unit volume of each p62 body in control siRNA– and *PPP2R5s* siRNA–treated cells. As shown in Fig. 6C, the fluorescence-derived volume of individual p62 bodies was first estimated, and the corresponding number of LC3-positive autophagic structures was then calculated. This analysis demonstrated that smaller p62 bodies accommodate proportionally more LC3-positive structures and that the number of LC3-positive structures per p62-body volume in *PPP2R5s*-knockdown cells was higher than that in control siRNA–treated cells (Fig. 6C). In the same scatterplot, representative condensates highlighted with red dots were selected for three-dimensional CLEM (3D-CLEM) using a focused ion beam–scanning electron microscope (FIB-SEM). 3D-CLEM reconstructions confirmed that the fluorescence-based volume measurements faithfully reflected the ultrastructural size of the p62 bodies and visualized multiple isolation-membrane cisternae closely apposed to the p62 bodies (Fig. 6D; Movies EV4 and EV5). These correlative data validate our quantitative light-microscopy analysis and demonstrate that *PPP2R5s* depletion disperses p62 into numerous smaller condensates with an expanded cumulative surface area, which are associated with increased ATG-protein recruitment. Indeed, knockdown of *PPP2R5s* reduced the total level of p62 protein (Figs. 3E and 6E). This reduction was completely rescued in *FIP200*-knockout cells (Fig. 6E), indicating that the decrease in p62 levels depends on FIP200-mediated autophagy. Upon treatment with Bafilomycin A_1_ (BafA_1_), an inhibitor of lysosomal acidification (Yoshimori et al, 1991), p62 accumulated in both control and *PPP2R5s*-knockdown cells, but the increase was significantly greater in *PPP2R5s*-knockdown cells (Fig. 6F). LC3-II levels rose to a comparable extent, indicating that *PPP2R5s* depletion preferentially accelerates p62 turnover relative to overall LC3 flux.

### Dynamics of phospho-mimetic p62 bodies

To validate the role of Ser403 phosphorylation in the TBK1- and PPP2R5s-dependent regulation of p62 body material properties and autophagic turnover, we generated *p62*-knockout Huh-1 cells expressing wild-type p62, phospho-defective p62^S403A^, and phospho-mimetic p62^S403E^. Immunofluorescence analysis using an anti-p62 antibody revealed that, similar to the phenotype observed in *PPP2R5s*-knockdown cells, p62 bodies formed by p62^S403E^ were more uniform in size and markedly smaller than those formed by wild-type p62 or p62^S403A^ (Fig. EV4A). The average diameters of p62 bodies formed by wild-type p62 and p62^S403A^ were 3.18 ± 0.13 μm and 3.44 ± 0.12 μm, respectively, whereas those formed by p62^S403E^ averaged only 1.02 ± 0.07 μm (Fig. EV4A). A similar reduction in p62 body size was also observed in *p62* and *FIP200* double-knockout Huh-1 cells expressing p62^S403E^ (wild-type p62: 3.95 ± 0.18 μm; p62^S403A^: 3.23 ± 0.14 μm; p62^S403E^: 2.12 ± 0.05 μm) (Fig. EV4A), confirming that this size reduction occurs independently of autophagy. To assess how Ser403 phosphorylation affects the internal dynamics of p62 condensates, we performed FRAP analysis in *p62*-knockout Huh-1 cells expressing GFP-tagged p62 variants. Consistent with the *PPP2R5s*-knockdown phenotype, GFP–p62^S403E^ condensates exhibited markedly slower fluorescence recovery than GFP–p62 or GFP–p62^S403A^, indicating reduced molecular mobility within phosphorylated condensates (Fig. EV4B). Moreover, as mentioned above, surface fluctuation analysis showed that the effective surface tension (*γ*) of p62^S403E^ condensates was significantly higher than that of wild-type or p62^S403A^ condensates (Fig. EV4C), demonstrating that Ser403 phosphorylation increases interfacial stiffness. Notably, the p62^S403A^ mutant did not exhibit detectable changes in size, fluidity, or surface tension compared with wild-type p62 (Fig. EV4).

Similar to *PPP2R5s*-knockdown cells, Huh-1 cells expressing the phospho-mimetic p62^S403E^ formed compact p62 bodies, a substantial fraction of which were enclosed by a single LC3-positive autophagosome (Fig. EV4D; Movie EV6). By contrast, cells expressing the phospho-defective p62^S403A^ contained larger p62 bodies that were surrounded by multiple isolation membranes emanating from the endoplasmic reticulum (ER) (Fig. EV4D; Movie EV7). Finally, to evaluate autophagic degradation, we examined the response to BafA_1_. Treatment with BafA_1_ induced a pronounced accumulation of p62 in cells expressing p62^S403E^, whereas only modest accumulation was observed in cells expressing wild-type p62 or p62^S403A^ (Fig. EV4E). LC3-II levels increased to a comparable extent among all three cell lines (Fig. EV4E), suggesting that the S403E substitution selectively accelerates p62 autophagic turnover without affecting overall autophagy flux. Together, these results indicate that mimicking Ser403 phosphorylation recapitulates the *PPP2R5s*-knockdown phenotype—namely, reduced condensate fluidity, increased surface tension, and enhanced autophagic turnover of p62 bodies—supporting a model in which Ser403 phosphorylation, promoted by TBK1 and opposed by PPP2R5s, dynamically regulates the material properties and autophagic fate of p62 condensates.

### Physiological significance of Ser403 phosphorylation in regulating p62 body dynamics and proteostasis

**Figure 7:**
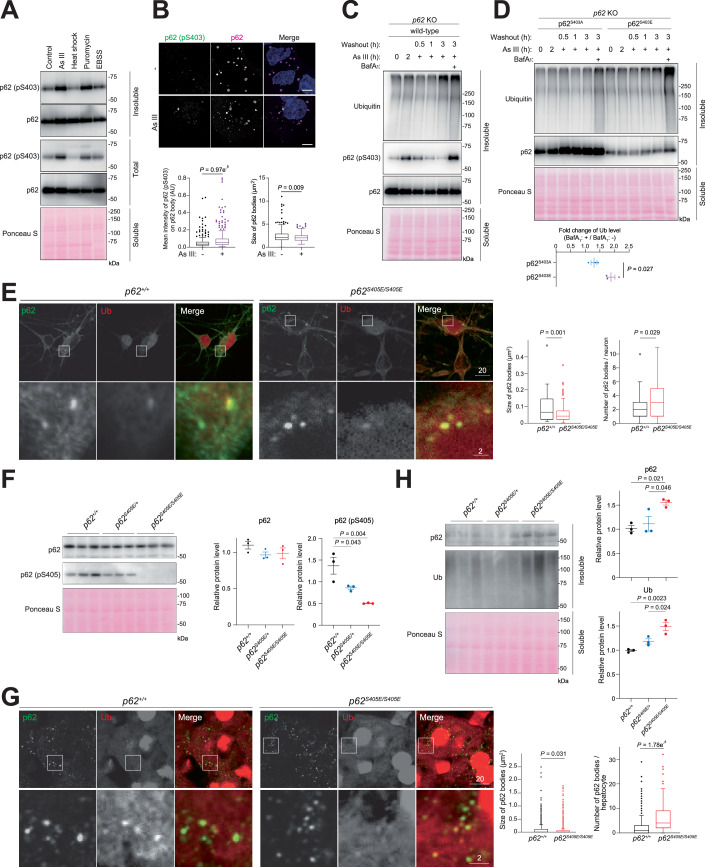
Physiological significance of Ser403-phosphorylated p62 bodies. (**A**) Immunoblot analysis of Huh-1 cells treated with 20 μM arsenite (As III) for 2 h, exposed to heat shock at 42 °C for 2 h, treated with 20 μg/mL puromycin for 2 h, or cultured in EBSS for 2 h. (**B**) Immunofluorescence of Huh-1 cells treated with As III for 2 h. p62-body diameter and Ser403-phosphorylated p62 signal intensity were quantified in control cells (*n* = 302) and As III treated cells (*n* = 304). Two-tailed unpaired *t* test. Scale bar, 10 µm. (**C**) Immunoblot of *p62*-KO Huh-1 cells expressing WT p62 treated with arsenite (As III) for 2 h and then released for the indicated times with or without BafA_1_. (**D**) Immunoblot analysis of *p62*-null cells expressing p62^S403A^ or p62^S403E^ exposed to As III in the presence or absence of BafA_1_. Ubiquitin band intensities in the 3 h wash-out samples were quantified (*n* = 3). Welch’s *t* test. (**E**) Immunostaining for p62 and ubiquitin in neurons from both genotypes. The numbers and diameters of p62 bodies in wild-type neurons (*n* = 50, *n* = 110) and *p62*^*S405E/S405E*^ neurons (*n* = 61, *n* = 209) after 4 weeks of differentiation were quantified. Statistical analysis was performed using the Mann–Whitney *U* test. Scale bars, 20 µm; enlarged views, 2 µm. (**F**) Immunoblot analysis of *p62*^*S405E/S405E*^ mouse livers. Liver homogenates from the indicated genotypes were subjected to SDS–PAGE followed by immunoblotting with anti-p62 and phospho-Ser405–specific antibodies. The band intensities of total and Ser405-phosphorylated p62 were quantified for each genotype (*n* = 3). Data are mean ± s.e.m. Statistical analysis was performed using one-way ANOVA with Tukey’s multiple-comparison test. (**G**) Immunostaining for p62 and ubiquitin in hepatocytes from each genotype. The numbers and diameters of p62 bodies in wild-type (*n* = 152, *n* = 960), and *p62*^*S405E/S405E*^ hepatocytes (*n* = 118, *n* = 1225) from 4-week-old mice were quantified. Statistical analysis was performed using the Mann–Whitney *U* test. Scale bars, 20 µm; enlarged views, 2 µm. (**H**) Immunoblot analysis of the detergent-insoluble fraction from *p62*^*S405E/S405E*^ mouse livers. The detergent-insoluble fractions of liver homogenates from the indicated genotypes (4-weeks-old female mice) were subjected to SDS–PAGE followed by immunoblotting with anti-p62 and anti-ubiquitin antibodies. The band intensities of p62 and ubiquitinated proteins were quantified for each genotype (*n* = 3). Data are mean ± s.e.m. Statistical analysis was performed using one-way ANOVA with Tukey’s multiple-comparison test. Source data are available online for this figure.

To assess the physiological relevance of Ser403 phosphorylation, Huh-1 cells were exposed to four types of proteotoxic stress—arsenite (As III), heat shock, puromycin treatment, and amino acid deprivation. Among these, As III induced the most robust increase in Ser403-phosphorylated p62 within the detergent-insoluble fraction (Fig. 7A). Double-label immunofluorescence revealed a marked elevation of Ser403-phosphorylated p62 within p62 bodies, accompanied by a notable reduction in condensate size (Fig. 7B). To exclude interference from endogenous p62, we used *p62*-knockout Huh-1 cells reconstituted with wild-type p62. Upon As III treatment, these cells also showed a strong increase in Ser403-phosphorylated p62 in the detergent-insoluble fraction (Fig. 7C). Following As III washout, the levels of Ser403-phosphorylated p62 declined gradually; this reduction was completely blocked by BafA_1_ (Fig. 7C). Similarly, BafA_1_ treatment for 3 h during the recovery phase resulted in a substantial accumulation of ubiquitinated proteins in the detergent-insoluble fraction (Fig. 7C). To directly examine the contribution of Ser403 phosphorylation to this response, we expressed either the phospho-mimetic p62^S403E^ or the phospho-deficient p62^S403A^ mutant in *p62*-knockout Huh-1 cells. After an As III pulse–chase followed by BafA_1_ treatment, both cell lines showed an increase in detergent-insoluble ubiquitinated proteins, but the accumulation was significantly greater in cells expressing p62^S403E^ compared to those expressing p62^S403A^ (Fig. 7D). These results support the idea that Ser403 phosphorylation within p62 bodies facilitates the clearance of stress-induced ubiquitinated substrates.

**Figure EV5:**
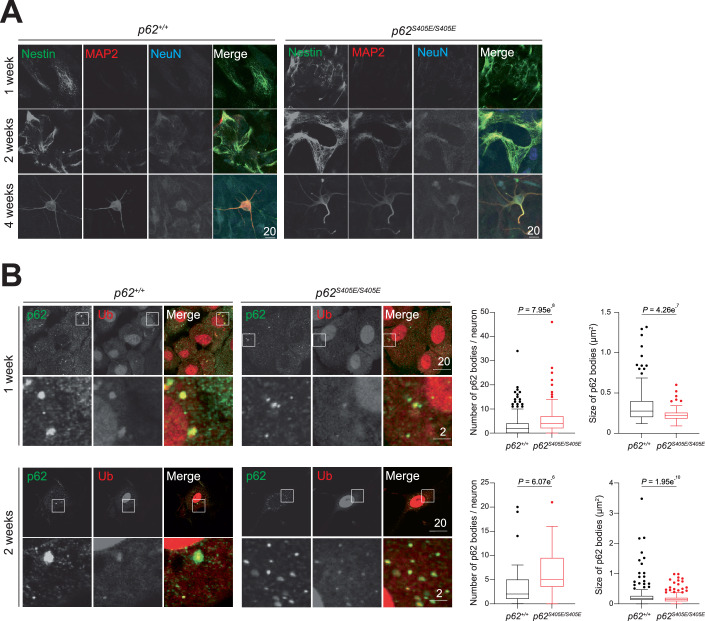
p62 body size and number in wild-type and *p62*^*S405E/S405E*^ ES-derived neurons. (**A**) Immunofluorescence analysis of neurons differentiated from parental and *p62*^*S405E/S405E*^ ES cells after 1, 2, and 4 weeks of culture. Both genotypes successfully differentiated into neurons, as shown by staining for neuronal markers. Scale bars, 20 µm. (**B**) Immunostaining for p62 and ubiquitin in neurons from both genotypes. The numbers and diameters of p62 bodies in wild-type neurons (*n* = 247, *n* = 147) and *p62*^*S405E/S405E*^ neurons (*n* = 141, *n* = 129) at 1 week, and in wild-type neurons (*n* = 75, *n* = 241) and *p62*^*S405E/S405E*^ neurons (*n* = 45, *n* = 298) at 2 weeks were quantified. Statistical analysis was performed using the Mann–Whitney *U* test. Horizontal bars indicate medians; boxes indicate the interquartile range (25th–75th percentiles); whiskers extend to 1.5× the interquartile range. Individual data points outside this range are plotted as outliers. Scale bars, 20 µm; enlarged views, 2 µm. Source data are available online for this figure.

Next, to investigate whether Ser403-dependent size reduction of p62 bodies occurs in quiescent neurons—where autophagy-based proteostasis is critical (Hara et al, 2006; Komatsu et al, 2006)—we generated homozygous *p62*^*S405E/S405E*^ knock-in mouse embryonic stem (ES) cells (murine Ser405 corresponds to human Ser403) and differentiated both parental and mutant ES cells into neurons over a period of 4 weeks (Fig. EV5A). Immunostaining for neural markers confirmed stepwise neuronal differentiation in both genotypes: nestin-positive neural precursors at week 1, nestin- and MAP2-positive immature neurons at week 2, and nestin-, MAP2-, and NeuN-positive post-mitotic neurons at week 4 (Fig. EV5A). Although biochemical experiments are technically challenging in neurons, immunostaining at 1, 2, and 4 weeks revealed p62- and ubiquitin-positive condensates in neuronal soma of both genotypes, indicating that p62 bodies form under basal, non-stressed conditions (Figs. 7E and  EV5B). Quantification across the time course showed that, in S405E knock-in neurons, p62 bodies were significantly smaller yet more numerous per soma than in wild-type neurons at all examined time points (Figs. 7E and  EV5B).

To further examine whether the “smaller-but-more” phenotype occurs in vivo, we generated *p62*^*S405E/S405E*^ knock-in mice. Immunoblotting of liver homogenates revealed comparable total p62 levels among wild-type, heterozygous, and homozygous mice, whereas immunoblotting with a phospho-Ser405-specific antibody confirmed successful knock-in, showing an ~50% reduction in the heterozygote and complete loss of signal in the homozygote (Fig. 7F). Immunofluorescence staining of liver sections from 4-week-old mice showed that, consistent with ES-derived neurons, p62 bodies in hepatocytes of *p62*^*S405E/S405E*^ mice were significantly smaller yet more numerous than those in wild-type and heterozygous mice (Fig. 7G). Quantitative analysis confirmed this shift toward smaller but more abundant p62– and ubiquitin–positive condensates in *p62*^*S405E/S405E*^ livers (Fig. 7G). In addition, immunoblot analysis of the detergent-insoluble fraction demonstrated an increase in p62 and ubiquitinated proteins in *p62*^*S405E/S405E*^ mice compared with wild-type or heterozygous littermates (Fig. 7H). Taken together, these findings demonstrate that Ser405 phosphorylation reshapes p62-condensate organization in differentiated neurons and hepatocytes, producing smaller and more numerous p62 bodies in vivo.

## Discussion

Phosphorylation of p62 at Ser403 has long been regarded primarily as a mechanism to enhance its binding to polyubiquitin chains, thereby facilitating cargo recognition during selective autophagy (Lim et al, 2015; Matsumoto et al, 2011; Pilli et al, 2012). However, our findings reveal that Ser403 phosphorylation exerts broader effects beyond modulating ubiquitin affinity. Specifically, it regulates the biophysical state, morphology, and degradative competence of p62 bodies themselves. This redefines Ser403 phosphorylation not merely as a cargo-binding enhancer but as a multifunctional switch that controls condensate architecture and degradability through autophagic pathways.

Selective autophagic degradation of phase-separated condensates is essential for cellular proteostasis (Kuno et al, 2022; Ohshima et al, 2022; Sun et al, 2018; Turakhiya et al, 2018; Wilfling et al, 2020; Yamasaki et al, 2020; Zaffagnini et al, 2018; Zhang et al, 2018). Among these, p62 bodies are known to function both as dynamic stress-responsive hubs and as substrates for autophagy (Kageyama et al, 2021). While previous studies have shown that their formation is driven by multivalent interactions with ubiquitinated proteins (Lim et al, 2015; Matsumoto et al, 2011; Pilli et al, 2012) and that their degradation involves core autophagy machinery (Ichimura et al, 2008; Pankiv et al, 2007; Turco et al, 2021; Turco et al, 2019), the molecular principles governing the degradability of p62 condensates have remained elusive. Here, we identify phosphorylation of p62 at Ser403 as a key molecular switch that regulates the physical state and degradative competence of p62 bodies. Using an integrative approach combining cell biology, biochemistry, structural modeling, and physiological models, we show that TBK1-mediated phosphorylation of p62 at Ser403 promotes autophagic degradation, while PP2A—particularly holoenzymes containing PPP2R5A, PPP2R5B, or PPP2R5E regulatory subunits—opposes this effect by dephosphorylating p62. These antagonistic modifications dynamically regulate the phosphorylation status of p62 within condensates and thereby fine-tune their physical properties and autophagic engagement.

Our structural and biophysical data provide direct mechanistic insight into how this kinase–phosphatase axis operates within p62 condensates. HS-AFM visualized the PPP2R5E subunit of PP2A as an elongated monomer that binds asymmetrically to one of the two DC domains of the KEAP1 dimer, corroborating the interaction predicted by AlphaFold3 and verified by NMR titration of the KEAP1-DC domain with the PPP2R5E loop. This architecture explains how PPP2R5E and p62 can simultaneously associate with a KEAP1 homodimer, forming a preassembled complex that co-localizes kinase and phosphatase activities within condensates to dynamically regulate Ser403 phosphorylation and degradability.

At the mesoscale, our Cahn–Hilliard–based simulations were designed to conceptually explore how changes in intermolecular interaction strength influence condensate organization. When the interaction parameter χ was low, droplets exhibited pronounced dynamic remodeling. In contrast, higher χ values suppressed condensate reorganization and resulted in the persistence of many small droplets. These modeling outcomes do not aim to reproduce detailed biophysical kinetics, but rather provide an abstract representation of how enhanced intermolecular interactions can stabilize condensates against dynamic remodeling. Complementing the simulation, Fourier-based fluctuation spectroscopy revealed that Ser403 phosphorylation or *PPP2R5* depletion increases the surface tension (γ) of p62 bodies, consistent with a shift toward a more compact, less fluid material state. Together, these results support a model in which Ser403 phosphorylation stabilizes p62 condensates, reduces their internal mobility, and limits size expansion, thereby rendering them competent for autophagic degradation.

Importantly, the similarity between wild-type p62 and the phospho-defective p62^S403A^ mutant, contrasted with the pronounced phenotype of the phospho-mimetic p62^S403E^ mutant, is most consistent with a threshold-dependent transition in condensate material state. Rather than acting as a graded regulator, Ser403 phosphorylation appears to shift condensates across a stability threshold, resulting in qualitative changes in size, mobility, and interfacial stiffness. In this framework, basal levels of phosphorylation are insufficient to induce a material-state transition, whereas sustained or elevated phosphorylation promotes the emergence of compact, gel-like condensates optimized for autophagic engagement.

Phosphorylation at Ser403 simultaneously shrinks p62 condensates and converts them from highly fluid droplets into gels. These two physical changes lower distinct yet complementary barriers to autophagosome formation. Size reduction increases the surface-to-volume ratio and improves geometrical compatibility with autophagosome membranes, while gelation provides a receptor-enriched and mechanically stable interface for isolation membrane nucleation and expansion. Our simulation and fluctuation analyses quantitatively support this model, showing that phosphorylation-driven strengthening of p62–ubiquitin interactions increases interfacial stiffness and limits condensate growth, thereby producing condensates optimized for efficient autophagic capture. Functionally, the phospho-mimetic p62^S403E^ mutant recapitulated all aspects of the *PPP2R5s*-knockdown phenotype—smaller condensate size, slower FRAP recovery, higher surface tension, and enhanced autophagic turnover—whereas the phospho-defective p62^S403A^ mutant formed larger, more fluid condensates and showed no increase in surface tension or autophagic turnover compared with p62^S403E^. Thus, the opposing activities of TBK1 and PPP2R5s define a reversible post-translational switch that governs the physical state and fate of p62 condensates.

Finally, our analysis of ES-cell-derived neurons demonstrates the physiological relevance of this mechanism. Quantitative imaging revealed that Ser405 (the murine equivalent of human Ser403) phosphorylation induces a “smaller-but-more” phenotype: p62 bodies in S405E knock-in neurons were not only smaller but also more numerous than in wild-type cells throughout neuronal differentiation. This behavior suggests that phosphorylation enhances nucleation while limiting further condensate expansion, maintaining a steady state of numerous small condensates even under basal conditions. Such dynamic tuning of p62 body number and size likely supports sustained proteostasis in post-mitotic neurons, where autophagy plays a critical housekeeping role (Hara et al, 2006; Komatsu et al, 2006). Beyond neuronal systems, our in vivo analysis in *p62*^*S405E/S405E*^ knock-in mice further substantiates the physiological relevance of Ser405 phosphorylation. In hepatocytes, p62 bodies exhibited the same “smaller-but-more” phenotype observed in neurons, accompanied by increased levels of detergent-insoluble p62 and ubiquitinated proteins. Rather than contradicting the cell-based finding that phosphorylation promotes autophagic clearance, these features are consistent with a high-flux steady state in vivo, where Ser405 phosphorylation lowers the nucleation barrier and stabilizes gel-like condensates, thereby enhancing their formation and capture efficiency while autophagic turnover is concurrently accelerated. When condensate formation slightly outpaces degradative capacity or lysosomal throughput, the apparent increase in the insoluble fraction may arise despite elevated flux. Thus, the S405E substitution shifts the equilibrium toward numerous small, autophagy-competent condensates with rapid turnover, rather than toward defective clearance. This finding suggests that phosphorylation-driven remodeling of p62 condensates operates across distinct cellular contexts—from post-mitotic, metabolically active neurons to proliferative tissues such as the liver, where autophagic degradation of p62 bodies is essential for maintaining proteostasis and preventing liver injury or tumorigenesis (Komatsu et al, 2007; Mathew et al, 2009; Takamura et al, 2011). In both systems, autophagy is continuously engaged to maintain homeostasis, and Ser403/405 phosphorylation provides a mechanism to sustain ubiquitin-dependent quality control under fluctuating proteostatic demands.

In light of these findings, we propose that p62 bodies serve dual roles: acting as signaling platforms for KEAP1 sequestration and NRF2 activation, and as condensate-based cargos for autophagic degradation of ubiquitinated proteins. The balance between these two roles is determined by the relative activities of TBK1 and PP2A within p62 condensates. When TBK1 predominates, Ser403 phosphorylation promotes condensate remodeling and efficient degradation; when PP2A activity prevails, dephosphorylated, more fluid condensates may retain KEAP1 binding and maintain NRF2 signaling. Thus, Ser403 phosphorylation functions as a molecular toggle that switches p62 bodies between signaling-active and degradation-ready states depending on the cellular context.

While our integrative framework connects structural, biophysical, and physiological scales, several limitations should be considered. The mathematical model simplifies condensate composition to a single order parameter and uniform interaction strength and therefore does not explicitly incorporate the potential contributions of minor client proteins such as KEAP1, NBR1, or TAX1BP1. In addition, the surface-fluctuation analysis assumes equilibrium thermal noise, whereas active mechanical forces in living cells may further modulate condensate behavior. Finally, although Ser403/405 phosphorylation is shown here to be physiologically relevant in neurons and liver tissue, its dynamic regulation under aging, metabolic stress, or disease conditions remains to be defined. Addressing these questions will refine our understanding of phosphorylation-driven condensate remodeling in vivo. Taken together, Ser403 phosphorylation functions as a threshold-dependent molecular switch that couples kinase–phosphatase signaling to condensate material-state transitions and selective autophagy, thereby ensuring efficient proteostasis across diverse cellular contexts.

## Methods

**Table:** Reagents and tools table

Reagent/resource	Reference or source	Identifier or catalog number
**Experimental models**		
Mouse: *p62*^*+/+*^	This study	N/A
Mouse: *p62*^*S405A/S405A*^	This study	N/A
Mouse: *p62*^*S405E/+*^	This study	N/A
Mouse: *p62*^*S405E/ S405E*^	This study	N/A
**Cells**		
Huh-1	RIKEN	Cat# JCRB0199; RRID: CVCL_2956
Huh-1 *p62* KO	Ichimura et al, 2013	N/A
Huh-1 *FIP200* KO	Kurusu et al, 2023	N/A
Huh-1 *TBK1* KO	This study	N/A
Huh-1 *PPP2R5A/B/E* TKO	This study	N/A
HEK293T	ATCC	Cat# CRL-3216
*p62*^*S405A/S405A*^ knock-in mES cells	This study	N/A
*p62*^*S403E/S403E*^ knock-in mES cells	This study	N/A
RENKA4 (C57BL/6N-derived mES cells)	This study	N/A
C57BL/6N-derived mES (RENKA)	Mishina and Sakimura, 2007	N/A
Expi293F cells	Thermo Fisher Scientific	Cat# A14527
Sf9 cells	Thermo Fisher Scientific	Cat# B82501
**Recombinant DNA**		
pEGFP-C1	Clontech	Cat# 6084-1
pCMV-PE2-P2A-GFP	Addgene	Cat# 132776; RRID: Addgene_132776
hU6-sgRNA plasmid	Yuza et al, 2018	N/A
pX330-U6-Chimeric_BB-CBh-hSpCas9	Addgene	Cat# 42230; RRID: Addgene_42230
pFastBac Dual expression vector	Thermo Fisher Scientific	Cat# 10712024
pOSF (One-STrEP-FLAG) vector	This study	N/A
pGEX6P-1 vector	Cytiva	Cat# 28954648
pGEX6P-1-8xUb	Genscript	N/A
pGEX6P-mCherry-p62	Ikeda et al, 2023	N/A
pGEX6P-1-SNAP-KEAP1-TwinStrep	This study	N/A
pGEX6P1-SNAP-PPP2R5E	This study	N/A
pFastBacDual-His-SNAP-NBR1	This study	N/A
pFastBacDual-His-SNAP-NBR1 (D50R)	This study	N/A
pOSF-SNAP-TBK1	This study	N/A
pOSF-SNAP-TAX1BP1	This study	N/A
pOSF-SNAP-AZI2	This study	N/A
pOSF-SNAP-TBK1BP1	This study	N/A
pGBHPS vector	Kobashigawa et al, 2009	N/A
pMRX_ires-puro	Kurusu et al, 2023	N/A
pMRX_ires-puro_p62	This study	N/A
pMRX_ires-puro_p62^S403A^	This study	N/A
pMRX_ires-puro_p62^S403E^	This study	N/A
pMRX_ires-puro_mGFP-p62	This study	N/A
pMRX_ires-puro_ mGFP-p62^S403A^	This study	N/A
pMRX_ires-puro_ mGFP-p62^S403E^	This study	N/A
pMRX_ires-puro_TBK1	This study	N/A
pMRX_ires-puro_TBK1^K38A^	This study	N/A
pZDonor-AAVS1-EF1-FLAG-PPP2R5E	This study	N/A
pZDonor-AAVS1-EF1-FLAG-PPP2R5E^Loop8A^	This study	N/A
**Antibodies**		
Guinea pig polyclonal anti-p62/SQSTM1 (C-terminus)	Progen Biotechnik	Cat# GP62-C; RRID: AB_2687531
Mouse monoclonal anti-p62 Ick ligand	BD Biosciences	Cat# 610833; RRID: AB_398152
Rabbit polyclonal anti-p62	MEDICAL & BIOLOGICAL LABORATORIES CO., LTD.	Cat# PM045; RRID: AB_1279301
Rabbit polyclonal anti-p62 (pSer403)	Gene Tex	Cat# GTX128171; RRID: AB_2885723
Rabbit monoclonal anti-pTBK1 (pS172)	Cell Signaling Technology	Cat# 5483; RRID: AB_3695654
Rabbit monoclonal anti-TBK1	Cell Signaling Technology	Cat# 38066; RRID: AB_2827657
Mouse polyclonal anti-PPP2R5A	Abcam	Cat# ab899621; RRID: AB_2042675
Rat monoclonal anti-PPP2R5B	Santa Cruz Biotechnology	Cat# sc-56954; RRID: AB_785345
Rabbit monoclonal anti-PPP2R5E	Abcam	Cat# ab198500; RRID: AB_3676292
Mouse polyclonal anti-PPP2CA	Cell Signaling Technology	Cat# 2038; RRID: AB_2169495
Rabbit polyclonal anti-LC3	MEDICAL & BIOLOGICAL LABORATORIES CO., LTD.	Cat# PM036; RRID: AB_2274121
Mouse monoclonal anti-Ubiquitin (P4D1)	Santa Cruz Biotechnology	Cat# sc-8017; RRID: AB_2762364
Anti-Pan Ubiquitin monobody	Cosmobio	Cat# MB-001-B
Rat monoclonal anti-Nestin	MERCK	Cat# MAB353; RRID: AB_2151130
Goat polyclonal anti-MAP2	Frontier Institute	Cat# MAP2-Go; RRID: AB_2571793
Chicken polyclonal anti-NeuN	MERCK	Cat# ABN91; RRID: AB_11205760
Mouse monoclonal anti-NBR1	Santa Cruz Biotechnology	Cat# 130380; RRID: AB_2149402
Rabbit monoclonal anti-TAX1BP1	Cell Signaling Technology	Cat# 5105; RRID: AB_11178939
Rabbit polyclonal anti-AZI2	ProteinTech	Cat# 15042-1; RRID: AB_3672616
Rabbit monoclonal anti-TBK1BP1	Cell Signaling Technology	Cat# 8605; RRID: AB_10839270
Rabbit polyclonal anti-KEAP1	Proteintech	Cat# 10503-2-AP; RRID: AB_2132625
Mouse monoclonal anti-DDDDK-tag	MEDICAL & BIOLOGICAL LABORATORIES CO., LTD.	Cat# M185-3 L; RRID: AB_11123930
Alexa Fluor 488-conjugated anti-rabbit IgG	Thermo Fisher Scientific	Cat# A21236; RRID: AB_2535805
Alexa Fluor Plus 647-conjugated anti-mouse IgG	Thermo Fisher Scientific	Cat# A21236; RRID: AB_2535805
Cy3-conjugated Streptavidin	Jackson ImmunoResearch Laboratories	Cat# 016-160-084; RRID: AB_2337244
HRP-conjugated goat polyclonal anti-rabbit IgG	Jackson ImmunoResearch Laboratories	Cat# 111-035-144; RRID: AB_2307391
HRP-conjugated goat Anti-Mouse IgG	Jackson ImmunoResearch Laboratories	Cat# 115-035-166; RRID: AB_2338511
HRP-conjugated goat polyclonal anti-guinea pig IgG	Jackson ImmunoResearch Laboratories	Cat# 106-035-003; RRID: AB_2337402
Anti-FLAG M2 affinity agarose gel	Merck	Cat# A2220; RRID: AB_10063035
Anti-mouse STAR RED	Abberior	Cat# STRED-1001-500UG; RRID: AB_3068620
Anti-Rabbit STAR ORANGE	Abberior	Cat# STRED-1002-500UG; RRID: AB_3068622
**Bacterial and viral strains**		
*E. coli* strain BL21(DE3)	Funakoshi	Cat# DS250
DH10Bac competent cells	Thermo Fisher Scientific	Cat# 10361012
**Oligonucleotides and other sequence-based reagents**		
pegRNA spacer for p62^S403E^	This study	5’-GACUGGAGUUCACCUGUAGA-3’
pegRNA RT template for p62^S403E^	This study	5’-GUGGACCCAGAG-3’
pegRNA PBS for p62^S403E^	This study	5’-ACAGGUGAACUCC-3’
siRNA targeting NBR1	Dharmacon	Cat# M-010522-01
siRNA targeting TAX1BP1	Dharmacon	Cat# M-016892-01
siRNA targeting AZI2	Dharmacon	Cat# M-014092-00
siRNA targeting TBK1BP1	Dharmacon	Cat# M-020406-01
siRNA targeting KEAP1	Dharmacon	Cat# M-012453-00
siRNA targeting PPP2CA	Dharmacon	Cat# L-003598-01
siRNA targeting PPP2CB	Dharmacon	Cat# L-003599-00
siRNA targeting PPP2R1A	Dharmacon	Cat# L-010259-00
siRNA targeting PPP2R1B	Dharmacon	Cat# L-017592-00
siRNA targeting PPP2R2A	Dharmacon	Cat# L-004824-00
siRNA targeting PPP2R2B	Dharmacon	Cat# L-003022-00
siRNA targeting PPP2R2C	Dharmacon	Cat# L-019167-00
siRNA targeting PPP2R2D	Dharmacon	Cat# L-032298-00
siRNA targeting PPP2R3A	Dharmacon	Cat# L-017376-00
siRNA targeting PPP2R3B	Dharmacon	Cat# L-019459-00
siRNA targeting PPP2R3C	Dharmacon	Cat# L-018203-01
siRNA targeting PPP2R4	Dharmacon	Cat# L-005214-00
siRNA targeting PPP2R5A	Dharmacon	Cat# L-009352-00
siRNA targeting PPP2R5B	Dharmacon	Cat# L-009366-00
siRNA targeting PPP2R5C	Dharmacon	Cat# L-009433-00
siRNA targeting PPP2R5D	Dharmacon	Cat# L-009799-00
siRNA targeting PPP2R5E	Dharmacon	Cat# L-008531-00
siRNA non-targeting Pool #2	Dharmacon	Cat# D-001206-14
siRNA ON-TARGETplus Control	Dharmacon	Cat# D-001810-10
sgRNA targeting TBK1	This study	5’-GATGAAGATCAACCTGGAAG-3’
sgRNA targeting PPP2R5E	This study	5’-GCGCTCCACTCTTAATGAAC
sgRNA targeting PPP2R5E	This study	5’-GACTACATTACAATAAGCAG-3’
sgRNA targeting PPP2R5A	This study	5’-CTTGGCCTCACATACAGGTA-3’
sgRNA targeting PPP2R5B	This study	5’-GCCATAGACCCGGTGCAGGA-3’
**Chemicals, enzymes and other reagents**		
Lipofectamine 3000 Transfection Reagent	Thermo Fisher Scientific	Cat# L3000015
Lipofectamine 2000 Transfection Reagent	Thermo Fisher Scientific	Cat# 1668030
Lipofectamine RNAiMAX Transfection Reagent	Thermo Fisher Scientific	Cat# 13778150
Lipofectamine LTX Reagent with PLUS Reagent	Thermo Fisher Scientific	Cat# 15338100
TransIT-Insect Transfection Reagent	Takara Bio	Cat# V6204
X-tremeGENE HP	Roche	Cat# 6366244001
ExpiFectamine 293 Transfection Kit	Thermo Fisher Scientific	Cat# A14525
bafilomycin A_1_	KOM Biomedical	Cat# BVT-0252-M001
TrypLE Select	Gibco, Thermo Fisher Scientific	Cat# 12563011
B27 Supplement	Gibco, Thermo Fisher Scientific	Cat# 17504044
leukemia inhibitory factor	Nakalai Tesque	Cat# 039-21461
fibroblast growth factor 2	PeproTech	Cat# 100-18B
cOmplete^TM^ EDTA-free protease inhibitor cocktail	Roche	Cat# 5056489001
Protease Inhibitor Cocktail(EDTA free)(100x)	Nakalai Tesque	Cat# 03969-34
gelatin	Sigma-Aldrich	Cat# G9391
Hoechst 33342	Thermo Fisher Scientific	Cat# 62249
gridded glass coverslips	Matsunami Glass	Cat# GC1310
saponin	Fujiwako	Cat# 199-18653
Epok812	Oken Shoji	Cat# 02-1003
MitoTracker Red CMXROS	Invitrogen	Cat# M7512
glass-bottom slides	Greiner Bio-One	Cat# 543078
bovine serum albumin	Nacalai Tesque	Cat# 08587-26
Sf-900 III SFM	Thermo Fisher Scientific	Cat# 12658027
GST-Accept beads	Nacalai Tesque	Cat# 09277-14
VIVASPIN 500 MWCO	Sartorius	Cat# VS0131
SNAP-Surface 488	New England Biolabs	Cat# S9124S
SNAP-Surface 649	New England Biolabs	Cat# S9159S
PD SpinTrap G-25 columns	Cytiva	Cat# 28918004
Expi293 Expression Medium	Thermo Fisher Scientific	Cat# A1435101
Strep-tactin 4Flow high capacity resin	IBA Lifesciences	Cat# 2-12500-010
desthiobiotin	Sigma-Aldrich	Cat# D1411-500MG
NEBuilder HiFi DNA Assembly Kit	New England Biolabs	Cat# 5520S
Ammonium-^15^N chloride	Sigma-Aldrich	Cat# 299251
D-Glucose-^13^C_6_	Sigma-Aldrich	Cat# 389374
Ni-NTA agarose	QIAGEN	Cat# 30210
HiTrap Desalting column	Cytiva	Cat# 29048684
Micro BCA Protein Assay	Thermo Fisher Scientific	Cat# 23235
Trypsin/LysC mix	Thermo Fisher Scientific	Cat# A40007
S-Trap columns	ProtiFi	Cat# C002-MICRO-0010PK
Quantitative Peptide Assay Kit	Thermo Fisher Scientific	Cat# 23275
4%-Paraformaldehyde Phosphate Buffer Solution	Nacalai Tesque	Cat# 09154-85
Dulbecco’s modified Eagle’s medium (DMEM), high glucose	Nakalai Tesque	Cat# 08459-64
Fetal bovine serum (FBS)	Thermo Fisher Scientific	Cat# A5256701
Penicillin–Streptomycin–Glutamine Mixed Solution	Nacalai Tesque	Cat# 06168-34
MEM Non-Essential Amino Acids Solution (x100)	Nacalai Tesque	Cat# 06344-56
100mM-Sodium Pyruvate Solution (x100)	Nacalai Tesque	Cat# 06977-34
Trypsin/EDTA	Nacalai Tesque	Cat# 32777-44
Opti-MEM reduced serum Medium	Thermo Fisher Scientific	Cat# 31985070
polybrene	Sigma-Aldrich	Cat# H9268
SlowFade Diamond antifade mount with DAPI	Thermo Fisher Scientific	Cat# S36964
Clarity Western ECL Substrate	Bio-Rad	Cat# 1705061
TritonX-100	Nacalai Tesque	Cat# 35501-15
As III	Sigma-Aldrich	Cat# S7400
Puromycin	FUJIFILM Wako Pure Chemical Corporation	Cat# 166-23153
Earle’s Balanced Salts (EBSS)	Sigma-Aldrich	Cat# E2888
**Software**		
FV31S-SW software	Olympus	version 2.6.1.243
CellPathfinder software	Yokogawa Electric Corp	N/A
Abberior MATRIX detector	Abberior Instruments	N/A
Lightbox software	Abberior Instruments	N/A
ImageJ software	Schindelin et al, 2012	RRID:SCR_003070
Photoshop 2021v25.0	Adobe	RRID:SCR_014199
Fiji software	Schindelin et al, 2012	RRID:SCR_002285
Proteome Discoverer™ 3.2	Thermo Fisher Scientific	RRID:SCR_014477
GraphPad Prism 9	GraphPad Software	RRID:SCR_002798
TopSpin	Bruker	version 4.1.4
Sparky	Goddard and Kneller	version 3.113
Kodec	Research Institute of Biomolecule Metrology Co.	N/A
UMEX RIBM HS-AFM	Research Institute of Biomolecule Metrology Co.	N/A
**Other**		

### Mathematical model simulation of p62 condensate formation

We formulated a mathematical model of p62 condensate formation based on previous studies (Yang et al, 2024).

The free energy, *F*, of the system can be written as:

1 $$\frac{F}{{k}_{{{{\rm{B}}}}}T}=\frac{1}{{a}^{3}}\int \left[\frac{b}{2}{\left(\nabla \left({\phi }_{{{{\rm{p}}}}62}-{\phi }_{{{{\rm{sol}}}}}\right)\right)}^{2}+\chi {\phi }_{{{{\rm{p}}}}62}{\phi }_{{{{\rm{sol}}}}}+{\phi }_{{{{\rm{p}}}}62}{{\mathrm{ln}}}\;{\phi }_{{{{\rm{p}}}}62}+{\phi }_{{{{\rm{sol}}}}}{{\mathrm{ln}}}\;{\phi }_{{{{\rm{sol}}}}}\right]{dV}$$

where *a* is the lattice size that is introduced when deriving the mixing free energy based on a lattice model, and ${\phi }_{{{{\rm{p}}}}62}$ and ${\phi }_{{{{\rm{sol}}}}}(=1-{\phi }_{{{{\rm{p}}}}62})$ are the p62 and solvent area-fraction (hereafter referred to as concentration), respectively. The symbol *b* represented a parameter related to the surface tension of the condensates. *χ* represented the strength of p62 self-interaction, including Ub-mediated p62 interactions, with $\chi =2.5$ for weak interactions and $\chi =4$ for tight interactions.

For the numerical simulation, the space was discretized by a lattice with 40 nm on a side, and a concentration field was assigned to each location. Because of ${\phi }_{{{{\rm{p}}}}62}+{\phi }_{{{{\rm{sol}}}}}=1$, we introduce a variable $\phi \equiv {\phi }_{{{{\rm{p}}}}62}-{\phi }_{{{{\rm{sol}}}}}$, and then ${\phi }_{{{{\rm{p}}}}62}=(1+\phi )/2$, and ${\phi }_{{{{\rm{sol}}}}}=(1-\phi )/2$.

The time evolution of the system was assumed to follow the Cahn-Hilliard equation with a random flux term coming from thermal noise:

2 $$\frac{\partial \phi }{\partial t}=\frac{\partial }{\partial \vec{x}}\cdot \left[L\left(\phi \right)\frac{\partial }{\partial \vec{x}}\left(\frac{\delta F}{\delta \phi }\right)\right]-\frac{\partial }{\partial \vec{x}}\cdot {J}^{({{{\rm{R}}}})}(\vec{x},t)$$

where ${J}^{({{{\rm{R}}}})}(\vec{x},t)$ stands for the random current coming from thermal noise, and satisfies the following fluctuation–dissipation theorem:

3 $$\left\langle {J}_{\alpha }^{\left({{{\rm{R}}}}\right)}\left(\vec{x},t\right){J}_{\beta }^{\left({{{\rm{R}}}}\right)}\left({\vec{x}}^{{\prime} },{t}^{{\prime} }\right)\right\rangle =2{k}_{B}{TL}\left(\phi \right)\delta \left(\vec{x}-{\vec{x}}^{{\prime} }\right)\delta (t-{t}^{{\prime} })$$

$L\left(\phi \right)$ is the transport coefficient and is given as

4 $$L={L}_{0}+{L}_{1}\frac{1}{4}(1+\phi )(1-\phi )$$

In this model, the transport coefficient $L\left(\phi \right)$ explicitly depends on concentration. When the intermolecular interaction parameter *χ* is large, the concentration difference between the p62-rich and solvent-rich phases increases, thereby suppressing molecular transport across the interface. Consequently, droplets remain smaller in size and the effective viscosity of the condensates increases. This is consistent with experimental observations such as slower fluorescence recovery in FRAP assays and the suppression of surface fluctuations.

Using *ϕ*, the free energy Eq. (1) is written as a functional of *ϕ* as

5 $$\frac{F}{{k}_{{{{\rm{B}}}}}T}=\frac{1}{{a}^{3}}\int \left[\frac{b}{2}{\left(\nabla \phi \right)}^{2}+\frac{\chi }{4}(1-{\phi }^{2})+\frac{1+\phi }{2}{{\mathrm{ln}}}\frac{1+\phi }{2}+\frac{1-\phi }{2}{{\mathrm{ln}}}\frac{1-\phi }{2}\right]{dV}$$

The chemical potential $\mu =\delta F/\delta \phi$ is derived from Eq. (3) as

6 $$\frac{\delta F}{\delta \phi }=\frac{{k}_{B}T}{{a}^{3}}\left[-b\varDelta \phi -\frac{1}{2}\chi \phi +\frac{1}{2}{{\mathrm{ln}}}\frac{1+\phi }{1-\phi }\right]$$

where Δ is the Laplacian defined as $\Delta \equiv \nabla \cdot \nabla$. The CH equation is standardly used in phase separation dynamics (Berry et al, 2018). A square region of 5.1 μm length with periodic boundary conditions was considered as a system, and it was assumed that adapter proteins were uniformly distributed in the cytoplasm at a concentration of $\phi =-0.35$ in the initial state. Other parameters were set as $\widetilde{b}=2/3,\widetilde{{L}_{0}}=0$, *and* $\widetilde{{L}_{1}}=2.0.$

### Cell

Wild-type (JCRB0199, RIKEN), *p62*-deficient (Ichimura et al, 2013), *FIP200*-deficient (Kurusu et al, 2023), and *PPP2R5A/B/E*-deficient Huh-1 cells were cultured in Dulbecco’s Modified Eagle Medium (DMEM) supplemented with 10% fetal bovine serum (FBS), 2 mM L-glutamine, 5 U/mL penicillin, and 50 μg/mL streptomycin. Single-guide RNAs (sgRNAs) targeting *NBR1*, *TAX1BP1*, *AZI2*, *TBK1BP1*, *TBK1*, *PPP2R5A*, *PPP2R5B*, and *PPP2R5E* were designed using the CRISPR Design Tool (http://crispr.mit.edu/) and cloned into the pX330-U6-Chimeric_BB-CBh-hSpCas9 plasmid (Addgene #42230). The sgRNA sequences used are listed in the Reagents and Tools Table. To generate *TBK1*, *PPP2R5A*, *PPP2R5B*, and *PPP2R5E* knockout (KO) Huh-1 cells, the respective pX330 constructs were co-transfected with pEGFP-C1 (#6084-1, Clontech Laboratories, Mountain View, CA, USA) into Huh-1 cells using Lipofectamine 3000 (Thermo Fisher Scientific, Waltham, MA, USA). After 48 h, GFP-positive cells were sorted by flow cytometry and expanded. Gene ablation was confirmed by heteroduplex mobility assay and immunoblotting using antibodies specific to each target protein. To establish *PPP2R5A/PPP2R5E* and *PPP2R5B/PPP2R5E* double-knockout (DKO) cells, *PPP2R5E*-KO cells were transfected with pX330 vectors targeting *PPP2R5A* or *PPP2R5B*, respectively. Triple-knockout (TKO) cells for *PPP2R5A/B/E* were generated by further transfection of *PPP2R5A/PPP2R5E*-DKO cells with the *PPP2R5B*-targeting construct.

For gene knockdown, Huh-1 cells were transfected with siGENOME or ON-TARGETplus siRNAs targeting *NBR1* (M-010522-01), *TAX1BP1* (M-016892-01), *AZI2* (M-014092-00), *TBK1BP1* (M-020406-01), *KEAP1* (M-012453-00), *PPP2R5A* (L-009352-00), *PPP2R5B* (L-009366-00), and *PPP2R5E* (L-008531-00), as well as non-targeting siRNA Pool #2 (D-001206-14) and ON-TARGETplus Non-targeting Control Pool (D-001810-01) (all from Dharmacon, Lafayette, CO, USA), using Lipofectamine RNAiMax (13778150, Thermo Fisher Scientific). Cells were incubated for 2 days, followed by a second transfection with the same siRNAs, and cultured for an additional 2 days. The siRNA sequences used are listed in the Reagents and Tools Table.

To monitor autophagic flux, Huh-1 cells were incubated in medium containing 100 nM bafilomycin A_1_ (BafA_1_; BVT-0252-M001, KOM Biomedical, Kyoto, Japan) for 24 h.

*p62*-knockout Huh-1 cells expressing wild-type p62, p62^S403E^, or p62^S403A^ were generated using a retroviral vector, as previously described (Kurusu et al, 2023).

To generate *PPP2R5E* knock-in cells, FLAG-tagged PPP2R5E or its mutant was knocked into the AAVS1 locus of *PPP2R5s*-deficient Huh 1 cells, using a TALEN-based knock-in strategy. TALEN constructs targeting the AAVS1 site (AAVS1-1: TGTCCCCTCCACCCCACA and AAVS1-2: TTTCTGTCACCAATCCTG) were used in combination with donor plasmids: pZDonor-AAVS1-EF1-3×FLAG-PPP2R5E (Puro), or pZDonor-AAVS1-EF1-3×FLAG- PPP2R5E mutant (Puro). Cells were co-transfected with the TALEN plasmids and the corresponding donor vector, and Puromycin-resistant clones were selected and validated by immunoblotting.

To generate *p62*^*S405E/S405E*^ knock-in mouse embryonic stem (mES) cells, we employed the recently developed prime editing system (Anzalone et al, 2019). Details of the procedure for establishing the edited mES cells will be described elsewhere (Mishina and Sakimura, 2007). Briefly, a prime-editing guide RNA (pegRNA) was designed and cloned into a U6 promoter-driven expression vector. The CAG promoter-driven prime editor 2 (PE2) and pegRNA expression plasmids were constructed using pCMV-PE2-P2A-GFP (#132776, Addgene) and hU6-sgRNA plasmid (Yuza et al, 2018). These vectors were co-transfected into RENKA4, a C57BL/6N-derived mES cell line, using Lipofectamine 3000 (Thermo Fisher Scientific). Knock-in mutations were validated by sequencing PCR-amplified genomic DNA from the transfected clones. The pegRNA sequences used are listed in the Reagents and Tools Table.

Mouse embryonic stem (ES) cells were dissociated into single cells using TrypLE Select (12563011, Gibco, Thermo Fisher Scientific) and resuspended at a density of 1 × 10⁵ cells/mL in serum-free MHM (media hormone mix) medium supplemented with B27 Supplement (17504044, Gibco, Thermo Fisher Scientific), leukemia inhibitory factor (LIF; 039-21461, Nakalai Tesque, Kyoto, Japan), and fibroblast growth factor 2 (FGF2; 100-18B, PeproTech, Rocky Hill, NJ, USA) at final concentrations of 1000 U/mL and 20 ng/mL, respectively, to induce neurosphere formation. Neurospheres were passaged every 5–7 days by enzymatic dissociation into single cells and re-cultured under the same conditions. For neuronal differentiation, neurospheres were plated onto poly-L-ornithine- and fibronectin-coated chamber slides and cultured for 7 days in MHM medium supplemented with B27 and 1% fetal bovine serum (FBS). Neuronal differentiation was evaluated by immunostaining with neuronal markers. Cells were authenticated using the STR profile and tested for mycoplasma contamination.

### Generation of *p62*^*S405E/S405E*^-knock-in mice

To generate *p62*^*S405E*^ knock-in mice, we used a prime editing system with conditions nearly identical to those used when producing *p62*^*S351E*^ knock-in mice (Ikeda et al, 2023). Briefly, we designed prime-editing guide RNA (pegRNA) containing the following sequences: spacer sequence, 5’- GCCUUCAUCCGAGAAACCCA -3’; reverse transcription template, 5’-AGAUGCUGGAGAUGG-3’; primer-binding site, 5’- GUUUCUCGGAUGA-3’. The CAG promoter-driven prime editor 2 (PE2) and the U6 promoter-driven pegRNA expression vectors were co-transfected into RENKA (Mishina and Sakimura, 2007), a C57BL/6N-derived mES cell line, using Lipofectamine 2000 (Thermo Fisher Scientific). Knock-in mutations in transfected mES clones were validated by sequencing of PCR products amplified from genomic DNAs. Culture of mES cells and generation of chimeric mice were carried out as previously described (Mishina and Sakimura, 2007). Mice were housed in specific pathogen–free facilities, and the Ethics Review Committee for Animal Experimentation of Juntendo University approved the experimental protocol (2025067).

### Immunoblot analysis

Cells were lysed in ice-cold TNE buffer (50 mM Tris-HCl [pH 7.5], 150 mM NaCl, 1 mM EDTA) containing 1% Triton X-100, 1% NP-40, 1% SDS, and a cOmplete™ EDTA-free protease inhibitor cocktail (5056489001, Roche, Basel, Switzerland). Lysates were centrifuged twice at 15,000 × *g* for 10 min at 4 °C, and the supernatants were collected as whole cell lysates. Protein concentrations were determined using a BCA protein assay (23225, Thermo Fisher Scientific). Samples were boiled in SDS sample buffer, separated by SDS-PAGE, and transferred to polyvinylidene difluoride (PVDF) membranes. Membranes were stained with Ponceau S to confirm protein transfer. Blots were incubated with primary antibodies, followed by HRP-conjugated secondary antibodies, and visualized using chemiluminescence. A full list of primary and secondary antibodies is provided in the Reagents and Tools Table. For fractionation of detergent-insoluble material, cells were lysed in ice-cold TNE buffer containing 0.2% Triton X-100 and a cOmplete™ EDTA-free protease inhibitor cocktail. The lysates were first centrifuged at 200 × *g* for 5 min at 4 °C to remove nuclei and debris, and the resulting supernatant was collected as the post-nuclear lysate. This was then centrifuged at 10,000 × *g* for 10 min at 4 °C to separate the soluble (supernatant) and insoluble (pellet) fractions.

### Immunoprecipitation analysis

For immunoprecipitation analysis, cells were lysed in immunoprecipitation (IP) buffer [20 mM Tris-HCl (pH 7.5), 150 mM NaCl, 1 mM EDTA, 1% NP-40, and 1% Triton X-100] supplemented with protease inhibitor cocktail (Roche, Basel, Switzerland). The lysates were centrifuged at 20,000×*g* for 10 min at 4 °C to remove debris. Supernatants were incubated with 30 μl of anti-FLAG M2 affinity agarose gel (A2220, Merck Millipore) under constant rotation for 2 h at 4 °C. The immunoprecipitates were washed five times with ice-cold IP buffer and boiled in SDS sample buffer for 5 min to elute bound proteins.

### Immunofluorescence analysis

Huh-1 cells and neuronal cells differentiated from ES cells were cultured on coverslips, washed with PBS, and fixed with 4% paraformaldehyde (PFA) for 15 min at room temperature. Cells were then permeabilized with 0.1% Triton X-100 in PBS for 5 min and blocked with 0.1% (w/v) gelatin (G9391, Sigma-Aldrich, Darmstadt, Germany) in PBS for 20 min. Cells were incubated with primary antibodies diluted in blocking buffer for 1 h, washed with PBS, and subsequently incubated with secondary antibodies for another 1 h. Nuclei were stained with Hoechst 33342 (62249, Thermo Fisher Scientific). A full list of primary and secondary antibodies used in this study is provided in the Reagents and Tools Table. Fluorescence images were acquired using an FV3000 confocal laser-scanning microscope equipped with FV31S-SW software (Olympus, Tokyo, Japan) and a UPlanXApo × 60, NA 1.42 oil immersion objective lens. Contrast and brightness were adjusted using Photoshop 2021 (v25.0, Adobe, California, USA). The number and size of p62-positive puncta in each cell, as well as the mean fluorescence intensity of each signal on p62-positive puncta, were quantified using a Benchtop High-Content Analysis System (CQ1, Yokogawa Electric Corp., Tokyo, Japan) and CellPathfinder software (Yokogawa Electric Corp.). For quantification of p62 bodies in neurons, fluorescence images were analyzed using custom scripts written in Python (v3.11), utilizing the OpenCV and NumPy libraries. Images were separated into RGB channels, and fixed intensity thresholds (150 for green/p62 channel and 50 for the red/ubiquitin channel) were applied to generate binary masks. Particles smaller than 0.02 µm^2^ (1.88 pixels^2^, based on a pixel size of 0.1 µm) were excluded. Colocalized puncta were defined as overlapping regions between green and red masks. The number and area of colocalized puncta were quantified and exported for statistical analysis.

### Correlative light and electron microscopy (CLEM)

CLEM analysis was performed as previously described (Kakuta et al, 2023; Tanida et al, 2024). Briefly, cells were cultured on gridded glass coverslips (GC1310, Matsunami Glass, Osaka, Japan). For CLEM shown in Figs. 1B and 3I, cells were fixed with 2% paraformaldehyde and 0.01% glutaraldehyde in PBS for 20 min, followed by permeabilization with 0.1% saponin (199-18653, Wako, Osaka, Japan) for 5 min. For Fig. 1B, cells were stained with antibodies against pS172-TBK1 and p62. For Fig. 3I, cells were stained with antibodies against p62 and PPP2R5E. Secondary antibodies conjugated to Alexa Fluor 555 or 647 were used accordingly. Cells were post-fixed with 2% glutaraldehyde for 1 h, followed by postfixation with 1% osmium tetroxide (OsO₄) for 30 min. Samples were dehydrated and embedded in epoxy resin (Epok812, 02-1003, Oken Shoji, Tokyo, Japan). Ultrathin sections were imaged using a confocal laser scanning microscope (LSM880 with Airyscan, Zeiss, Munich, Germany) equipped with a 63×/1.4 NA oil-immersion objective lens. After fluorescence imaging, the same sections were stained with uranyl acetate and lead citrate and examined using a scanning electron microscope (Helios NanoLab 660, FEI, Oregon, USA).

For 3D-CLEM shown in Figs. 6D and  EV4D, cells were labeled with MitoTracker Red CMXROS (M7512, Invitrogen, Carlsbad, CA, USA) for 30 min and fixed with 2% paraformaldehyde and 0.1% glutaraldehyde in PBS for 30 min. After permeabilization with 0.1% saponin, cells were stained as follows: Cells were stained with p62 and LC3. Z-stack fluorescence images were acquired using the LSM880 with Airyscan. After imaging, cells were post-fixed with glutaraldehyde and OsO₄, followed by en bloc staining with 1% uranyl acetate, dehydration, and resin embedding. Serial block-face imaging of the same areas identified by fluorescence microscopy was performed using a focused ion beam-scanning electron microscope (FIB-SEM; Helios NanoLab 660, FEI). Correlation and 3D reconstruction of FIB-SEM and fluorescence images were carried out using Amira 3D software (FEI).

Detailed antibody information is provided in the Reagents and Tools Table.

### Stimulated emission depletion (STED)

STED imaging was carried out on an INFINITY-line microscope (Abberior Instruments, Göttingen, Germany) built on an inverted IX83 stand (Olympus) with a 60× oil-immersion objective (UPLXAPO60X; NA 1.45, Olympus). Immunostained samples were detected with secondary antibodies conjugated to Abberior STAR ORANGE or STAR RED. STAR ORANGE was excited with a pulsed 560 nm laser, and STAR RED with a pulsed 640 nm laser; both fluorophores were depleted with a pulsed 775 nm STED beam. Emission was collected using an Abberior MATRIX detector. Image acquisition was controlled by Lightbox software (Abberior Instruments). Excitation power, STED intensity and pixel dwell time were adjusted empirically to minimise photobleaching while maintaining optimal signal-to-noise ratios.

### In vitro liquid–liquid phase separation (LLPS) assay

The in vitro liquid–liquid phase separation (LLPS) assay was performed as previously described (Ikeda et al, 2023). To observe p62–8×Ub condensates, mCherry-tagged wild-type p62 was mixed with 8×Ub in the presence or absence of SNAP-tagged factors in LLPS assay buffer (150 mM NaCl, 20 mM HEPES-KOH, pH 7.5, 1 mM TCEP). The following SNAP-tagged proteins were used:

SNAP(488)-NBR1, SNAP(488)-NBR1^D50R^ (labeled with SNAP-Surface 488), SNAP-TAX1BP1, SNAP-AZI2, and SNAP(649)-TBK1 (labeled with SNAP-Surface 649). SNAP-TBK1BP1 was also included as indicated.

The final concentrations were as follows:

- SNAP(488)-NBR1 or SNAP(488)-NBR1^D50R^, 0.1 μM
- SNAP(649)-TBK1, 0.5 μM
- SNAP-TAX1BP1, 0.3 μM
- SNAP-AZI2, 0.5 μM
- SNAP-TBK1BP1, 0.5 μM
- mCherry-p62 wild-type, 16.5 μM
- 8×Ub, 10 μM

Each mixture was spotted onto glass-bottom slides (Cat# 543078, Greiner Bio-One, Frickenhausen, Germany) pre-coated with 0.3% (w/v) bovine serum albumin (Cat# 08587-26, Nacalai Tesque, Kyoto, Japan), and incubated at ~23 °C for 30 min prior to imaging. Fluorescence images were acquired using an FV3000 confocal laser-scanning microscope (Olympus) equipped with 488-, 561-, and 640-nm lasers, and processed using Fiji software (https://imagej.net/software/fiji/downloads) (Schindelin et al, 2012).

### Purification of SNAP-NBR1 using baculovirus expression system

cDNA encoding GST-HRV 3 C protease recognition site–SNAP-tagged NBR1 wild-type or D50R mutant was cloned into the pFastBac Dual expression vector (Cat# 10712024, Thermo Fisher Scientific). The constructs were transformed into DH10Bac competent cells (Cat# 10361012, Thermo Fisher Scientific) to generate recombinant bacmids. Sf9 cells (Cat# B82501, Thermo Fisher Scientific) were maintained in Sf-900 III SFM (Cat# 12658027, Thermo Fisher Scientific) at 27 °C. Two micrograms of purified bacmid DNA were transfected into Sf9 cells using either TransIT-Insect Transfection Reagent (Cat# V6204, Takara Bio, Shiga, Japan) or X-tremeGENE HP (Cat# 6366244001, Roche). After 5 days, recombinant baculoviruses were harvested and further amplified in Sf9 cells. Sf9 cells were then infected with the amplified viruses and harvested after 3–5 days. Cells were lysed in resuspension buffer (50 mM Tris-HCl, pH 8.0, 500 mM NaCl, 1 mM TCEP) by sonication. Lysates were incubated with GST-Accept beads (Cat# 09277-14, Nacalai Tesque) at 4 °C for at least 2 h. After washing three times with the same buffer, SNAP-tagged NBR1 was cleaved from the beads using HRV 3C protease at 4 °C for at least 20 h. The cleaved proteins were concentrated using VIVASPIN 500 (MWCO 50,000) (Cat# VS0131, Sartorius, Göttingen, Germany), and labeled with SNAP-Surface 488 (Cat# S9124S, New England Biolabs, Ipswich, MA, USA) for 1 h. Unbound dye was removed using PD SpinTrap G-25 columns (Cat# 28918004, Cytiva, Marlborough, MA, USA).

### Purification of recombinant proteins using Expi293 expression system

cDNAs encoding SNAP-tagged TBK1, TAX1BP1, AZI2, and TBK1BP1 were cloned into the pOSF (One-STrEP-FLAG) vector. Expi293F cells (Cat# A14527, Thermo Fisher Scientific) were cultured in Expi293 Expression Medium (Cat# A1435101, Thermo Fisher Scientific) at 37 °C with 5% CO₂ at 125 rpm. Cells were transfected with the respective pOSF constructs using the ExpiFectamine 293 Transfection Kit (Cat# A14525, Thermo Fisher Scientific), according to the manufacturer’s protocol. After 2 days, cells were harvested and lysed in resuspension buffer (25 mM Tris-HCl, pH 8.0, 300 mM NaCl, 2 mM DTT) by sonication. Lysates were incubated with Strep-tactin 4Flow high capacity resin (Cat# 2-12500-010, IBA Lifesciences, Göttingen, Germany) at 4 °C for at least 30 min. After washing five times with the same buffer, proteins were eluted with 2.5 mM desthiobiotin (Cat# D1411-500MG, Sigma-Aldrich, St. Louis, MO, USA). Purified SNAP-TBK1 was labeled with SNAP-Surface 649 (Cat# S9159S, New England Biolabs) as described above.

### Plasmids for recombinant protein expression in bacteria

The pGEX6P-1 vector (GE Healthcare) was used for the expression of both KEAP1 and PPP2R5E proteins in *E. coli*. To improve protein solubility, a SNAP tag was inserted upstream of both coding sequences. For high-purity protein preparation, the SNAP–KEAP1 construct was designed with an N-terminal glutathione S-transferase (GST) tag and a C-terminal Twin-Strep tag (SAWSHPQFEKGGGSGGGSGGSAWSHPQFEK). To allow tag removal by HRV 3 C protease after purification, a LEVLFQGP recognition sequence was introduced immediately downstream of the GST tag and upstream of the Twin-Strep tag. The genes were amplified by PCR, and all insertions were performed using the NEBuilder HiFi DNA Assembly Kit (New England Biolabs). The final constructs were verified by DNA sequencing.

### Protein expression and purification for HS-AFM imaging

*E. coli* strain BL21(DE3) was used for the expression of both SNAP–KEAP1 and SNAP–PPP2R5E. Protein expression was induced with 0.1 mM isopropyl β-D-thiogalactopyranoside (IPTG) at 16 °C overnight. Cells were harvested by centrifugation and lysed by sonication in Buffer A (50 mM HEPES–NaOH, pH 8.0, 500 mM NaCl) supplemented with 1 mM EDTA, 1 mM Tris(2-carboxyethyl)phosphine (TCEP), 1 mM phenylmethylsulfonyl fluoride (PMSF), a protease inhibitor cocktail (Nacalai Tesque), DNase I (Takara), and RNase A (Nacalai Tesque). For SNAP–KEAP1, after removal of insoluble debris by centrifugation, the supernatant was applied to Strep-Tactin XT 4Flow high-capacity resin (IBA Lifesciences). The resin was washed with Buffer A, and bound proteins were eluted with 50 mM D-biotin in Buffer A. The eluate was subsequently applied to GST-accept resin (Nacalai Tesque). For SNAP–PPP2R5E, after removal of insoluble debris by centrifugation, the supernatant was applied to GST-accept resin (Nacalai Tesque). After washing with Buffer A, HRV 3 C protease carrying a GST tag was added to the resin, and on-column cleavage was performed for ~16 h at 4 °C. The cleaved SNAP–KEAP1 and SNAP–PPP2R5E proteins were collected and concentrated to ~80 μM in Buffer A supplemented with 10% glycerol using Vivaspin 500 concentrators (GE Healthcare). The presence and purity of SNAP–KEAP1 and SNAP–PPP2R5E were confirmed by SDS–PAGE (Appendix Fig. S2). Purified proteins were stored at −80 °C until use.

### Sample preparation for HS-AFM imaging

For HS-AFM imaging of SNAP-KEAP1 or SNAP-PPP2R5E, each protein was diluted to 10 nM in imaging buffer (20 mM HEPES-NaOH pH 7.4, 100 mM NaCl) immediately before observation and deposited onto freshly cleaved mica mounted on a glass stage (diameter 1.5 mm, height 2 mm). For imaging of KEAP1–PPP2R5E interactions, 10 nM SNAP-KEAP1 and 10 nM SNAP-PPP2R5E were mixed in imaging buffer in a 0.2-mL tube immediately before observation and deposited onto freshly cleaved mica. After incubation for 3–5 min, the mica surface was rinsed and immersed in the liquid cell containing ~100 μL of imaging buffer.

### HS-AFM imaging

HS-AFM observations were performed as previously described (Uchihashi et al, 2012). Images were acquired in tapping mode using a sample-scanning HS-AFM instrument (MS-NEX, Research Institute of Biomolecule Metrology Co., Ltd.). Cantilevers (USC-F1.2-k0.15, NanoWorld) with dimensions of ~7 μm length, ~2 μm width, and ~0.08 μm thickness, and equipped with electron beam–deposited (EBD) tips (tip radius <10 nm), were used. The resonant frequency and spring constant were 1.2 MHz in air and 0.15 N/m, respectively. Imaging conditions were as follows: scan size, 80 × 80 nm²; pixel resolution, 80 × 80; imaging rate, 5 frames/s. Imaging was performed at 23 °C. HS-AFM images were processed and analyzed using Kodec and ImageJ software.

### siRNA-based screening

Huh-1 cells were transfected with ON-TARGETplus siRNAs (Dharmacon, Lafayette, CO, USA) targeting each catalytic and regulatory subunit of PP2A. After 48 h, a second transfection was performed using the same siRNA. Cells were harvested and lysed, and the lysates were subjected to SDS-PAGE, followed by immunoblot analysis using antibodies against p62 and Ser403-phosphorylated p62. The siRNA target sequences used for the screen are listed in the Reagents and Tools Table.

### Fluorescence recovery after photobleaching analysis (FRAP)

Huh-1 cells expressing monomeric GFP (mGFP)-tagged p62, as well as *p62*-knockout Huh-1 cells expressing mGFP-p62, mGFP-p62^S403E^, or mGFP-p62^S403A^, were generated using a retroviral vector, as previously described (Kurusu et al, 2023). For FRAP analysis, p62 bodies positive for mGFP fluorescence were photobleached using a 488 nm laser. For multi-spot bleaching, three to five circular regions of interest (ROIs) covering the entire p62 bodies were simultaneously photobleached, while carefully minimising bleaching of surrounding areas. Following bleaching, time-lapse images were acquired at around ~10-s intervals using the Z-drift compensation (ZDC) system to maintain continuous focus and monitor fluorescence recovery. Because the p62 bodies occasionally shifted position between frames, ROI positions were manually adjusted before fluorescence intensity was quantified. To generate recovery curves, the fluorescence intensity of mGFP-p62 at each time point was normalized by subtracting the background values to remove experimental noise, and all values were divided by the pre-bleached baseline measure. The normalized intensities were plotted over time to obtain fluorescence recovery curve for each p62 body. Image acquisition and analysis were performed using FV31S-SW software (version 2.6.1.243) and cellSens Dimension Desktop (version 3.2, Build 23706) (Olympus). The mobile fraction (Mf) was calculated from individual measurements using the following equation: Mf = (F∞ − F₀) / (Fᵢ − F₀), where F∞ is the fluorescence intensity at recovery plateau, Fᵢ is the initial fluorescence intensity before bleaching, and F₀ is the fluorescence intensity immediately after bleaching.

### Surface tension estimation from condensate shape fluctuations

To investigate the dynamics of condensate surface fluctuations, we adopted an analysis framework based on our previous work (Shimobayashi et al, 2025). Briefly, we defined a polar coordinate system $(r,\varphi )$ centered at the condensate’s center of mass. The angular coordinate *φ* was discretized into *N* points with spacing $\Delta \varphi \left(\right.=2\pi /N$). The camera-based intensity profile was interpolated into this coordinate system using MATLAB’s interp2 function.

The condensate contour $r(r,\varphi )$ was extracted as the location where the intensity crossed a global threshold, determined with subpixel accuracy via linear interpolation. Assuming a circular time-averaged interface, we defined the fluctuation at time delay $\Delta t$ as:

$\delta {r}(\varphi ,\Delta t)=r(\varphi ,t+\Delta t)-r(\varphi ,t)$. This fluctuation was expanded into a Fourier series:

$$\delta \,r\left(n,\Delta t\right)={\sum}_{m=0}^{N-1}\delta r\left(m,\Delta t\right){e}^{{im}\varphi_n},$$

The coefficients $\delta {r}\left(m,\Delta t\right)$ were computed using MATLAB’s Fast Fourier Transform (FFT). Here, *m* is the azimuthal mode index of the fluctuation.

To evaluate the fluctuation spectrum, we calculated the ensemble average:

$$\left\langle {\left|\Delta {B}_{m}(\Delta t)\right|}^{2}\right\rangle \frac{1}{M}{\sum}_{j=1}^{M}{\left|\delta {r}_{j}(m,\Delta t)\right|}^{2}$$

where *M* is the ensemble size and $\delta {r}_{j}(m,\Delta t)$ is the *m*-th Fourier component of the *j*th realization.

The theoretical expression for the spectrum of thermally driven surface fluctuations, $\left\langle {\left|\Delta {B}_{m}(\Delta t)\right|}^{2}\right\rangle$, is derived as follows:

$$\left\langle {\left|\Delta {B}_{m}(\Delta t)\right|}^{2}\right\rangle = 	 {\sum}_{n=1}^{\infty }\frac{2{r}_{0}^{2}{k}_{B}T}{{\varepsilon }_{n+m}}{\left({N}_{n+m,m}{P}_{n+m}^{m}\left(0\right)\right)}^{2}\\ 	 \times \left(1-\exp \left(-\frac{{\varepsilon }_{n+m}\Delta {{{\rm{t}}}}}{{r}_{0}^{2}\xi }\right)\right),$$

$${\varepsilon }_{l}=(l\left(l+1\right)-2)\left[\kappa l\left(l+1\right)+\gamma {r}_{0}^{2}\right]$$

Here, ${N}_{l,m}=\sqrt{\frac{2l+1}{4\pi }\frac{\left(l-m\right)!}{\left(l+m\right)!}}$. ${P}_{l}^{m}$ is the associated Legendre polynomial. The other parameters in the equations are: ${r}_{0}$, the mean condensate radius; ${k}_{B}$, the Boltzmann constant; *T*, the temperature; *γ*, the surface tension; *κ*, the bending modulus; and *ξ*, the friction coefficient.

Therefore, by fitting this theoretical expression to experimentally measured fluctuation spectra, the surface tension (*γ*) can be quantitatively determined.

### Immunoelectron microscopy

For immunoelectron microscopy, *PPP2R5s* knockdown Huh-1 cells were fixed with 2% paraformaldehyde-0.1% glutaraldehyde in PBS. Cells were then embedded in LR white resin (Electron Microscopy Sciences, Hatfield, PA, USA), and polymerized by ultraviolet radiation at −20 °C for 48 h. Ultrathin sections of LR white-embedded samples were immunolabeled as below. Sections on nickel grids were incubated with 1% BSA in PBS for 20 min, with anti-p62 antibody (BD, #610832) for 2 h, and with 12 nm colloidal gold anti-mouse IgG (Jackson ImmunoResearch Laboratories, 715-205-150) for 2 h. These sections were stained with uranyl acetate and lead citrate, then examined with a transmission electron microscope JEM-1400 Flash (JEOL, Tokyo, Japan).

### Quantification of LC3-positive structures on p62 bodies

Z-stack images of immunostained control and *PPP2R5s*-knockdown Huh-1 cells were acquired using an LSM880 confocal microscope equipped with Airyscan (Zeiss). The images were processed using maximum intensity projection and analyzed using ImageJ software (Schindelin et al, 2012). The volume of the p62 bodies larger than 100 square pixels was estimated from the lengths of the major and minor axes of the projected ellipse of the p62 bodies. Since it was difficult to accurately measure the length of third *Z* axis, it was assumed to be the same as the minor axis. The number of LC3-positive puncta in each p62 body was counted manually.

### Nuclear magnetic resonance (NMR)

To construct an expression plasmid encoding N-terminal GB1-His-tagged PPP2R5E (131-145), the gene was amplified by PCR and cloned into a pGBHPS vector (Kobashigawa et al, 2009). Uniformly ^15^N-labeled GB1-hexahistidine-tagged PPP2R5E (131–145) was overexpressed in *E coli*. BL21(DE3) cells cultured in a minimal M9 medium containing 1.0 g/L ^15^NH_4_Cl (Cambridge Isotope Laboratories). ^15^N-labeled GB1-hexahistidine-tagged PPP2R5E (131-145) was purified using Ni-NTA agarose (QIAGEN) and subsequently buffer-exchanged into 20 mM HEPES (pH 7.0), 150 mM NaCl solution using a HiTrap Desalting column (Cytiva). KEAP1-DC was purified as reported previously (PMID 24011591). All NMR measurements were performed at 25˚C using Bruker 800 MHz AVANCE NEO NMR equipped with a CPTCI probe with 20 mM HEPES-NaOH, 150 mM NaCl pH 7.0 as buffer. NMR signal assignment was performed by HNCA spectra with natural abundance ^13^C using ^15^N-labeled GB1-hexahistidine-tagged PPP2R5E (131-145) samples adjusted to 1.6 mM, which could be assigned for all main chain amide signals derived from GB1 and PPP2R5E (Fig. 4D). In titration experiments, 0.1, 0.2, 0.3, 2, and 10 molar equivalents of KEAP1-DC were added to ^15^N-labeled GB1-hexahistidine-tagged PPP2R5E (131-145) adjusted to 10 µM, and [^1^H-^15^N] HSQC was measured under each condition.

### p62 body purification and LC-MS/MS sample preparation

The purification of p62 bodies was carried out essentially as described previously (Kurusu et al, 2023), with minor modifications. mEGFP-p62–expressing Huh1 cells were harvested from twelve 10-cm culture dishes. The p62 body fraction was sorted using a cell sorter (SH800; Sony) equipped with a 70-μm nozzle. mEGFP-p62 bodies were detected based on back-scattered light and green fluorescence. The collected fractions were concentrated by centrifugation at 10,000×*g* for 7 min, and proteins were further concentrated by methanol–chloroform precipitation. The resulting protein pellet was resuspended in 35 μl of buffer, of which 2.5 μl was freeze-dried. Protein concentration was determined using the Micro BCA Protein Assay (Thermo Fisher Scientific).

Thirty micrograms of both pre- and post-fraction samples were digested with a Trypsin/LysC mix (Thermo Fisher Scientific) using S-Trap columns (ProtiFi, Huntington, NY, USA). The resulting peptides were eluted, dried in a SpeedVac concentrator, and reconstituted in 0.1% trifluoroacetic acid (TFA) and 5% acetonitrile (ACN). Peptide concentrations were determined using the Quantitative Peptide Assay kit (Thermo Fisher Scientific).

### DIA analysis and semi-absolute quantification of protein abundance

Data-independent acquisition (DIA) analysis was carried out following the protocol reported previously (Takada et al, 2025). The data were analyzed using the CHIMERYS 4.0 intelligent search algorithm (MSAID GmbH) implemented in Thermo Scientific™ Proteome Discoverer™ 3.2. A predicted spectral library was generated from the human FASTA database (taxonomy ID = 9606, version 2025-02-05) using the INFERYS™ deep learning framework (MSAID GmbH) for all tryptic +1 to +6 peptides between 7 and 30 amino acids in length. The maximum number of missed cleavage sites for trypsin was set to 2. Carbamidomethylation of cysteine was set as a fixed modification, while oxidation (Met), GlyGly (Lys), and phosphorylation (Ser, Thr, and Tyr) were set as variable modifications. Peptide identifications were filtered at a false discovery rate (FDR) < 0.01. Protein abundances were estimated using the Top3 (Hi-3) method (Silva et al, 2006).

### Statistical analysis

Statistical analyses were performed using the unpaired *t* test (Welch’s *t* test), Tukey’s multiple comparison test, or the Mann–Whitney *U* test, as appropriate. One-way ANOVA was used for multiple group comparisons. All analyses were conducted using GraphPad Prism 9 (GraphPad Software, San Diego, CA, USA). All tests were two-sided, and *P* values less than 0.05 were considered statistically significant. Exact *P* values are indicated; for values below the detection limit of the software, *P* < 1 × 10⁻¹⁵ is reported.

## Supplementary information

Appendix

Peer Review File

Movie EV1

Movie EV2

Movie EV3

Movie EV4

Movie EV5

Movie EV6

Movie EV7

Source data Fig. 1

Source data Fig. 2

Source data Fig. 3

Source data Fig. 4

Source data Fig. 5

Source data Fig. 6

Source data Fig. 7

Figures EV1-EV5 Source Data

Expanded View Figures

## Data Availability

The mass spectrometry proteomics data have been deposited to the ProteomeXchange Consortium via the PRIDE partner repository with the dataset identifier PXD074538 (https://www.ebi.ac.uk/pride/archive/projects/PXD074538). NMR chemical shift assignments have been deposited in the Biological Magnetic Resonance Data Bank (BMRB) under accession number 53579. All data supporting the findings of this study are provided within the paper. The source data of this paper are collected in the following database record: biostudies:S-SCDT-10_1038-S44318-026-00785-1.
