# Supplementary material for: Phosphorylation tunes p62 condensates to drive autophagic degradation of ubiquitinated proteins
Source: EMBO J. 2026 May 5;45(12):4061–93. doi: 10.1038/s44318-026-00785-1 (PMC13270050; doi:10.1038/s44318-026-00785-1)
Supplement: Supplementary file 1 — Appendix [file 44318_2026_785_MOESM1_ESM.pdf]

Phosphorylation tunes p62 condensates to drive autophagic degradation of ubiquitinated proteins

Appendix Contexts

Appendix Figure S1	Page 2
Appendix Figure S2	Page 3
Appendix Figure S3	Page 4

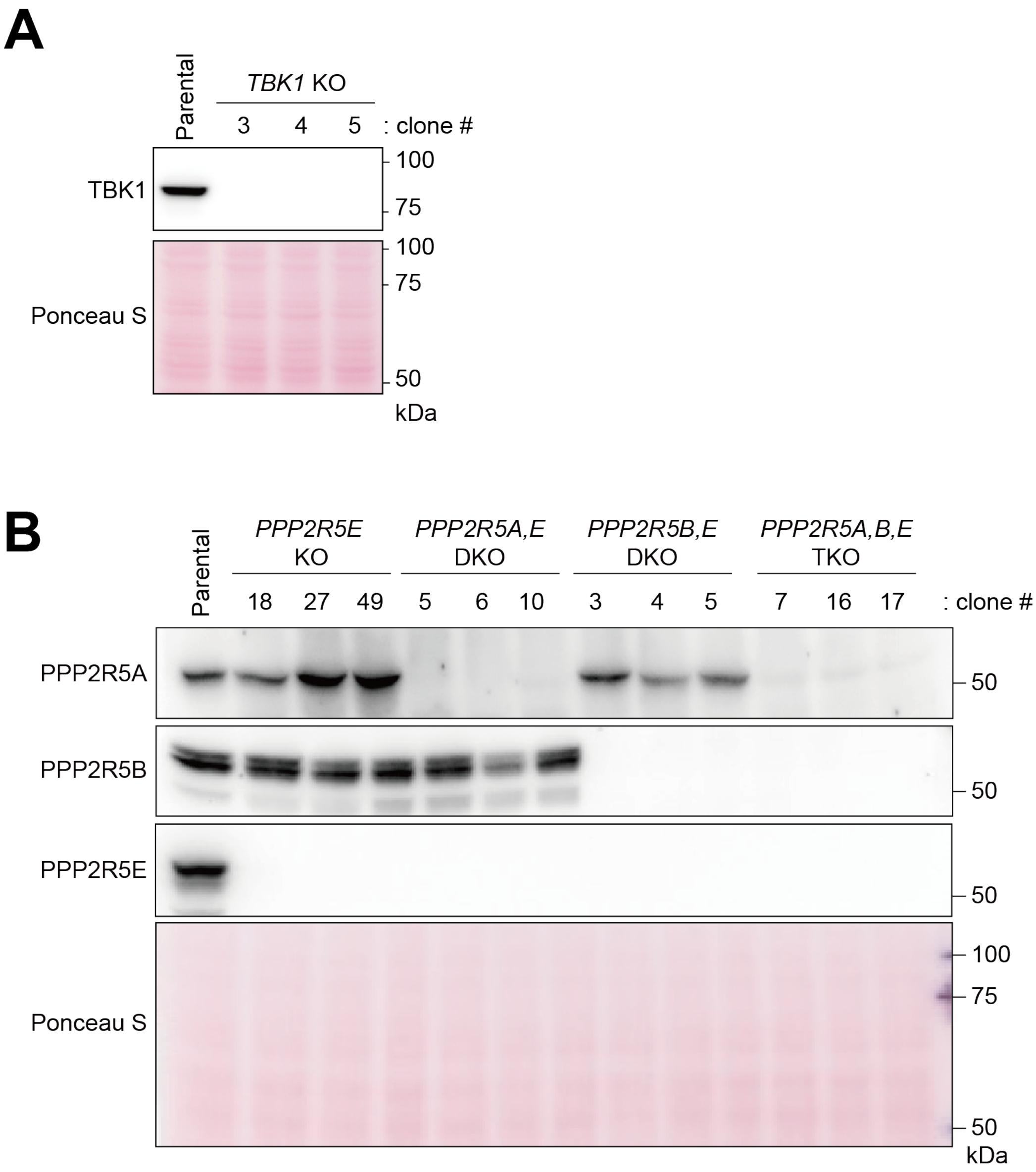

Appendix Figure S1. Generation of knockout cell lines.

(**A, B**) Immunoblot analysis of *TBK1*-knock-out (A) and *PPP2R5A/B/E* triple-knock-out (B) Huh-1 cells using the indicated antibodies. Results are representative of three independent experiments.

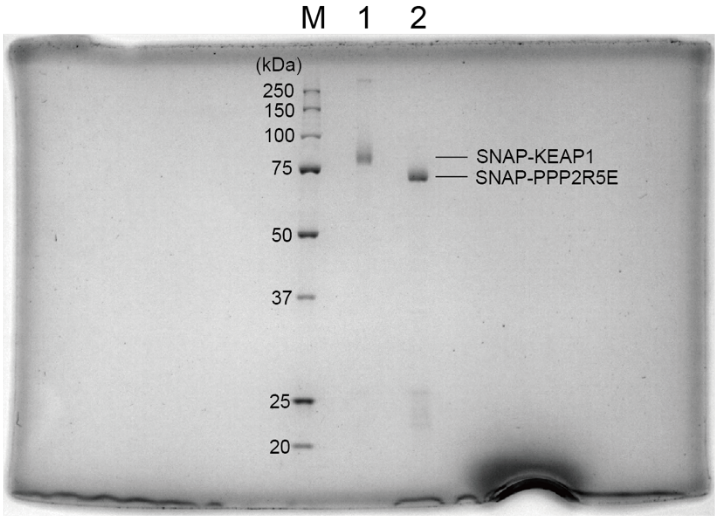

Appendix Figure S2. SDS-PAGE analysis of purified SNAP-KEAP1 and SNAP-PPP2R5E. Purified SNAP-KEAP1 (90.1 kDa) and SNAP-PPP2R5E (74.1 kDa) were analyzed by 12% SDS-PAGE. Lane M, molecular weight marker (Precision Plus Protein™ Unstained Protein Standards (Bio-Rad)); lane 1, SNAP-KEAP1; lane 2, SNAP-PPP2R5E.

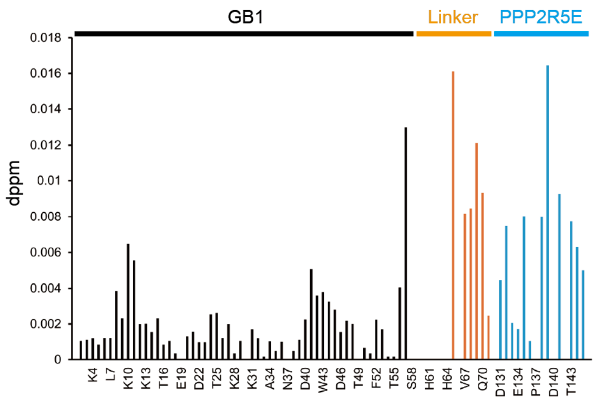

Appendix Figure S3. The chemical shift perturbations.

The chemical shift perturbations upon the addition of KEAP1 in Figure 4E are plotted for each residue.
