# Supplementary material for: Phosphorylation tunes p62 condensates to drive autophagic degradation of ubiquitinated proteins
Source: EMBO J. 2026 May 5;45(12):4061–93. doi: 10.1038/s44318-026-00785-1 (PMC13270050; doi:10.1038/s44318-026-00785-1)
Supplement: Supplementary file 3 — Movie EV1 [file 44318_2026_785_MOESM3_ESM.zip › Movie EV1/Movie EV1_legend.docx]

**Movie EV1. HS-AFM imaging of SNAP-PPP2R5E.**

Height scale: 0–3 nm. Scale bar: 20 nm.
