# Supplementary material for: Phosphorylation tunes p62 condensates to drive autophagic degradation of ubiquitinated proteins
Source: EMBO J. 2026 May 5;45(12):4061–93. doi: 10.1038/s44318-026-00785-1 (PMC13270050; doi:10.1038/s44318-026-00785-1)
Supplement: Supplementary file 4 — Movie EV2 [file 44318_2026_785_MOESM4_ESM.zip › Movie EV2/Movie EV2_legend.docx]

**Movie EV2. HS-AFM imaging of SNAP-KEAP1.**

Height scale: 0–4 nm. Scale bar: 20 nm.
