# Supplementary material for: Phosphorylation tunes p62 condensates to drive autophagic degradation of ubiquitinated proteins
Source: EMBO J. 2026 May 5;45(12):4061–93. doi: 10.1038/s44318-026-00785-1 (PMC13270050; doi:10.1038/s44318-026-00785-1)
Supplement: Supplementary file 5 — Movie EV3 [file 44318_2026_785_MOESM5_ESM.zip › Movie EV3/Movie EV3_legend.docx]

**Movie EV3. HS-AFM imaging of KEAP1–PPP2R5E interaction.**

Height scale: 0–4 nm. Scale bar: 20 nm.
