# Supplementary material for: Phosphorylation tunes p62 condensates to drive autophagic degradation of ubiquitinated proteins
Source: EMBO J. 2026 May 5;45(12):4061–93. doi: 10.1038/s44318-026-00785-1 (PMC13270050; doi:10.1038/s44318-026-00785-1)
Supplement: Supplementary file 7 — Movie EV5 [file 44318_2026_785_MOESM7_ESM.zip › Movie EV5/Movie EV5_legend.docx]

**Movie EV5. 3D CLEM of p62-autophagosome structures in *PPP2R5s* knockdown cells.**

Three-dimensional CLEM of p62 bodies and associated autophagosomes in *PPP2R5* siRNA-treated Huh-1 cells. Scale bar, 700 nm.
