# Supplementary material for: Phosphorylation tunes p62 condensates to drive autophagic degradation of ubiquitinated proteins
Source: EMBO J. 2026 May 5;45(12):4061–93. doi: 10.1038/s44318-026-00785-1 (PMC13270050; doi:10.1038/s44318-026-00785-1)
Supplement: Supplementary file 8 — Movie EV6 [file 44318_2026_785_MOESM8_ESM.zip › Movie EV6/Movie EV6_legend.docx]

**Movie EV6. 3D CLEM of p62–autophagosome structures in *p62*-knockout cells expressing p62^S403E^.**

Three-dimensional CLEM analysis of p62 bodies and their associated autophagosomes in *p62*-deficient Huh-1 cells expressing the phosphomimetic mutant p62^S403E^. Scale bar, 1000 nm.
