# Supplementary figures and images for: Phosphorylation tunes p62 condensates to drive autophagic degradation of ubiquitinated proteins (part 1 of 2)
Source: EMBO J. 2026 May 5;45(12):4061–93. doi: 10.1038/s44318-026-00785-1 (PMC13270050; doi:10.1038/s44318-026-00785-1)

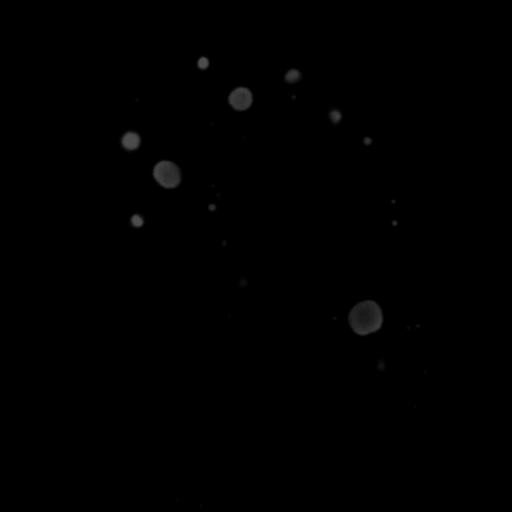

Supplement: Supplementary file 10 — Source data Fig. 1 [file 44318_2026_785_MOESM10_ESM.zip › Source data Fig. 1/1A/1A_TBK1-p62_.p62.tif]

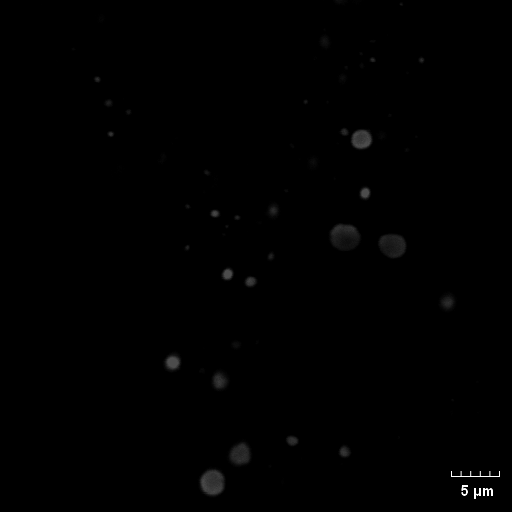

Supplement: Supplementary file 10 — Source data Fig. 1 [file 44318_2026_785_MOESM10_ESM.zip › Source data Fig. 1/1A/1A_TBK1(pS172)-p62_p62.tif]

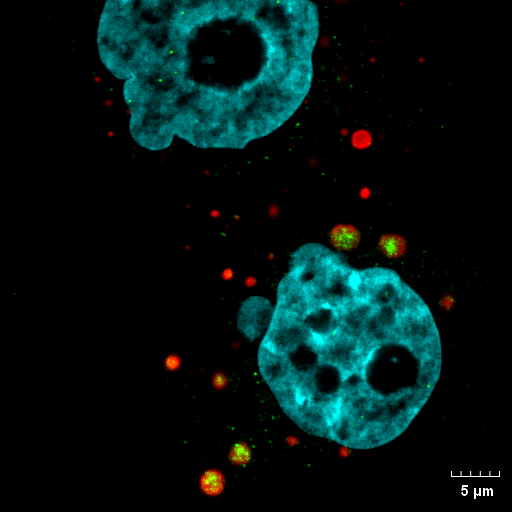

Supplement: Supplementary file 10 — Source data Fig. 1 [file 44318_2026_785_MOESM10_ESM.zip › Source data Fig. 1/1A/1A_TBK1(pS172)-p62_Merge.tif]

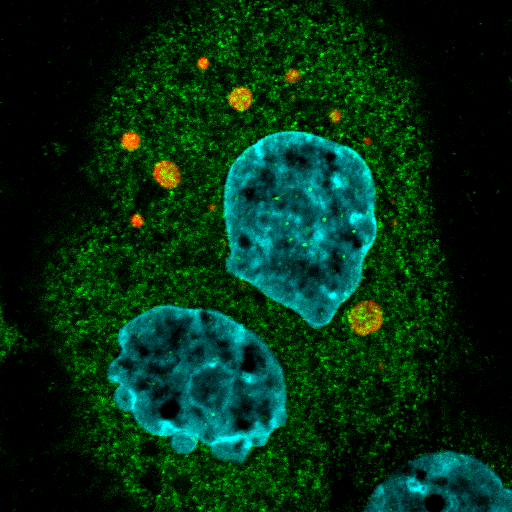

Supplement: Supplementary file 10 — Source data Fig. 1 [file 44318_2026_785_MOESM10_ESM.zip › Source data Fig. 1/1A/1A_TBK1-p62_Merge.tif]

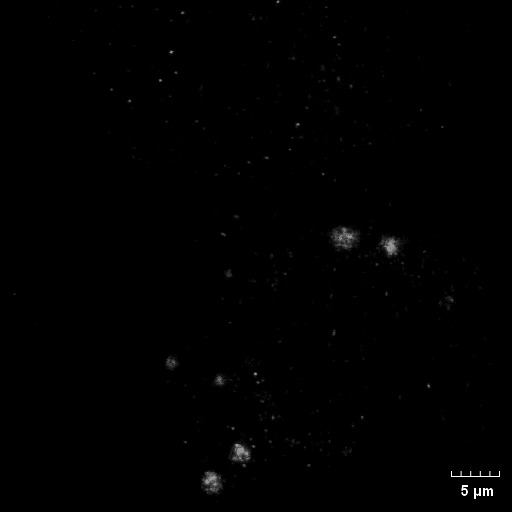

Supplement: Supplementary file 10 — Source data Fig. 1 [file 44318_2026_785_MOESM10_ESM.zip › Source data Fig. 1/1A/1A_TBK1(pS172)-p62_TBK1(pS172).tif]

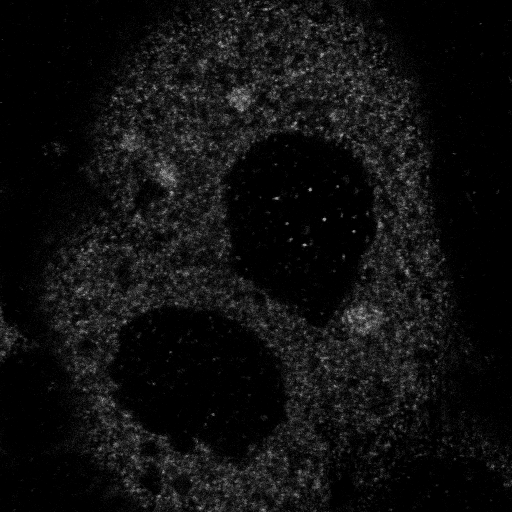

Supplement: Supplementary file 10 — Source data Fig. 1 [file 44318_2026_785_MOESM10_ESM.zip › Source data Fig. 1/1A/1A_TBK1-p62_TBK1.tif]

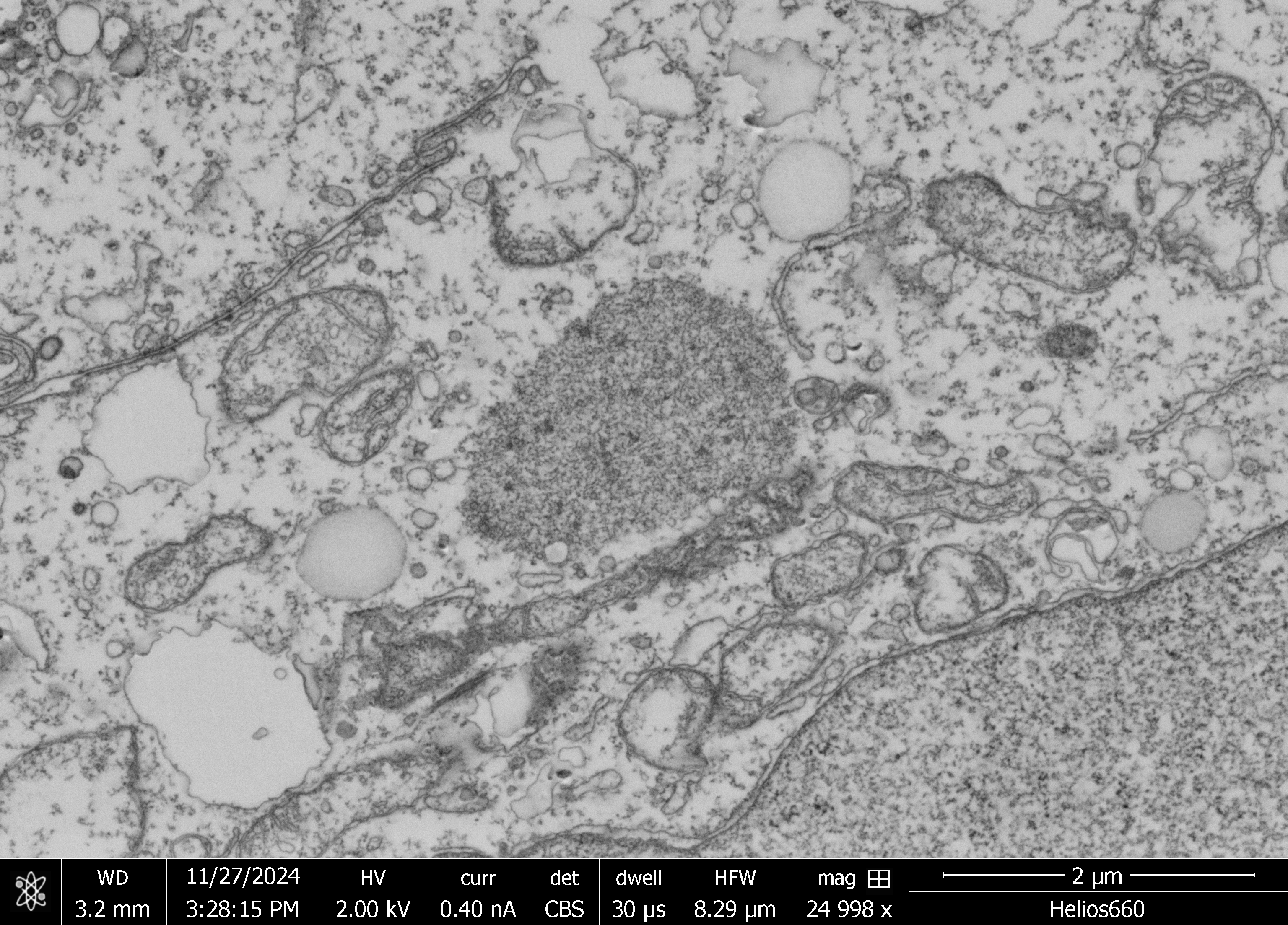

Supplement: Supplementary file 10 — Source data Fig. 1 [file 44318_2026_785_MOESM10_ESM.zip › Source data Fig. 1/1B/1B_EM_Image-2.tif]

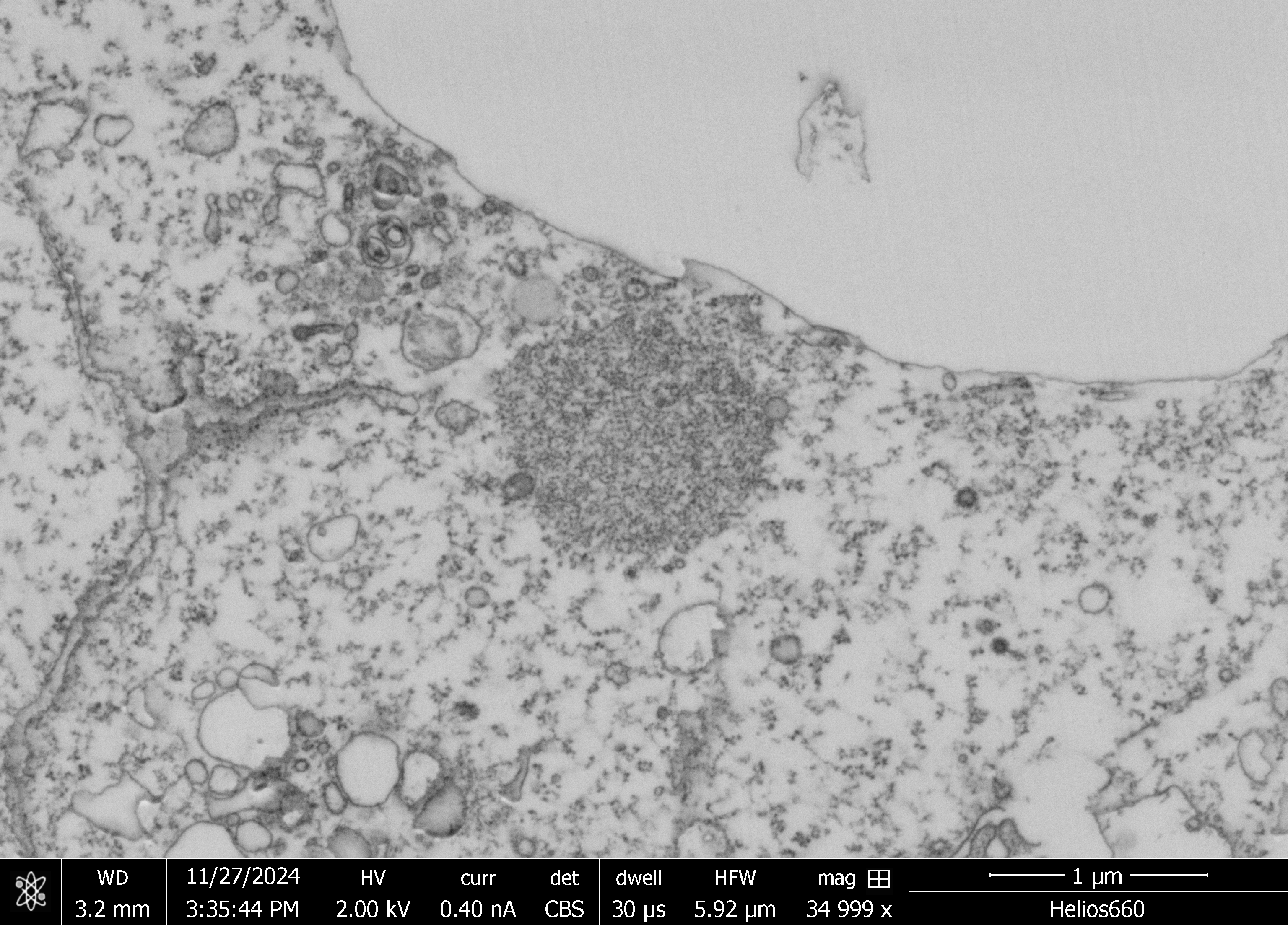

Supplement: Supplementary file 10 — Source data Fig. 1 [file 44318_2026_785_MOESM10_ESM.zip › Source data Fig. 1/1B/1B_EM_Image-1.tif]

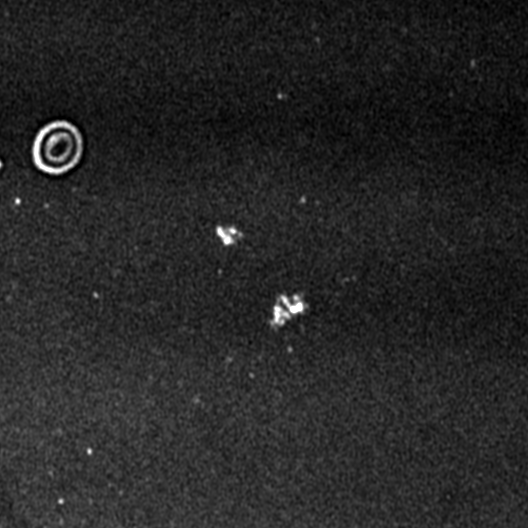

Supplement: Supplementary file 10 — Source data Fig. 1 [file 44318_2026_785_MOESM10_ESM.zip › Source data Fig. 1/1B/1B_Fluorescence_overview.tif]

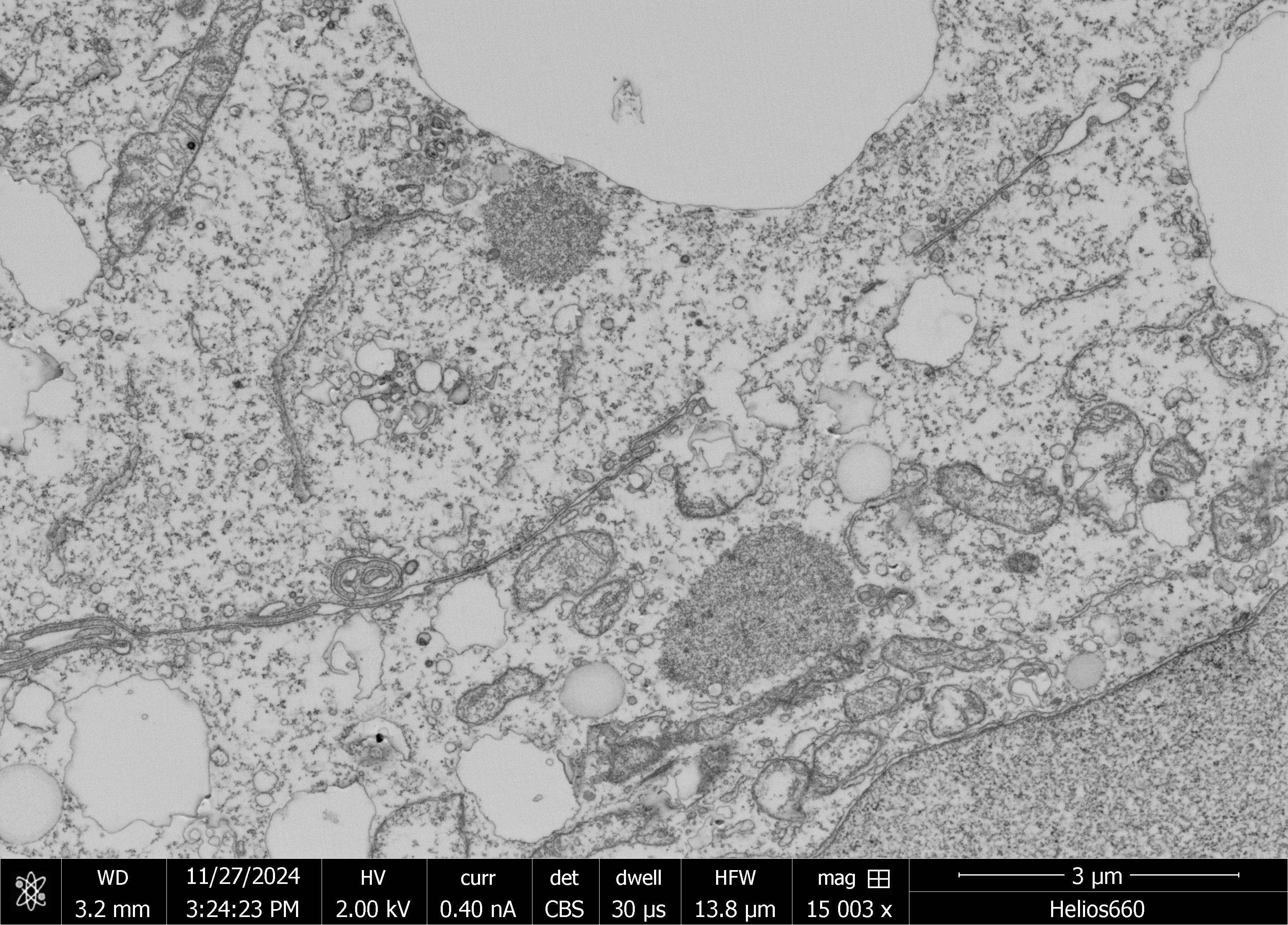

Supplement: Supplementary file 10 — Source data Fig. 1 [file 44318_2026_785_MOESM10_ESM.zip › Source data Fig. 1/1B/1B_EM_overview.tif]

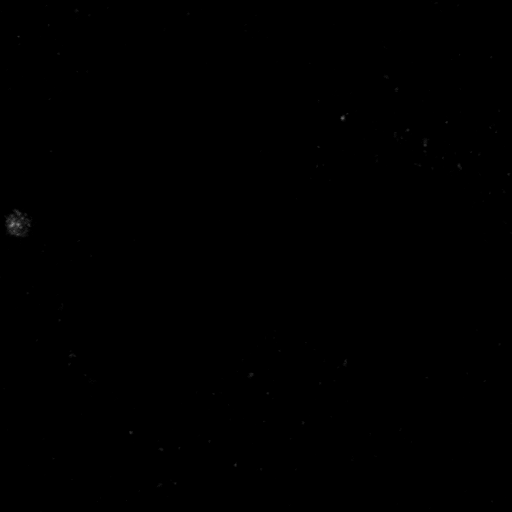

Supplement: Supplementary file 10 — Source data Fig. 1 [file 44318_2026_785_MOESM10_ESM.zip › Source data Fig. 1/1E/1E_TAX1BP1KD_TBK1(pS172)-p62_TBK1(pS172).tif]

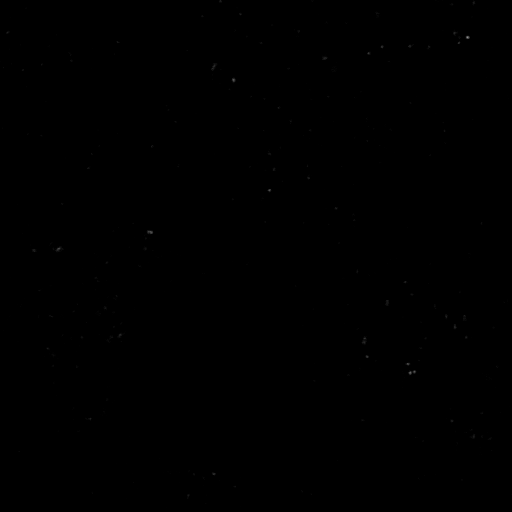

Supplement: Supplementary file 10 — Source data Fig. 1 [file 44318_2026_785_MOESM10_ESM.zip › Source data Fig. 1/1E/1E_AZI2KD_TBK1(pS172)-p62_TBK1(pS172).tif]

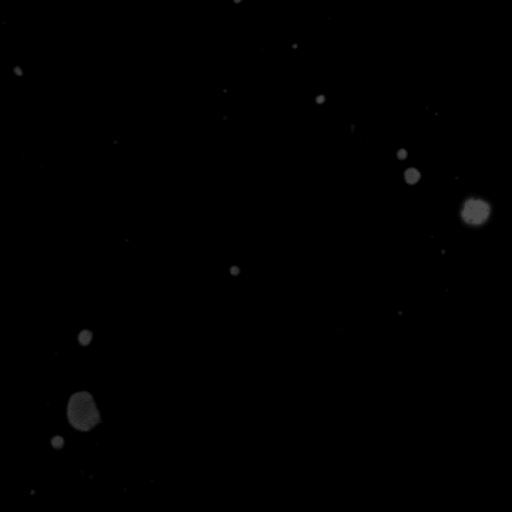

Supplement: Supplementary file 10 — Source data Fig. 1 [file 44318_2026_785_MOESM10_ESM.zip › Source data Fig. 1/1E/1E_TBK1BP1KD_TBK1(pS172)-p62_p62.tif]

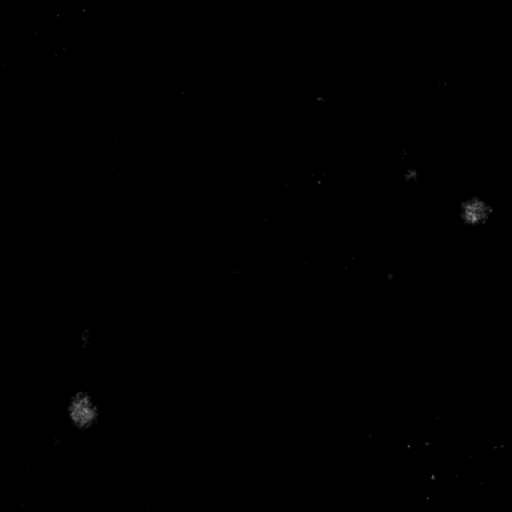

Supplement: Supplementary file 10 — Source data Fig. 1 [file 44318_2026_785_MOESM10_ESM.zip › Source data Fig. 1/1E/1E_TBK1BP1KD_TBK1(pS172)-p62_TBK1(pS172).tif]

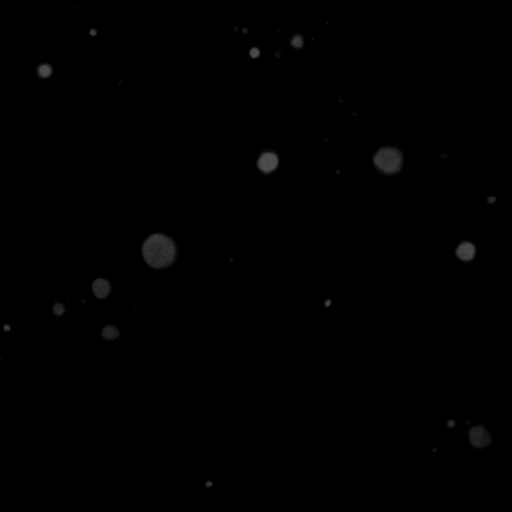

Supplement: Supplementary file 10 — Source data Fig. 1 [file 44318_2026_785_MOESM10_ESM.zip › Source data Fig. 1/1E/1E_AZI2KD_TBK1(pS172)-p62_p62.tif]

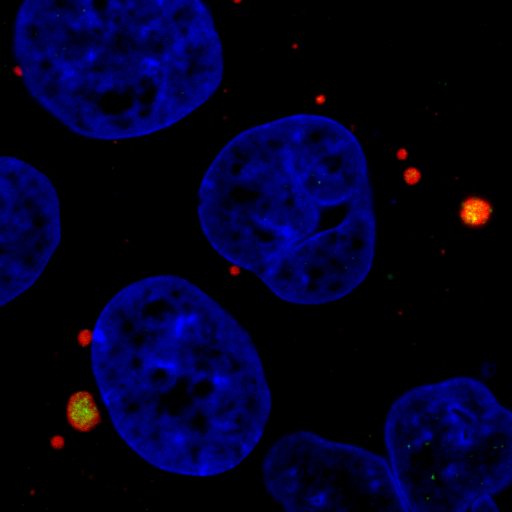

Supplement: Supplementary file 10 — Source data Fig. 1 [file 44318_2026_785_MOESM10_ESM.zip › Source data Fig. 1/1E/1E_TBK1BP1KD_TBK1(pS172)-p62_Merge.tif]

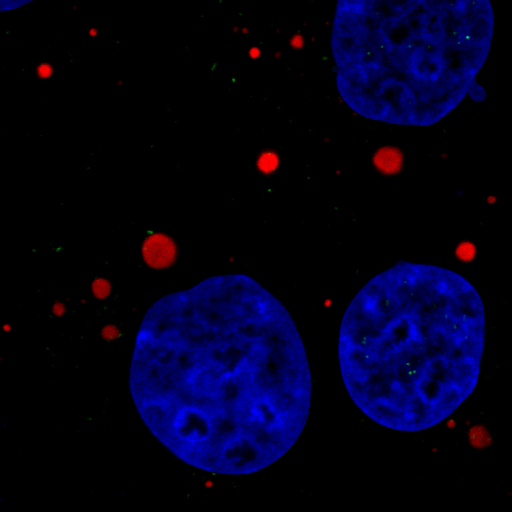

Supplement: Supplementary file 10 — Source data Fig. 1 [file 44318_2026_785_MOESM10_ESM.zip › Source data Fig. 1/1E/1E_AZI2KD_TBK1(pS172)-p62_Merge.tif]

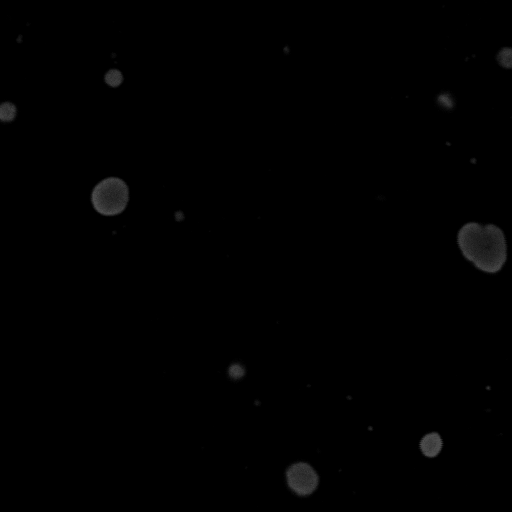

Supplement: Supplementary file 10 — Source data Fig. 1 [file 44318_2026_785_MOESM10_ESM.zip › Source data Fig. 1/1E/1E_NT_TBK1(pS172)-p62_p62.tif]

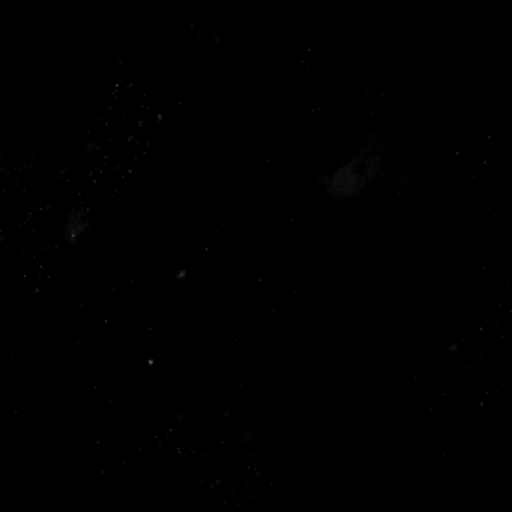

Supplement: Supplementary file 10 — Source data Fig. 1 [file 44318_2026_785_MOESM10_ESM.zip › Source data Fig. 1/1E/1E_NBR1KD_TBK1(pS172)-p62_TBK1(pS172).tif]

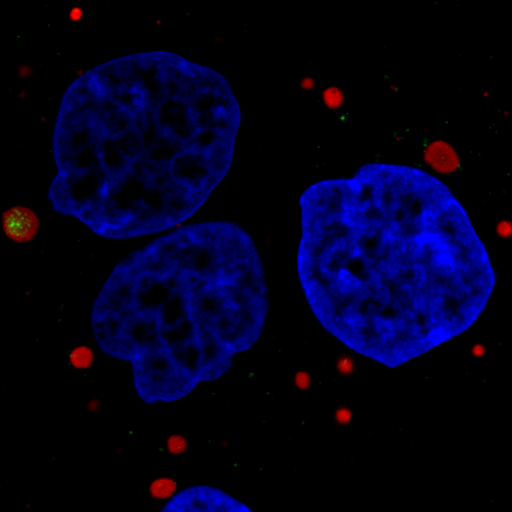

Supplement: Supplementary file 10 — Source data Fig. 1 [file 44318_2026_785_MOESM10_ESM.zip › Source data Fig. 1/1E/1E_TAX1BP1KD_TBK1(pS172)-p62_Merge.tif]

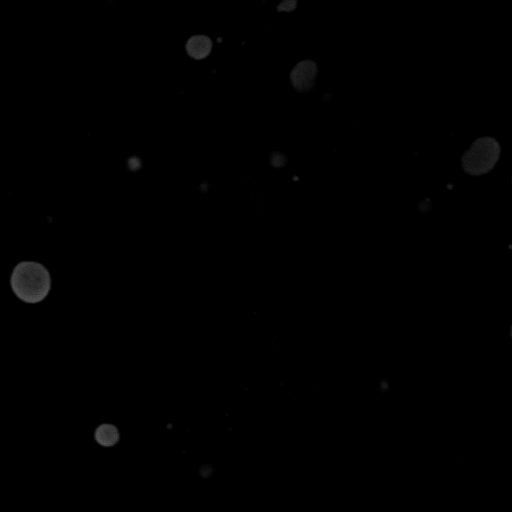

Supplement: Supplementary file 10 — Source data Fig. 1 [file 44318_2026_785_MOESM10_ESM.zip › Source data Fig. 1/1E/1E_NBR1KD_TBK1(pS172)-p62_p62.tif]

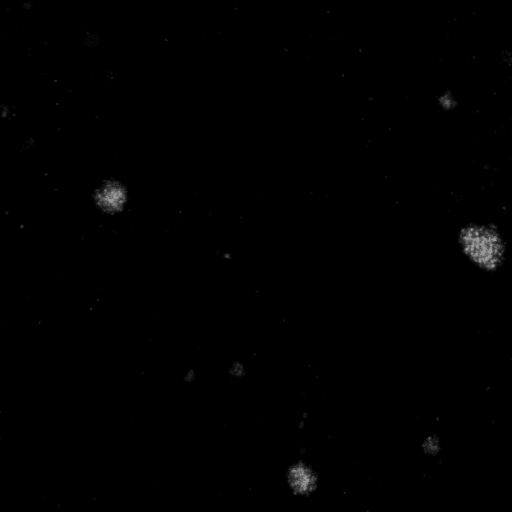

Supplement: Supplementary file 10 — Source data Fig. 1 [file 44318_2026_785_MOESM10_ESM.zip › Source data Fig. 1/1E/1E_NT_TBK1(pS172)-p62_TBK1(pS172).tif]

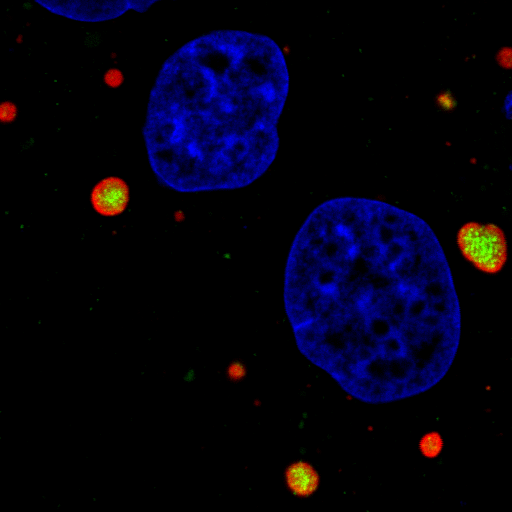

Supplement: Supplementary file 10 — Source data Fig. 1 [file 44318_2026_785_MOESM10_ESM.zip › Source data Fig. 1/1E/1E_NT_TBK1(pS172)-p62_Merge.tif]

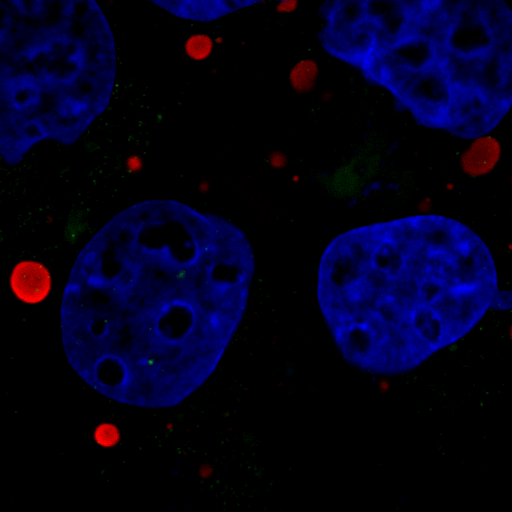

Supplement: Supplementary file 10 — Source data Fig. 1 [file 44318_2026_785_MOESM10_ESM.zip › Source data Fig. 1/1E/1E_NBR1KD_TBK1(pS172)-p62_Merge.tif]

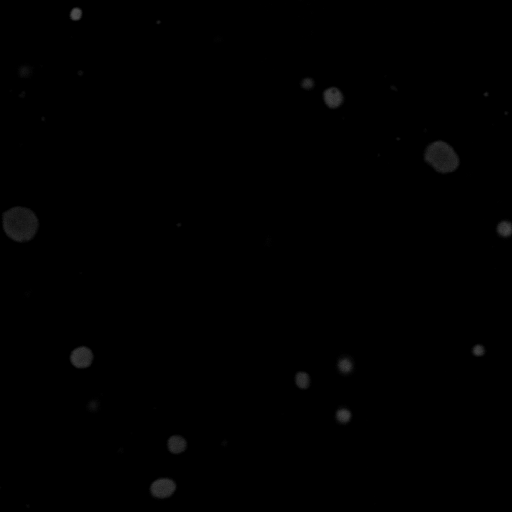

Supplement: Supplementary file 10 — Source data Fig. 1 [file 44318_2026_785_MOESM10_ESM.zip › Source data Fig. 1/1E/1E_TAX1BP1KD_TBK1(pS172)-p62_p62.tif]

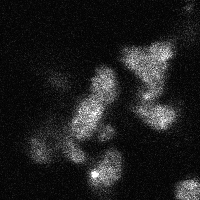

Supplement: Supplementary file 10 — Source data Fig. 1 [file 44318_2026_785_MOESM10_ESM.zip › Source data Fig. 1/1D/Image-7.tif]

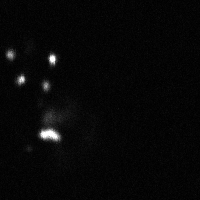

Supplement: Supplementary file 10 — Source data Fig. 1 [file 44318_2026_785_MOESM10_ESM.zip › Source data Fig. 1/1D/Image-6.tif]

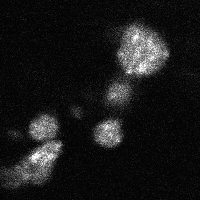

Supplement: Supplementary file 10 — Source data Fig. 1 [file 44318_2026_785_MOESM10_ESM.zip › Source data Fig. 1/1D/Image-4.tif]

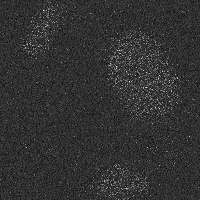

Supplement: Supplementary file 10 — Source data Fig. 1 [file 44318_2026_785_MOESM10_ESM.zip › Source data Fig. 1/1D/Image-5.tif]

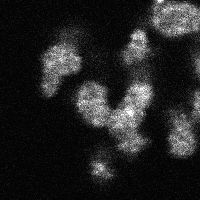

Supplement: Supplementary file 10 — Source data Fig. 1 [file 44318_2026_785_MOESM10_ESM.zip › Source data Fig. 1/1D/Image-1.tif]

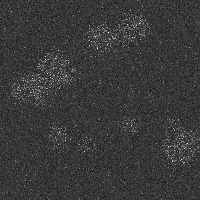

Supplement: Supplementary file 10 — Source data Fig. 1 [file 44318_2026_785_MOESM10_ESM.zip › Source data Fig. 1/1D/Image-2.tif]

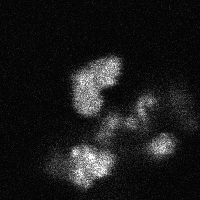

Supplement: Supplementary file 10 — Source data Fig. 1 [file 44318_2026_785_MOESM10_ESM.zip › Source data Fig. 1/1D/Image-3.tif]

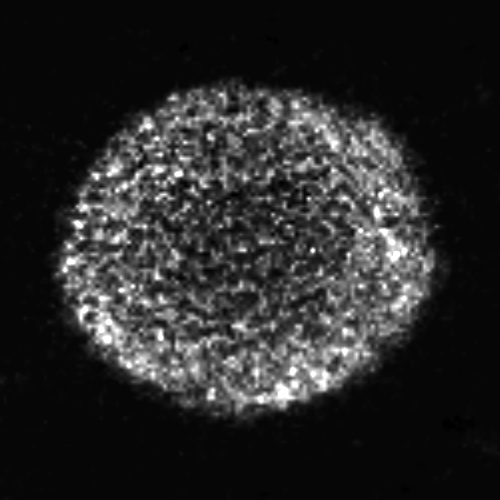

Supplement: Supplementary file 10 — Source data Fig. 1 [file 44318_2026_785_MOESM10_ESM.zip › Source data Fig. 1/1C/1C_p62-TAX1BP1_p62.tif]

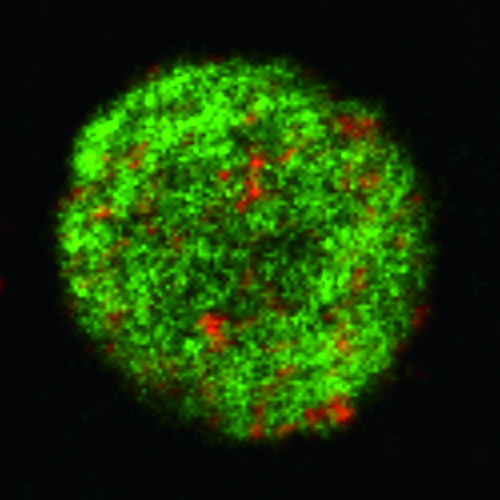

Supplement: Supplementary file 10 — Source data Fig. 1 [file 44318_2026_785_MOESM10_ESM.zip › Source data Fig. 1/1C/1C_p62-NBR1_Merge.tif]

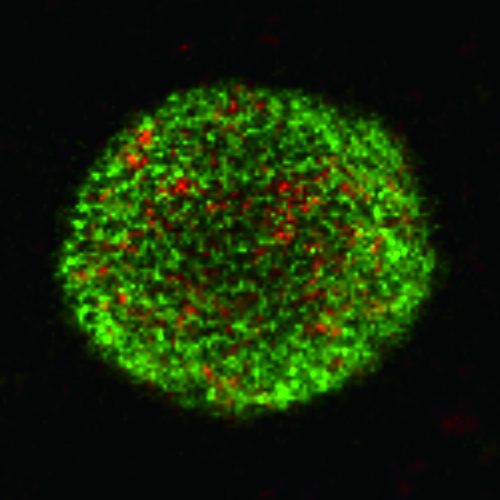

Supplement: Supplementary file 10 — Source data Fig. 1 [file 44318_2026_785_MOESM10_ESM.zip › Source data Fig. 1/1C/1C_p62-TAX1BP1_Merge.tif]

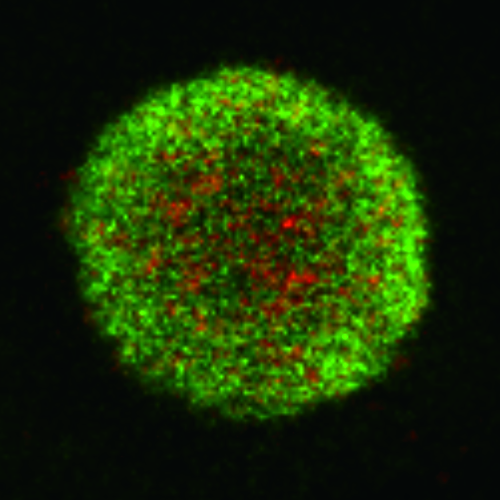

Supplement: Supplementary file 10 — Source data Fig. 1 [file 44318_2026_785_MOESM10_ESM.zip › Source data Fig. 1/1C/1C_p62-pTBK1_Merge.tif]

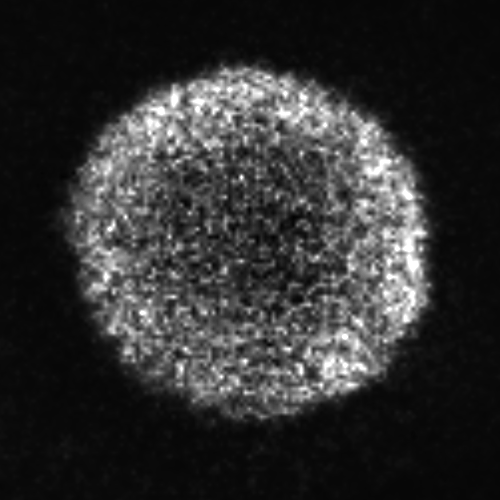

Supplement: Supplementary file 10 — Source data Fig. 1 [file 44318_2026_785_MOESM10_ESM.zip › Source data Fig. 1/1C/1C_p62-pTBK1_p62.tif]

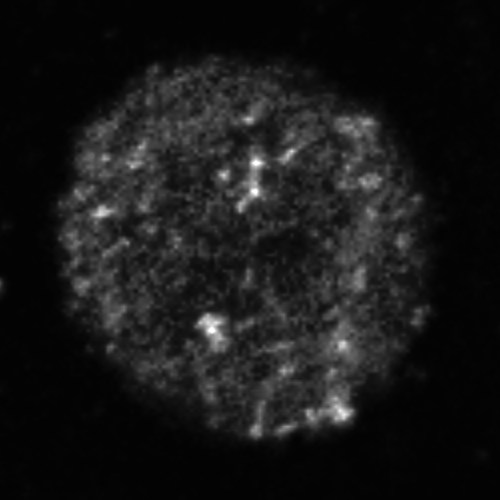

Supplement: Supplementary file 10 — Source data Fig. 1 [file 44318_2026_785_MOESM10_ESM.zip › Source data Fig. 1/1C/1C_p62-NBR1_NBR1.tif]

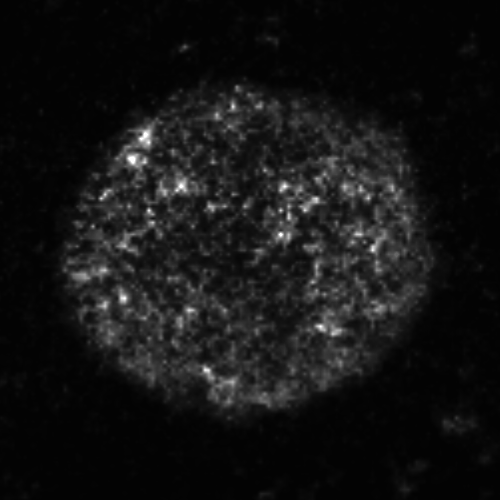

Supplement: Supplementary file 10 — Source data Fig. 1 [file 44318_2026_785_MOESM10_ESM.zip › Source data Fig. 1/1C/1C_p62-TAX1BP1_TAX1BP1.tif]

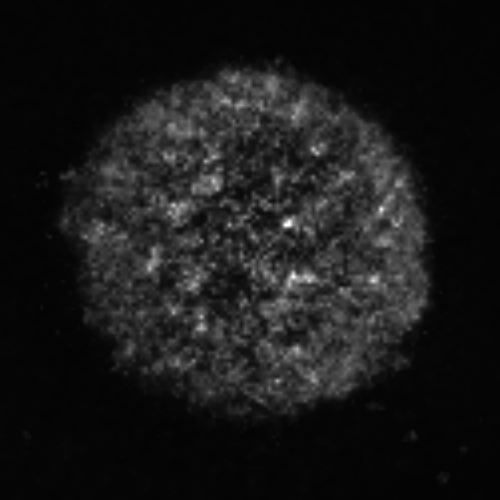

Supplement: Supplementary file 10 — Source data Fig. 1 [file 44318_2026_785_MOESM10_ESM.zip › Source data Fig. 1/1C/1C_p62-pTBK1_TBK1pS1172.tif]

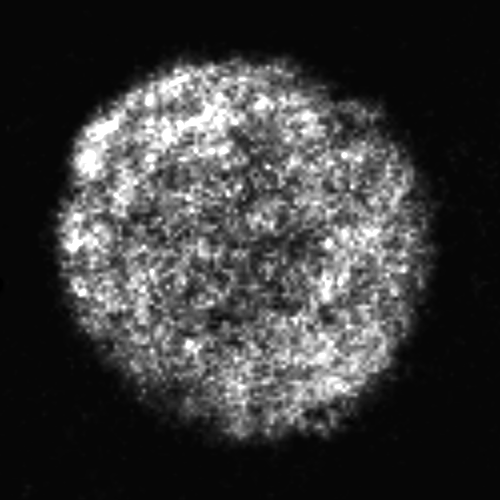

Supplement: Supplementary file 10 — Source data Fig. 1 [file 44318_2026_785_MOESM10_ESM.zip › Source data Fig. 1/1C/1C_p62-NBR1_p62.tif]

Figure 2A

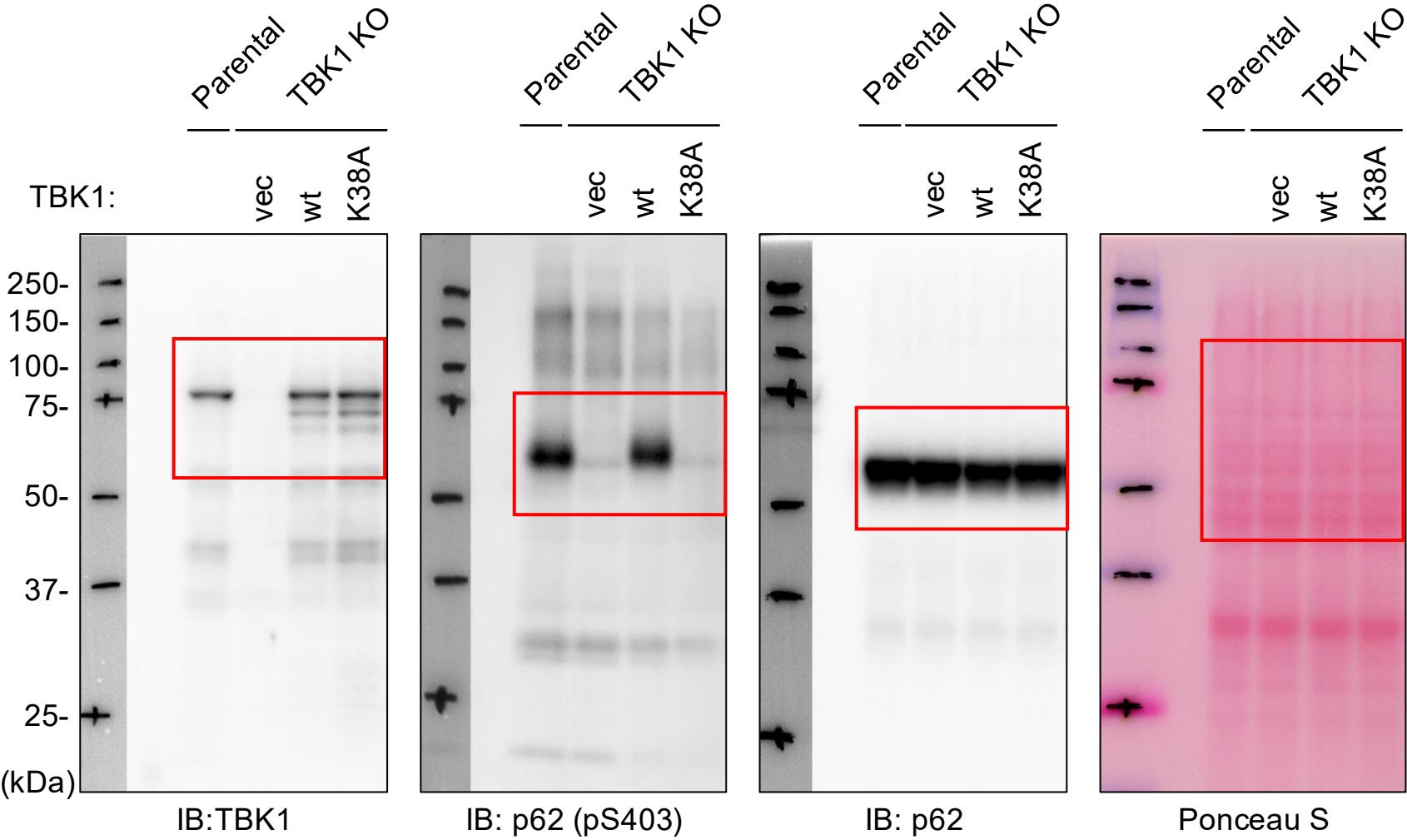

Supplement: Supplementary file 11 — Source data Fig. 2 [file 44318_2026_785_MOESM11_ESM.zip › Source data Fig. 2/2A/2A.pdf]

Figure 2C

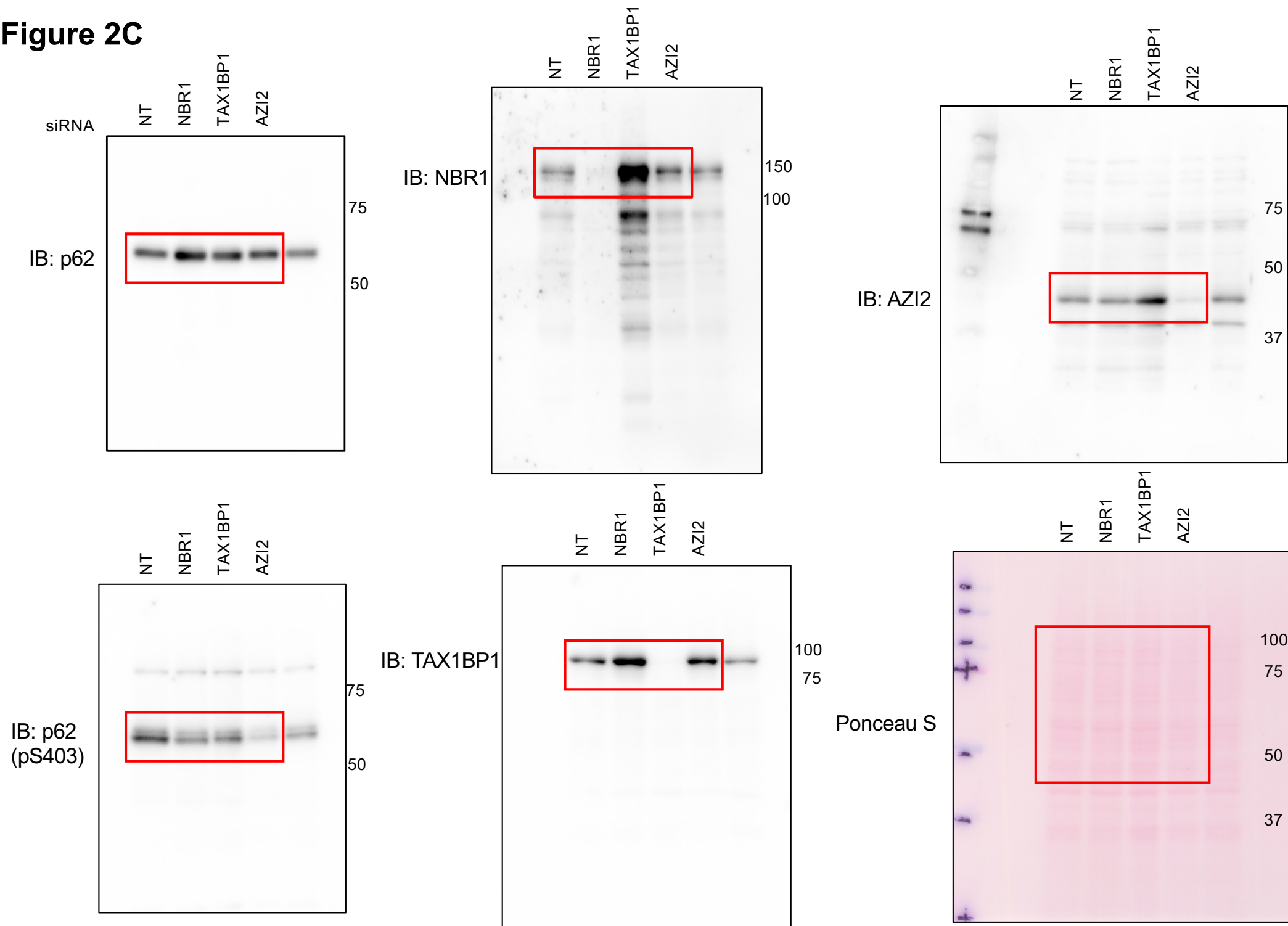

Supplement: Supplementary file 11 — Source data Fig. 2 [file 44318_2026_785_MOESM11_ESM.zip › Source data Fig. 2/2C/2C.pdf]

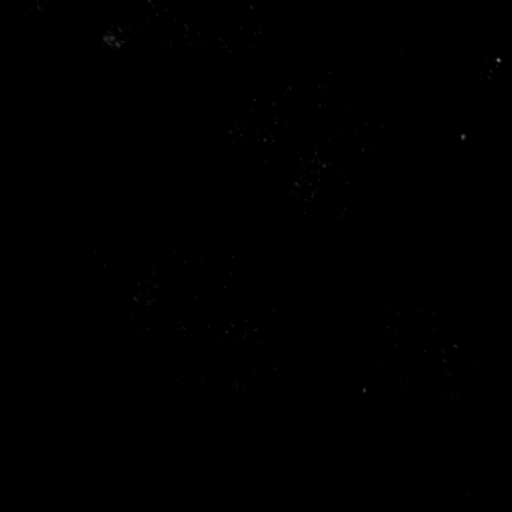

Supplement: Supplementary file 11 — Source data Fig. 2 [file 44318_2026_785_MOESM11_ESM.zip › Source data Fig. 2/2D/2D_TAX1BP1KD_p62(pS403)-p62_p62(pS403).tif]

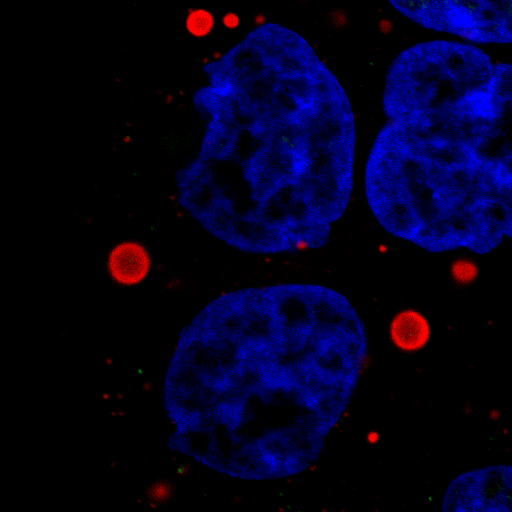

Supplement: Supplementary file 11 — Source data Fig. 2 [file 44318_2026_785_MOESM11_ESM.zip › Source data Fig. 2/2D/2D_NBR1KD_p62(pS403)-p62_Merge.tif]

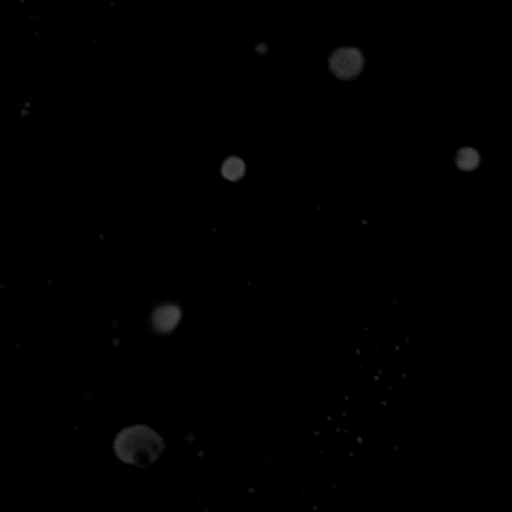

Supplement: Supplementary file 11 — Source data Fig. 2 [file 44318_2026_785_MOESM11_ESM.zip › Source data Fig. 2/2D/2D_NT_p62(pS403)-p62_p62.tif]

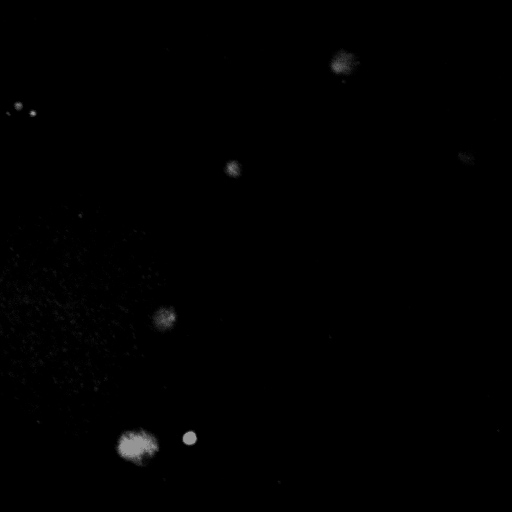

Supplement: Supplementary file 11 — Source data Fig. 2 [file 44318_2026_785_MOESM11_ESM.zip › Source data Fig. 2/2D/2D_NT_p62(pS403)-p62_p62(pS403).tif]

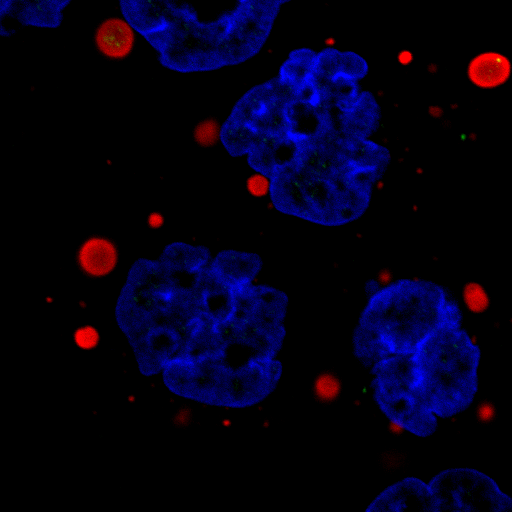

Supplement: Supplementary file 11 — Source data Fig. 2 [file 44318_2026_785_MOESM11_ESM.zip › Source data Fig. 2/2D/2D_TAX1BP1KD_p62(pS403)-p62_Merge.tif]

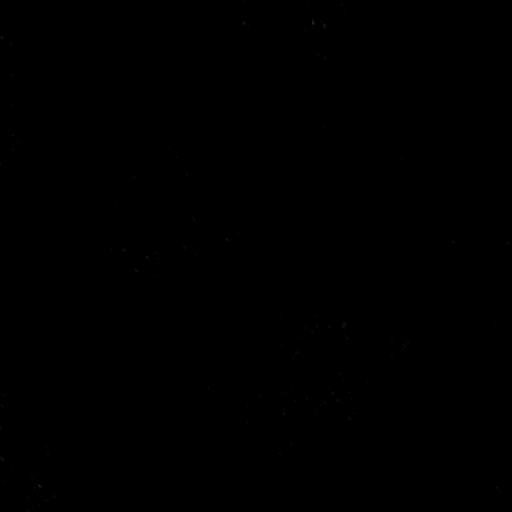

Supplement: Supplementary file 11 — Source data Fig. 2 [file 44318_2026_785_MOESM11_ESM.zip › Source data Fig. 2/2D/2D_AZI2KD_p62(pS403)-p62_p62(pS403).tif]

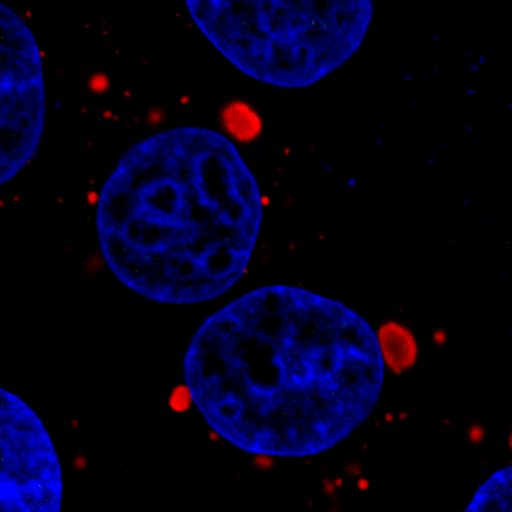

Supplement: Supplementary file 11 — Source data Fig. 2 [file 44318_2026_785_MOESM11_ESM.zip › Source data Fig. 2/2D/2D_AZI2KD_p62(pS403)-p62_Merge.tif]

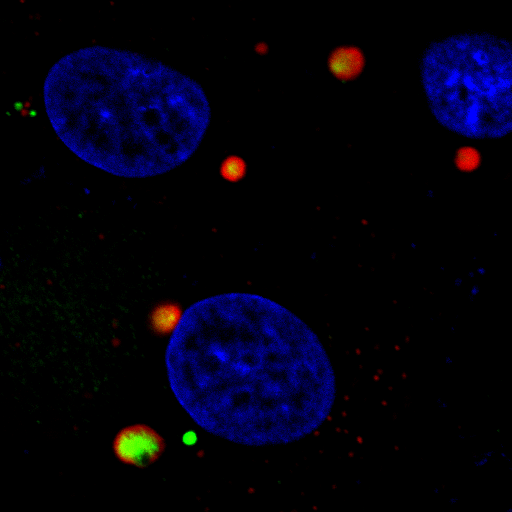

Supplement: Supplementary file 11 — Source data Fig. 2 [file 44318_2026_785_MOESM11_ESM.zip › Source data Fig. 2/2D/2D_NT_p62(pS403)-p62_Merge.tif]

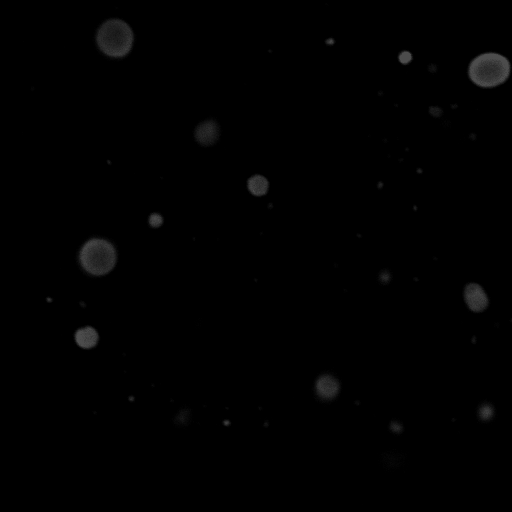

Supplement: Supplementary file 11 — Source data Fig. 2 [file 44318_2026_785_MOESM11_ESM.zip › Source data Fig. 2/2D/2D_TAX1BP1KD_p62(pS403)-p62_p62.tif]

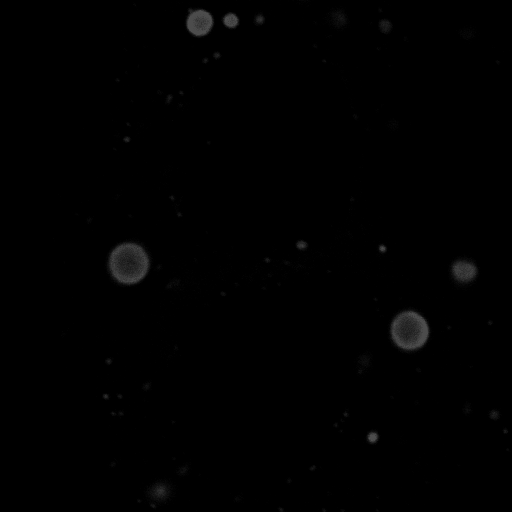

Supplement: Supplementary file 11 — Source data Fig. 2 [file 44318_2026_785_MOESM11_ESM.zip › Source data Fig. 2/2D/2D_NBR1KD_p62(pS403)-p62_p62.tif]

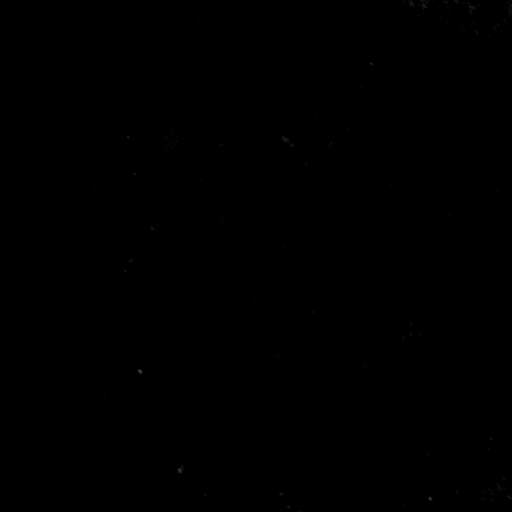

Supplement: Supplementary file 11 — Source data Fig. 2 [file 44318_2026_785_MOESM11_ESM.zip › Source data Fig. 2/2D/2D_NBR1KD_p62(pS403)-p62_p62(pS403).tif]

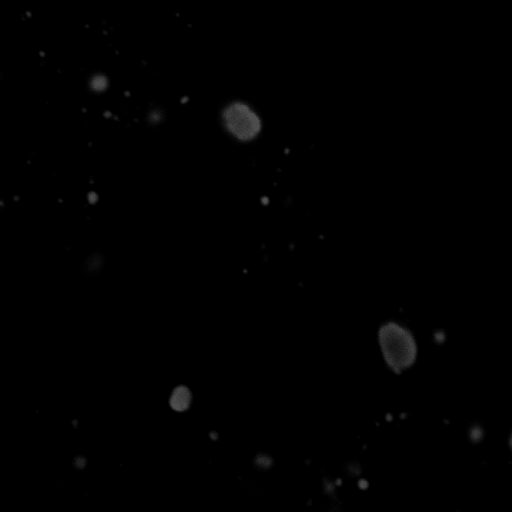

Supplement: Supplementary file 11 — Source data Fig. 2 [file 44318_2026_785_MOESM11_ESM.zip › Source data Fig. 2/2D/2D_AZI2KD_p62(pS403)-p62_p62.tif]

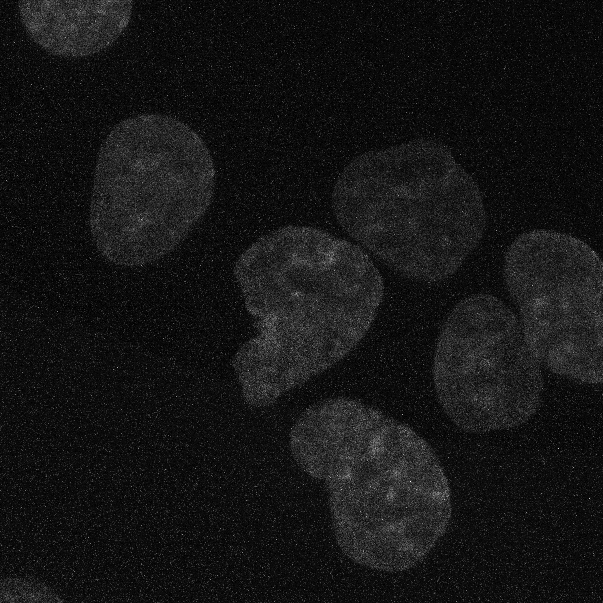

Supplement: Supplementary file 11 — Source data Fig. 2 [file 44318_2026_785_MOESM11_ESM.zip › Source data Fig. 2/2B/TBK1 KO_vector.tif]

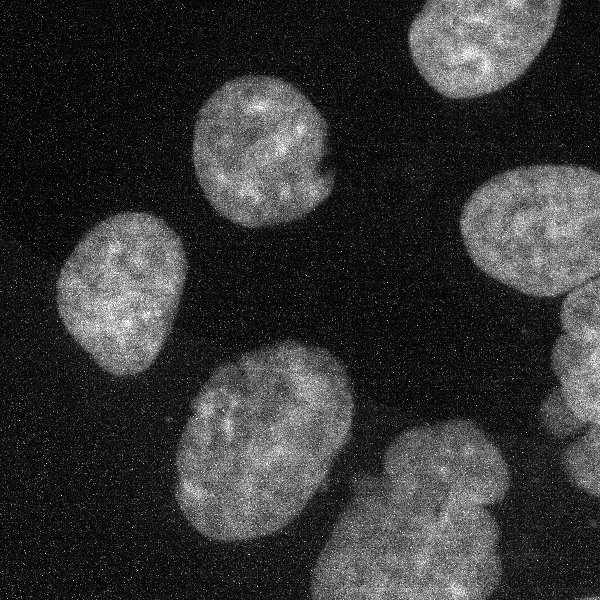

Supplement: Supplementary file 11 — Source data Fig. 2 [file 44318_2026_785_MOESM11_ESM.zip › Source data Fig. 2/2B/TBK1 KO_wt.tif]

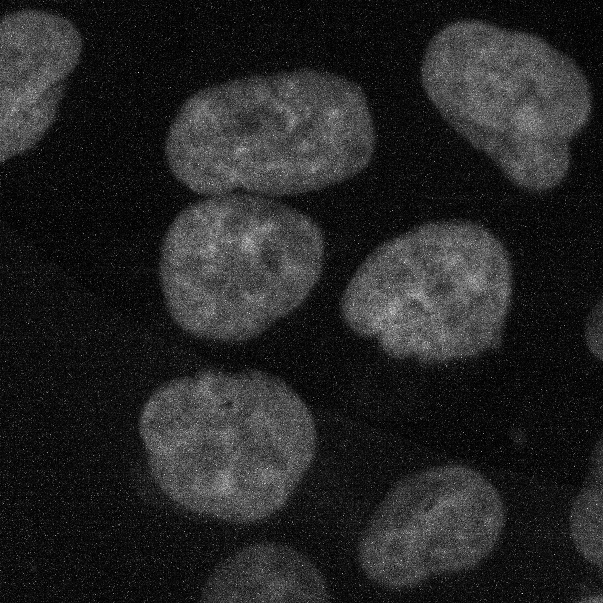

Supplement: Supplementary file 11 — Source data Fig. 2 [file 44318_2026_785_MOESM11_ESM.zip › Source data Fig. 2/2B/TBK1 KO_K38A.tif]

### Figure 3E

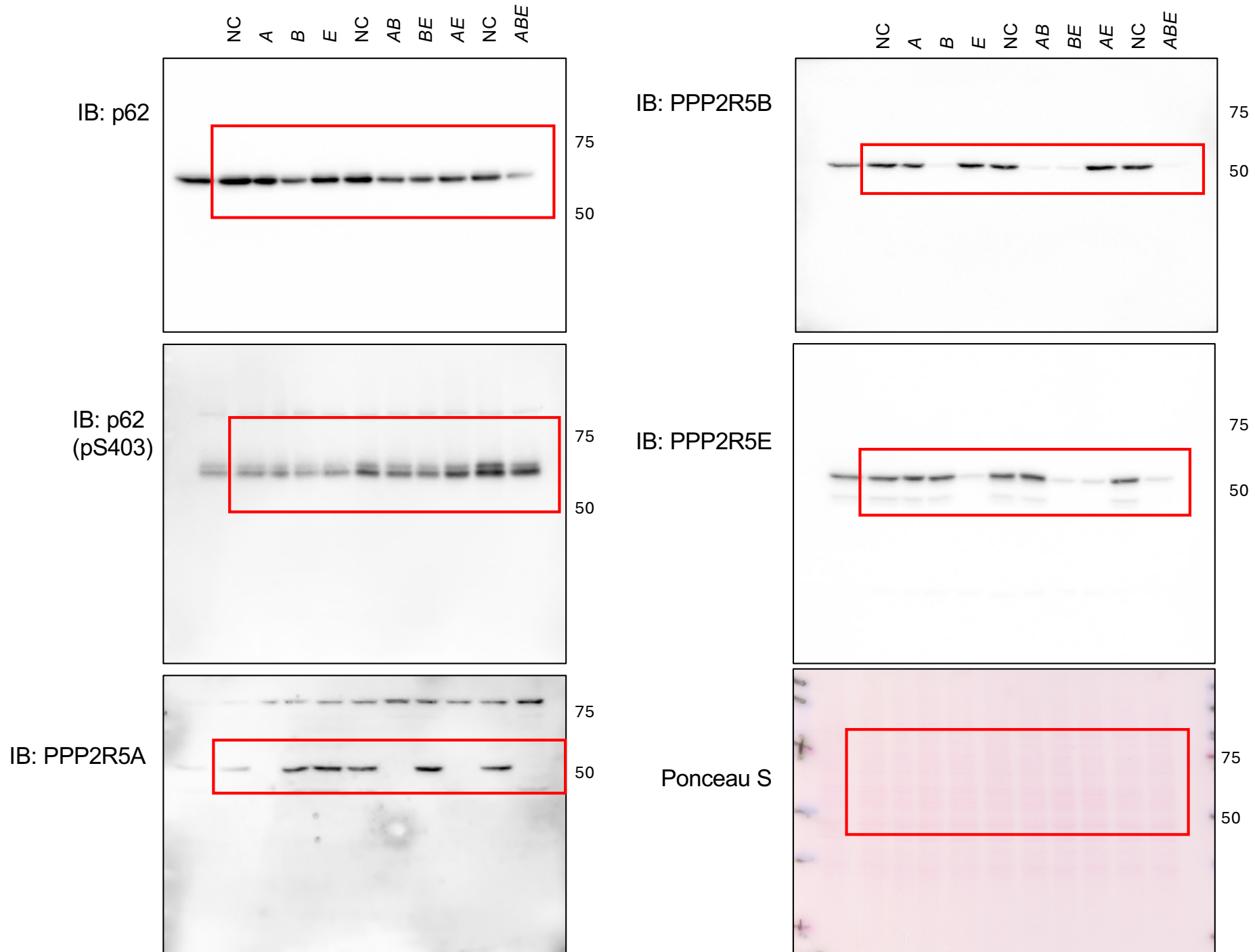

Supplement: Supplementary file 12 — Source data Fig. 3 [file 44318_2026_785_MOESM12_ESM.zip › Source data Fig. 3/3E/3E.pdf]

Figure 3B

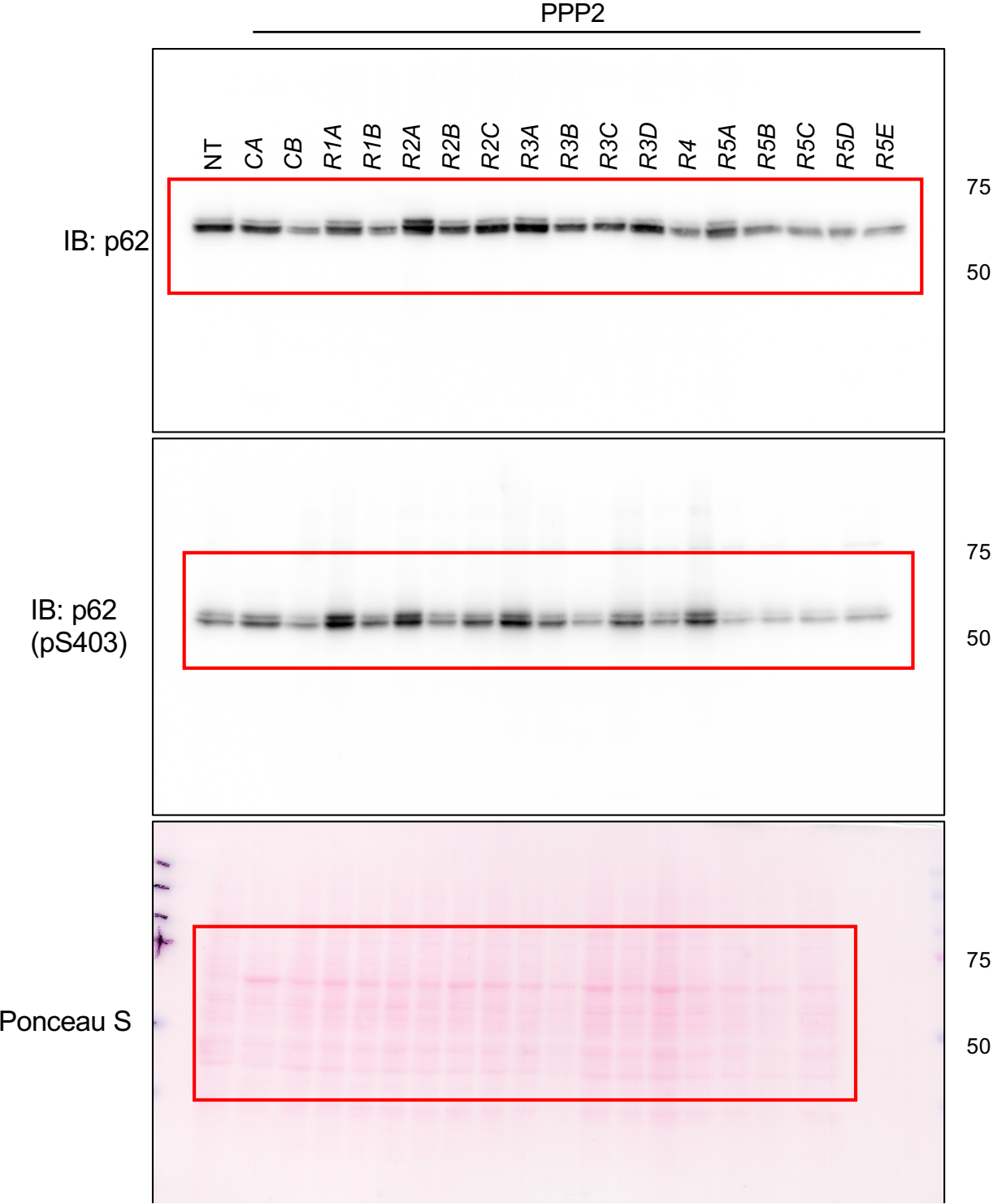

Supplement: Supplementary file 12 — Source data Fig. 3 [file 44318_2026_785_MOESM12_ESM.zip › Source data Fig. 3/3B/3B.pdf]

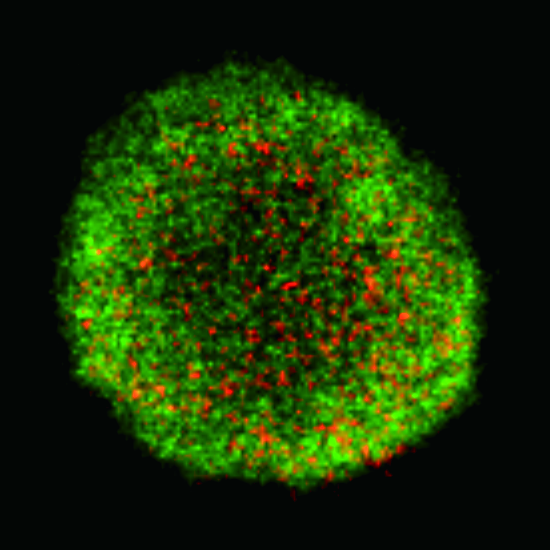

Supplement: Supplementary file 12 — Source data Fig. 3 [file 44318_2026_785_MOESM12_ESM.zip › Source data Fig. 3/3H/3H_p62-PPP2R5E_Merge.tif]

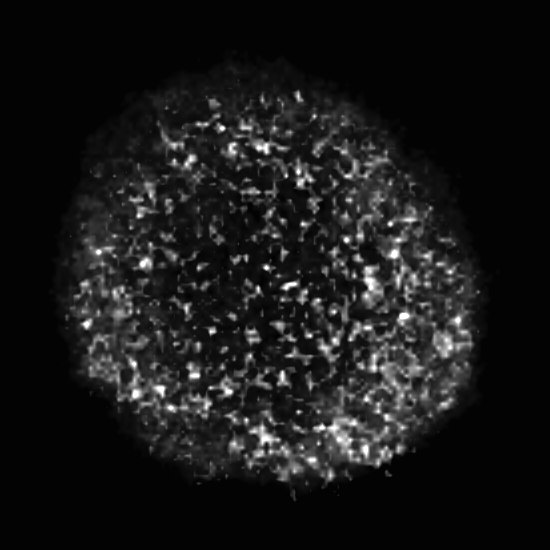

Supplement: Supplementary file 12 — Source data Fig. 3 [file 44318_2026_785_MOESM12_ESM.zip › Source data Fig. 3/3H/3H_p62-PPP2R5E_PPP2R5E.tif]

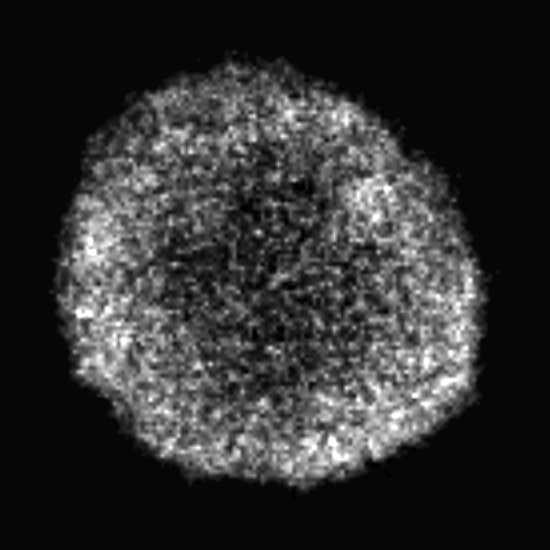

Supplement: Supplementary file 12 — Source data Fig. 3 [file 44318_2026_785_MOESM12_ESM.zip › Source data Fig. 3/3H/3H_p62-PPP2R5E_p62.tif]

Figure 3A

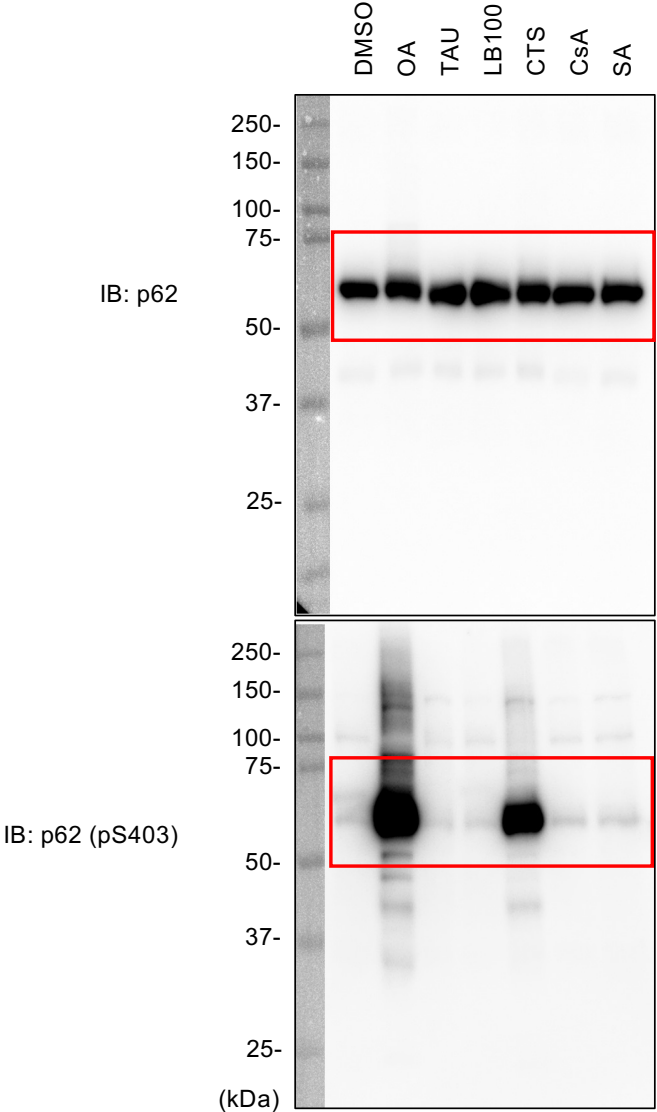

Supplement: Supplementary file 12 — Source data Fig. 3 [file 44318_2026_785_MOESM12_ESM.zip › Source data Fig. 3/3A/3A.pdf]

Figure 3F

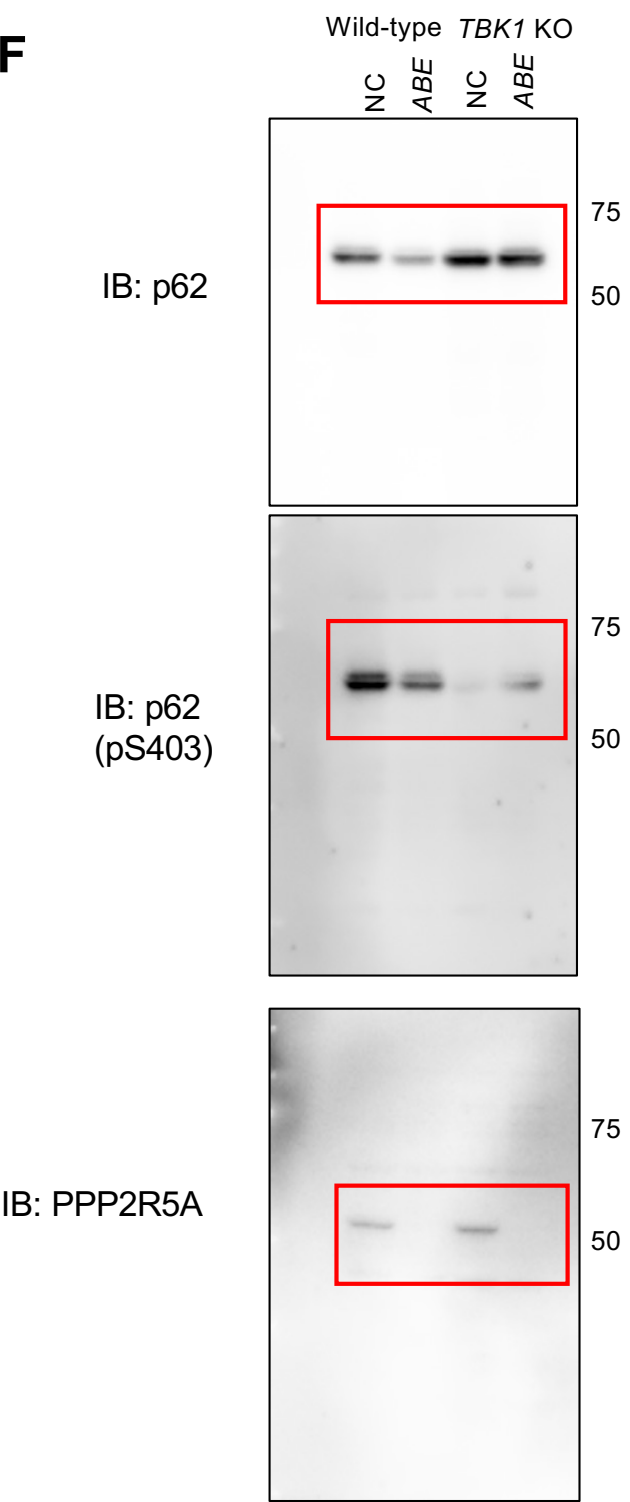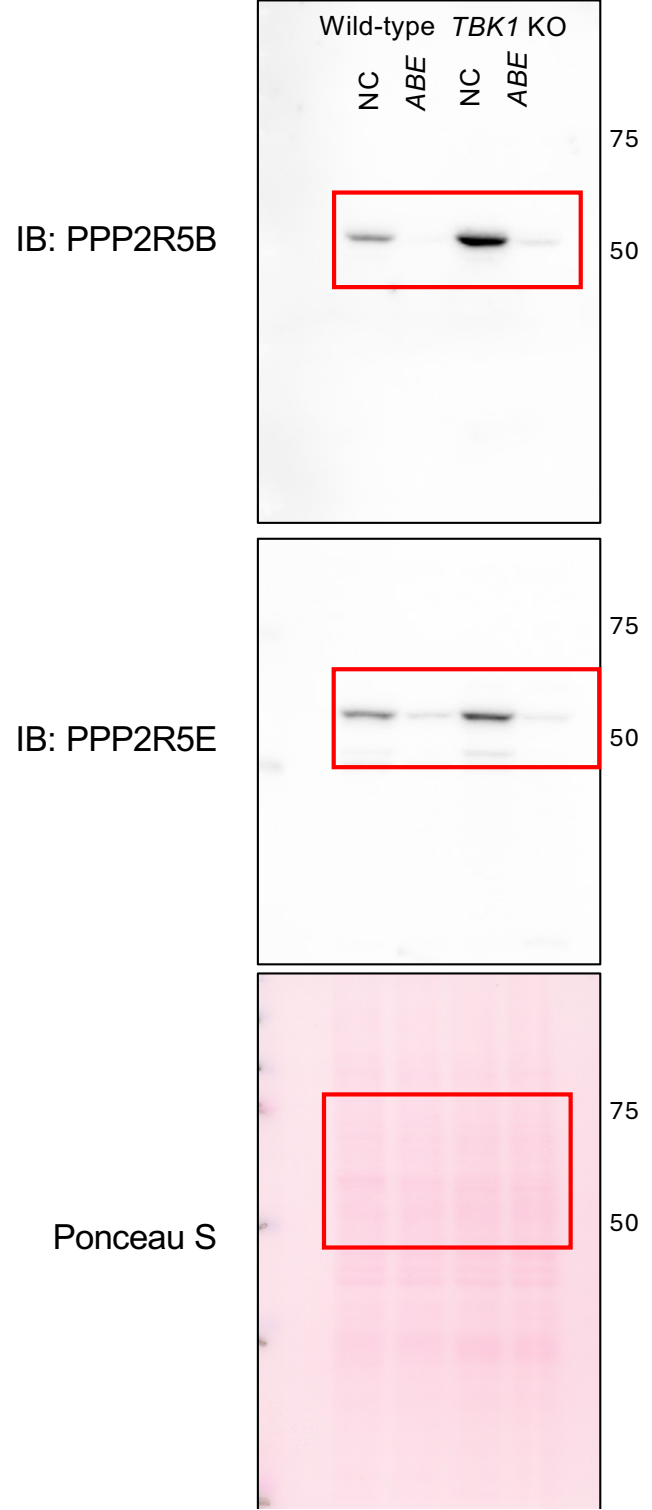

Supplement: Supplementary file 12 — Source data Fig. 3 [file 44318_2026_785_MOESM12_ESM.zip › Source data Fig. 3/3F/3F.pdf]

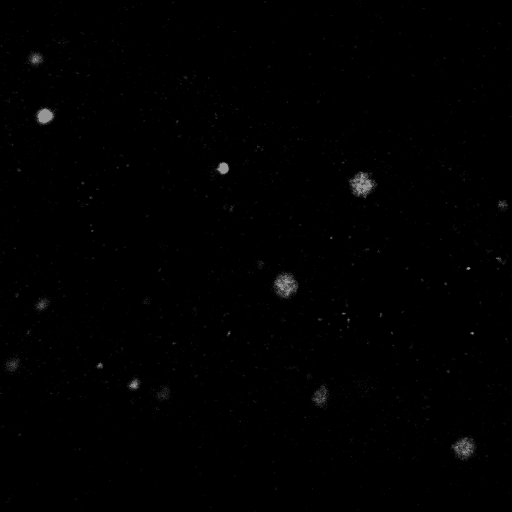

Supplement: Supplementary file 12 — Source data Fig. 3 [file 44318_2026_785_MOESM12_ESM.zip › Source data Fig. 3/3G/3G_PPP2R5E-p62_PPP2R5E.tif]

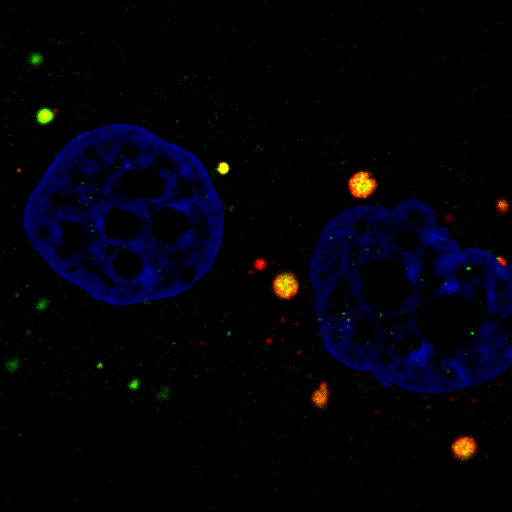

Supplement: Supplementary file 12 — Source data Fig. 3 [file 44318_2026_785_MOESM12_ESM.zip › Source data Fig. 3/3G/3G_PPP2R5E-p62_Merge.tif]

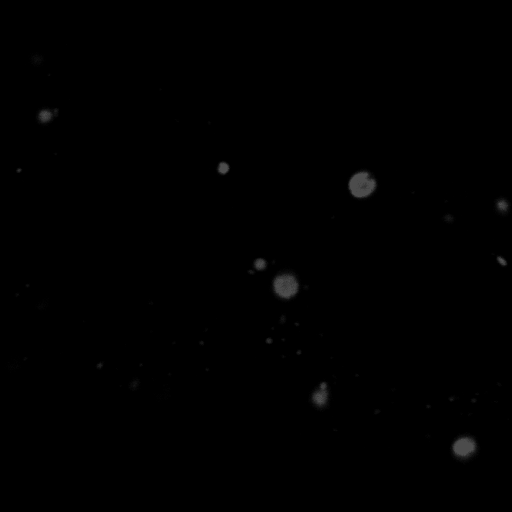

Supplement: Supplementary file 12 — Source data Fig. 3 [file 44318_2026_785_MOESM12_ESM.zip › Source data Fig. 3/3G/3G_PPP2R5E-p62_p62.tif]

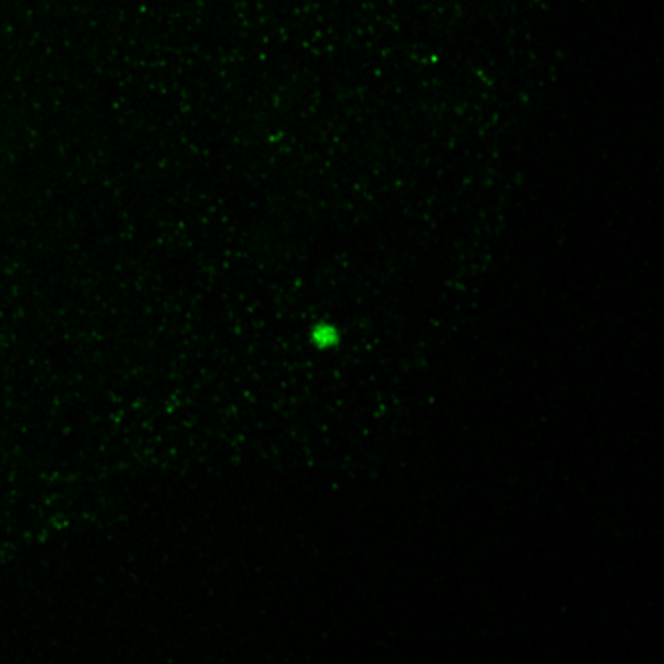

Supplement: Supplementary file 12 — Source data Fig. 3 [file 44318_2026_785_MOESM12_ESM.zip › Source data Fig. 3/3I/3I_c1_PPP2R5E.tif]

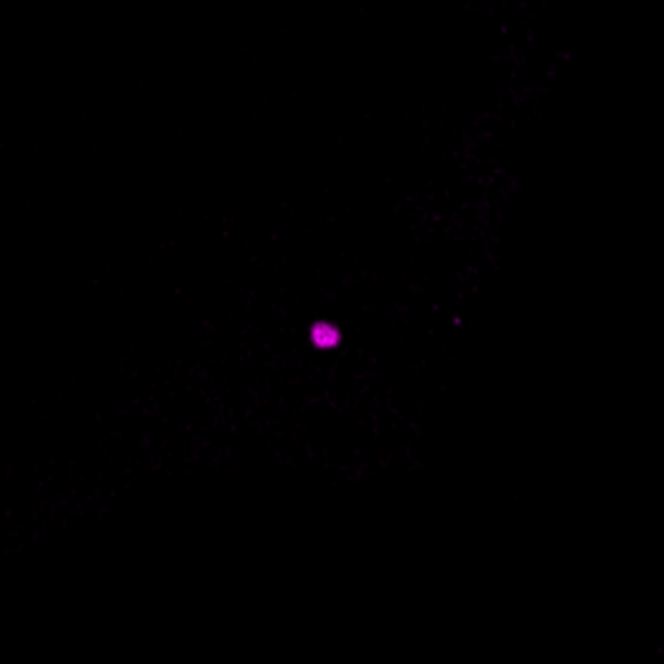

Supplement: Supplementary file 12 — Source data Fig. 3 [file 44318_2026_785_MOESM12_ESM.zip › Source data Fig. 3/3I/3I_c2_p62.tif]

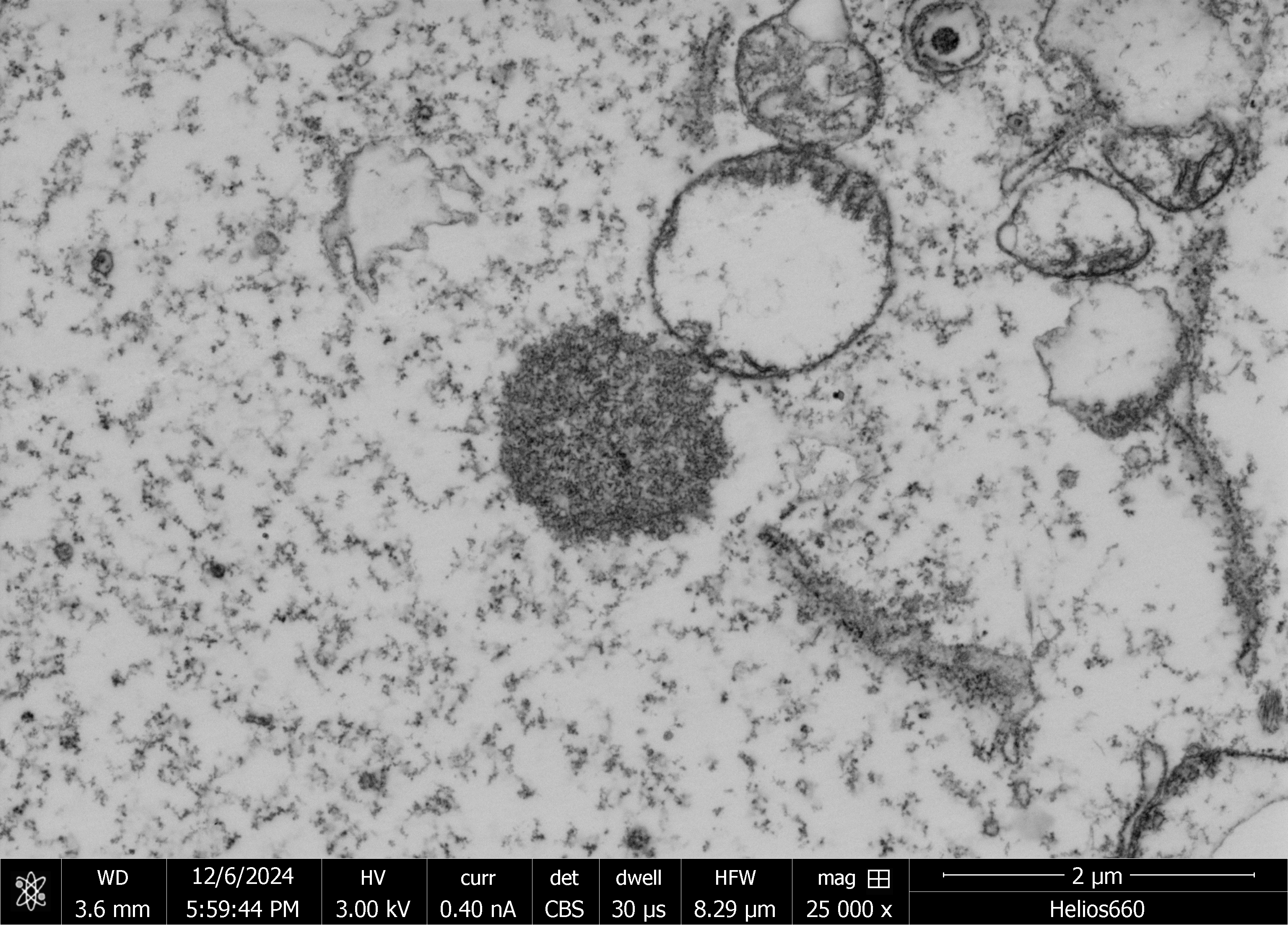

Supplement: Supplementary file 12 — Source data Fig. 3 [file 44318_2026_785_MOESM12_ESM.zip › Source data Fig. 3/3I/3I_EM_cropped.tif]

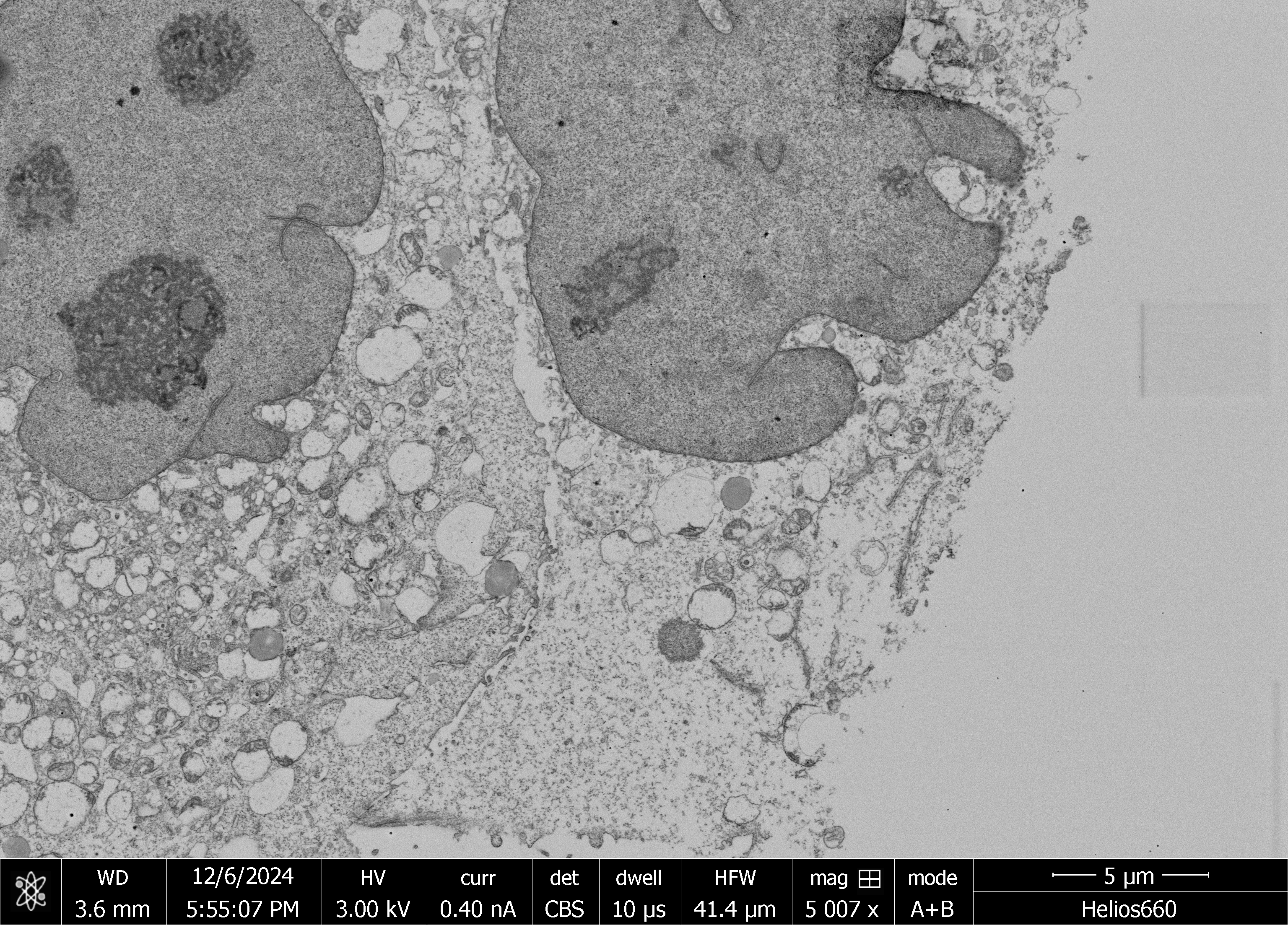

Supplement: Supplementary file 12 — Source data Fig. 3 [file 44318_2026_785_MOESM12_ESM.zip › Source data Fig. 3/3I/3I_EM_overview.tif]

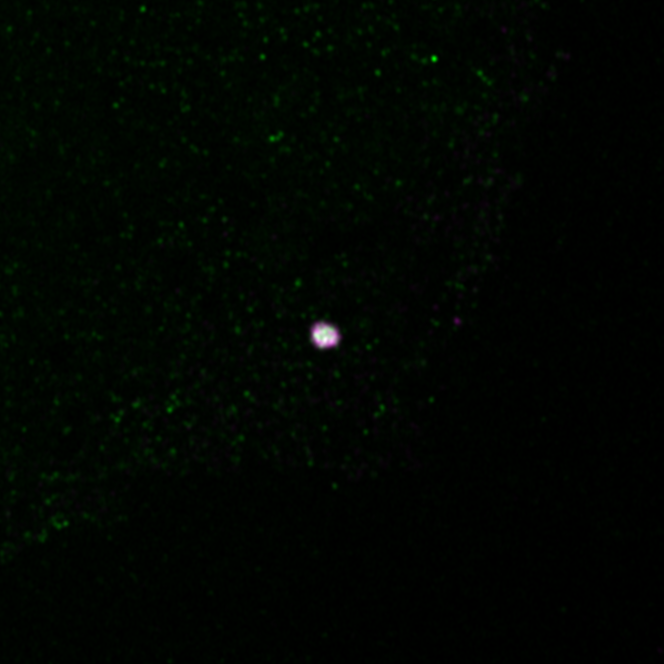

Supplement: Supplementary file 12 — Source data Fig. 3 [file 44318_2026_785_MOESM12_ESM.zip › Source data Fig. 3/3I/3I_merge.tif]

Figure 4F

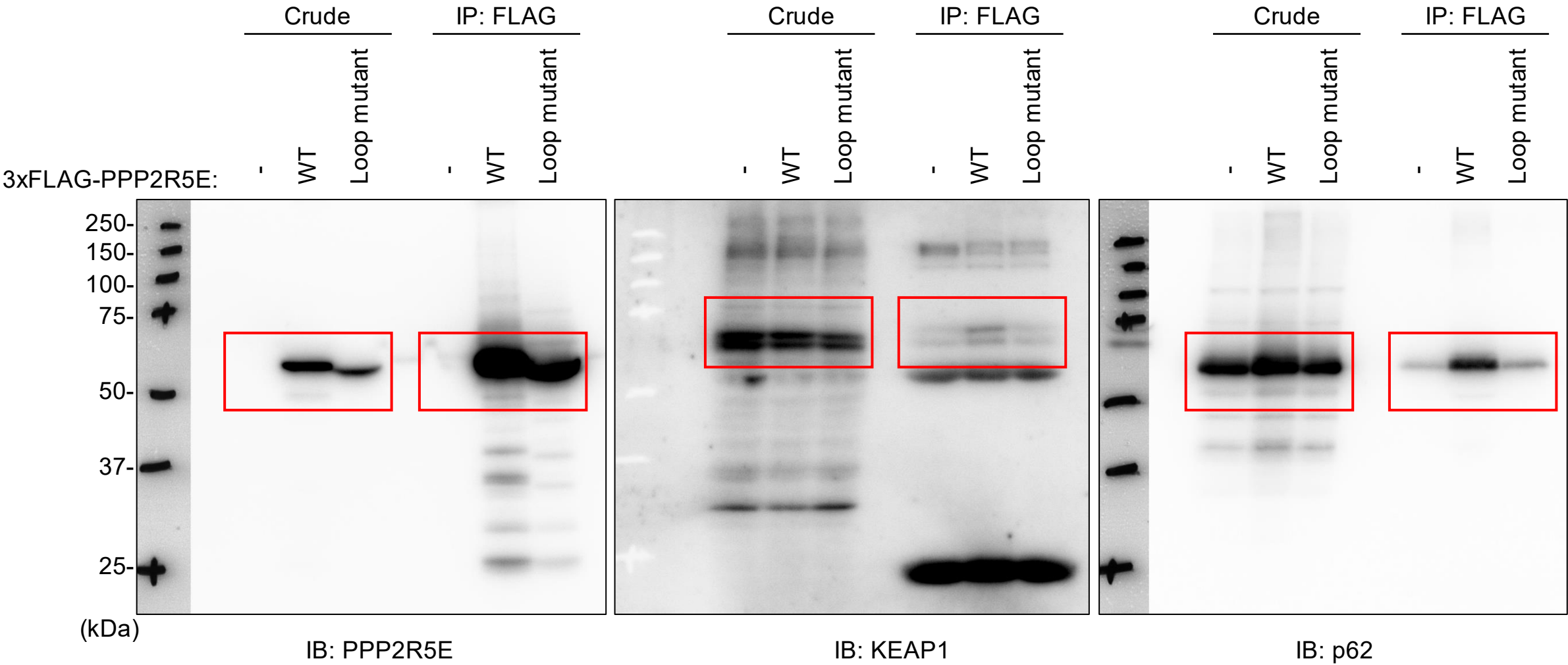

Supplement: Supplementary file 13 — Source data Fig. 4 [file 44318_2026_785_MOESM13_ESM.zip › Source data Fig. 4/4F/4F.pdf]

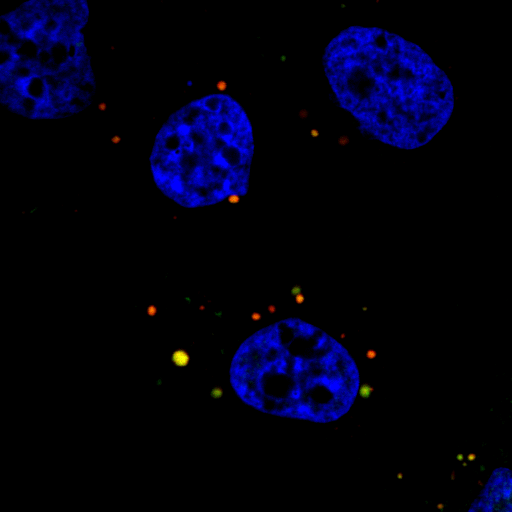

Supplement: Supplementary file 13 — Source data Fig. 4 [file 44318_2026_785_MOESM13_ESM.zip › Source data Fig. 4/4G/4G_PPP2R5ELoop mut_KEAP1-p62_Merge.tif]

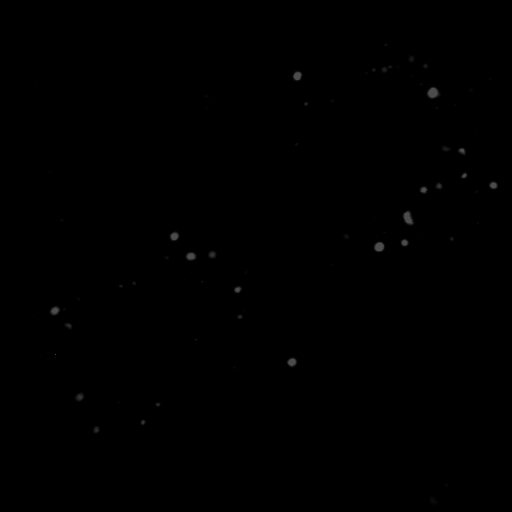

Supplement: Supplementary file 13 — Source data Fig. 4 [file 44318_2026_785_MOESM13_ESM.zip › Source data Fig. 4/4G/4G_-_KEAP1-p62_p62.tif]

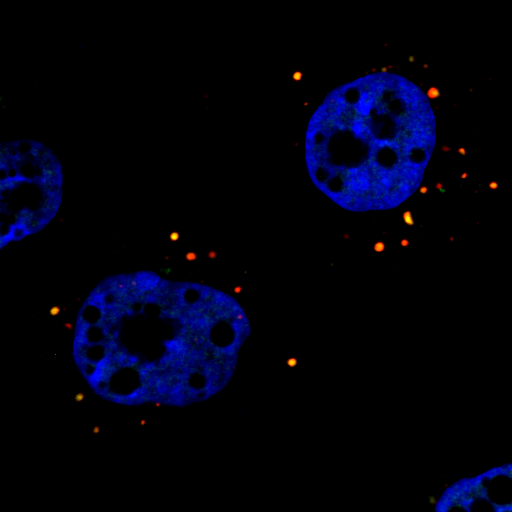

Supplement: Supplementary file 13 — Source data Fig. 4 [file 44318_2026_785_MOESM13_ESM.zip › Source data Fig. 4/4G/4G_-_KEAP1-p62_Merge.tif]

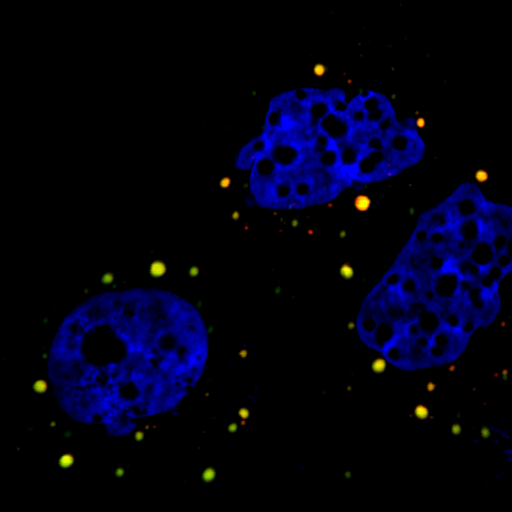

Supplement: Supplementary file 13 — Source data Fig. 4 [file 44318_2026_785_MOESM13_ESM.zip › Source data Fig. 4/4G/4G_PPP2R5E_PPP2R5E-p62_Merge.tif]

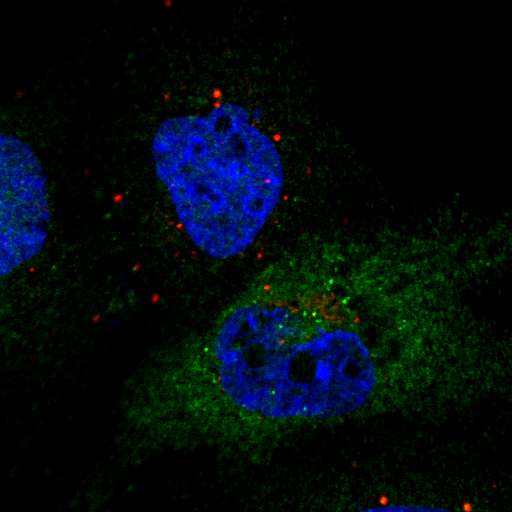

Supplement: Supplementary file 13 — Source data Fig. 4 [file 44318_2026_785_MOESM13_ESM.zip › Source data Fig. 4/4G/4G_PPP2R5ELoop mut_PPP2R5E-p62_Merge.tif]

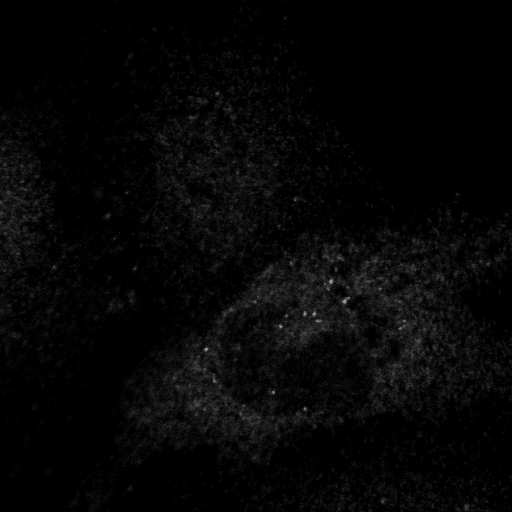

Supplement: Supplementary file 13 — Source data Fig. 4 [file 44318_2026_785_MOESM13_ESM.zip › Source data Fig. 4/4G/4G_PPP2R5ELoop mut_PPP2R5E-p62_PPP2R5E.tif]

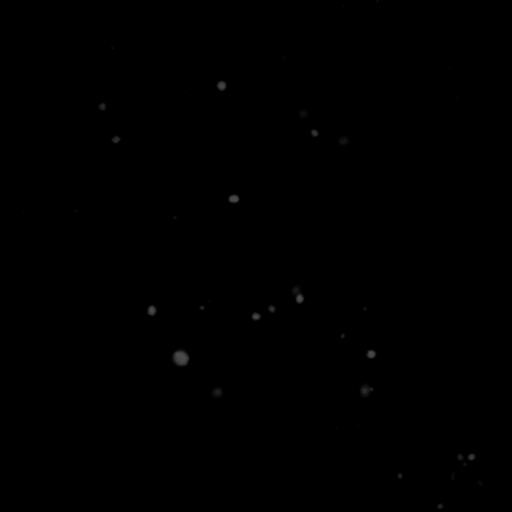

Supplement: Supplementary file 13 — Source data Fig. 4 [file 44318_2026_785_MOESM13_ESM.zip › Source data Fig. 4/4G/4G_PPP2R5ELoop mut_KEAP1-p62_p62.tif]

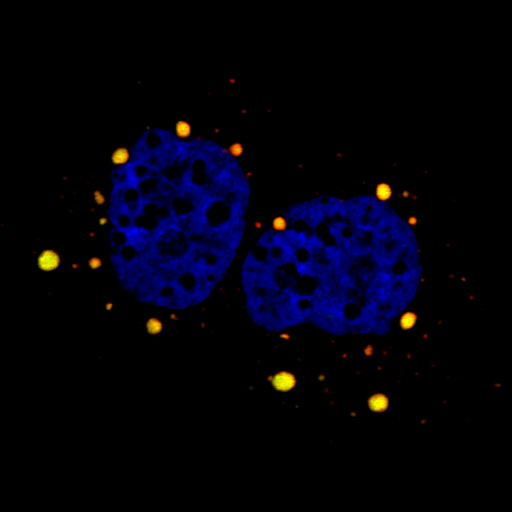

Supplement: Supplementary file 13 — Source data Fig. 4 [file 44318_2026_785_MOESM13_ESM.zip › Source data Fig. 4/4G/4G_PPP2R5E_KEAP1-p62_Merge.tif]

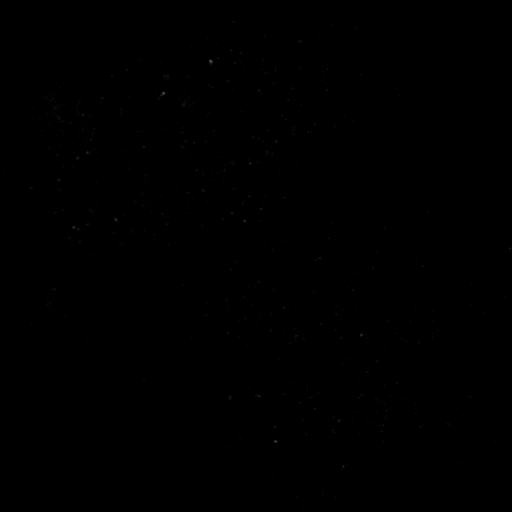

Supplement: Supplementary file 13 — Source data Fig. 4 [file 44318_2026_785_MOESM13_ESM.zip › Source data Fig. 4/4G/4G_-_PPP2R5E-p62_PPP2R5E.tif]

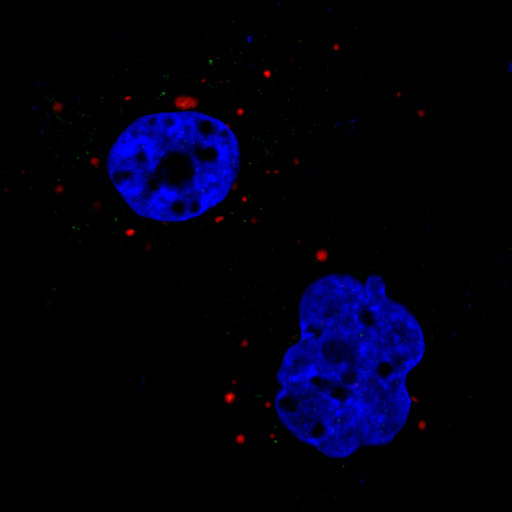

Supplement: Supplementary file 13 — Source data Fig. 4 [file 44318_2026_785_MOESM13_ESM.zip › Source data Fig. 4/4G/4G_-_PPP2R5E-p62_Merge.tif]

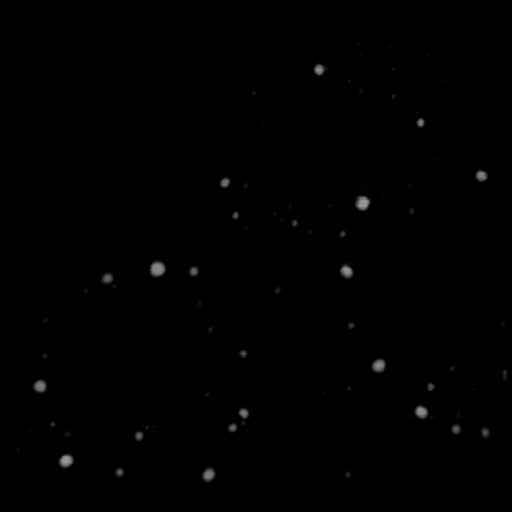

Supplement: Supplementary file 13 — Source data Fig. 4 [file 44318_2026_785_MOESM13_ESM.zip › Source data Fig. 4/4G/4G_PPP2R5E_PPP2R5E-p62_PPP2R5E.tif]

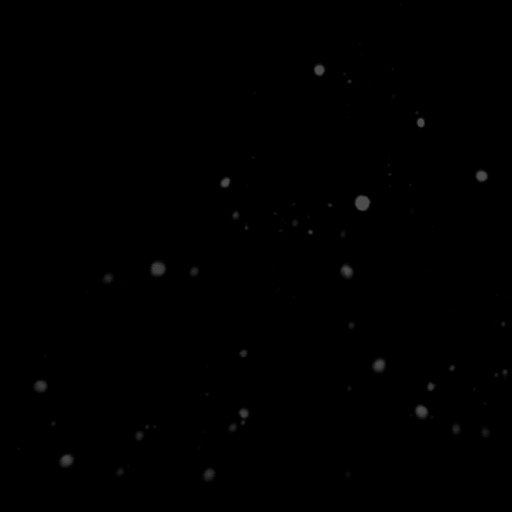

Supplement: Supplementary file 13 — Source data Fig. 4 [file 44318_2026_785_MOESM13_ESM.zip › Source data Fig. 4/4G/4G_PPP2R5E_PPP2R5E-p62_p62.tif]

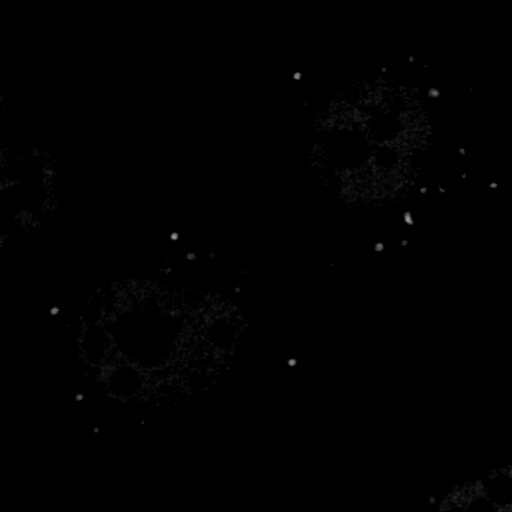

Supplement: Supplementary file 13 — Source data Fig. 4 [file 44318_2026_785_MOESM13_ESM.zip › Source data Fig. 4/4G/4G_-_KEAP1-p62_KEAP1.tif]

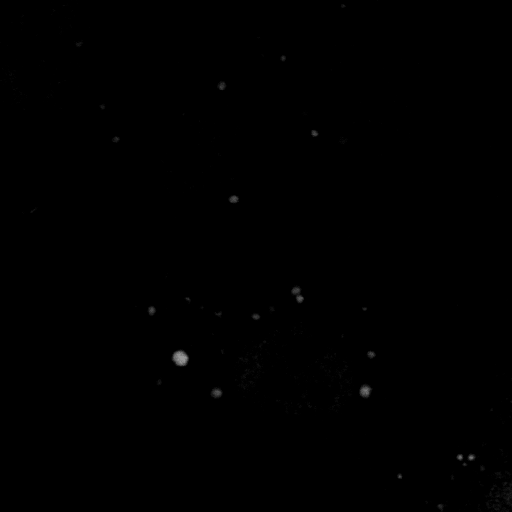

Supplement: Supplementary file 13 — Source data Fig. 4 [file 44318_2026_785_MOESM13_ESM.zip › Source data Fig. 4/4G/4G_PPP2R5ELoop mut_KEAP1-p62_KEAP1.tif]

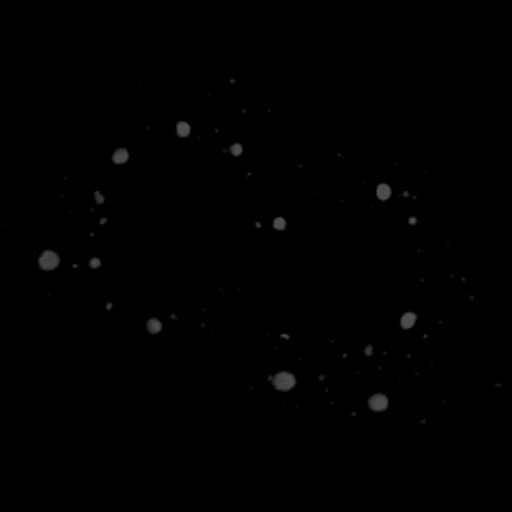

Supplement: Supplementary file 13 — Source data Fig. 4 [file 44318_2026_785_MOESM13_ESM.zip › Source data Fig. 4/4G/4G_PPP2R5E_KEAP1-p62_p62.tif]

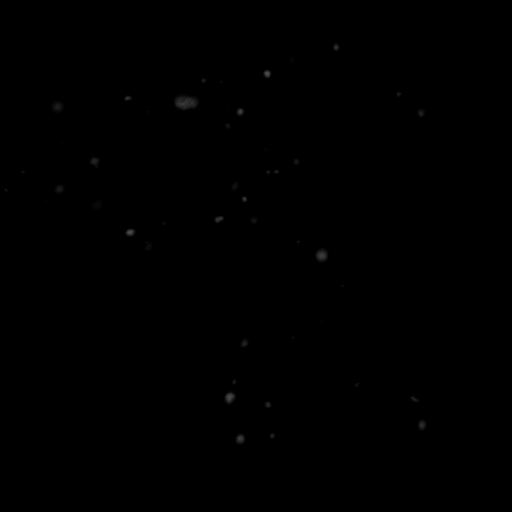

Supplement: Supplementary file 13 — Source data Fig. 4 [file 44318_2026_785_MOESM13_ESM.zip › Source data Fig. 4/4G/4G_-_PPP2R5E-p62_p62.tif]

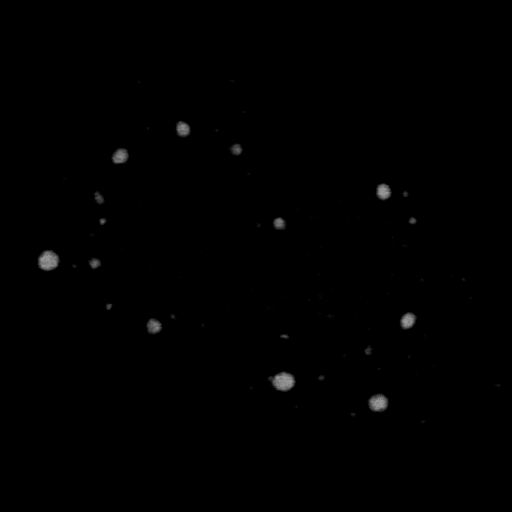

Supplement: Supplementary file 13 — Source data Fig. 4 [file 44318_2026_785_MOESM13_ESM.zip › Source data Fig. 4/4G/4G_PPP2R5E_KEAP1-p62_KEAP1.tif]

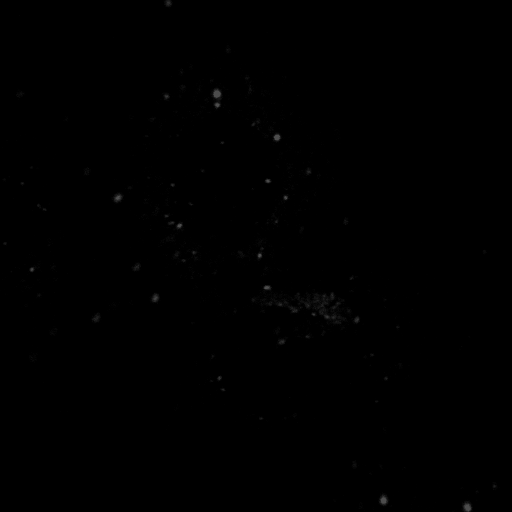

Supplement: Supplementary file 13 — Source data Fig. 4 [file 44318_2026_785_MOESM13_ESM.zip › Source data Fig. 4/4G/4G_PPP2R5ELoop mut_PPP2R5E-p62_p62.tif]

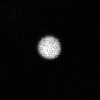

Supplement: Supplementary file 14 — Source data Fig. 5 [file 44318_2026_785_MOESM14_ESM.zip › Source data Fig. 5/5C/5C_FRAP_NT.tif]

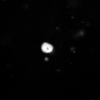

Supplement: Supplementary file 14 — Source data Fig. 5 [file 44318_2026_785_MOESM14_ESM.zip › Source data Fig. 5/5C/5C_FRAP_ABE KD.tif]

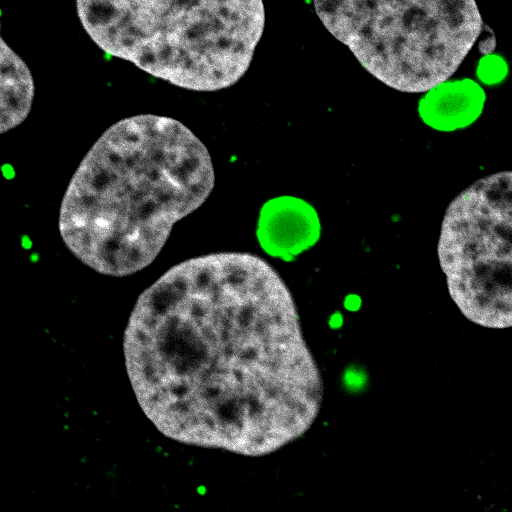

Supplement: Supplementary file 14 — Source data Fig. 5 [file 44318_2026_785_MOESM14_ESM.zip › Source data Fig. 5/5B/5B_FIP200KO NT_p62.tif]

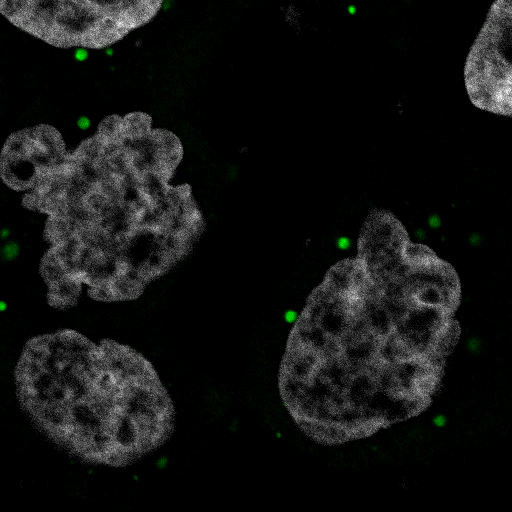

Supplement: Supplementary file 14 — Source data Fig. 5 [file 44318_2026_785_MOESM14_ESM.zip › Source data Fig. 5/5B/5B_Parental siPPP2R5A,B,E_p62.tif]

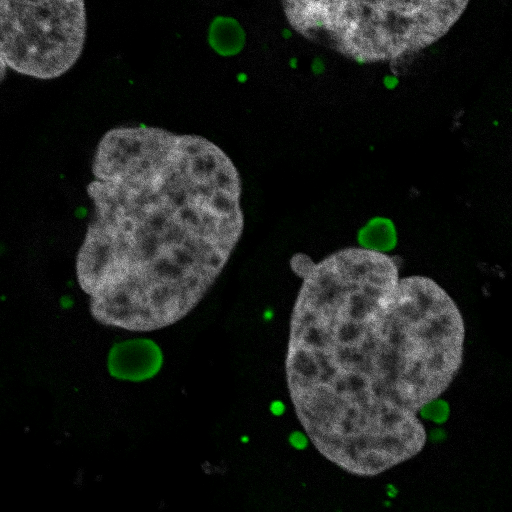

Supplement: Supplementary file 14 — Source data Fig. 5 [file 44318_2026_785_MOESM14_ESM.zip › Source data Fig. 5/5B/5B_Parental NT_p62.tif]

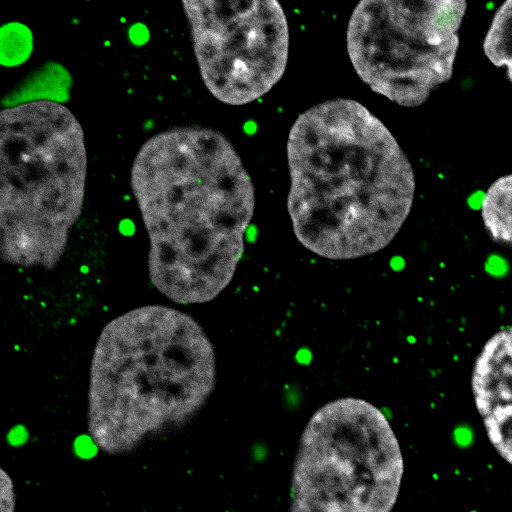

Supplement: Supplementary file 14 — Source data Fig. 5 [file 44318_2026_785_MOESM14_ESM.zip › Source data Fig. 5/5B/5B_FIP200KO siPPP2R5A,B,E_p62.tif]

Figure 6F

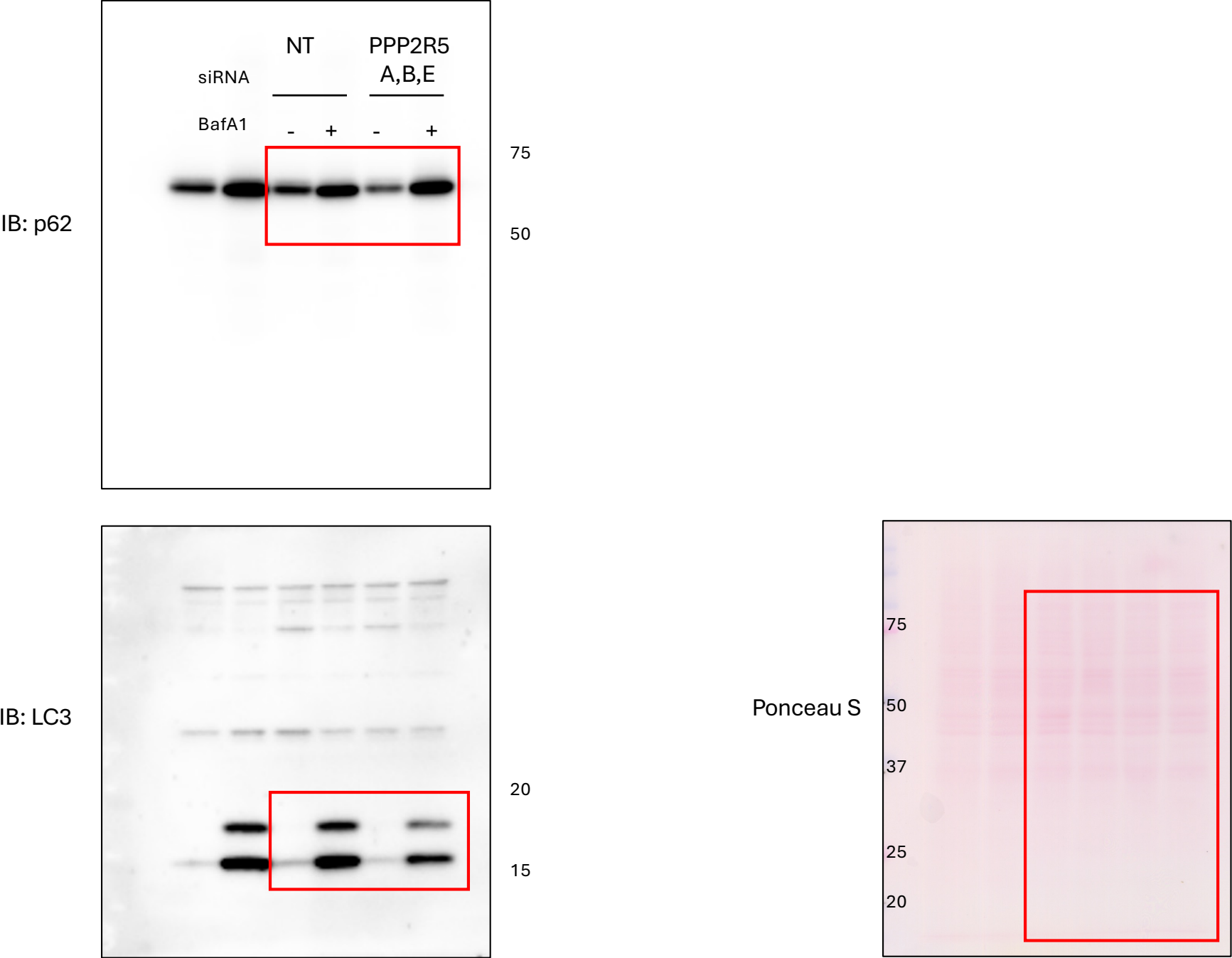

Supplement: Supplementary file 15 — Source data Fig. 6 [file 44318_2026_785_MOESM15_ESM.zip › Source data Fig. 6/6F/6F.pdf]

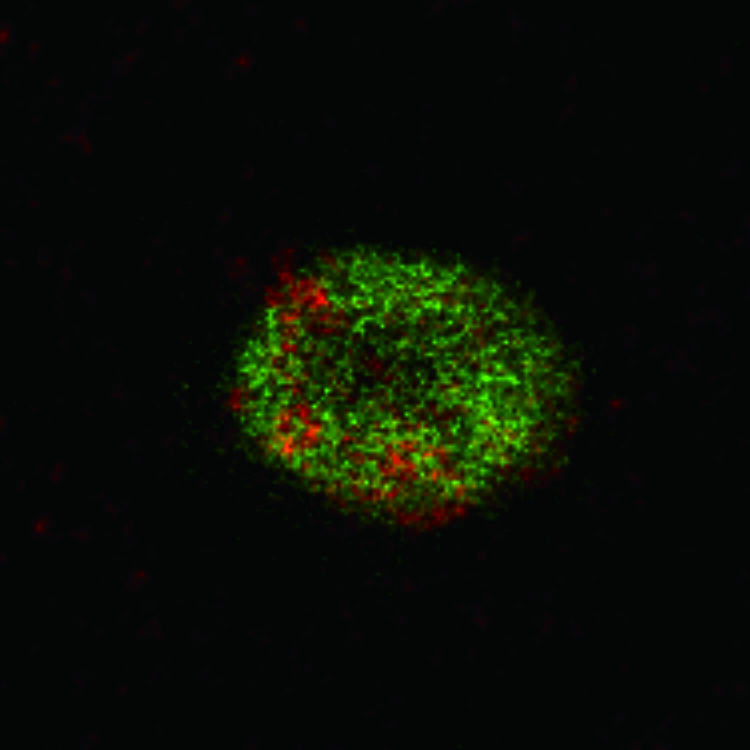

Supplement: Supplementary file 15 — Source data Fig. 6 [file 44318_2026_785_MOESM15_ESM.zip › Source data Fig. 6/6A/6A_p62-LC3_TKD_Merge.tif]
