# Supplementary figures and images for: Phosphorylation tunes p62 condensates to drive autophagic degradation of ubiquitinated proteins (part 2 of 2)
Source: EMBO J. 2026 May 5;45(12):4061–93. doi: 10.1038/s44318-026-00785-1 (PMC13270050; doi:10.1038/s44318-026-00785-1)

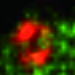

Supplement: Supplementary file 15 — Source data Fig. 6 [file 44318_2026_785_MOESM15_ESM.zip › Source data Fig. 6/6A/6A_p62-LC3_NT_HM_Merge.tif]

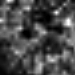

Supplement: Supplementary file 15 — Source data Fig. 6 [file 44318_2026_785_MOESM15_ESM.zip › Source data Fig. 6/6A/6A_p62-LC3_TKD_HM_p62.tif]

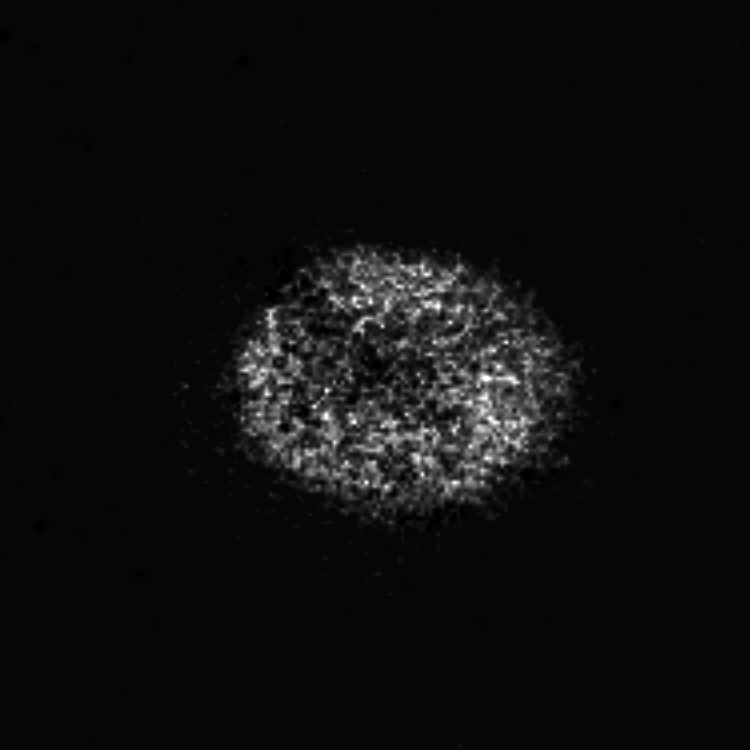

Supplement: Supplementary file 15 — Source data Fig. 6 [file 44318_2026_785_MOESM15_ESM.zip › Source data Fig. 6/6A/6A_p62-LC3_TKD_p62.tif]

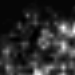

Supplement: Supplementary file 15 — Source data Fig. 6 [file 44318_2026_785_MOESM15_ESM.zip › Source data Fig. 6/6A/6A_p62-LC3_NT_HM_p62.tif]

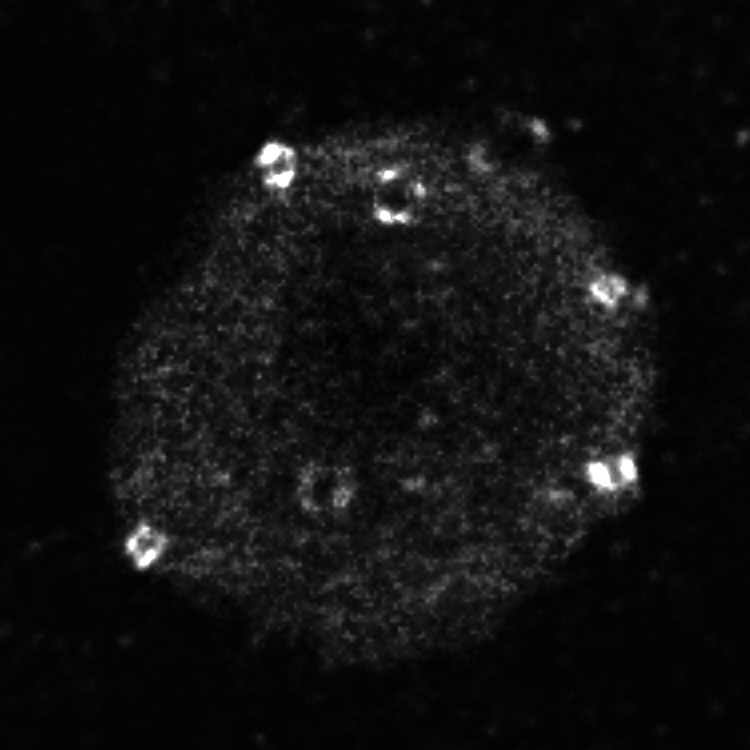

Supplement: Supplementary file 15 — Source data Fig. 6 [file 44318_2026_785_MOESM15_ESM.zip › Source data Fig. 6/6A/6A_p62-LC3_NT_LC3.tif]

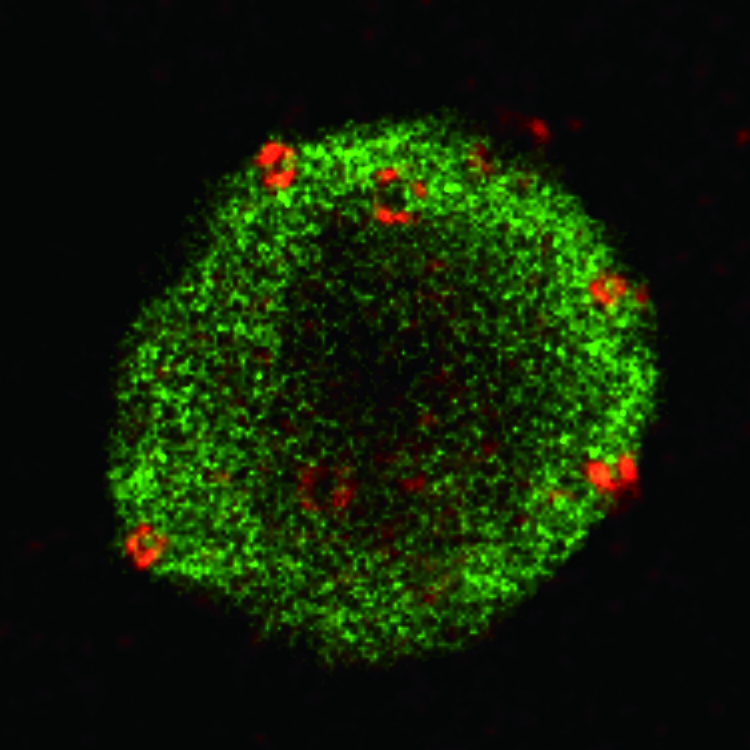

Supplement: Supplementary file 15 — Source data Fig. 6 [file 44318_2026_785_MOESM15_ESM.zip › Source data Fig. 6/6A/6A_p62-LC3_NT_Merge.tif]

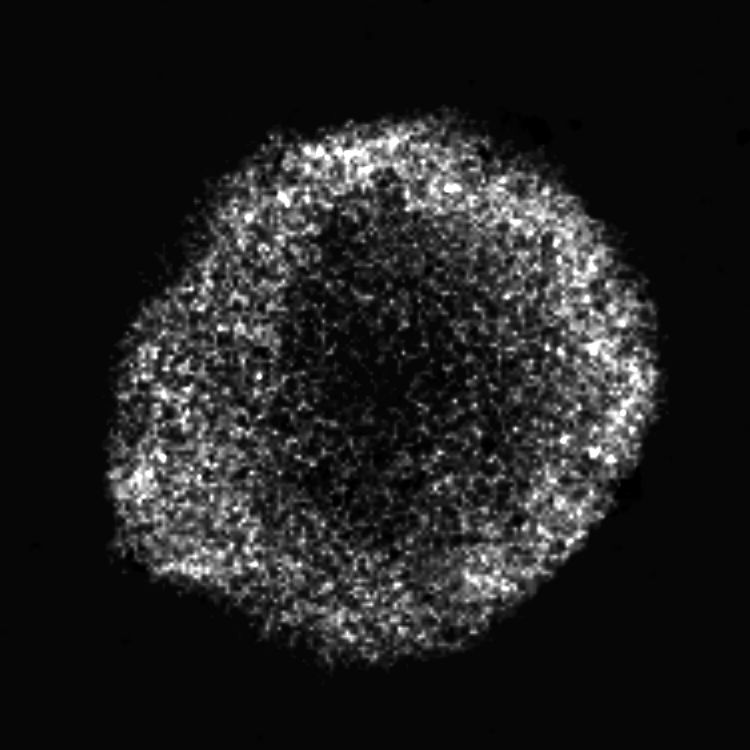

Supplement: Supplementary file 15 — Source data Fig. 6 [file 44318_2026_785_MOESM15_ESM.zip › Source data Fig. 6/6A/6A_p62-LC3_NT_p62.tif]

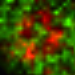

Supplement: Supplementary file 15 — Source data Fig. 6 [file 44318_2026_785_MOESM15_ESM.zip › Source data Fig. 6/6A/6A_p62-LC3_TKD_HM_Merge.tif]

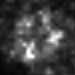

Supplement: Supplementary file 15 — Source data Fig. 6 [file 44318_2026_785_MOESM15_ESM.zip › Source data Fig. 6/6A/6A_p62-LC3_TKD_HM_LC3.tif]

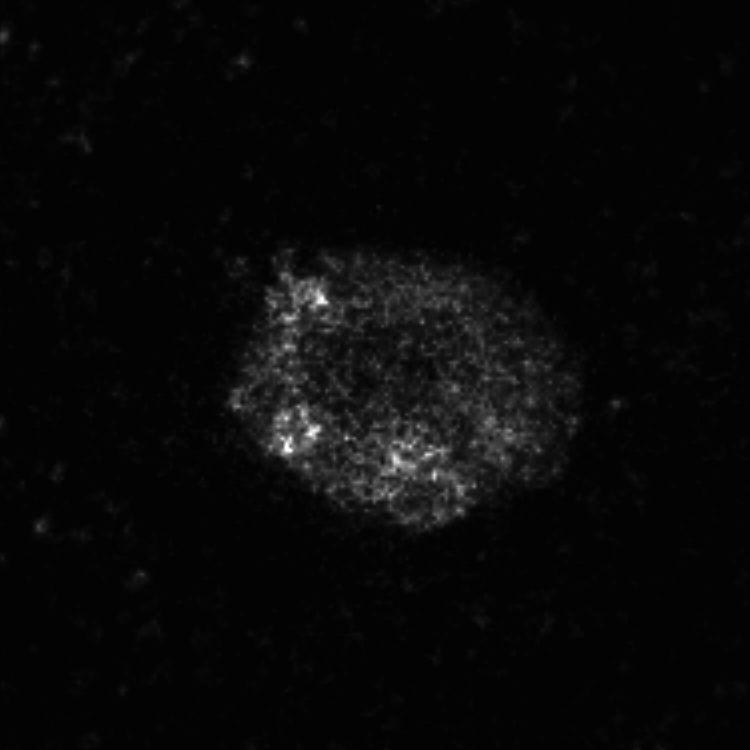

Supplement: Supplementary file 15 — Source data Fig. 6 [file 44318_2026_785_MOESM15_ESM.zip › Source data Fig. 6/6A/6A_p62-LC3_TKD_LC3.tif]

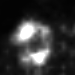

Supplement: Supplementary file 15 — Source data Fig. 6 [file 44318_2026_785_MOESM15_ESM.zip › Source data Fig. 6/6A/6A_p62-LC3_NT_HM_LC3.tif]

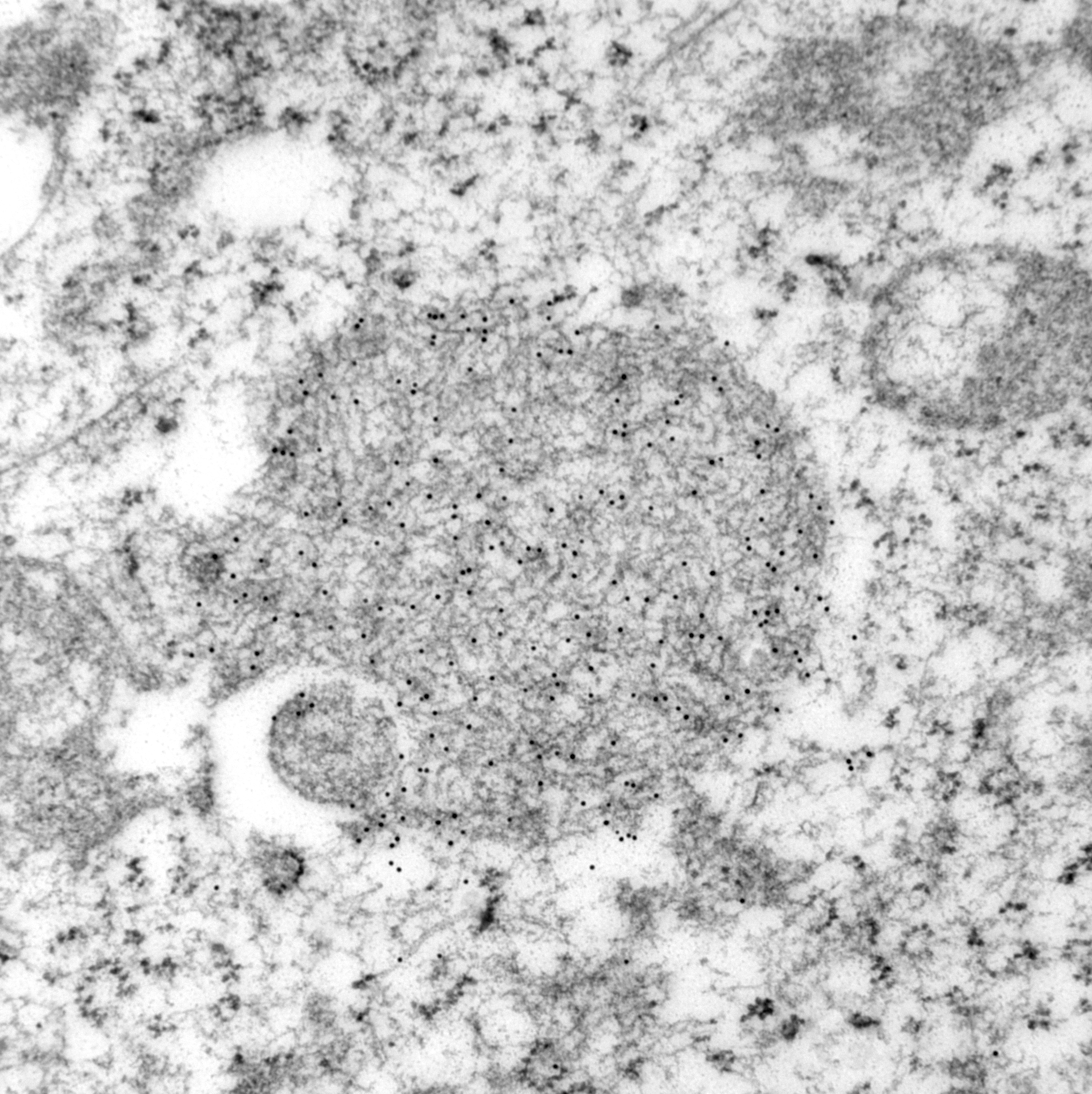

Supplement: Supplementary file 15 — Source data Fig. 6 [file 44318_2026_785_MOESM15_ESM.zip › Source data Fig. 6/6B/6B_X15k_8bit.tif]

Figure 6E

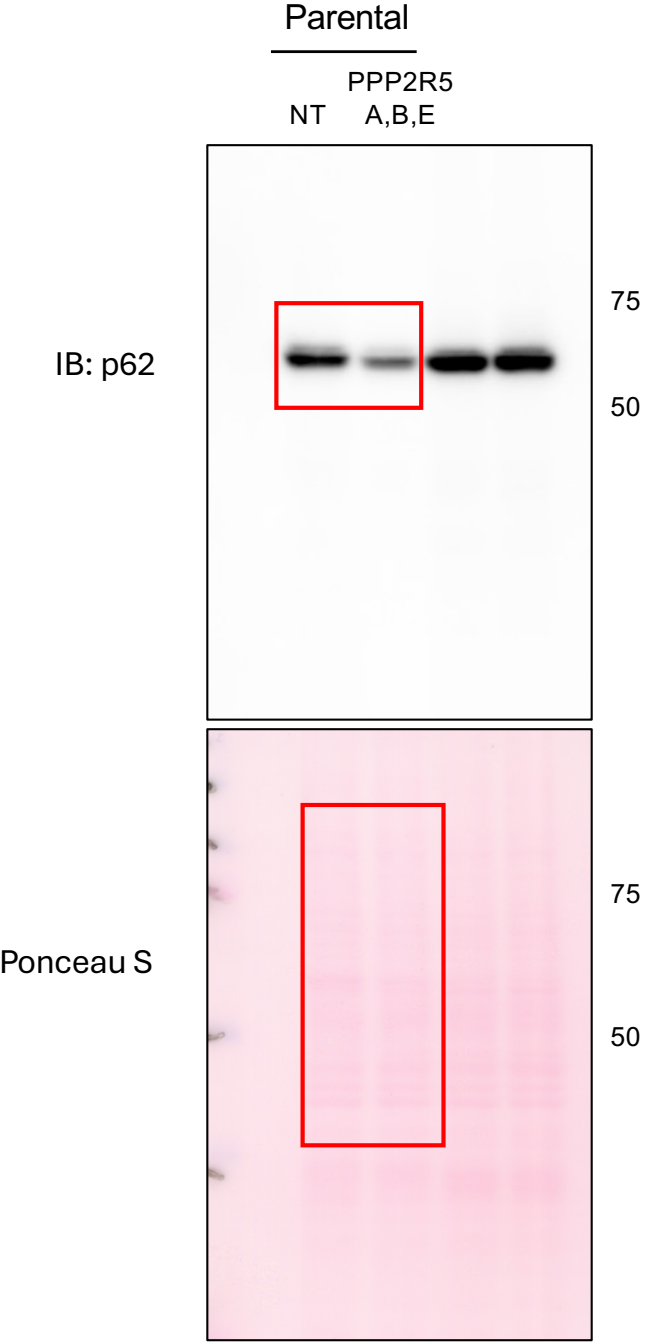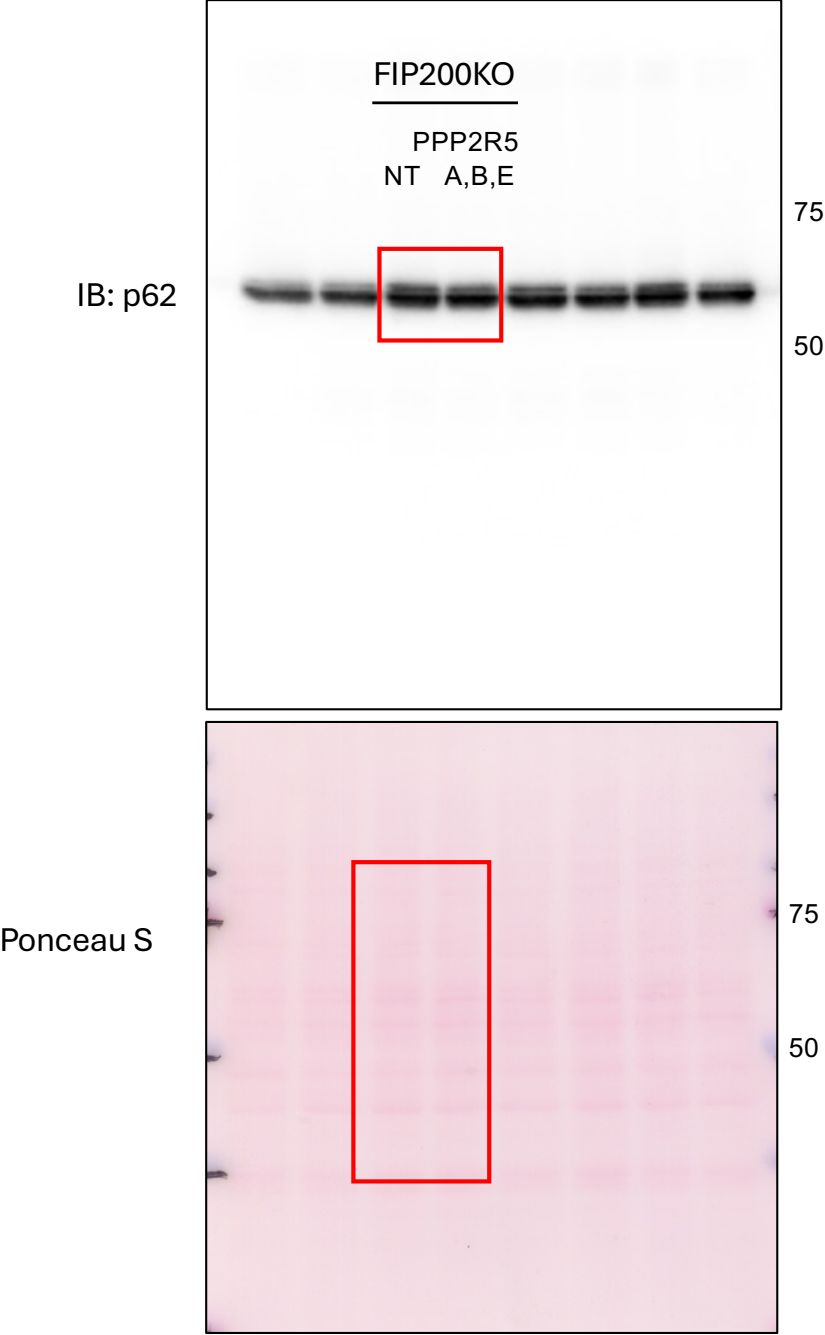

Supplement: Supplementary file 15 — Source data Fig. 6 [file 44318_2026_785_MOESM15_ESM.zip › Source data Fig. 6/6E/6E.pdf]

Figure 7D

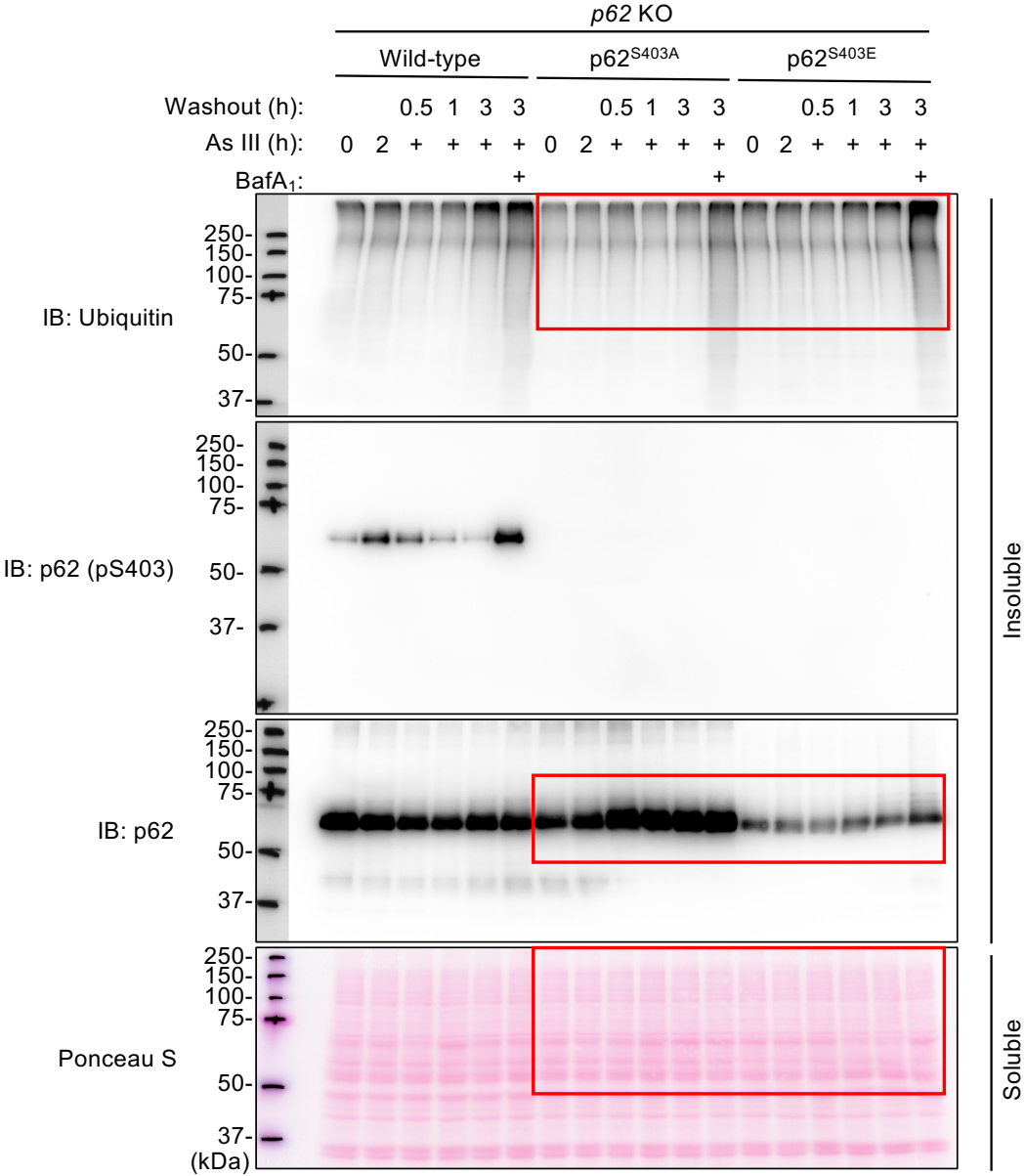

Supplement: Supplementary file 16 — Source data Fig. 7 [file 44318_2026_785_MOESM16_ESM.zip › Source data Fig. 7/7D/7D.pdf]

Figure 7C

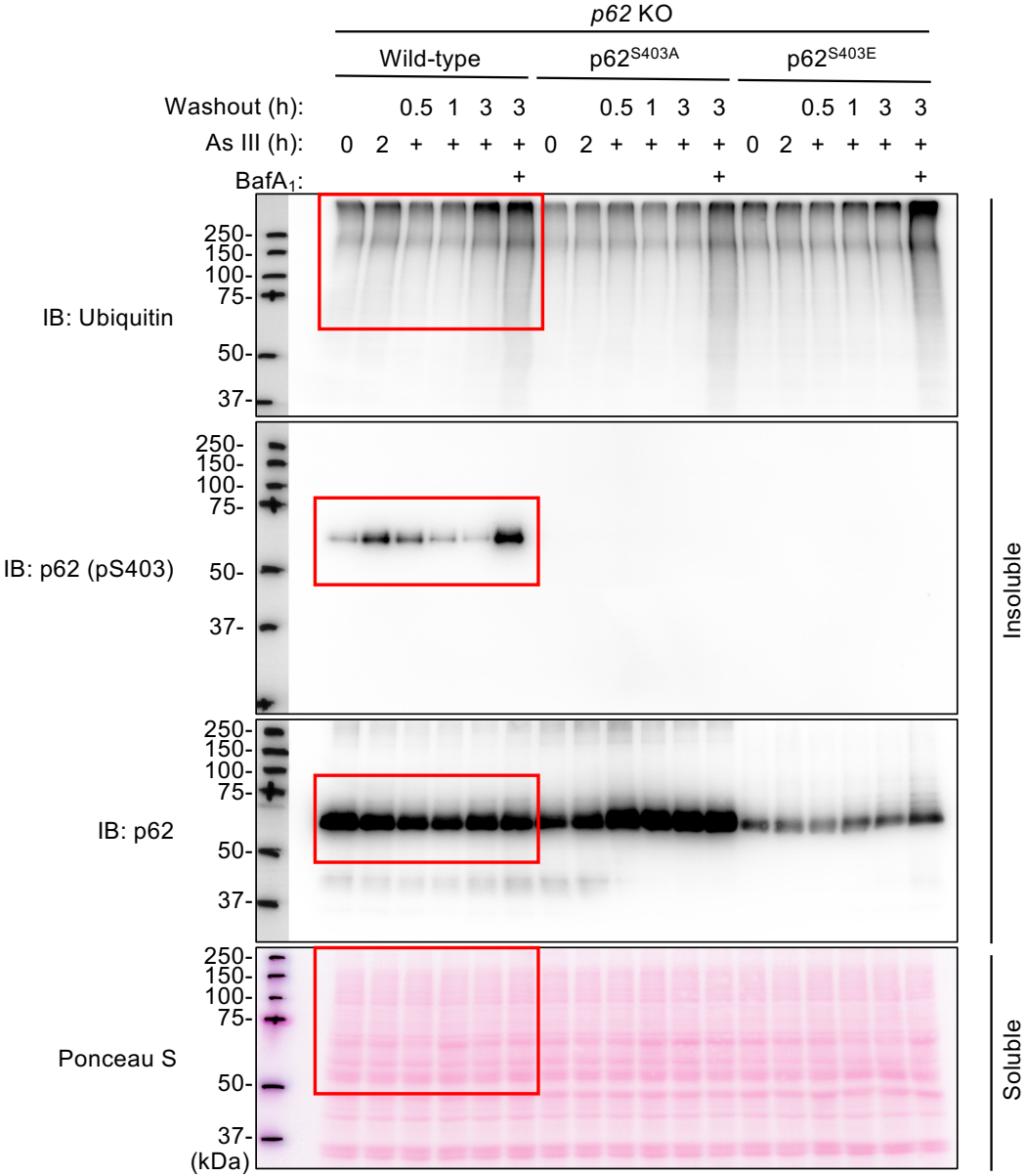

Supplement: Supplementary file 16 — Source data Fig. 7 [file 44318_2026_785_MOESM16_ESM.zip › Source data Fig. 7/7C/7C.pdf]

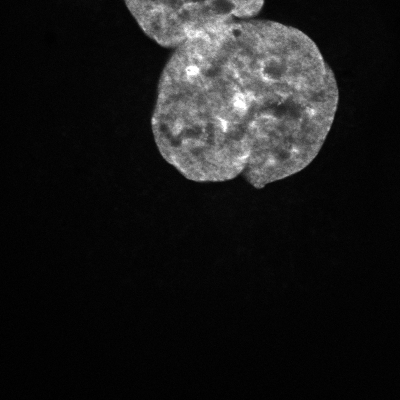

Supplement: Supplementary file 16 — Source data Fig. 7 [file 44318_2026_785_MOESM16_ESM.zip › Source data Fig. 7/7B/AsIII.tif]

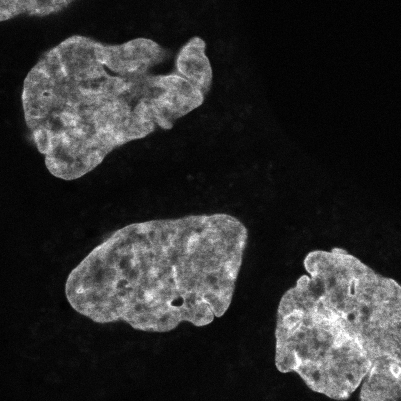

Supplement: Supplementary file 16 — Source data Fig. 7 [file 44318_2026_785_MOESM16_ESM.zip › Source data Fig. 7/7B/control.tif]

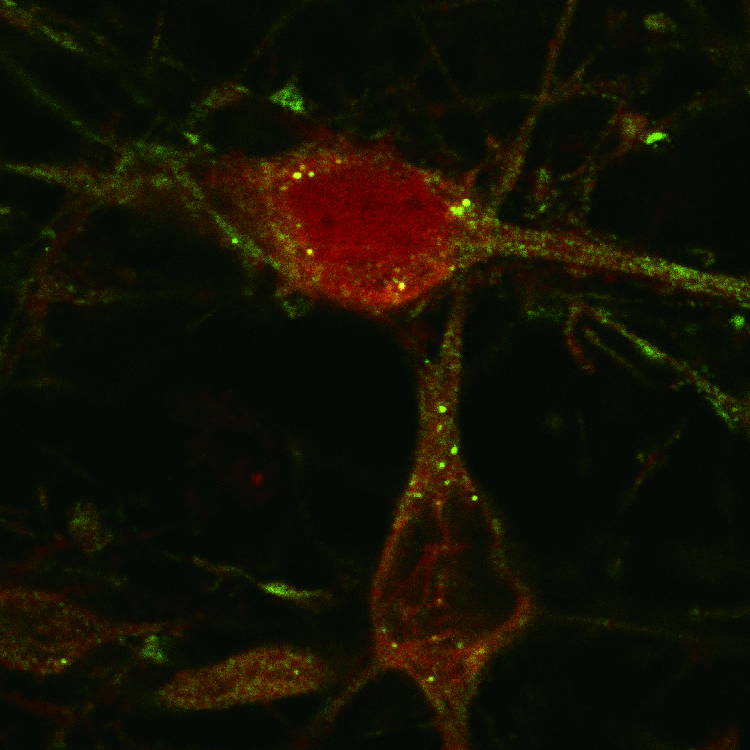

Supplement: Supplementary file 16 — Source data Fig. 7 [file 44318_2026_785_MOESM16_ESM.zip › Source data Fig. 7/7E/7E_p62S405E:S405E_Merge.tif]

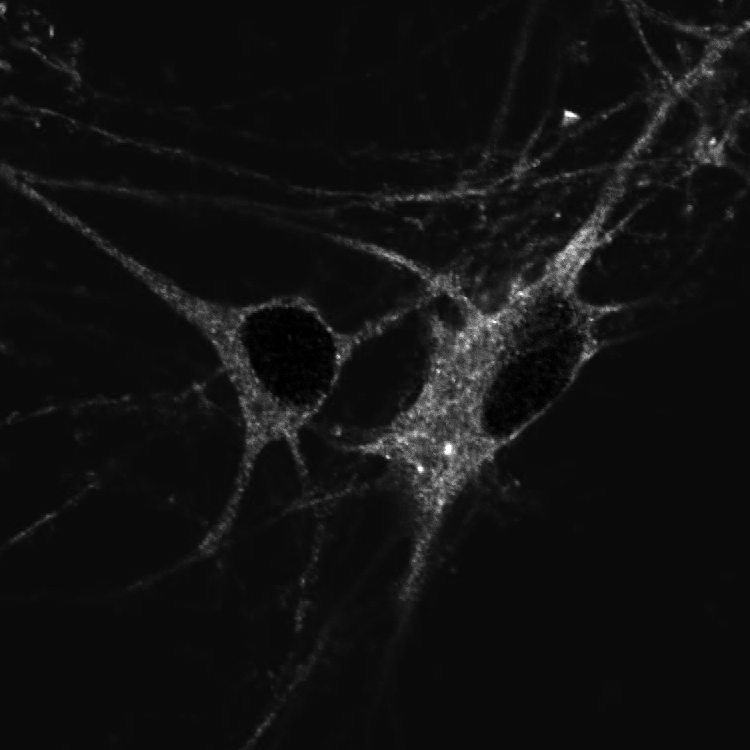

Supplement: Supplementary file 16 — Source data Fig. 7 [file 44318_2026_785_MOESM16_ESM.zip › Source data Fig. 7/7E/7E_p62+:+_p62.tif]

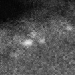

Supplement: Supplementary file 16 — Source data Fig. 7 [file 44318_2026_785_MOESM16_ESM.zip › Source data Fig. 7/7E/7E_p62S405E:S405E_HM_Ub.tif]

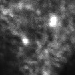

Supplement: Supplementary file 16 — Source data Fig. 7 [file 44318_2026_785_MOESM16_ESM.zip › Source data Fig. 7/7E/7E_p62+:+_HM_p62.tif]

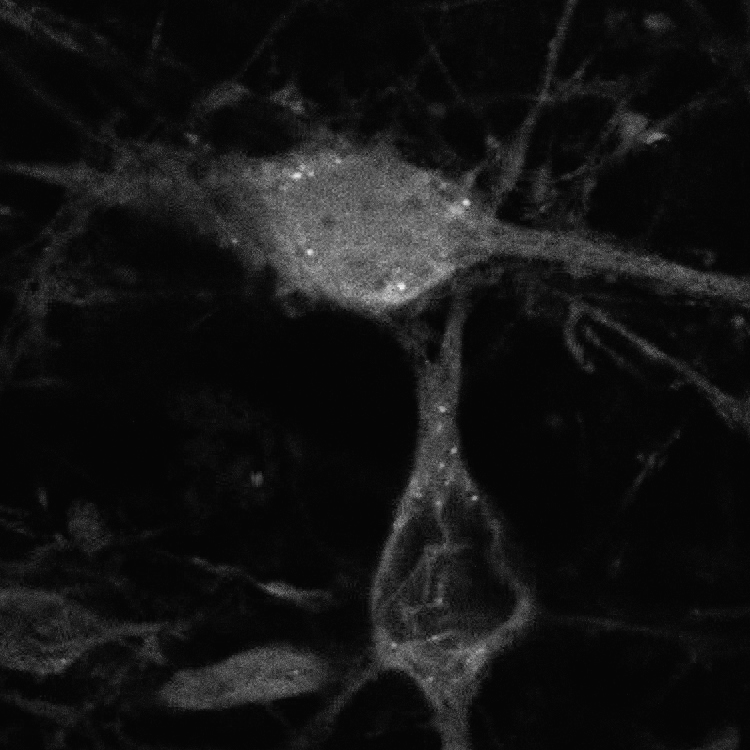

Supplement: Supplementary file 16 — Source data Fig. 7 [file 44318_2026_785_MOESM16_ESM.zip › Source data Fig. 7/7E/7E_p62S405E:S405E_Ub.tif]

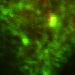

Supplement: Supplementary file 16 — Source data Fig. 7 [file 44318_2026_785_MOESM16_ESM.zip › Source data Fig. 7/7E/7E_p62+:+_HM_Merge.tif]

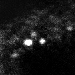

Supplement: Supplementary file 16 — Source data Fig. 7 [file 44318_2026_785_MOESM16_ESM.zip › Source data Fig. 7/7E/7E_p62S405E:S405E_HM_p62.tif]

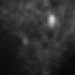

Supplement: Supplementary file 16 — Source data Fig. 7 [file 44318_2026_785_MOESM16_ESM.zip › Source data Fig. 7/7E/7E_p62+:+_HM_Ub.tif]

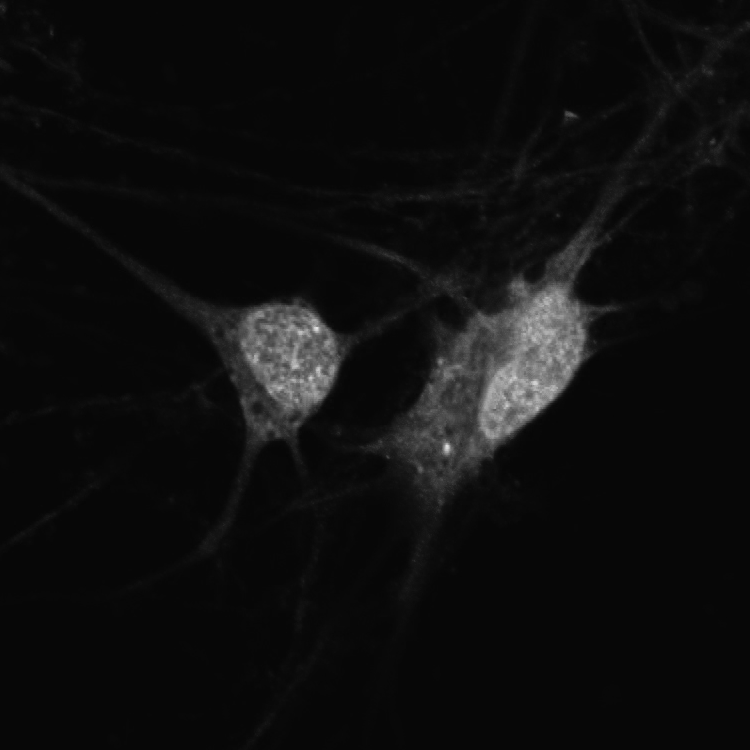

Supplement: Supplementary file 16 — Source data Fig. 7 [file 44318_2026_785_MOESM16_ESM.zip › Source data Fig. 7/7E/7E_p62+:+_Ub.tif]

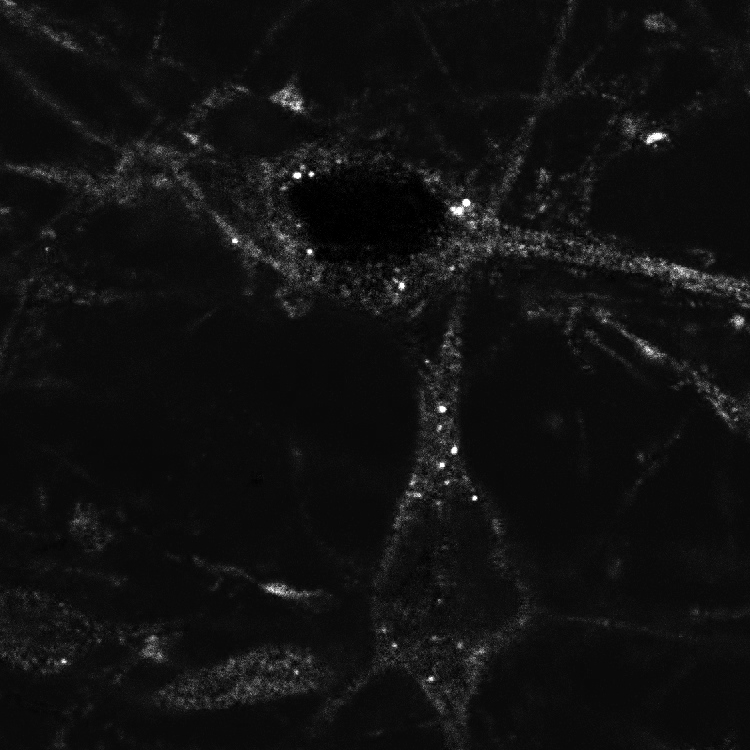

Supplement: Supplementary file 16 — Source data Fig. 7 [file 44318_2026_785_MOESM16_ESM.zip › Source data Fig. 7/7E/7E_p62S405E:S405E_p62.tif]

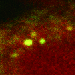

Supplement: Supplementary file 16 — Source data Fig. 7 [file 44318_2026_785_MOESM16_ESM.zip › Source data Fig. 7/7E/7E_p62S405E:S405E_HM_Merge.tif]

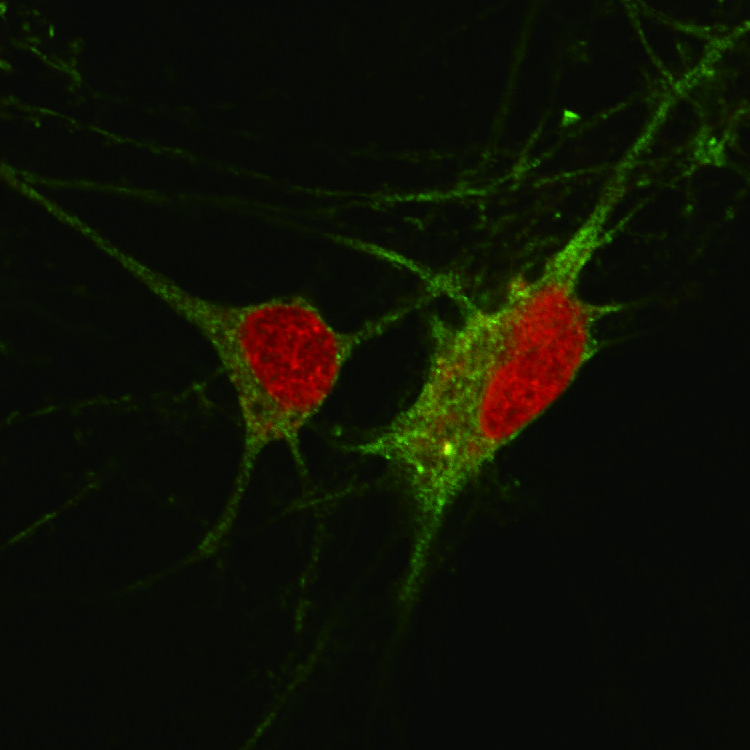

Supplement: Supplementary file 16 — Source data Fig. 7 [file 44318_2026_785_MOESM16_ESM.zip › Source data Fig. 7/7E/7E_p62+:+_Merge.tif]

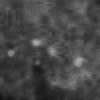

Supplement: Supplementary file 16 — Source data Fig. 7 [file 44318_2026_785_MOESM16_ESM.zip › Source data Fig. 7/7G/7G_p62+:+_HM_Merge_Ub.tif]

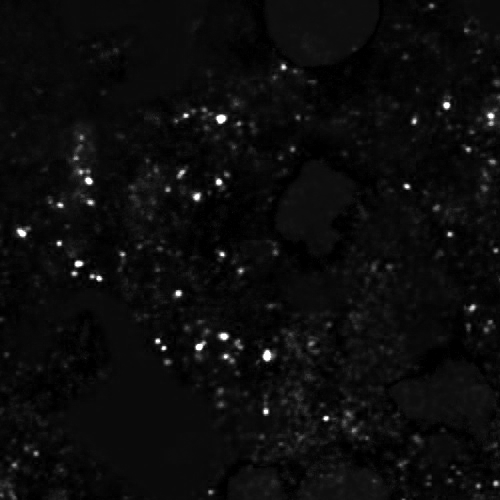

Supplement: Supplementary file 16 — Source data Fig. 7 [file 44318_2026_785_MOESM16_ESM.zip › Source data Fig. 7/7G/7G_p62+:+_p62.tif]

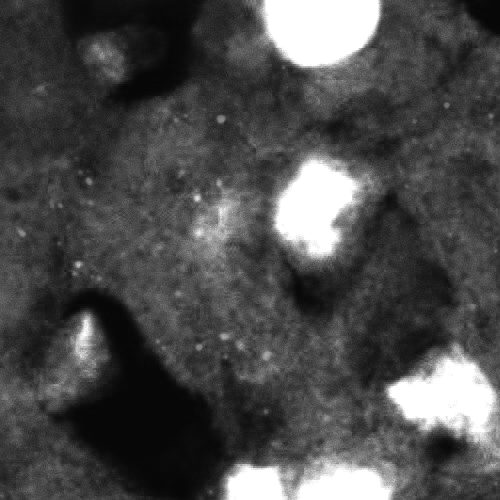

Supplement: Supplementary file 16 — Source data Fig. 7 [file 44318_2026_785_MOESM16_ESM.zip › Source data Fig. 7/7G/7G_p62+:+_Ub.tif]

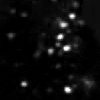

Supplement: Supplementary file 16 — Source data Fig. 7 [file 44318_2026_785_MOESM16_ESM.zip › Source data Fig. 7/7G/7G_p62S40E:S405E_HM_p62.tif]

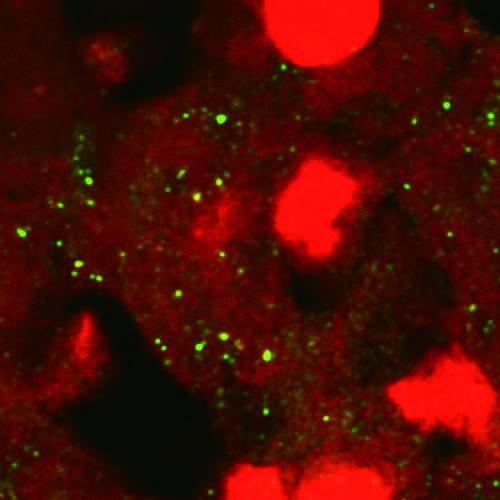

Supplement: Supplementary file 16 — Source data Fig. 7 [file 44318_2026_785_MOESM16_ESM.zip › Source data Fig. 7/7G/7G_p62+:+_Merge.tif]

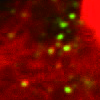

Supplement: Supplementary file 16 — Source data Fig. 7 [file 44318_2026_785_MOESM16_ESM.zip › Source data Fig. 7/7G/7G_p62S40E:S405E_HM_Merge.tif]

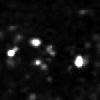

Supplement: Supplementary file 16 — Source data Fig. 7 [file 44318_2026_785_MOESM16_ESM.zip › Source data Fig. 7/7G/7G_62+:+_HM_Merge_p62.tif]

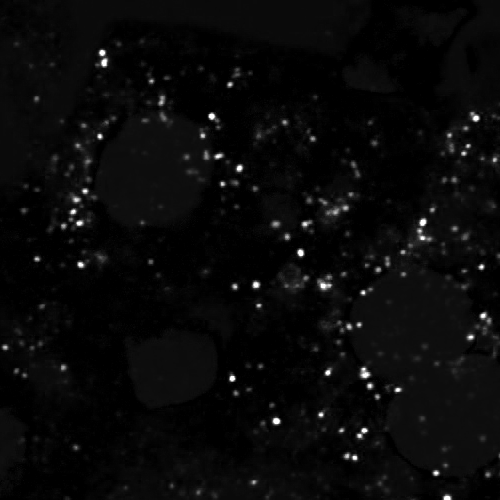

Supplement: Supplementary file 16 — Source data Fig. 7 [file 44318_2026_785_MOESM16_ESM.zip › Source data Fig. 7/7G/7G_p62S405E:S405E_p62.tif]

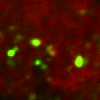

Supplement: Supplementary file 16 — Source data Fig. 7 [file 44318_2026_785_MOESM16_ESM.zip › Source data Fig. 7/7G/7G_p62+:+_HM_Merge.tif]

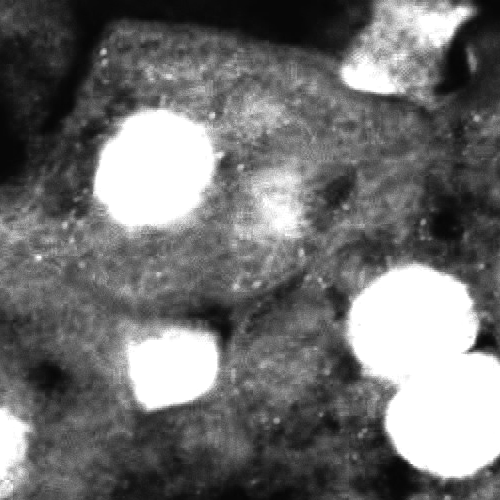

Supplement: Supplementary file 16 — Source data Fig. 7 [file 44318_2026_785_MOESM16_ESM.zip › Source data Fig. 7/7G/7G_p62S405E:S405E_Ub.tif]

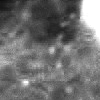

Supplement: Supplementary file 16 — Source data Fig. 7 [file 44318_2026_785_MOESM16_ESM.zip › Source data Fig. 7/7G/7G_p62S40E:S405E_HM_Ub.tif]

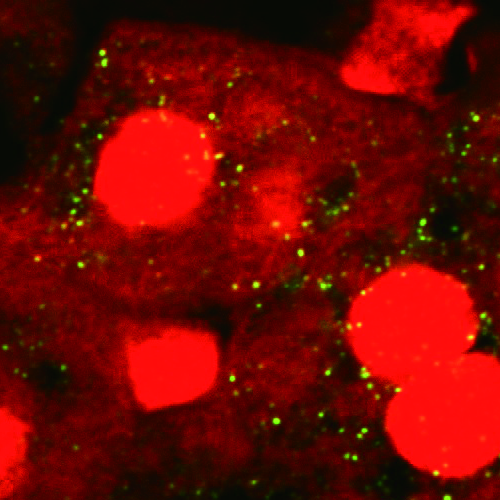

Supplement: Supplementary file 16 — Source data Fig. 7 [file 44318_2026_785_MOESM16_ESM.zip › Source data Fig. 7/7G/7G_p62S405E:S405E_Merge.tif]

Figure 7F

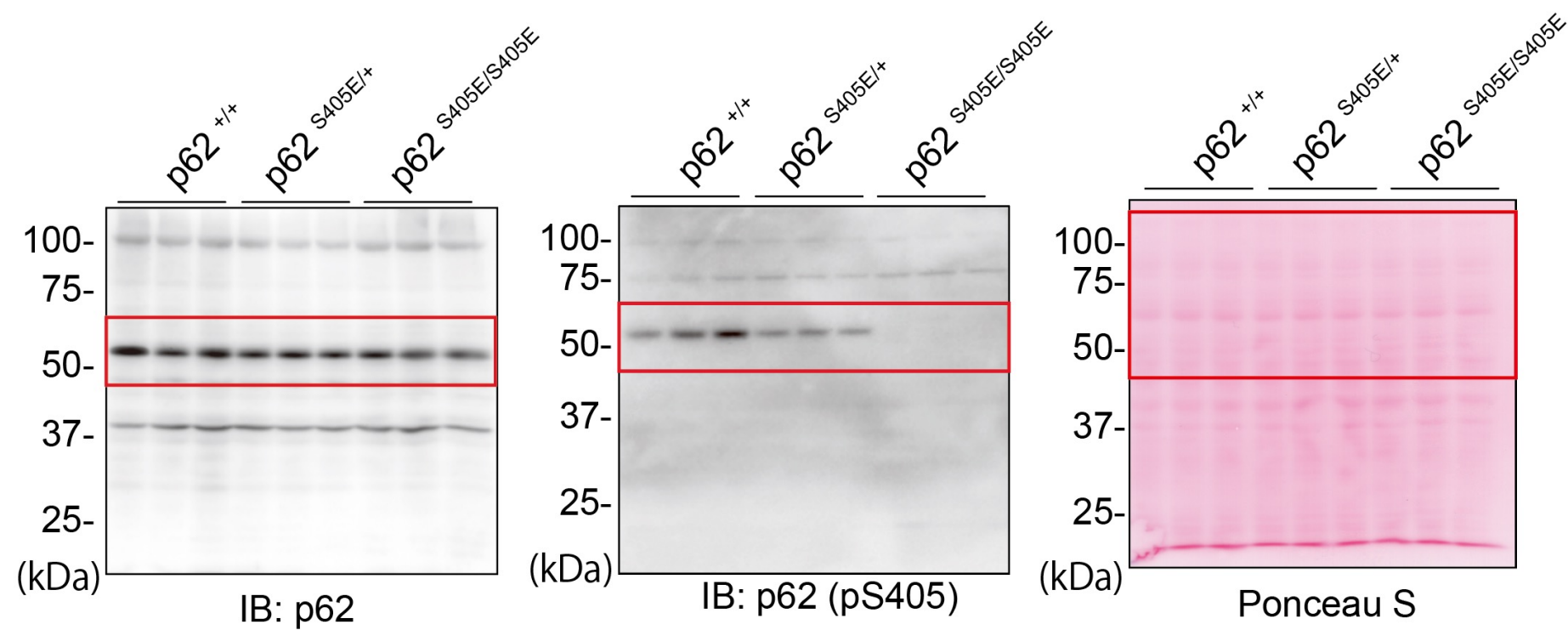

Supplement: Supplementary file 16 — Source data Fig. 7 [file 44318_2026_785_MOESM16_ESM.zip › Source data Fig. 7/7F/7F.pdf]

Figure 7A

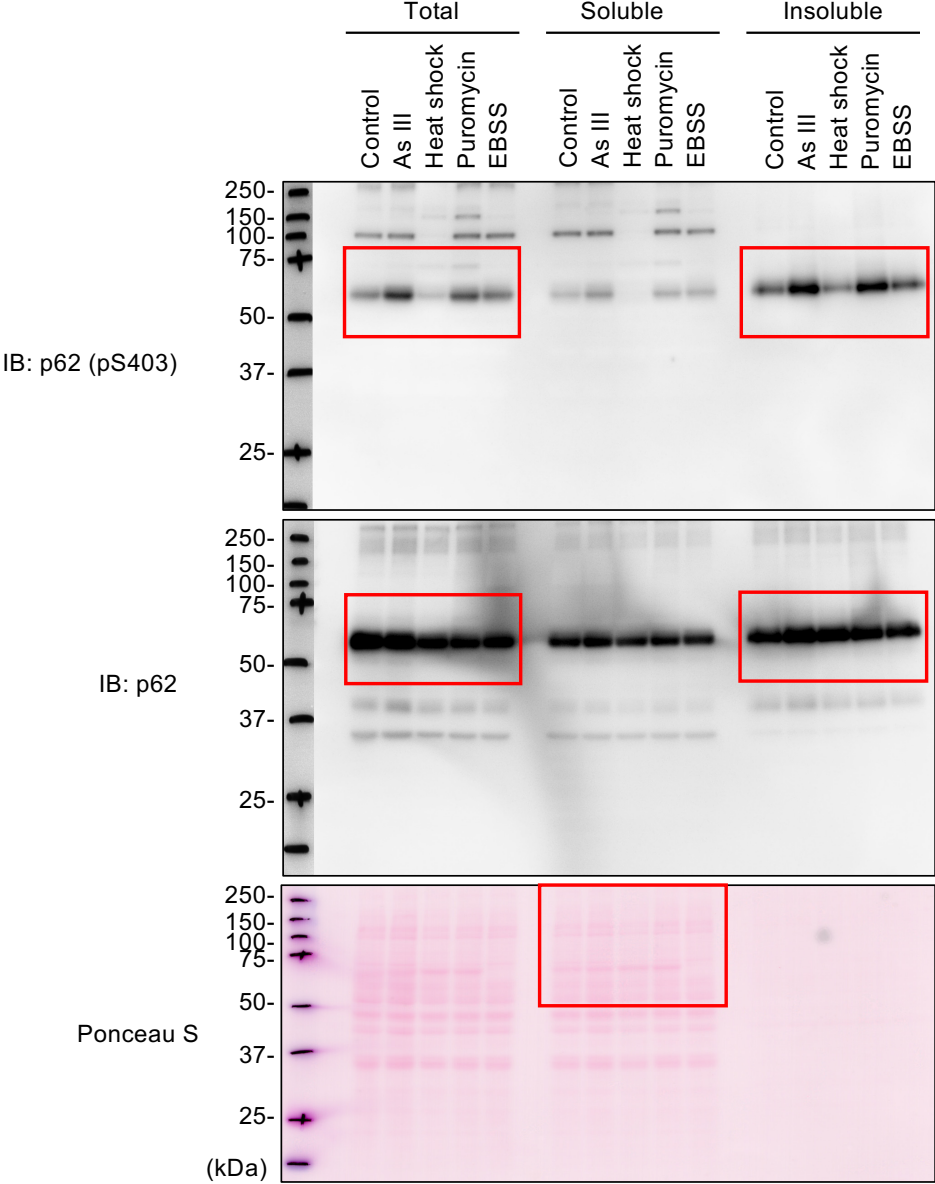

Supplement: Supplementary file 16 — Source data Fig. 7 [file 44318_2026_785_MOESM16_ESM.zip › Source data Fig. 7/7A/7A.pdf]

Figure 7H

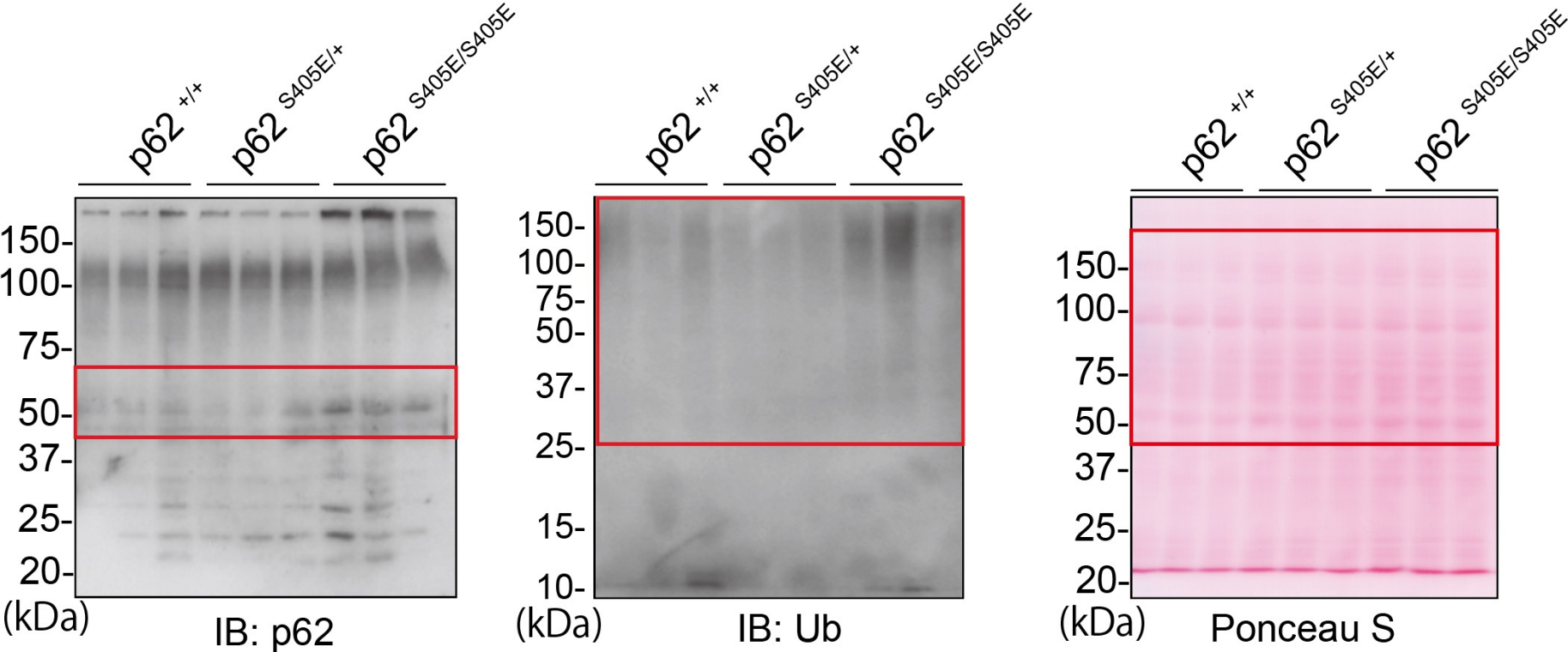

Supplement: Supplementary file 16 — Source data Fig. 7 [file 44318_2026_785_MOESM16_ESM.zip › Source data Fig. 7/7H/7H.pdf]
