# Supplementary material for: Phosphorylation tunes p62 condensates to drive autophagic degradation of ubiquitinated proteins
Source: EMBO J. 2026 May 5;45(12):4061–93. doi: 10.1038/s44318-026-00785-1 (PMC13270050; doi:10.1038/s44318-026-00785-1)
Supplement: Supplementary file 18 — Expanded View Figures [file 44318_2026_785_MOESM18_ESM.pdf]

# Expanded View Figures

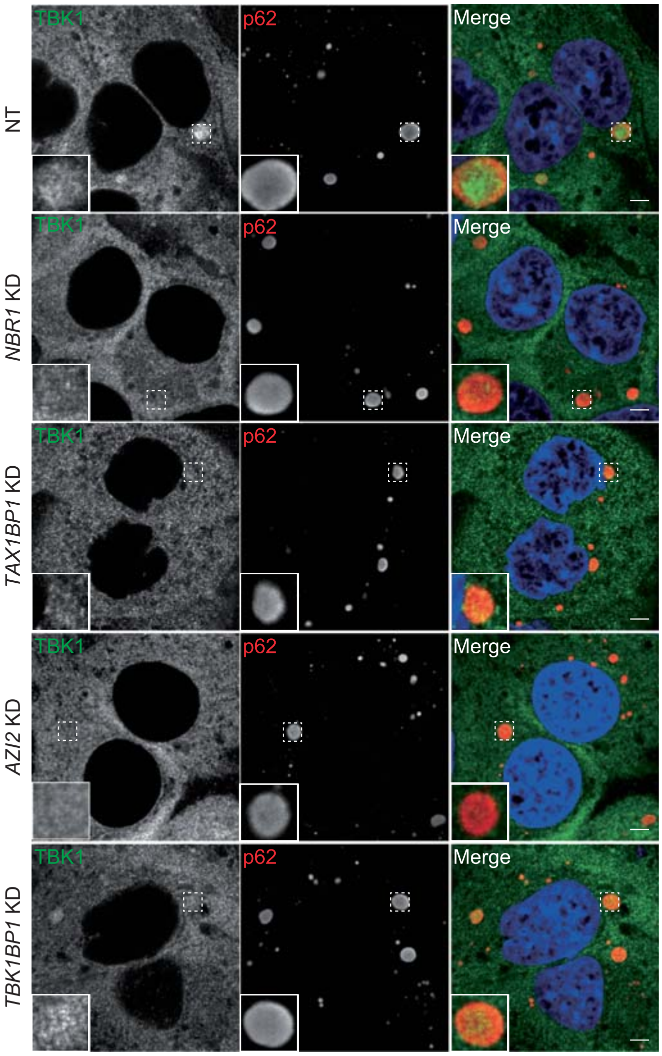


**Figure EV1. TBK1 localizes to p62 bodies.**

Immunofluorescence microscopy. Huh-1 cells transfected with siRNAs targeting *NBR1*, *TAX1BP1*, *AZI2*, or *TBK1BP1* were stained with antibodies against p62 and TBK1. Scale bars, 5 μm. Source data are available online for this figure.


© The Author(s)

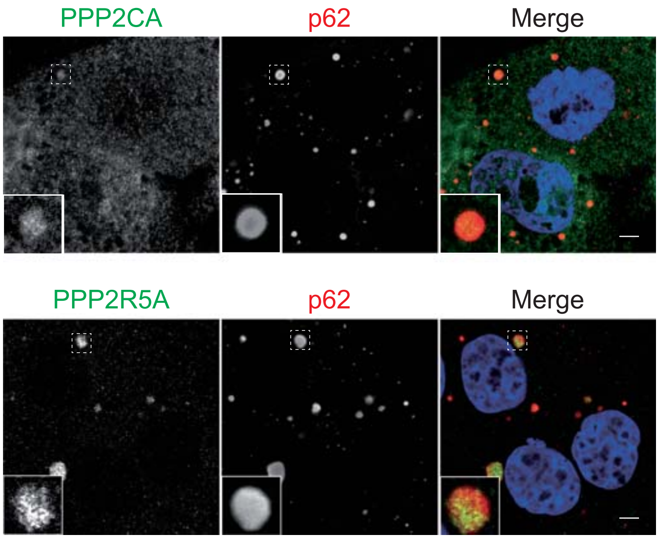


**Figure EV2. PPP2CA and PPP2R5A colocalize with p62 bodies.**

Immunofluorescence microscopy of Huh-1 cells stained for PPP2CA and p62 (upper) or PPP2R5A and p62 (bottom). Scale bars, 5 μm. Source data are available online for this figure.

© The Author(s)

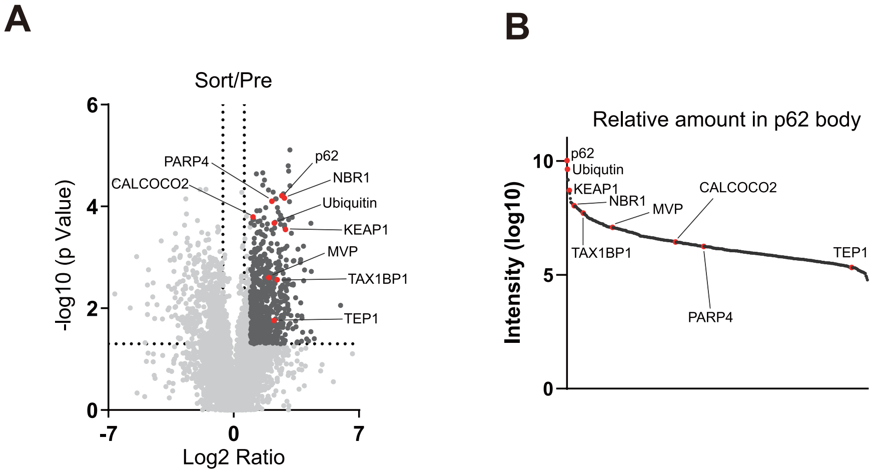


C

| Parcentage of Total protein abundance (%) | | |
|---|---|---|
| p62 | 42.8073 | ± 2.0507 |
| Ubiquitin | 17.7471 | ± 0.6177 |
| KEAP1 | 2.0685 | ± 0.2685 |
| NBR1 | 0.4443 | ± 0.0205 |
| TAX1BP1 | 0.2088 | ± 0.0252 |
| CALCOCO2 | 0.0113 | ± 0.0006 |
| MVP | 0.0495 | ± 0.0030 |
| PARP4 | 0.073 | ± 0.0005 |
| TEP1 | 0.0009 | ± 0.0004 |

mean ± SD (%)

**Figure EV3. Proteomic analysis of p62 bodies.**

(**A**) Proteomic profiles of pre- and post-sorted p62 bodies isolated from mEGFP–p62–expressing Huh-1 cells ($n = 3$). Statistics: two-tailed unpaired $t$ test. Selective autophagy receptors (NBR1, TAX1BP1, and CALCOCO2) and known client proteins (Ubiquitin, KEAP1, PARP4, and TEP1) are highlighted in red. (**B**) Relative abundance of each protein in p62 bodies was calculated based on label-free quantification (LFQ) intensity. Data are presented as the mean from three independent experiments. (**C**) The abundance ratio of each protein was calculated from the relative abundance data shown in (**B**). The total amount was defined as the sum of abundances of all p62 body–enriched proteins. Source data are available online for this figure.

 © The Author(s)

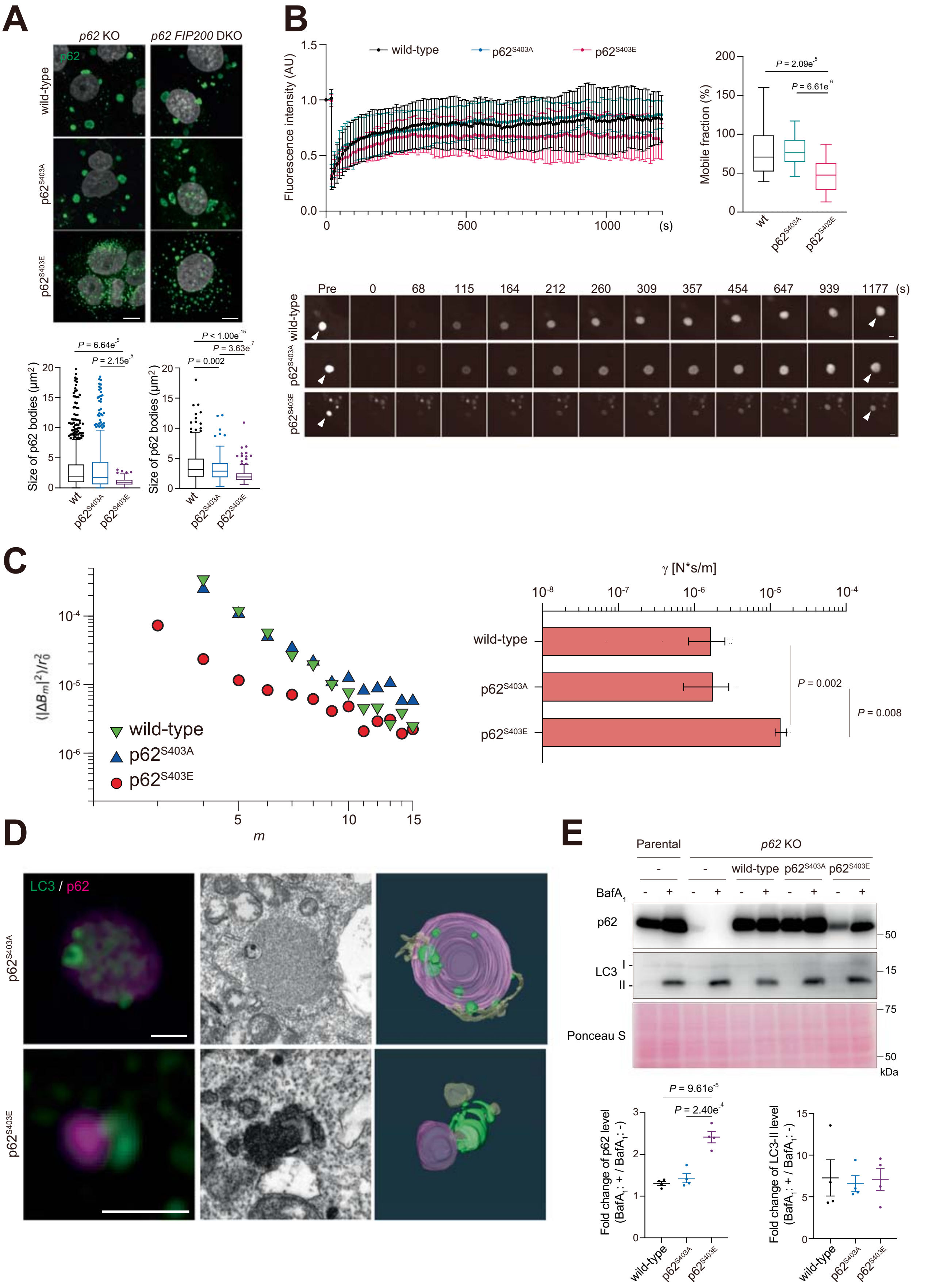
A
p62 KO
p62 FIP200 DKO
wild-type
p62S403A
p62S403E
Size of p62 bodies (μm²)
B
wild-type
p62S403A
p62S403E
Fluorescence intensity (AU)
Mobile fraction (%)
C
γ [N*s/m]
m
D
LC3 / p62
E
Parental
p62 KO
BafA1
p62
LC3
Ponceau S
kDa
Fold change of p62 level (BafA1: + / BafA1: -)
Fold change of LC3-II level (BafA1: + / BafA1: -)

**Figure EV4. Phospho-mimetic p62 enhances p62 body function.**

(**A**) Immunofluorescence of *p62*-knock-out (KO) and *p62/FIP200* Huh-1 cells re-expressing wild-type (WT) p62, the phospho-defective mutant $p62^{S403A}$, or the phospho-mimetic mutant $p62^{S403E}$. Diameters of individual p62 bodies were quantified in p62-KO cells expressing WT p62 ($n = 1154$), $p62^{S403A}$ ($n = 529$) or $p62^{S403E}$ ($n = 81$), and in *p62/FIP200* double-KO cells expressing WT p62 ($n = 349$, $p62^{S403A}$ ($n = 187$) or $p62^{S403E}$ ($n = 410$). Statistics: one-way ANOVA with Tukey's multiple-comparison test. Exact *P* values are indicated; values below the detection limit are reported as $P < 1 \times 10^{-15}$. Horizontal bars indicate medians; boxes indicate the interquartile range (25th–75th percentiles); whiskers extend to 1.5× the interquartile range. Individual data points outside this range are plotted as outliers. Scale bar, 10 µm. (**B**) FRAP analysis of GFP-tagged p62 variants. FRAP curve is shown as mean ± s.d. Mobile fractions were determined for cells expressing WT p62 ($n = 26$), $p62^{S403A}$ ($n = 23$) or $p62^{S403E}$ ($n = 31$). One-way ANOVA with Tukey's test. Scale bar, 1 µm. (**C**) Surface tension analysis of p62 condensates. Fourier spectra of condensates in p62-KO Huh-1 cells expressing WT p62, $p62^{S403A}$ or $p62^{S403E}$. The spectra were obtained by decomposing radial fluctuations into azimuthal modes (*m*) and fitted to a theoretical model of thermally driven surface fluctuations to estimate effective surface tension (γ).(Bottom) Quantification of γ (N·s/m) obtained from the fitting analysis in p62-KO Huh-1 cells expressing p62 wild-type ($n = 4$), $p62^{S403A}$ ($n = 3$) or $p62^{S403E}$ ($n = 3$). Data are presented as mean ± s.e.m. Statistics: one-way ANOVA followed by Tukey's multiple-comparison test. (**D**) Three-dimensional CLEM showing LC3 colocalization with p62 bodies in cells expressing $p62^{S403A}$ or $p62^{S403E}$. LC3 (green), p62 (magenta), and the ER (yellow). Scale bar, 1 µm. (**E**) Immunoblot analysis of autophagic flux in parental and *p62*-null cells reconstituted with WT or mutant p62 in the absence or presence of bafilomycin $A_1$ ($BafA_1$). Band intensities for p62 and LC3-II were normalised to total protein ($n = 3$). Data are mean ± s.e.m. Statistics: one-way ANOVA with Tukey's multiple-comparison test. Source data are available online for this figure.

 © The Author(s)

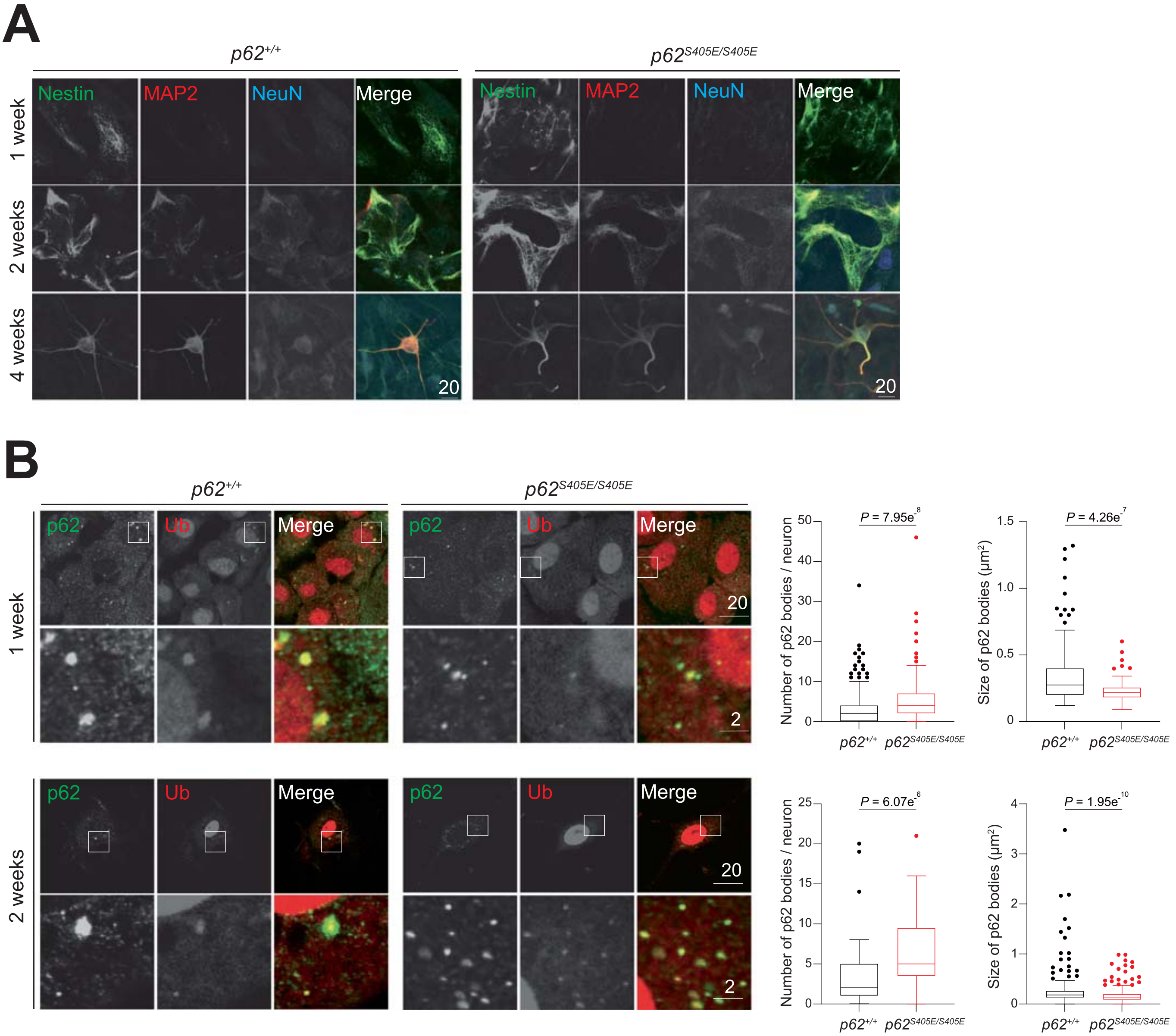


**Figure EV5. p62 body size and number in wild-type and *p62$^{S405E/S405E}$* ES-derived neurons.**

(**A**) Immunofluorescence analysis of neurons differentiated from parental and *p62$^{S405E/S405E}$* ES cells after 1, 2, and 4 weeks of culture. Both genotypes successfully differentiated into neurons, as shown by staining for neuronal markers. Scale bars, 20 μm. (**B**) Immunostaining for p62 and ubiquitin in neurons from both genotypes. The numbers and diameters of p62 bodies in wild-type neurons ($n = 247$, $n = 147$) and *p62$^{S405E/S405E}$* neurons ($n = 141$, $n = 129$) at 1 week, and in wild-type neurons ($n = 75$, $n = 241$) and *p62$^{S405E/S405E}$* neurons ($n = 45$, $n = 298$) at 2 weeks were quantified. Statistical analysis was performed using the Mann–Whitney *U* test. Horizontal bars indicate medians; boxes indicate the interquartile range (25th–75th percentiles); whiskers extend to 1.5× the interquartile range. Individual data points outside this range are plotted as outliers. Scale bars, 20 μm; enlarged views, 2 μm. Source data are available online for this figure.

© The Author(s)
