# Supplementary material for: Phosphorylation tunes p62 condensates to drive autophagic degradation of ubiquitinated proteins
Source: EMBO J. 2026 May 5;45(12):4061–93. doi: 10.1038/s44318-026-00785-1 (PMC13270050; doi:10.1038/s44318-026-00785-1)
Supplement: Supplementary file 2 — Peer Review File [file 44318_2026_785_MOESM2_ESM.pdf]

THE EMBO JOURNAL

Phosphorylation tunes p62 condensates to drive autophagic degradation of ubiquitinated proteins

Satoko Komatsu-Hirota, Keisuke Tabata, Yu-Shin Sou, Soichiro Kakuta, Jun-ichi Sakamaki, Hikaru Tsuchiya, Jiachen Li, Hiroyuki Kumeta, Yuji Sakai, Yuko Fujioka, Daisuke Noshiro, Shunsuke Shimobayashi, Tomo Kurimura, Takashi Taniguchi, Manabu Abe, Masato Koike, Hideaki Morishita, Nobuo Noda, and Masaaki Komatsu

Corresponding author(s): Masaaki Komatsu (mkomatsu@juntendo.ac.jp)

Review Timeline:

Submission Date:	22nd Dec 25
Editorial Decision:	4th Feb 26
Revision Received:	18th Feb 26
Editorial Decision:	18th Mar 26
Revision Received:	24th Mar 26
Accepted:	2nd Apr 26

Editor: William Teale

Transaction Report:

(Note: With the exception of the correction of typographical or spelling errors that could be a source of ambiguity, letters and reports are not edited. Depending on transfer agreements, referee reports obtained elsewhere may or may not be included in this compilation. Referee reports are anonymous unless the Referee chooses to sign their reports.)

Dear Masaaki,

Thank you for submitting your manuscript for consideration by the EMBO Journal. It has now been carefully considered by an arbitrating referee, whose comments are shown below.

However, before we can proceed, the referee has asked for a comprehensive point-by-point response to all of the original reviewers' concerns. Once I receive this document I will immediately share it with the arbitrating reviewer and proceed according to their advice.

Best wishes,

William

William Teale, PhD
Editor
The EMBO Journal
w.teale@embojournal.org

Referee #1:

This study linking p62 modifications to condensate properties is of considerable interest to the autophagy community.
In the revised manuscript, the authors have addressed some of the major concerns. However, they have not provided a point-by-point response to the previous reviewer comments, and as a result, many issues remain unresolved.

For example, Reviewer 1 noted:
"TBK1BP1 knockdown data in Fig 1e, Fig 2c, Fig 2d appears not consistent. Fig 1e pTBK1 intensity in the quantification seems not reduced in TBK1BP1 knockdown."

This concern has not been adequately addressed. Indeed, a clear p62 pS403 band is visible in the TAX1BP1 KD condition in Figure 2c, yet no signal is detected in Figure 2d. Such inconsistencies need to be explained or the data reconciled.

This is one of several instances where reviewer concerns remain unaddressed. Given the number of outstanding issues, I would recommend allowing a further round of revision, but the manuscript cannot be accepted for publication in its current form. The authors should be asked to provide a comprehensive point-by-point response addressing all previous comments, not only the major concerns.

Arbitrating referee

General comment

This study linking p62 modifications to condensate properties is of considerable interest to the autophagy community. In the revised manuscript, the authors have addressed some of the major concerns. However, they have not provided a point-by-point response to the previous reviewer comments, and as a result, many issues remain unresolved.

Specific comments:

Comment-1

For example, Reviewer 1 noted:

"TBK1BP1 knockdown data in Fig 1e, Fig 2c, Fig 2d appears not consistent. Fig 1e pTBK1 intensity in the quantification seems not reduced in TBK1BP1 knockdown."

Reply-1

We thank the reviewer for pointing out this issue. As detailed in our point-by-point response to Reviewer 1 (comment 3), the experiment in revised Fig. 1E was performed using *TBK1BP1* knockdown, whereas the experiments in revised Fig 2C and 2D were conducted using *TAX1BP1* (different gene) knockdown.

These panels therefore represent analyses of different proteins, and the apparent inconsistency results from comparing experiments that perturb distinct components of the TBK1 recruitment pathway.

Comment-2

This concern has not been adequately addressed. Indeed, a clear p62 pS403 band is visible in the TAX1BP1 KD condition in Figure 2c, yet no signal is detected in Figure 2d. Such inconsistencies need to be explained or the data reconciled.

Reply-2

In Huh-1 cells, siRNA knockdown is not complete, and the immunofluorescence images necessarily include both knocked-down and non−knocked-down cells; the latter retain clear p62-pS403 positivity, as shown in the additional figure provided for review (Figure R1).

When p62-pS403 levels are quantified across the entire cell population by confocal image cytometry, a clear overall reduction is observed in *TAX1BP1*-knockdown cells (revised Fig 2D), fully consistent with the immunoblot result in revised Fig 2C.

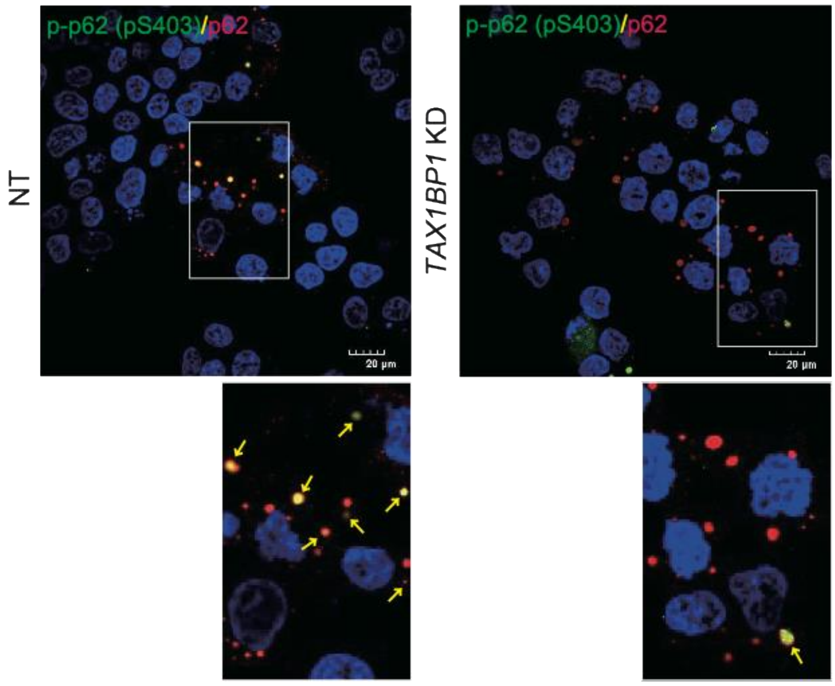

Figure R1

Comment-3

This is one of several instances where reviewer concerns remain unaddressed. Given the number of outstanding issues, I would recommend allowing a further round of revision, but the manuscript cannot be accepted for publication in its current form. The authors should be asked to provide a comprehensive point-by-point response addressing all previous comments, not only the major concerns.

Reply-3

We appreciate the reviewer's assessment. In accordance with this recommendation, we provide a comprehensive point-by-point response addressing all previous reviewer comments, including both major and minor concerns. In preparing this revision, we carefully re-examined all reviewer comments and revised the manuscript and figures accordingly in the EMBO Journal format. While the core conclusions remain unchanged, we have corrected overstatements, clarified ambiguous descriptions, and refined the wording throughout the manuscript to ensure that all interpretations are fully supported by the data. For convenience, we also provide a compiled PDF including the revised manuscript, main figures, Figure EVs, and Appendix Figures together with this point-by-point response. The complete point-by-point response is provided below.

Complete point-by-point response

Reviewer #1 (Remarks to the Author):

General comment

Previously, several studies demonstrated that S403 phosphorylation enhances p62 affinity to ubiquitin binding. In current manuscript, the authors show that phosphorylation at Ser403 determines p62 punctate size and dynamics, enhancing p62 condensate piecemeal levels and autophagic removal of ubiquitinated proteins. Specifically, several conclusions have been made: 1) the AZI2-TAX1BP1-NBR1 axis mediates Ser403 phosphorylation; 2) the phosphorylation occurs within P62 bodies; 3) PP2A dephosphorylates S403 phosphorylation; 4) KEAP1 bridges PP2A and p62; 5) S403 phosphorylation causes gelation of p62 puncta and more efficient degradation of p62 puncta. Identification of PP2A dephosphorylating pS403 is interesting. It may be useful to provide further in-depth evidence in multiple occasions. For example, in the data showing the AZI2-TAX1BP1-NBR1axis mediates p62 Ser403 phosphorylation, protein interactions may be investigated. PPP2R5A, B, E are recruited via KEAP1 as a bridge, and the direct interaction between these proteins may be tested. In vitro LLPS experiment for p62, p62 S403A and p62 S403E could be tested.

Specific comments:

Comment-1

1. It is unclear how the TBK1BP1-AZI2-TAX1BP1-NBR1 axis regulates TBK1 recruitment into p62 bodies. The interaction between TBK1 and NBR1 should be tested. Currently, it is unclear NBR1 can directly interact with TBK1. It is also interesting to test if other proteins can physically interact with TBK1.

Reply-1

We thank the reviewer for raising this mechanistic question. In response, we provide additional evidence supporting the sequential adaptor mechanism by which TBK1 is recruited into p62 condensates.

(1) TAX1BP1–NBR1 interaction is structurally and biochemically supported.

AlphaFold3 predicts a stable interface between TAX1BP1 and NBR1 (Figure R2A and North BJ *et al.*, *J Cell Biol* 2025), consistent with previously reported biochemical data (Turco E *et al.*, *Nat Commun* 2021; Bauer B *et al.*, *EMBO J* 2024).

(2) Functional relevance demonstrated by LLPS reconstitution.

In our purified-component LLPS system, a TAX1BP1 mutant defective in NBR1 binding fails to partition into NBR1–p62 condensates (Figure R2B), demonstrating that NBR1 binding is required for TAX1BP1 recruitment.

These additional data further clarify the adaptor chain leading to TBK1 recruitment into p62 condensates. Because this mechanistic refinement represents a natural extension of the present work and will be developed in a subsequent study, we have not incorporated these datasets into the main manuscript. For transparency, the AlphaFold3 structural model and the LLPS data using the NBR1-binding–deficient TAX1BP1 mutant are provided as Figure R2.

Comment-2

2. Fig 1a, why does TBK1 localize inside p62 condensates, while TBK1 pS172 signals surround these condensates?

Reply-2

Given that TBK1 pS172 marks the activated kinase (Kishore N *et al*., *J Biol Chem* 2002), the distinct distributions of TBK1 and pTBK1 suggest that TBK1 recruitment and activation occur in spatially separable steps. In our images, pTBK1 does not form a continuous ring but appears as puncta within specific subregions of p62 condensates (revised Fig 1A), indicating that TBK1 activation occurs locally rather than uniformly throughout the condensate.

Although adaptor-mediated clustering of TBK1 (*e.g.*, by AZI2, TBK1BP1, and autophagy receptors such as p62, OPTN, and NDP52) offers a plausible mechanistic basis for such spatial

activation patterns (*e.g.*, Adriaenssens E *et al.*, *Nat Struct Mol Biol* 2024 and Yamano K *et al.*, *EMBO J* 2024), additional analysis will be required to determine the precise molecular architecture underlying this organization.

For this reason, we have limited the description in the main text to the observed spatial distributions of TBK1 and pTBK1, without further mechanistic interpretation beyond what is directly supported by the data (Page 5, lines 102-104 in the revised manuscript).

Comment-3

3. TBK1BP1 knockdown data in Fig 1e, Fig 2c, Fig 2d appears not consistent. Fig 1e. pTBK1 intensity in the quantification seems not reduced in TBK1BP1 knockdown.

Reply-3

In revised Fig 1E, the knockdown experiment was performed for *TBK1BP1*, whereas the experiments shown in revised Fig 2C and 2D were conducted using *TAX1BP1* (not ***TBK1BP1***) knockdown cells.

Comment-4

4. Fig 3 - this is an interesting part of the story. It suggests that PPP2R5A, B, E contributes to dephosphorylation of pS403. The loading levels are heterogenous in Fig 3b.

Reply-4

We performed three independent experiments and quantified the band intensities. The results are presented as a quantitative graph in revised Fig 3B.

Comment-5

5. In the middle-panel images with mCherry-KEAP1 in Fig 4f, the two p62 bodies in the cell at the lowest position, where no KEAP1 signals can be seen, colocalize with PPP2R5E. This raises a question whether KEAP1 is genuinely required for PPP2R5E localization in p62 bodies.

Reply-5

The images in original Fig 4F are based on overexpression and therefore do not allow definitive conclusions regarding the requirement of KEAP1 for PPP2R5E localization. To address this issue, we replaced this analysis with a more stringent experimental approach.

Specifically, we generated *PPP2R5A/B/E* triple knockout cells in which either wild-type PPP2R5E or a KEAP1-binding–deficient PPP2R5E mutant (PPP2R5E$^{Loop\ mutant}$) was knocked in, and examined their localization relative to endogenous p62 and KEAP1. As shown in revised Fig 4G, the PPP2R5E$^{Loop\ mutant}$ exhibited a diffuse cytoplasmic distribution and failed to localize to p62 bodies, whereas wild-type PPP2R5E robustly localized to p62 bodies. Notably, in cells expressing the PPP2R5E$^{Loop\ mutant}$, p62 bodies were smaller, yet KEAP1 colocalization with p62 bodies was still observed.

These results indicate that KEAP1 binding is required for PPP2R5E localization to p62 bodies, while KEAP1 recruitment to p62 bodies can occur independently of PPP2R5E.

Comment-6

6. In Fig 4, the direct interaction between PP2A and KEAP1 could be tested.

Reply-6

To directly test the interaction between PP2A and KEAP1, we focused on PPP2R5E, a regulatory B subunit that confers substrate specificity to the PP2A holoenzyme. We generated *PPP2R5A/B/E* triple knockout cells in which either wild-type PPP2R5E or a KEAP1-binding–deficient PPP2R5E mutant (PPP2R5E$^{Loop\ mutant}$) was knocked in. We then performed immunoprecipitation assays to examine the interaction between PPP2R5E and KEAP1. As shown in revised Fig 4F, KEAP1 co-immunoprecipitated with wild-type PPP2R5E but not with the PPP2R5E loop mutant.

Using purified recombinant proteins, high-speed AFM visualized direct binding between KEAP1 and PPP2R5E (revised Fig 4C and Movie EV3). This confirms that KEAP1 can directly engage PPP2R5E in the absence of any additional cellular factors.

Comment-7

7. S403 phosphorylation is not predicted to alter the p62-LC3 interaction. Why the number of LC3-positive structures per p62 body volume is elevated by S403 phosphorylation (Fig 5f)?

Reply-7

We agree that phosphorylation at S403 is not predicted to directly alter the p62−LC3 interaction. Instead, our data indicate that S403 phosphorylation reduces the size of p62 bodies (revised Fig 5). As a result, the surface-area−to-volume ratio of p62 bodies increases, providing more accessible interface for isolation-membrane engagement. This geometric effect likely explains

the increased number of LC3-positive structures per p62-body volume observed in revised Fig 6C.

This concept is described in the Discussion, where we propose that size reduction increases the surface-to-volume ratio and improves compatibility with autophagosome membranes, while the gel-like transition enhances mechanical stability for isolation-membrane nucleation and expansion (Page 15, lines 446-460).

Comment-8

8. In Fig 6b, the FRAP signal difference between WT p62, p62 S403A and p62 S430E is modest.

Reply-8

The issues raised regarding initial intensity offsets, acquisition noise, potential multi-spot bleaching, and global photobleaching were addressed by reprocessing the raw FRAP data and clarifying the acquisition and analysis workflow. Briefly, background correction was applied prior to normalization, resulting in consistent initial intensities across conditions, and the revised recovery curves support our original conclusion.

A detailed description of the corrected FRAP workflow and validation is provided in our response to Reviewer 3, Comment 2, and the corresponding updates are described in the revised Methods.

In the revised manuscript, the original Fig 6B is now presented as revised Fig EV4B.

Comment-9

9. In Fig 6, it is important to experiment in vitro LLPS of WT p62, S403A P62, S403E P62.

Reply-9

We appreciate the reviewer's suggestion. We have performed *in vitro* LLPS assays using purified WT, S403A, and S403E p62. However, we found that the material properties observed *in vitro* do not reflect the *in vivo* material state of p62 bodies, primarily due to fundamental differences in ubiquitin chain architecture.

In standard *in vitro* assays using linear 8× ubiquitin, S403E p62 formed large, highly spherical droplets, which contrasts with the smaller and more gel-like p62 bodies observed *in vivo*. UB-AQUA analysis of purified p62 bodies from cells revealed that endogenous ubiquitin chains are predominantly K48–K63 branched chains, whereas linear ubiquitin is scarce (unpublished

data). Such branched architectures are expected to strongly influence intermolecular crosslinking and condensate rheology and cannot be recapitulated by linear ubiquitin reagents. Consistently, using our recently developed LLPS assay, we observed that p62 undergoes LLPS with K63-linked ubiquitin but not with K48-linked chains, suggesting that p62 condensation is initially driven by K63-linked ubiquitin and subsequently modulated by K48–K63 branching *in vivo*.

Because physiologically relevant ubiquitin chains in p62 bodies are branched rather than linear, we believe that *in vitro* LLPS assays using linear ubiquitin do not provide a biologically meaningful context for evaluating material-state differences among WT, S403A, and S403E p62. Reconstitution with branched ubiquitin chains will be required to faithfully model endogenous p62 body properties; however, such approaches are currently under development and lie beyond the scope of the present study.

Comment-10

10. In Fig 6d and g, rigorously, a transfection efficiency control (such as a GFP plasmid) could be included.

Reply-10

The experiments shown in original Fig 6D and 6G were not performed using transient transfection but rather using stable cell lines generated by retroviral transduction. Therefore, variations in transfection efficiency are not applicable to these experiments.

In the revised manuscript, we consistently used stable expression systems, knock-in cell lines, and knock-in mouse models to minimize potential artifacts associated with overexpression. In addition, the original Fig 6D and 6G are now presented as revised Fig. EV4E and revised Fig 7D, respectively.

Reviewer #2 (Remarks to the Author):

General comment

In this manuscript, Komatsu and colleagues propose that TBK1 and PP2A are recruited to p62/SQSTM1 condensates, where these proteins play opposing roles in the control of the phosphorylation status of S403 on p62. This phosphorylation controls the size and the dynamics of p62 bodies and enhance their clearance via the autophagy machinery. Overall, these studies provide important new insight into autophagic clearance of p62 bodies and the role of the phosphorylation at Ser403, which is proposed to act as a tunable rheostat in the control of p62

body size and turnover.

Although the experiments are well-done and the results are generally supportive of this model, the authors also propose KEAP1 as a bridge that recruits p62 bodies; moreover, the results in Figure 4 suggest an interdependence between PP2A subunits and KEAP1 in the control of p62 body size and dynamics. As KEAP1 is an E3 ubiquitin ligase, it is not completely clear whether the changes in p62 body size and dynamics are directed by Ser403 phosphorylation or a more direct effect of KEAP1-mediated ubiquitination at these complexes.

Comment-1

1) For the in vitro reconstitution assays in Fig. 1d and S1a, quantification of TBK1 incorporation into the p62 bodies should be performed, given the conclusions that are being made with regard to TBK1. Also, it will be helpful to include all of the conditions and images from those two panels into a single panel, since all of the comparisons in S1a are being made to the top row of Fig. 1d, forcing the reader to toggle back and forth.

Reply-1

In the *in vitro* LLPS assay, TBK1 localization to p62 bodies was strictly dependent on the presence of all components, including 8× ubiquitin, p62, NBR1, TAX1BP1, and AZI2/TBK1BP1. Omission of any single component abolished TBK1 colocalization with p62 bodies (revised Fig 1D).

As suggested by the reviewer, we have now merged the conditions previously displayed in Fig. 1D and Supplementary Fig. S1A into a single consolidated panel (revised Fig 1D) to improve clarity and avoid forcing the reader to toggle between two figures.

Our conclusions regarding TBK1 recruitment to p62 bodies are primarily based on cell-based analyses. Specifically, TBK1 localization to p62 bodies was quantitatively evaluated using knockdown of individual factors (revised Fig 1E), and the spatial distribution and quantification of p62 S403 phosphorylation were analyzed in cells (revised Fig 2C and 2D).

Comment-2

2) Overall, the proposed model does not disentangle the effects of S403 phosphorylation vs. ubiquitination at p62 bodies due to the presence of E3 ligases such as KEAP1. The results in Fig 4e-g point to a role for KEAP1 in the recruitment of PPP2R5 subunits to p62 bodies. At the same time, KEAP1 knockdown or knockout causes a decrease in the size of p62 bodies. Although this attributed to changes in the S403 phosphorylation status, these KEAP1

interactions with p62 may more directly impact the amount of ubiquitin present at p62 bodies. If so, how does contribute to their size? Similarly, does triple knockdown of PPP2R5 subunits impact the amount of ubiquitin present in these p62 condensates?

Reply-2

We thank the reviewer for raising this important point. We agree that it is essential to distinguish between a potential “bridging” function of KEAP1 and a role of KEAP1 as an E3 ubiquitin ligase at p62 bodies.

i. p62-body purification and quantitative proteomics argue against KEAP1 E3 ligase activity within p62 bodies.

   Using our established method for p62-body purification (Kurusu R *et al*., *Dev Cell* 2023), we performed quantitative mass spectrometry to determine the composition of endogenous p62 bodies (revised Fig EV3). While KEAP1 was detected at high abundance, the essential E3 ligase component RBX1 was not detected. If KEAP1 functioned as an active CUL3–KEAP1–RBX1 E3 ligase within p62 bodies, RBX1 would be expected to be present at near-stoichiometric levels relative to KEAP1. Its absence strongly suggests that KEAP1 does not act as an E3 ligase in p62 bodies.

ii. KEAP1 directly binds PPP2R5E independently of ubiquitination.

   Using high-speed AFM (revised Fig 4C) and endogenous immunoprecipitation from *PPP2R5E* knock-in cells (revised Fig 4F), we detected a direct interaction between KEAP1 and PPP2R5E. Importantly, this interaction was abolished by a PPP2R5E$^{\text{Loop mutant}}$ that is defective in KEAP1 binding (revised Fig 4G). These results indicate that KEAP1 functions as a recruiter of PP2A regulatory subunits rather than as an E3 ligase in this context.

iii. Implications for p62-body size and ubiquitin content.

   Taken together, these findings suggest that the effects of KEAP1 depletion on p62-body size and dynamics are best explained by impaired PP2A recruitment and consequent alterations in p62 Ser403 phosphorylation, rather than by changes in ubiquitin deposition mediated by KEAP1. Accordingly, the reduction in p62-body size observed upon KEAP1 knockdown or knockout reflects altered phosphorylation-dependent material properties rather than a direct reduction in ubiquitin content.

Comment-3

3) Since KEAP1 is proposed to act as a molecular bridge to recruit PP2A, does endogenous

KEAP1 localize to p62 bodies in control cells? Only overexpressed mCherry-tagged constructs are shown in Fig 4f. KEAP1 localization to p62 bodies has primarily been described in the context of autophagy inhibition or oxidative stress. Is there recruitment of this protein to p62 complexes in the absence of stressors? Furthermore, what is the effect of PPP2R5 triple knockdown on KEAP1 localization to p62 bodies?

Reply-3

Endogenous KEAP1 colocalization with p62 bodies under non-stressed conditions is shown in revised Fig 4G. In *PPP2R5A/B/E* triple-knockout cells, p62 bodies are reduced in size; however, endogenous KEAP1 remains detectable on these smaller p62 bodies, indicating that PP2A regulatory subunits are not required for KEAP1 recruitment.

Under stress conditions, phosphorylation of p62 at Ser403 is markedly increased (revised Fig 7A and 7B). Although additional work will be required to define how stress-dependent signaling pathways influence KEAP1 association with p62 bodies, such analyses are beyond the scope of the present study.

Comment-4

4) In Fig 5e, what is meant by authors' statement that the p62 body is "segmented by isolation membranes." An isolation membrane is not apparent in the image provided, possibly due to the fixation required for immuno-EM. Are the authors referring to the smaller condensate in the bottom left of the image that appears broken off? Clarification and labels (arrows) in the image are needed.

Reply-4

As the reviewer correctly notes, membrane morphology is not optimally preserved under the fixation conditions required for immuno-electron microscopy. In such preparations, isolation membranes frequently appear electron-lucent due to widening of the space between the inner and outer membranes, a characteristic feature of immuno-EM−processed samples.

To clarify our interpretation, we have added an arrow in the revised Fig 6B to indicate the isolation-membrane−like structure in the lower-left region of the image and have revised the corresponding figure legend for improved clarity.

Comment-5

5) In Fig 6a-b, it is curious that S403A does not cause any increase in p62 body size or any

significant change in the FRAP dynamics compared to wild type p62. Is there a threshold effect for unphosphorylated S403 on p62 body size? The authors may wish to provide some discussion.

Reply-5

We appreciate the reviewer's insightful comment. The lack of a detectable increase in p62-body size (revised Fig EV4A) or any significant change in FRAP dynamics (revised Fig. EV4B) in the S403A mutant suggests that the absence of Ser403 phosphorylation alone is not sufficient to further stabilize or enlarge p62 bodies beyond the basal state observed for wild-type p62.

We propose that p62-body properties are regulated in a non-linear manner, with Ser403 phosphorylation acting in a switch-like or threshold-dependent manner rather than as a graded determinant. In this model, basal p62 bodies formed by unphosphorylated p62 reside below the threshold required for a material-state transition; thus, preventing phosphorylation (S403A) does not markedly affect size or dynamics. In contrast, phosphorylation at Ser403 (as mimicked by S403E) drives p62 bodies across this threshold, leading to reduced size and altered material properties. This interpretation is consistent with our data showing that Ser403 phosphorylation induces qualitative rather than incremental changes in p62-body organization. We discussed these points in the discussion section of the revised manuscript (page 15, lines 438-445).

Comment-6

6) What is the effect of S430A and S403E on KEAP1 recruitment to p62 bodies compared to controls?

Reply-6

We thank the reviewer for this question. We did not directly examine KEAP1 recruitment in cells expressing S403A or S403E mutants. However, our available data indicate that Ser403 phosphorylation does not influence KEAP1 localization to p62 bodies.

In *PPP2R5A/B/E* triple-knockout cells, where Ser403 phosphorylation is markedly elevated, endogenous KEAP1 remains robustly localized to p62 bodies (revised Fig 4G). This demonstrates that KEAP1 recruitment is maintained even under conditions of enhanced Ser403 phosphorylation.

Furthermore, in the same experiment, expression of a PPP2R5E$^{\text{Loop mutant}}$, which cannot bind KEAP1, did not alter KEAP1 localization to p62 bodies, indicating that KEAP1 recruitment is independent of PP2A regulatory subunit binding.

These observations, together with extensive prior evidence that KEAP1 binding to p62 is mediated by the Keap1-interacting region (Ser349-dependent) and not by Ser403 (Ikeda R *et al.*, *EMBO J* 2023), support the conclusion that neither S403A nor S403E is expected to directly affect KEAP1 recruitment. Accordingly, the effects of Ser403 phosphorylation described in this study are best explained by altered PP2A recruitment and phosphorylation-dependent changes in condensate material properties, rather than by modulation of KEAP1 binding.

Minor comment-1

1) In Fig 3f, the loss of TBK1 reduces but does not "abolish" the effects of triple PPP2R5 isoform knockdown on pS403 levels, suggesting other kinases may compensate for TBK1 in the control of p62 bodies. Although further experiments on this is beyond the scope of this study, the conclusion suggesting TBK1 as the sole kinase should be tempered.

Reply-1

We agree with the reviewer that the loss of TBK1 reduces, but does not completely abolish, the effects of *PPP2R5A/B/E* triple knockdown on p62 Ser403 phosphorylation (revised Fig 3F), indicating that additional kinases may partially compensate for TBK1 in this context. We therefore do not intend to imply that TBK1 is the sole kinase regulating p62 bodies (Page 7, lines 176-180 in the revised manuscript).

Reviewer #3 (Remarks to the Author):

General comment

The paper investigates the regulation and key regulators of P62 phosphorylation at Ser403, as well as the impact of this modification on the organization, material properties, and function of P62 bodies in autophagy. The work includes a diverse range of complementary microscopy-based methodologies, including confocal, super-resolution, correlative light and electron microscopy, and FRAP, as well as basic image analysis and quantification. The main findings include the localization of Ser403 regulators and downstream autophagy machinery to substructures within P62 bodies and associated phenotypic changes in P62 bodies fluidity and size distribution.

Overall, the paper tackles many important aspects of p62 body physiology and displays rich high-level microscopy data to address them. Yet, despite the substantial experimental work, I find many of the observations incomplete and overinterpreted, primarily concerning the

biophysical and material properties analysis. Furthermore, the manuscript frequently blurs the line between novel findings and confirmatory data, leaving the reader uncertain about what is being claimed as new.

Major comments

Comment-1

1) Mathematical model and Oswald ripening – The authors claim (lines 217–219) that "when intermolecular interactions are weak, droplets undergo Ostwald ripening and gradually coarsen into large droplets," based on their mathematical modeling (Figure 5A) and phenotypic observations under PPP2R5 knockdown. However, I find both the theoretical and experimental basis for this conclusion problematic.

In general, weaker intermolecular interactions are associated with reduced surface tension, which in soft-matter systems tends to suppress Ostwald ripening rather than promote it. The simulation appears to support the opposite behavior, likely due to the use of parameter values more appropriate for highly arrested systems—specifically, those involving strong, aggregation-prone, or covalent-like interactions that restrict internal mobility and inhibit material exchange. These assumptions however are inconsistent with the known behavior of P62 bodies, which retain substantial internal dynamics, as demonstrated by the FRAP data (Figure 5C) and supported by previous literature. As a result, the simulations do not accurately represent the physical properties of the system and are misleading. On top of that, the minimal snapshots that the authors present, the lack of time-dependent analysis, given the minimal caption of Figure 5A, which only states "computational simulations" without any description of the results, assumptions, or parameters, adds little to the readers ability to interpret the analysis and gives the impression of a superficial minimally explained modeling effort. Also important to point out that while the main driver of Oswald ripening is the reduction of surface area, the nonspherical shapes predicted by the model are puzzling.

Experimentally, the data also do not support the interpretation of Ostwald ripening. Under PPP2R5 knockdown, the authors observe a global reduction in P62 body size, but Ostwald ripening is characterized by the selective growth of larger droplets at the expense of smaller ones—not uniform size reduction. The observed phenotype could be explained by simply the lack of PPP2R5 localization, which decreases the biomolecular mass localized to P62 bodies, or could reflect a general dissolution of P62 bodies. Can the authors provide evidence that

these aren't the cases? Furthermore, the authors do not report the number of droplets or track their evolution over time - both could shed light on the mechanism. Time-lapse imaging to follow individual droplets and assess their size trajectories would be necessary to validate or reject a ripening-based mechanism.

Reply-1

We revised both the conceptual framework and the mathematical modeling in response to the reviewer's comments.

(1) Clarification of the physical assumptions and experimental constraints

We first clarified our working hypothesis: phosphorylation-dependent changes in p62−ubiquitin interactions alter the material properties of p62 condensates. To constrain the model experimentally, we incorporated quantitative proteomic data from purified endogenous p62 bodies, which demonstrate that p62 and ubiquitin are the dominant components, whereas other associated proteins (including KEAP1) are present at molar abundances 2−3 orders of magnitude lower (revised Fig EV3). Although LFQ intensities (~60% of total signal) reflect mass contribution, molar abundance analysis supports a simplified two-component p62−ubiquitin physical system, justifying the revised modeling approach.

(2) Reformulation of the mathematical model

We completely reformulated the model using a Flory−Huggins free-energy description coupled to Cahn−Hilliard dynamics with thermal fluctuations (revised Fig 5A). The revised framework incorporates:

- symmetric mixing free energy,
- a physically appropriate interfacial energy term $\frac{b}{2}(\nabla\phi)^2$,
- concentration-dependent mobility $L(\phi)$, and
- a stochastic flux term satisfying the fluctuation−dissipation theorem.

This formulation is consistent with a dynamically exchanging condensate and aligns with the substantial internal mobility observed experimentally by FRAP.

(3) Removal of the original Ostwald−ripening interpretation

In light of the reviewer's critique, we removed the original interpretation linking the *PPP2R5* knockdown phenotype to Ostwald ripening. While the revised model can exhibit coarsening behavior in certain parameter regimes, we no longer use it to argue for a ripening-based mechanism in p62 bodies. Instead, we now employ the model conceptually to illustrate how changes in intermolecular interaction strength qualitatively influence condensate dynamics (Pages 14-15, lines 427-438 in the revised manuscript).

(4) Experimental alignment and revised interpretation

Experimentally, *PPP2R5* depletion reduces p62-body fluidity (FRAP), which correlates with smaller condensates. In the revised simulations, the tighter-interaction condition yields lower effective mobility L, and condensate growth is limited over the simulated time window. This qualitative behavior matches the experimental phenotype.

Accordingly, we have revised our conclusions: we no longer claim that weak interactions promote Ostwald ripening. Rather, the behavior is more accurately described as mobility-limited coarsening. We explicitly acknowledge that interfacial tension (γ) can promote coarsening; however, although γ-dependent energetics are included in the framework, coarsening remains suppressed when transport (mobility) is reduced. Thus, although a larger concentration difference can increase γ and favor coarsening, the strong reduction in mobility observed under the tighter-interaction condition dominates in this parameter regime. The observed behavior reflects a balance between interfacial driving forces and mobility constraints, with reduced mobility limiting condensate growth within the simulated time window. This interpretation is fully consistent with the experimental phenotype and supports the use of the present model and parameter set as a conceptual tool for understanding p62-condensate dynamics.

Comment-2

2) There are several issues with the experimental execution, data analysis, data presentation, and interpretation of the FRAP results in figure 5C and 6b. Firstly, while the mean recovery of the blue curve in fig 5C appears lower than that of the black, a closer look shows that the blue curve starts from a lower initial intensity (~10–15% lower), suggesting that a greater portion of the P62 body, including its immobile fraction, was photobleached on average. This of course, can happen, but without normalizing it, fitting, and extrapolating a recovery rate, it undermines the comparison and casts doubt on the conclusion that the NT assemblies are more dynamic

and more liquid-like.

Moreover, the data curves themselves appear surprisingly noisy given the number of assemblies analyzed. This raises concerns about how the measurements were acquired. One possibility is that photobleaching was performed simultaneously on multiple assemblies within the same cell, making all recovery curves susceptible to fluctuations in imaging conditions, such as light intensity or focus drift (the time-lapse shown below the curves, show a clear shift in focus at t = 309). If recovery was not normalized to total cell fluorescence or background, such artifacts could significantly distort the data - Figure 6C further supports this concern, as the area surrounding the photobleached assembly shows a significant drop in fluorescence even at t=1177s, indicating that the entire cell may have experienced significant photobleaching. In addition, the presentation of statistical variation is inconsistent: the FRAP recovery curves show symmetric error bars, whereas the mobile fraction analysis includes asymmetric outlier bars, which is difficult to reconcile without further clarification. Altogether, the quality of data analysis is insufficient to support the authors conclusions. The differences in trendlines and calculated t1/2 values between the conditions appear minimal – particularly in 5C. As such, I find the evidence for altered liquidity between the two conditions to be weak and not convincingly demonstrated.

Reply-2

We thank the reviewer for pointing out the difference in initial post-bleach intensities in the original Fig 5C. We reprocessed all raw FRAP datasets by applying background correction prior to normalization and repeated the entire analysis. With this corrected workflow, the initial fluorescence values are now consistent across all conditions, indicating that the previous offset resulted from incomplete background subtraction.

Importantly, the revised analysis incorporates background subtraction and normalization at every time point, thereby minimizing potential effects of acquisition noise, focus drift, and global photobleaching. As an additional control, we confirmed that the fluorescence intensity in non-bleached regions remained stable relative to the normalized prebleach baseline, excluding whole-cell photobleaching as a confounding factor. The revised recovery curves and updated mobile-fraction values (revised Fig 5C and revised Fig EV4B) support our original conclusion.

Regarding the reviewer's concern about multi-spot bleaching, all FRAP experiments were performed under the imaging conditions recommended for the FV3000 system, including Z-drift compensation and minimal laser exposure outside the bleach ROI. We note that the

revised curves display substantially reduced noise, consistent with proper normalization and acquisition-stability correction.

The updated analysis workflow is now fully described in the revised Methods section and is consistent with FRAP procedures reported for the same microscope system (Yang Z *et al.*, *EMBO J* 2024).

Smaller points:

Minor comment-1

• *Terminology - The manuscript uses several non-standard, ambiguous, and sometimes confusing terms that hinder clarity. For instance, using miniaturization instead of simply stating decrease in size. In the case of a complex structure its important cause the authors don't show any proof that the substructures within P62 drop in similar proportions. Another example is piecemealed. Firstly, the authors only show stagnant images of multiple LC3 substructures within an individual body without any time-dependence. Do the authors show that it leads to size reduction? Is the entire P62 body decomposed in a piecemeal manner? Do the authors show that? Another nonstandard term is Tunable rheostat, which in my opinion is unnecessary.*

Reply-1

We thank the reviewer for this helpful comment. In accordance with the suggestion, we have revised the manuscript to replace non-standard or potentially ambiguous terms with clearer and more conventional terminology. Specifically, we now use expressions such as "decrease in size" instead of "miniaturization," avoid the term "piecemealed," and removed the phrase "tunable rheostat." These revisions improve clarity without altering the scientific conclusions.

Comment-2

• *Unclear distinction throughout the manuscript between original findings and validations. For instance, TBK1 recruitment and activation in P62, involvement of TBK1BP1 in its activation have been described.*

Reply-2

We thank the reviewer for raising this important point. We agree that several components of the selective-autophagy machinery—including p62/SQSTM1, NBR1, TAX1BP1, and the TBK1 adaptor family (AZI2/NAP1 and TBK1BP1/SINTBAD)—have been implicated in cargo recognition and upstream signaling in prior studies (*e.g.*, Turco E *et al.*, *Nat Commun* 2021,

Bauer B *et al.*, *EMBO J* 2024, North BJ *et al.*, *J Cell Biol* 2025). These studies established that these receptors and adaptors cooperate in aggrephagy initiation or TBK1 activation in other cellular contexts.

However, none of these reports defined how TBK1 is recruited into p62 condensates or how TBK1 becomes spatially organized and activated within these phase-separated structures. In particular, previous work did not address:

· the sequential AZI2–TAX1BP1–NBR1–p62 adaptor pathway that we reconstitute and show to be necessary for TBK1 incorporation;

· the spatial architecture in which TBK1 resides in the condensate interior while pTBK1 segregates to the periphery;

· the functional coupling between TBK1 recruitment, p62 Ser403 phosphorylation, and the phosphorylation-dependent material-state transition of p62 bodies;

· the integration of PP2A regulatory subunits into the same condensates to form a local kinase–phosphatase module that tunes condensate degradability.

Thus, although individual interactions among these proteins have been described previously, their coordinated assembly and mechanistic function within p62 condensates have not, to our knowledge, been demonstrated. We have revised the Introduction and Discussion to better distinguish between elements supported by prior literature and the mechanistic insights that are unique to this study.

Comment-3

• Figure 5D-E – Fluorescent and EM images beautifully show a P62 body. However, it remains unclear whether the same structures are being visualized in both modalities. Specifically, the EM substructure shown appears devoid of P62 antibody labeling, whereas the fluorescence images show multiple such structures in a single P62 assembly and more importantly, they are strongly enriched in P62. Without additional markers—such as LC3 antibodies—to confirm the identity of the EM-resolved structures, it is difficult to conclude with confidence that these substructures are of the same type.

In addition, the scale bars in 5D are partially missing and perhaps somewhat inconsistent. The upper panels have no scale bars, and the lower panels, which represent an image and an enlarged zoomed-in image, have two similar 1 um. Based on the dimensionality I guess the enlarged one should represent roughly 400 nm. This is nearly the size of the entire assembly shown in the EM image and much larger than the internal substructure.

Reply-3

We thank the reviewer for this helpful comment. Under standard immuno-electron microscopy conditions (revised Fig 6B), membrane morphology is not optimally preserved, and isolation membranes frequently appear electron-lucent due to widening of the space between the inner and outer membranes—a well-established characteristic of samples processed for immuno-EM.

To address the reviewer's concern regarding the correspondence between fluorescence and EM structures, we note that LC3–p62 double labeling was performed independently in both the STED analysis (revised Fig 6A) and the FIB–SEM CLEM analysis (revised Fig 6D). These multimodal datasets confirm that the structures observed by EM represent LC3- and p62-positive assemblies.

In addition, in response to the comment about scale bars, we have added the missing scale information to the revised STED images (scale bars: 1 μm; enlarged views: 100 nm), ensuring consistency and clarity across imaging modalities.

Taken together, these considerations support the conclusion that the structures visualized in fluorescence and EM represent the same p62- and LC3-positive assemblies.

Comment-4

• *Quantification of Fig. 5f – Firstly, I don't see the point of the graph to the right because the data clearly shows that there are two subpopulations with different characteristic sizes, where the larger ones are absent from the si cells. Interpreting it as causing a reduction in mean size is unsupported by the data. It's more likely that the formation of large assemblies is inhibited.*

Reply-4

We thank the reviewer for pointing out that the size distribution contains two subpopulations and that the loss of larger assemblies in si-*PPP2R5s* cells complicates interpretation of mean size values. Because the goal of this analysis was not to evaluate mean p62-body size but rather to examine how condensate size relates to the density of LC3-positive substructures, we have removed the mean-size panel from the revised figure. The revised Fig 6F now focuses solely on the volume-normalized LC3 counts, which most directly reflect the intended biological interpretation.

Summary evaluation

The manuscript raises concerns regarding the interpretation of modeling results, the robustness and presentation of FRAP data, and the overall support for key claims about P62 body

dynamics and size reduction. I recommend that the authors substantially revise and clarify their analyses, apply more rigorous quantitative methods where appropriate, and more clearly distinguish between speculative interpretations and conclusions directly supported by experimental evidence.

Reply-summary evaluation

We appreciate the reviewer's overall assessment and agree with the concerns raised. In response, we have substantially revised the manuscript to clarify the interpretation of the modeling results, strengthen the robustness and presentation of the FRAP analyses, and more clearly distinguish between conclusions directly supported by experimental evidence and more speculative interpretations.

Importantly, we implemented three major revisions to directly address these points:

1. Mathematical model reformulation:

We completely reformulated the modeling framework using a Flory–Huggins free-energy description coupled to Cahn–Hilliard dynamics with thermal fluctuations (revised Fig 5A). This physically grounded formulation resolves the conceptual and interpretational issues associated with the previous minimal model and aligns with the experimentally observed condensate behavior.

2. Comprehensive FRAP reanalysis:

We reprocessed all raw FRAP datasets by applying background correction prior to normalization and repeated the analyses (revised Fig 5C and Fig. EV4B). With this corrected workflow, the initial fluorescence values are now consistent across conditions, confirming that the previous offset arose from incomplete background subtraction. The kinetic differences between conditions remain robust.

3. Direct measurement of material-state parameters:

Using thermal contour-fluctuation analysis, we quantified the effective surface tension (γ) of purified p62 bodies (revised Fig 5D and Fig EV4C). Both *PPP2R5s* knockdown and the phospho-mimetic S403E mutant increased γ, demonstrating a transition toward a more compact, gel-like material state. This behavior is incompatible with condensate dissolution and directly supports our interpretation that Ser403 phosphorylation stabilizes p62 condensates and limits their dynamics.

Together, these revisions substantially strengthen the rigor, clarity, and quantitative support for our conclusions regarding p62 condensate dynamics and size regulation.

Dear Masaaki,

This is a short note to let you know that the arbitrating referee is satisfied with your point-by-point responses, and sees the article as suitable for publication (see below). I will write again shortly with a list of minor editorial points that should be addressed.

Best wishes,

William

William Teale, PhD
Editor
The EMBO Journal
w.teale@embojournal.org

Instructions for preparing your revised manuscript:

Read our guidance for manuscript revisions and related editorial policies: https://link.springer.com/journal/44318/submission-guidelines#cms-Revised-submissions

Please also check that the title and abstract of the manuscript are brief, yet explicit, even to non-specialists.

When assembling figures, please refer to our figure preparation guideline in order to ensure proper formatting and readability in print as well as on screen:
https://media.springernature.com/original/springer-cms/rest/v1/content/27825798/data/v1

IMPORTANT: When you send the revision we will require
- a point-by-point response to the referees' comments, with a detailed description of the changes made (as a word file).
- a word file of the manuscript text.
- individual production quality figure files (one file per figure)
- a complete author checklist
- Expanded View files (replacing Supplementary Information)
- a Reagents and Tools Table as part of the Methods section

Please remember: Digital image enhancement is acceptable practice, as long as it accurately represents the original data and conforms to community standards. If a figure has been subjected to significant electronic manipulation, this must be noted in the figure legend or in the 'Methods' section. The editors reserve the right to request original versions of figures and the original images that were used to assemble the figure.

We realize that it is difficult to revise to a specific deadline. In the interest of protecting the conceptual advance provided by the work, we recommend a revision within 3 months (16th Jun 2026). Please discuss the revision progress ahead of this time with the editor if you require more time to complete the revisions. Use the link below to submit your revision:

https://emboj.msubmit.net/cgi-bin/main.plex

Referee #1:

The provided point-by-point response adequately addresses and clarifies the reviewers' concerns. In my opinion, the manuscript has been sufficiently improved to warrant publication.

Response to editorial points

We thank the editor for the careful review and constructive guidance. We have addressed all editorial points as detailed below.

1. Data Availability Statements

Comment:

Combine the Data Availability Statements into a single section.

Response:

We have consolidated all Data Availability information into a single section in the revised manuscript (Page 31, lines 951–957).

2. Conflict of Interests

Comment:

Change the title of the 'Conflict of Interests' statement to the 'Disclosure and Competing Interests Statement',

Response:

The section title has been revised accordingly (Page 32, lines 971–972).

3. Author contributions

Comment:

Remove the author contributions section from the manuscript.

Response:

The author contributions section has been removed from the manuscript.

4. "data not shown"

Comment:

Replace references to 'data not shown'; these may either be with 'unpublished observations' together with the name of the person who made the observation in brackets, or by showing the data themselves in an appendix figure.

Response:

The single instance of “data not shown” has been replaced with “unpublished observations,” including attribution (Page 7, lines 184–185).

5. Funding discrepancy (JP25H00315)

Comment:

Ensure that the funding listed in the Acknowledgments section matches the separate entries in our online submission system; there is a discrepancy for the MEXT grant JP25H00315.

Response:

The funding information has been corrected to match the entries in the online submission system, including the MEXT grant JP25H00315 (Page 31, line 962).

6. Figure files

Comment:

Upload main and EV figures as separate production quality Figure files (using the nomenclature Figure 1, etc. Figure EV1, etc.).

Response:

All main figures and Figure EVs have been uploaded as separate production-quality files with appropriate nomenclature.

7. Figure 7H callout

Comment:

Include a callout for Figure 7H.

Response:

A callout for Figure 7H has been added in the main text (Page 13, line 388).

8. Appendix author list

Comment:

Remove the author list from the first page of the appendix

Response:

The author list has been removed from the appendix.

9. Source data

Comment:

Provide source data, one zipped file for each figure, and one for the supplementary figures.

Response:

Source data have been provided as one zipped file per figure and one for the Figure EVs.

10. Movie files

Comment:

zip movie files with their legend provided in a text file, one zip folder per movie file; the nomenclature is Movie EV1, etc.; remove movie captions from the manuscript file.

Response:

Each movie has been provided as a separate zipped file containing the video and a corresponding legend text file. Movie captions have been removed from the manuscript.

11. Methods section title

Comment:

Rename the 'Materials and Methods' section as 'Methods'.

Response:

The section has been renamed accordingly (Page 18, line 511).

12. Section order

Comment:

Use the following section order: Title page - Abstract - Introduction - Results - Discussion - Methods - Data Availability - Acknowledgments - Disclosure and Competing Interests Statement - References - Figure Legends - (Main Tables with legends if applicable) - Expanded View Figure Legends.

Response:

The manuscript has been reorganized to match the requested section order.

13. Dataset availability

Comment:

Ensure datasets PXD074538, 53579 are publicly available, and provide URLs in the Data Availability section.

Response:

Datasets PXD074538 and 53579 have been made publicly available, and their URLs have been included in the Data Availability section (Page 31, lines 951–957).

14. Exact P values

Comment:

Provide exact p values in the legends of figures 1E, 2D, 4G, 5B, EV4 A.

Response:

Exact P values have been included directly in the figures. For values below the detection limit of the software, $P < 1 \times 10^{-15}$ is reported. This convention is also stated in the figure legends (Fig 1E; 2D; 4G; 5B; and Fig EV4A) and the Methods section (Page 31, lines 942–943) for clarity and consistency.

15. Statistical tests

Comment:

Indicate the statistical test used for data analysis in the legends of figures 3A, B, E, F; EV3 A, EV4 C.

Response:

Statistical tests have been explicitly specified in each of the relevant figure legends (Fig 3A, B, E, F; Fig EV3A; and EV4C), as requested.

16. Box plot definitions (Figures 2,3,4,5,EV)

Comment:

Define box plots in terms of minima, maxima, centre, bounds of box and whiskers, and percentile in the legends of figures 2B, D; 3G, 4G, 5B, C, EV4 A, EV5 B.

Response:

Box plot definitions have been explicitly added to the relevant figure legends (Fig 2B, D; 3G; 4G; 5B, C; Fig. EV4A; Fig. EV5B), including median (centre), interquartile range (box; 25th–75th percentiles), and whiskers.

17. Box plot definitions (Figure 1E)

Comment:

Define box plots in terms of minima, maxima, centre in the legends of figures 1E.

Response:

The legend for Figure 1E has been revised to explicitly define median and minimum/maximum values.

18. n definition

Comment:

Define n in the legends of figures EV3 A, EV4 C, EV5 B.

Response:

n values have been defined in all specified figure legends (EV3A; EV4 C; and EV5 B).

19. Error bars

Comment:

Define error bars in the legends of figures 3A, B, E, F; 6E, F; 7F, H; EV4 C, E.

Response:

Error bars have been defined consistently across all relevant figure legends (Fig 3A, B, E, F; 6E, F; 7F, H; and EV4 C, E).

20. Arrowheads (Figure 1D)

Comment:

Define the white arrow heads in the legend of figure 1D.

Response:

The legend has been revised to define the white arrowheads (Page 35, lines 1149–1150).

2nd Revision - Editorial Decision **2nd Apr 2026**

Dear Masaaki,

I am pleased to inform you that your manuscript has been accepted for publication in the EMBO Journal.

Congratulations to you and all involved!

Your manuscript will be processed for publication by EMBO Press. It will be copy edited and you will receive page proofs prior to publication. Please note that you will be contacted by Springer Nature Author Services to complete licensing and payment information.

You may qualify for financial assistance for your publication charges - either via a Springer Nature fully open access agreement or an EMBO initiative. Check your eligibility: https://link.springer.com/journal/44318/how-to-publish-with-us

Should you be planning a Press Release on your article, please get in contact with embo_production@springernature.com as early as possible in order to coordinate publication and release dates.

If you have any questions, please do not hesitate to contact the Editorial Office. Thank you for your contribution to The EMBO Journal.

Best wishes,

William

William Teale, PhD
Editor
The EMBO Journal
w.teale@embojournal.org

*****
Please note that it is The EMBO Journal policy for the transcript of the editorial process (containing referee reports and your response letters) to be published as an online supplement to each paper. If you should prefer removal of any referee-only figures included in the point-by-point response(s), e.g. because they may still be used for future publication or because they have been reproduced from published work by others, please do let us know immediately via response email.
More information is available here: https://link.springer.com/partners/embo-press/editorial-policies#Peer%20review

** Click here to be directed to your login page: https://emboj.msubmit.net

EMBO Press Author Checklist

Corresponding Author Name: Masaaki Komatsu
Journal Submitted to: The EMBO Journal
Manuscript Number: EMBOJ-2025-123399R1

USEFUL LINKS FOR COMPLETING THIS FORM
The EMBO Journal - Author Guidelines
EMBO Reports - Author Guidelines
Molecular Systems Biology - Author Guidelines
EMBO Molecular Medicine - Author Guidelines

Reporting Checklist for Life Science Articles (updated January
This checklist is adapted from Materials Design Analysis Reporting (MDAR) Checklist for Authors. MDAR establishes a minimum set of requirements in transparent reporting in the life sciences (see Statement of Task: 10.31222/osf.io/9sm4x). Please follow the journal's guidelines in preparing your
Please note that a copy of this checklist will be published alongside your article.

Abridged guidelines for figures
1. Data
The data shown in figures should satisfy the following conditions:
- the data were obtained and processed according to the field's best practice and are presented to reflect the results of the experiments in an accurate and unbiased manner.
- ideally, figure panels should include only measurements that are directly comparable to each other and obtained with the same assay.
- plots include clearly labeled error bars for independent experiments and sample sizes. Unless justified, error bars should not be shown for technical
- if n<5, the individual data points from each experiment should be plotted. Any statistical test employed should be justified.
- Source Data should be included to report the data underlying figures according to the guidelines set out in the authorship guidelines on Data

2. Captions
Each figure caption should contain the following information, for each panel where they are relevant:
- a specification of the experimental system investigated (eg cell line, species name).
- the assay(s) and method(s) used to carry out the reported observations and measurements.
- an explicit mention of the biological and chemical entity(ies) that are being measured.
- an explicit mention of the biological and chemical entity(ies) that are altered/varied/perturbed in a controlled manner.
- the exact sample size (n) for each experimental group/condition, given as a number, not a range;
- a description of the sample collection allowing the reader to understand whether the samples represent technical or biological replicates (including how many animals, litters, cultures, etc.).
- a statement of how many times the experiment shown was independently replicated in the laboratory.
- definitions of statistical methods and measures:
  - common tests, such as t-test (please specify whether paired vs. unpaired), simple χ2 tests, Wilcoxon and Mann-Whitney tests, can be unambiguously identified by name only, but more complex techniques should be described in the methods section;
  - are tests one-sided or two-sided?
  - are there adjustments for multiple comparisons?
  - exact statistical test results, e.g., P values = x but not P values < x;
  - definition of 'center values' as median or average;
  - definition of error bars as s.d. or s.e.m.

Please complete ALL of the questions below.
Select "Not Applicable" only when the requested information is not relevant for your study.

Materials

Newly Created Materials	Information included in the manuscript?	In which section is the information available? (Reagents and Tools Table, Materials and Methods, Figures, Data Availability Section)
New materials and reagents need to be available; do any restrictions apply?	Yes	Materials and Methods, and Resources table

Antibodies	Information included in the manuscript?	In which section is the information available? (Reagents and Tools Table, Materials and Methods, Figures, Data Availability Section)
For **antibodies** provide the following information:<br>- Commercial antibodies: RRID (if possible) or supplier name, catalogue number and or/clone number<br>- Non-commercial: RRID or citation	Yes	Materials and Methods, and Resources table

DNA and RNA sequences	Information included in the manuscript?	In which section is the information available? (Reagents and Tools Table, Materials and Methods, Figures, Data Availability Section)
**Short novel DNA or RNA including primers, probes**: provide the sequences.	Yes	Materials and Methods, and Resources table

Cell materials	Information included in the manuscript?	In which section is the information available? (Reagents and Tools Table, Materials and Methods, Figures, Data Availability Section)
**Cell lines:** Provide species information, strain. Provide accession number in repository **OR** supplier name, catalog number, clone number, and/**OR** RRID.	Yes	Materials and Methods, and Resources table
**Primary cultures:** Provide species, strain, sex of origin, genetic modification status.	Yes	Materials and Methods
Report if the cell lines were recently **authenticated** (e.g., by STR profiling) and tested for mycoplasma contamination.	Yes	Materials and Methods

Experimental animals	Information included in the manuscript?	In which section is the information available? (Reagents and Tools Table, Materials and Methods, Figures, Data Availability Section)
**Laboratory animals or Model organisms:** Provide species, strain, sex, age, genetic modification status. Provide accession number in repository **OR** supplier name, catalog number, clone number, **OR** RRID.	Yes	Materials and Methods, and Figure legends
**Animal observed in or captured from the field:** Provide species, sex, and age where possible.	Not Applicable	
Please detail **housing and husbandry conditions**.	Yes	Materials and Methods

Plants and microbes	Information included in the manuscript?	In which section is the information available? (Reagents and Tools Table, Materials and Methods, Figures, Data Availability Section)
**Plants:** provide species and strain, ecotype and cultivar where relevant, unique accession number if available, and source (including location for collected wild specimens).	Not Applicable	
**Microbes:** provide species and strain, unique accession number if available, and source.	Not Applicable	

Human research participants	Information included in the manuscript?	In which section is the information available? (Reagents and Tools Table, Materials and Methods, Figures, Data Availability Section)
If collected and within the bounds of privacy constraints report on age, sex and gender or ethnicity for all study participants.	Not Applicable	

Core facilities	Information included in the manuscript?	In which section is the information available? (Reagents and Tools Table, Materials and Methods, Figures, Data Availability Section)
If your work benefited from core facilities, was their service mentioned in the acknowledgments section?	Not Applicable	

Design

Study protocol	Information included in the manuscript?	In which section is the information available? (Reagents and Tools Table, Materials and Methods, Figures, Data Availability Section)
If study protocol has been **pre-registered, provide DOI in the manuscript**. For clinical trials, provide the trial registration number **OR** cite DOI.	Not Applicable	
Report the **clinical trial registration number** (at ClinicalTrials.gov or equivalent), where applicable.	Not Applicable	

Laboratory protocol	Information included in the manuscript?	In which section is the information available? (Reagents and Tools Table, Materials and Methods, Figures, Data Availability Section)
Provide DOI OR other citation details if **external detailed step-by-step protocols** are available.	Not Applicable	

Experimental study design and statistics	Information included in the manuscript?	In which section is the information available? (Reagents and Tools Table, Materials and Methods, Figures, Data Availability Section)
Include a statement about **sample size** estimate even if no statistical methods were used.	Yes	Figure legends
Were any steps taken to minimize the effects of subjective bias when allocating animals/samples to treatment (e.g. **randomization procedure**)? If yes, have they been described?	Not Applicable	
Include a statement about **blinding** even if no blinding was done.	Not Applicable	
Describe **inclusion/exclusion criteria** if samples or animals were excluded from the analysis. Were the criteria pre-established?<br><br>If sample or data points were omitted from analysis, report if this was due to attrition or intentional exclusion and provide justification.	Not Applicable	
For every figure, are **statistical tests** justified as appropriate? Do the data meet the assumptions of the tests (e.g., normal distribution)? Describe any methods used to assess it. Is there an estimate of variation within each group of data? Is the variance similar between the groups that are being statistically compared?	Yes	Materials and Methods, and Figure legends

Sample definition and in-laboratory replication	Information included in the manuscript?	In which section is the information available? (Reagents and Tools Table, Materials and Methods, Figures, Data Availability Section)
In the figure legends: state number of times the experiment was **replicated** in laboratory.	Yes	Figure legends
In the figure legends: define whether data describe **technical or biological replicates**.	Yes	Figure legends

Ethics

Ethics	Information included in the manuscript?	In which section is the information available? (Reagents and Tools Table, Materials and Methods, Figures, Data Availability Section)
Studies involving **human participants**: State details of **authority granting ethics approval** (IRB or equivalent committee(s), provide reference number for approval.	Not Applicable	
Studies involving **human participants**: Include a statement confirming that **informed consent** was obtained from all subjects and that the experiments conformed to the principles set out in the WMA Declaration of Helsinki and the Department of Health and Human Services Belmont Report.	Not Applicable	
Studies involving **human participants:** For publication of **patient photos**, include a statement confirming that consent to publish was obtained.	Not Applicable	
Studies involving experimental **animals**: State details of **authority granting ethics approval** (IRB or equivalent committee(s), provide reference number for approval. Include a statement of compliance with ethical regulations.	Yes	Materials and Methods
Studies involving **specimen and field samples:** State if relevant **permits** obtained, provide details of authority approving study; if none were required, explain why.	Not Applicable	

Dual Use Research of Concern (DURC)	Information included in the manuscript?	In which section is the information available? (Reagents and Tools Table, Materials and Methods, Figures, Data Availability Section)
Could your study fall under dual use research restrictions? Please check biosecurity documents and list of **select agents and toxins** (CDC): https://www.selectagents.gov/sat/list.htm	Not Applicable	
If you used a select agent, is the security level of the lab appropriate and reported in the manuscript?	Not Applicable	
If a study is subject to dual use research of concern regulations, is the name of the **authority granting approval and reference number** for the regulatory approval provided in the manuscript?	Not Applicable	

Reporting

The MDAR framework recommends adoption of discipline-specific guidelines, established and endorsed through community initiatives. Journals have their own policy about requiring specific guidelines and recommendations to complement MDAR.

Adherence to community standards	Information included in the manuscript?	In which section is the information available? (Reagents and Tools Table, Materials and Methods, Figures, Data Availability Section)
State if relevant guidelines or checklists (e.g., **ICMJE, MIBBI, ARRIVE, PRISMA**) have been followed or provided.	Not Applicable	
For **tumor marker prognostic studies,** we recommend that you follow the **REMARK** reporting guidelines (see link list at top right). See author guidelines, under 'Reporting Guidelines'. Please confirm you have followed these guidelines.	Not Applicable	
For **phase II and III randomized controlled trials**, please refer to the **CONSORT** flow diagram (see link list at top right) and submit the CONSORT checklist (see link list at top right) with your submission. See author guidelines, under 'Reporting Guidelines'. Please confirm you have submitted this list.	Not Applicable	

Data Availability

Data availability	Information included in the manuscript?	In which section is the information available? (Reagents and Tools Table, Materials and Methods, Figures, Data Availability Section)
Have **primary datasets** been deposited according to the journal's guidelines (see 'Data Deposition' section) and the respective accession numbers provided in the Data Availability Section?	Yes	Data Availability
Were **human clinical and genomic datasets** deposited in a public access-controlled repository in accordance to ethical obligations to the patients and to the applicable consent agreement?	Not Applicable	
Are **computational models** that are central and integral to a study available without restrictions in a machine-readable form? Were the relevant accession numbers or links provided?	Not Applicable	
If publicly available data were reused, provide the respective **data citations in the reference list**.	Not Applicable	
