# Supplementary material for: Phosphorylation tunes p62 condensates to drive autophagic degradation of ubiquitinated proteins
Source: EMBO J. 2026 May 5;45(12):4061–93. doi: 10.1038/s44318-026-00785-1 (PMC13270050; doi:10.1038/s44318-026-00785-1)
Supplement: Supplementary file 17 — Figures EV1-EV5 Source Data [file 44318_2026_785_MOESM17_ESM.zip › Source Data for Expanded View and Appendix/Figure EV4 Source Data.zip!/Figure EV4 Source Data/EV4E/EV4E.pdf]

Figure EV4E

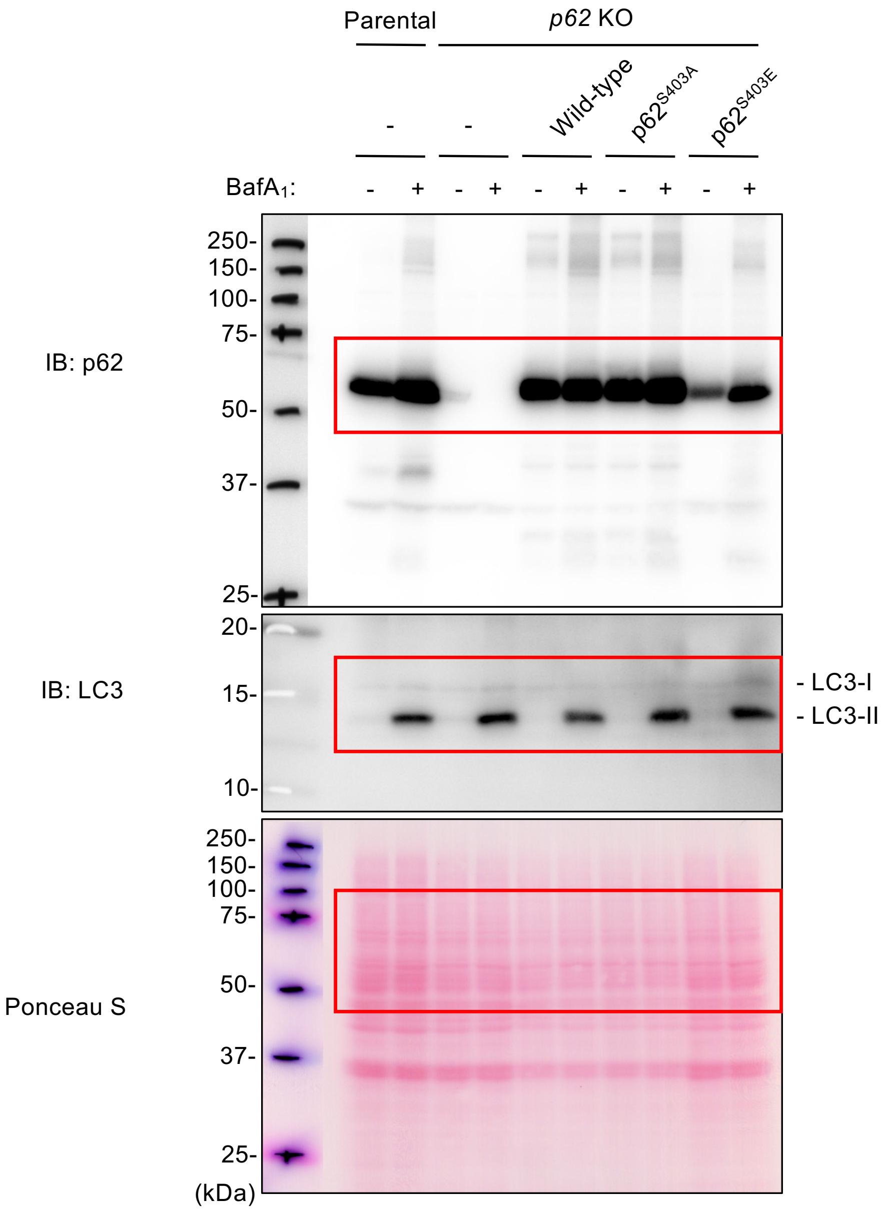
Parental
p62 KO
-
-
Wild-type
p62S403A
p62S403E
BafA1:
- + - + - + - + - +
IB: p62
250-
150-
100-
75-
50-
37-
25-
IB: LC3
20-
15-
10-
- LC3-I
- LC3-II
Ponceau S
250-
150-
100-
75-
50-
37-
25-
(kDa)
