# Supplementary material for: Phosphorylation tunes p62 condensates to drive autophagic degradation of ubiquitinated proteins
Source: EMBO J. 2026 May 5;45(12):4061–93. doi: 10.1038/s44318-026-00785-1 (PMC13270050; doi:10.1038/s44318-026-00785-1)

# Appendix Figure S1B

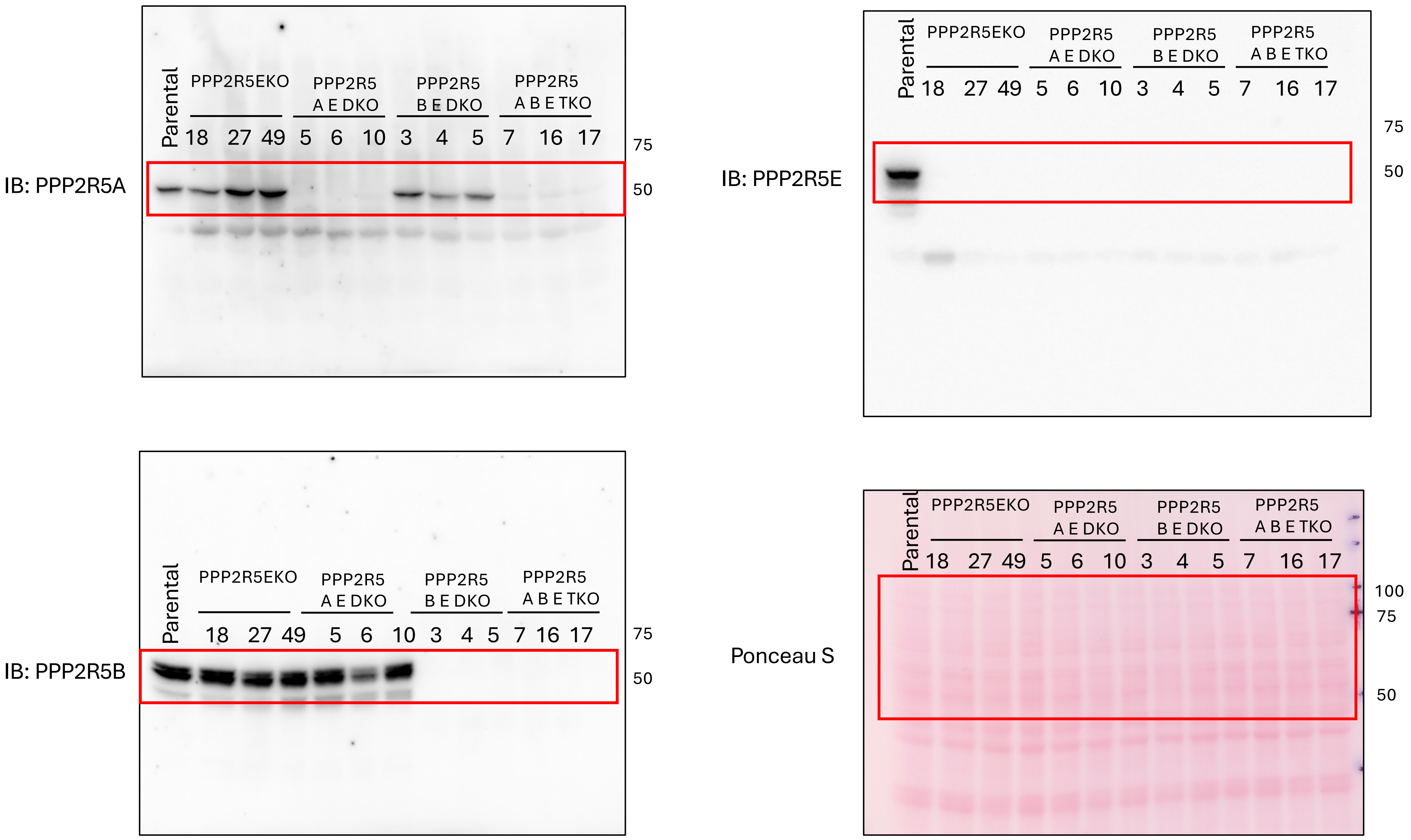

Supplement: Supplementary file 17 — Figures EV1-EV5 Source Data [file 44318_2026_785_MOESM17_ESM.zip › Source Data for Expanded View and Appendix/Appendix Figure Source Data.zip!/Appendix Figure Source Data/Appendix Figure S1/S1B/Appendix Figure S1B.pdf]
